# Early-life growth and emotional, behavior and cognitive outcomes in childhood and adolescence in the EU child cohort network: individual participant data meta-analysis of over 109,000 individuals

**DOI:** 10.1016/j.lanepe.2025.101247

**Published:** 2025-02-26

**Authors:** Romy Gonçalves, Sophia Blaauwendraad, Demetris Avraam, Andrea Beneíto, Marie-Aline Charles, Ahmed Elhakeem, Joaquin Escribano, Louise Etienne, Gonzalo García-Baquero Moneo, Ana Gonçalves Soares, Jasmin de Groot, Veit Grote, Dariusz Gruszfeld, Kathrin Guerlich, Monica Guxens, Barbara Heude, Berthold Koletzko, Aitana Lertxundi, Manuel Lozano, Hanan El Marroun, Rosie McEachan, Angela Pinot de Moira, Gillian Santorelli, Katrine Strandberg-Larsen, Muriel Tafflet, Chloe Vainqueur, Elvira Verduci, Martine Vrijheid, Marieke Welten, John Wright, Tiffany C. Yang, Romy Gaillard, Vincent W.V. Jaddoe

**Affiliations:** a The Generation R Study Group, Erasmus University Medical Center, Rotterdam, the Netherlands; b Department of Paediatrics, Sophia's Children's Hospital, Erasmus University Medical Center, Rotterdam, the Netherlands; c Department of Public Health, Section of Epidemiology, University of Copenhagen, Copenhagen, Denmark; d Catalan Institute of Health-Camp de Tarragona, Tarragona, Spain; e Epidemiology and Environmental Health Joint Research Unit, FISABIO−Universitat Jaume I−Universitat de València, Valencia, Spain; f Université de Paris, Centre of Research in Epidemiology and Statistics, Inserm, Inrae, Paris, France; g Ined, Inserm, EFS Joint Unit Elfe, Paris, France; h MRC Integrative Epidemiology Unit at the University of Bristol, Bristol, United Kingdom; i Population Health Sciences, Bristol Medical School, University of Bristol, Bristol, United Kingdom; j Department of Paediatrics, Sant Joan Reus Hospital, University Rovira i Virgili, IISPV, Reus, Spain; k Centre Hospitalier Chretien St. Vincent, Rocourt, Liège-Rocourt, Belgium; l Faculty of Biology, University of Salamanca, Salamanca, Spain; m Biogipuzkoa Health Research Institute, Donostia, Spain; n Division of Metabolic and Nutritional Medicine, Department of Paediatrics, Dr. von Hauner Children's Hospital, LMU University Hospital, LMU Munich, Munich, Germany; o German Center for Child and Adolescent Health, Munich, Germany; p Neonatal Department and Neonatal Intensive Care Unit, Children's Memorial Health Institute, Warsaw, Poland; q ISGlobal, Barcelona, Spain; r ICREA, Barcelona, Spain; s Universitat Pompeu Fabra, Barcelona, Spain; t Spanish Consortium for Research on Epidemiology and Public Health (CIBERESP), Instituto de Salud Carlos III, Madrid, Spain; u Department of Child and Adolescent Psychiatry/Psychology, Erasmus MC, University Medical Centre, Rotterdam, the Netherlands; v Department of Preventive Medicine and Public Health, Faculty of Medicine, University of the Basque Country (UPV/EHU), Leioa, Spain; w Preventive Medicine and Public Health, Food Sciences, Toxicology and Forensic Medicine Department, Universitat de València, Valencia, Spain; x Department of Psychology, Education and Child Studies, Erasmus School of Social and Behavioural Science, Erasmus University Rotterdam, Rotterdam, the Netherlands; y Bradford Institute for Health Research, Bradford Teaching Hospitals NHS Foundation Trust, Bradford, BD9 6RJ, United Kingdom; z Imperial College London, United Kingdom; aa Department of Paediatrics, Vittore Buzzi Children's Hospital, University of Milan, Milan, Italy

**Keywords:** Preterm birth, Birth weight, Infant growth, Behavior, Cognition, Attention-deficit hyperactivity disorder, Autism spectrum disorder, Intelligence quotient

## Abstract

**Background:**

Fetal and infant development might be critical for cognitive outcomes and psychopathology later in life. We assessed the associations of birth characteristics and early life growth with behavior and cognitive outcomes from childhood to adolescence.

**Methods:**

We used harmonized data of 109,481 children from 8 European birth cohorts. Birth weight, gestational age, and body mass index (BMI) tertiles at the age of 2 years were used as the exposure variables. Outcomes included internalizing and externalizing problems and attention-deficit hyperactivity disorder (ADHD), autism spectrum disorder (ASD), and non-verbal intelligence quotient (Non-verbal IQ) in childhood (4–10 years), early adolescence (11–16 years), and late adolescence (17–20 years). We used 1-stage individual participant data meta-analyses using generalized linear models.

**Findings:**

A one-week older gestational age was associated with lower scores for internalizing problems (difference −0·48 (95% CI: −0·59, −0·37)), externalizing problems (difference −0·34 (95% CI: −0·44, −0·23)), and ADHD symptoms (difference −0·38 (95% CI: −0·49, −0·27)), and with higher scores for non-verbal IQ (difference 0·65 (95% CI: 0·41, 0·89)). As compared to term birth, preterm birth was associated with higher internalizing problems (difference 3·43 (95% CI: 2·52, 4·33)) and externalizing problems (difference 2·31 (95% CI: 1·16, 3·46)), ADHD symptoms (difference 4·15 (95% CI: 3·15, 5·16)), ASD symptoms (difference 3·23 (95% CI: 0·37, 6·08)), and lower non-verbal IQ (difference −5·44 (95% CI: −7·44, −3·44)). Small size for gestational age at birth (SGA) in comparison with appropriate size for gestational age (AGA) was associated with higher ADHD symptoms (difference 4·88 (95% CI: 3·87, 5·90)) and lower Non-verbal IQ (difference −7·02 (95% CI: −8·84, −5·21)). Large size for gestational age at birth was associated with lower ADHD symptoms (difference −1·09 (95% CI: −1·73, 0·45)) and higher non-verbal IQ (difference 2·47 (95% CI: 0·77, 4·18)). Explorative analyses showed that as compared to children with an appropriate size for gestational age at birth and a normal BMI at the age of 2 years, children born SGA who remained small at 2 years had the lowest non-verbal IQ score (difference −8·14 percentiles (95% CI: −11·89, −4·39)).

**Interpretation:**

Both fetal and early childhood growth are associated with emotional, behavioral and cognitive outcomes throughout childhood and adolescence. Compensatory infant growth might partly attenuate the adverse effects of suboptimal fetal growth. Future studies are needed to identify the potential for optimizing mental health outcomes in new generations by improving early-life growth.

**Funding:**

This project received funding from the 10.13039/501100007601European Union's Horizon 2020 research and innovation programme (LIFECYCLE, grant agreement No 733206, 2016; EUCAN-Connect grant agreement No 824989; ATHLETE, grant agreement No 874583).

**Research in context**

**Evidence before this study**

Pubmed was searched for articles regarding the associations of fetal and infant growth and behavior and cognitive outcomes in later life. The terms used were “Fetal growth”, “Birth weight”, “growth”, “weight”, “Gestational age”, “behavior”, “cognition”, “attention-deficit hyperactivity disorder” (ADHD), “autism spectrum disorder” (ASD), “IQ”, “internalizing problems”, and “externalizing problems”. We repeated these searches throughout the research period between November 2022 and August 2024. Most studies were performed in small numbers and predominantly among children born preterm or with low birth weight. Among studies based in the general population, preterm birth, low birth weight, and small size for gestational age at birth were associated with adverse behavior and cognitive outcomes. Literature regarding fetal and infant growth combinations in a general population is scarce and the magnitude of effects remains unclear.

**Added value of this study**

The current study, including individual level participant data of 109,481 children between 4 and 20 years old presenting data from different European birth cohorts is the largest to assess the associations of fetal and infant growth patterns related to both behavior and cognitive outcomes. We focused on the associations of birth characteristics and size in early childhood with behavior and cognitive outcomes across childhood and adolescence.

**Implications of all the available evidence**

We observed that adverse birth characteristics are associated with higher scores for internalizing problems, externalizing problems, and ADHD symptoms. Small size for gestational age at birth, which is not followed by catch up-growth in infancy, was associated with lower cognitive outcomes. Both fetal and early childhood growth are associated with emotional, behavioral and cognitive outcomes throughout childhood and adolescence. In children born with adverse birth characteristics, optimizing infant growth might contribute to the prevention of psychopathology and poor cognitive outcomes later in life.

## Introduction

Central nervous system development starts in the very early stages of pregnancy and continues throughout life.^1^^,^^2^ Disruptions in optimal fetal development are associated with cognitive and psychopathology outcomes in adulthood.^3^^,^^4^ Previous studies have reported associations between low birth weight and preterm birth with an increased risk of internalizing and externalizing problems, attention-deficit hyperactivity disorder (ADHD), autism spectrum disorder (ASD), and lower intelligence quotient (IQ).^4^, ^5^, ^6^, ^7^, ^8^, ^9^ These associations might not be restricted to these extremes and be present across the full spectrum.^9^, ^10^, ^11^, ^12^ Also, birth weight and gestational age at birth, may not be the causal factor per se, but may reflect fetal growth adaptations and are the starting point of infant development.^9^^,^^13^ Children born preterm or with a low birth weight might have been exposed to different exposures and are known to follow different postnatal growth patterns as compared to their term born and normal birth weight peers.^14^^,^^15^ It is not known whether compensatory infant growth and development leads to better neurodevelopmental outcomes in children born preterm or with a low birth weight. Large-scale studies examining the associations of birth weight combined with subsequent infant growth with behavior and cognitive outcomes are scarce.^16^^,^^17^ One study conducted in Belarus among more than 11,000 children showed that both increased birth weight and weight gain during infancy was associated with higher IQ.^16^ Identification of birth characteristics and early life growth patterns at risk for adverse long-term outcomes could identify windows of opportunity for novel strategies, such as monitoring behavior and cognitive development in children born preterm or of low birth weight and strategies to optimize neurodevelopmental outcomes in children at risk.

We hypothesized that both adverse birth outcomes, birth weight as the end point of fetal development, and early childhood growth might be critical for cognitive and psychopathology later in life. Compensatory early childhood growth in children born after suboptimal fetal growth might be beneficial for emotional, behavioral and cognitive development in children.^16^^,^^18^ We assessed the associations of birth characteristics and early-life growth with behavior and cognitive outcomes in an individual participant data (IPD) meta-analysis among 109,481 children from 8 European cohort studies from pregnancy onwards. We used the infrastructure of the EU Child Cohort Network, which brings together data from pregnancy and childhood from European cohort studies and focused on the outcomes assessed in the age ranges of 4–20 years.^19^

## Methods

### Study design and participating cohorts

We included 109,481 mother-child pairs from eight European population-based birth cohorts collaborating in the EU Child Cohort Network established by the LifeCycle Project.^19^ Cohorts included the Avon Longitudinal Study of Parents and Children (ALSPAC, United Kingdom),^20^, ^21^, ^22^, ^23^ Born in Bradford (BiB United Kingdom),^24^^,^^25^ European Childhood Obesity Project Trial (CHOP; Germany, Belgium, Italy, Poland & Spain),^26^^,^^27^ the Danish National Birth Cohort (DNBC, Denmark),^28^ the French Longitudinal Study since Childhood (Etude Longitudinale Française depuis l'Enfance) (ELFE, France),^29^ Etude sur les Déterminants de la santé de l'Enfant Nancy & Poitiers (EDEN-Nancy & EDEN-Poitiers, France),^30^ the Generation R Study (GenerationR, The Netherlands),^31^ The INfancia y Medio Ambiente Project (INMA, Spain).^32^ All cohorts participated in either the LifeCycle—EU Child Cohort Network or the Advancing Tools for Human Early Lifecourse Exposome Research and Translation (ATHLETE) Project.^19^^,^^33^ The populations in the cohorts used in this study were recruited between 1991 and 2011. Information on the profile and design of each study is provided in Text S1. Cohorts were eligible for this study if they had the following exposure data: birth weight, gestational age at birth and weight and height at about 24 months of age, and information on either internalizing and externalizing problems, autism spectrum disorder (ASD), attention-deficit hyperactivity disorder (ADHD) or non-verbal intelligence quotient (Non-verbal IQ) in children or adolescents aged 4–20 years. The flowchart of the current study population is given in Figures S1 and S2. All cohorts received approval from their local institutional review boards and all study participants gave written informed consent to participate in the respective cohorts and secondary data analyses. Data were harmonized across these cohorts. The harmonized data were kept within each institution and analyzed through the R-based federated data analysis platform DataSHIELD.^34^, ^35^, ^36^ This study followed the Strengthening the Reporting of Observational Studies in Epidemiology (STROBE) reporting guideline.^37^

### Birth characteristics and early childhood growth measures

Birth characteristics were collected from midwife and hospital records in all cohorts. We used gestational age continuous (weeks) and categorized as preterm (<37 weeks), term (37–42 weeks), and post term (>42 weeks) birth. We used birth weight continuous (per 500 g) and categorized as low birth weight (<2500 g), normal birth weight (2500–4500 g), and high birth weight (>4500 g). Size for gestational age was defined using the WHO fetal growth charts.^38^ Small for gestational age (SGA) was defined as weight being below the 5th percentile, appropriate size for gestational age (AGA) was defined as weight being between the 5th and the 95th percentile and large for gestational age (LGA) was defined as weight being above the 95th percentile. Early childhood weight and height was assessed by using an age interval between 18 and 30 months, in which the measurement closest to 24 months was selected, and body mass index (BMI) was calculated (kg/m^2^). Because age intervals were constructed and healthy BMI ranges differ per age, we used BMI tertiles in our population, defining the lowest tertile as low BMI, the middle tertile as normal BMI, and the third tertile as high BMI.

### Emotional, behavioral and cognitive outcomes

Information about the emotional, behavior and cognitive outcomes in the EU Child Cohort Network has been described previously.^39^ Internalizing and externalizing problems, ADHD symptoms, ASD symptoms and Non-verbal IQ were assessed in the cohorts at different ages and with different instruments. Briefly, internalizing and externalizing problems were measured by using the Strengths and Difficulties Questionnaire (SDQ)^40^ or the Child Behavior Checklist (CBCL)^41^ for both instruments higher scores indicate more emotional and behavioral problems. Both are parent-reported, except for in DNBC cohort for late adolescents. ADHD symptoms were measured by using the hyperactivity subscale of the SDQ,^40^ the Conner's Parent Rating Scale-Revised Short Form (CPRS-R:S)^42^ or the Diagnostic and Statistical Manual of Mental Disorders IV (DSM-IV),^43^ but only in INMA cohort. The SDQ and the CPRS-R:S are both parent-reported, whereas the DSM-IV questionnaire was filled out by teachers. For all instruments higher scores, indicate more ADHD symptoms. ASD symptoms were measured by using the Social Responsiveness Scale (SRS; GENR)^44^ or the Childhood Autism Spectrum Test (CAST; INMA).^45^ For both tests, higher scores indicate more ASD symptoms. ASD symptoms were assessed with either parent-reported questionnaires or an interview conducted at the research facility. Last, non-verbal IQ was mainly assessed using the Snijders-Oomen Niet-verbale intelligentie Test- Revisie (SON-R 2.5–7)^46^ and the Wechsler Preschool and Primary Scale of Intelligence Third Edition (WPPSI-III).^47^ A global non-verbal IQ can be established using these tests. The tests were administered during a visit to the research facility and either handwritten or computer based. In each separate cohort at each age point internal percentiles were constructed as part of the EU Child Cohort Network data harmonization.^19^^,^^39^ A detailed description of the instruments used in the cohorts is given in Text S2. In each separate cohort at each age point internal percentiles were constructed as part of the EU Child Cohort Network data harmonization.^19^ Tables S2 and S3 show the descriptive statistics and distribution of emotional, behavior and cognitive outcomes in each cohort. We constructed age intervals (childhood: 4·0–10·9 years; early adolescence: 11·0–16·9 years; and late adolescence: 17·0–20·0 years) for internalizing problems, externalizing problems and for ADHD symptoms. ASD symptoms and Non-verbal IQ scores were only available in childhood (4·0–10·9 years). When more than one assessments were available in one age interval, we used the assessments at the oldest age.

### Covariates

A set of covariates was selected a priori, based on literature search, and is presented in a Directed Acyclic Graph (DAG) in Figure S3. The maternal covariates included age at delivery (continuous, years), education level based on the International Standard Classification of Education 97/2011 (ISCED- 97/2011) (categorical, low/medium/high), ethnicity (categorical, Western/non-Western/Mixed), pre-pregnancy BMI (continuous, kg/m^2^), parity (categorical, nulliparous/multiparous), and smoking during pregnancy (binary, yes/no). The maternal education level was based on the highest completed education at the time of delivery. Child covariates used: sex (binary, boy/girl) and age at outcome measurement (continuous, years).

### Statistical analysis

First, we described the participant characteristics per cohort and for all cohorts combined. Second, we conducted a 1-stage individual participant data meta-analysis using generalized linear models to analyze the associations of birth characteristics and the behavior and cognitive outcomes in different age windows. Main exposures included birth characteristics included gestational age at birth and birth weight both continuously and categorized. Outcomes included internalizing problems, externalizing problems and ADHD symptoms in childhood, early adolescence, and late adolescence, and ASD symptoms and Non-verbal IQ scores in childhood. Third, we assessed the associations of nine combinations of different gestational age adjusted sizes at birth (SGA, AGA, LGA) and early childhood BMI tertiles as growth patterns, with behavior and cognitive outcomes. We assessed the statistical interaction between size at birth and early childhood BMI for these associations. All models were adjusted for sex, age at outcome measurement and cohort in the basic models, and additionally for maternal age, education, pre-pregnancy BMI, parity, and smoking during pregnancy in the main model. We did not adjust for ethnicity since most cohorts had one predominant ethnicity group. To correct for multiple testing, we used the Bonferroni correction method taking account for five outcomes and specified multiple-test corrected p-values as <0·01. As a sensitivity analysis, we performed a 2-stage individual participant meta-analysis of the main models, by calculating the effect estimates in each cohort separately. Subsequently the combined estimate across all cohorts was calculated by random effects meta-analysis using a restricted maximum likelihood estimation (REML). Missing values for covariates were imputed using multiple imputation on cohort level by the fully conditional specification method, and pooled results from 5 imputed datasets were reported. A detailed description is given in Text S3. The percentage of missing values ranged from 0 to 27·6% (DNBC, maternal education level). CHOP was not included in the categorical analyses of birth weight and gestational age, since preterm born and low birth weight children were specifically excluded in this cohort. DataSHIELD withholds results if the number of data in a cohort is less than 30 for data-security reasons. As a result, GenerationR and INMA were not included in the birth weight categories analyses for early adolescence internalizing and externalizing problems. When constructing the nine growth patterns some cohorts had few or no data in any of the nine categories, therefore the analyses could only be performed in childhood for the internalizing and externalizing problems, ADHD symptoms and Non-verbal IQ score. All analysis were performed using DataSHIELD dsBaseClient package version 6·3·0.^34^, ^35^, ^36^

### Ethics approval

Procedures for ethical approval are given in Text S4.

### Role of the funding source

The funding sources did not play any role in the study design, the data collection, the data analysis, the interpretation of results, the writing of the report and in the decision to submit the study for publication.

## Results

### Subject characteristics

The descriptives of this study population for each cohort are given in Table 1 and Table 2. The behavior and cognitive outcomes were measured at different ages and with different frequency in the cohorts. Internalizing and externalizing problems scores in childhood were available for 109,481. ASD symptoms scores were only available in GenerationR and INMA and available for 8860 children solely in childhood. Tables S1–S3 show the descriptives and missing value information of the study populations and outcome measurements in age intervals.

**Table 1:** Subject characteristics.

	All cohorts (n = 109,481)	ALSPAC, UK (n = 10,511)	BIB, UK (n = 2346)	CHOP, Germany (n = 723)	DNBC, Denmark (n = 74,893)	EDEN, France (n = 1259)	ELFE, France (n = 10,889)	Generation R, Netherlands (n = 7331)	INMA, Spain (n = 1529)
Birth years	1991–2010	1991–1992	2008–2010	2002–2004	1998–2002	2003–2006	2004–2005	2002–2006	2004–2008
**Maternal**									
Age in years, median (IQR)	29·9 (26·8, 33·0)	26·0 (23·0, 30·0)	28·0 (24·0, 32·0)	31·0 (28·0, 34·0)	30·0 (27·0, 33·0)	30·0 (27·0, 33·0)	31·0 (28·0, 34·0)	32·0 (28·0, 35·0)	32·0 (30·0, 35·0)
Pre-pregnancy BMI in kg/m^2^, median (IQR)	22·6 (20·6, 25·3)	22·2 (20·5, 24·4)	24·9 (21·8, 28·8)	22·3 (20·4, 25·5)	22·6 (20·7, 25·4)	22·2 (20·1, 25·4)	22·1 (20·2, 25·1)	22·7 (20·8, 28·7)	22·6 (20·8, 25·1)
Education, n (%)									
High	39,010 (42·1)	1430 (14·3)	629 (28·6)	223 (30·9)	26,926 (49·7)	753 (60·0)	7381 (67·7)	3216 (48·0)	552 (36·7)
Medium	34,945 (37·8)	6927 (69·0)	304 (13·8)	365 (50·6)	20,023 (36·9)	446 (35·6)	3022 (27·7)	2032 (30·4)	626 (41·5)
Low	18,640 (20·1)	1670 (16·7)	1263 (57·5)	133 (18·4)	7255 (13·4)	55 (4·4)	486 (4·5)	1448 (21·6)	330 (21·8)
Ethnicity, n (%)									
Western	16,248 (69·0)	NA	703 (30·0)	NA	NA	1096 (99·0)	8718 (83·3)	4290 (60·2)	1441 (94·6)
Non-Western	5834 (24·7)	NA	1579 (67·4)	NA	NA	6 (0·5)	1018 (9·8)	2150 (30·1)	81 (5·4)
Mixed	1481 (6·3)	NA	60 (2·6)	NA	NA	5 (0·5)	722 (6·9)	694 (9·7)	NA
Parity									
0	51,461 (48·2)	4553 (44·9)	837 (36·3)	361 (50·0)	36,030 (48·1)	583 (46·4)	4987 (46·3)	3992 (56·4)	818 (56·0)
1 or more	55,310 (51·8)	5538 (54·1)	1467 (63·7)	361 (50·0)	38,861 (51·9)	674 (53·6)	5782 (53·7)	3102 (43·6)	643 (44·0)
Smoking during pregnancy, n (%)	25,099 (23·6)	2404 (25·7)	228 (9·7)	202 (28·0)	18,647 (25·3)	274 (21·8)	1803 (16·6)	1593 (25·0)	448 (29·7)
**Birth**									
Gestational age in weeks, median (IQR)	40·2 (39·1, 41·0)	40·4 (39·4, 41·4)	39·9 (38·7, 40·7)	40·6 (39·6, 41·6)	40·1 (39·0, 41·0)	40·4 (39·4, 40·4)	39·9 (39·0, 40·7)	40·7 (39·6, 41·6)	40·0 (39·0, 40·9)
Birth weight in grams, (median, IQR)	3515 (3167, 3855)	3440 (3110, 3760)	3200 (2840, 3560)	3300 (3050, 3550)	3580 (3220, 3930)	3310 (3000, 3620)	3340 (3040, 3640)	3430 (3070, 3774)	3290 (3000, 3550)
Sex of the child, n *(%)*									
Boy	53,180 (50·1)	5403 (51·2)	1169 (50·7)	340 (47·0)	37,026 (49·4)	667 (53·0)	5599 (51·4)	3694 (50·4)	781 (51·0)
Girl	52,998 (49·3)	5108 (48·8)	1177 (49·3)	383 (53·0)	37,866 (50·6)	592 (47·0)	5287 (48·6)	3637 (49·6)	748 (49·0)
**Early childhood BMI**									
Age in months, median (IQR)	24·0 (23·1, 24·4)	25·0 (25·0, 25·0)	24·0 (23·0, 25·0)	24·0 (23·0, 24·0)	18·0 (18·0, 18·0)	24·0 (23·0, 24·0)	24·0 (23·0, 24·0)	24·0 (24·0, 25·0)	23·0 (18·0, 24·0)
BMI in kg/m^2^, median (IQR)	16·2 (15·4, 17·1)	16·7 (15·9, 17·6)	16·5 (15·6, 17·5)	15·9 (15·2, 16·8)	16·6 (15·7, 17·6)	16·0 (15·1, 16·8)	15·9 (15·0, 16·8)	16·5 (15·7, 17·4)	16·4 (15·5, 17·3)

Values are median (IQR), or number (valid %). Characteristics are based on observed not imputed data. BMI: body mass index. The population sample comprises of participants with complete data on birth weight and at least one outcome measurement available.

**Table 2:** Emotional, behavior and cognitive characteristics.

	All cohorts (n = 109,481)	ALSPAC, UK (n = 10,511)	BIB, UK (n = 2346)	CHOP, Germany (n = 723)	DNBC, Denmark (n = 74,893)	EDEN, France (n = 1259)	ELFE, France (n = 10,889)	Generation R, Netherlands (n = 7331)	INMA, Spain (n = 1529)
**Internalizing problems percentiles,** median (IQR)									
Childhood	47·0 (8·0, 67·8)	44·0 (22·0, 72·0)	51·0 (23·0, 75·0)	42·5 (22·8, 68·9)	47·0 (1·0, 64·0)	48·0 (19·0, 78·0)	46·0 (12·0, 73·0)	51·5 (28·3, 77·9)	47·0 (19·0, 73·0)
Early adolescence	43·3 (23·9, 71·3)	45·0 (24·0, 73·0)	NA	40·9 (22·4, 69·2)	43·0 (24·0, 71·0)	NA	NA	60·4 (28·3, 88·1)	40·0 (14·0, 68·0)
Late adolescence	47·9 (24·0, 68·2)	46·0 (24·0, 74·0)	NA	NA	48·0 (24·0, 68·0)	NA	NA	NA	NA
**Externalizing problems percentiles,** median (IQR)									
Childhood	44·9 (17·3, 71·1)	49·0 (21·0, 72·0)	45·0 (25·0, 74·0)	41·6 (22·6, 71·3)	43·0 (14·0, 70·0)	52·0 (25·0, 75·0)	45·0 (22·0, 70·0)	53·5 (26·2, 79·0)	44·0 (19·8, 72·0)
Early adolescence	40·7 (20·3, 74·5)	44·0 (22·0, 72·0)	NA	46·4 (22·7, 76·6)	40·0 (20·0, 75·0)	NA	NA	51·9 (26·2, 74·6)	47·0 (20·0, 69·0)
Late adolescence	44·0 (17·8, 68·1)	43·0 (14·0, 70·0)	NA	NA	44·0 (18·0, 68·0)	NA	NA	NA	NA
**ADHD score percentiles,** median (IQR)									
Childhood	43·9 (20·3, 73·2)	48·0 (13·0, 66·0)	NA	49·1 (20·2, 72·3)	43·0 (22·0, 75·0)	53·0 (23·0, 78·0)	40·0 (14·0, 69·0)	50·1 (28·2, 75·4)	47·0 (24·0, 73·0)
Early adolescence	46·2 (4·0, 68·1)	47·0 (19·0, 74·0)	NA	50·0 (23·0, 70·4)	46·0 (1·0, 67·0)	NA	NA	NA	50·0 (24·0, 74·0)
Late adolescence	38·9 (21·8, 70·2)	36·0 (18·0, 75·0)	NA	NA	39·0 (22·0, 70·0)	NA	NA	NA	NA
**ASD score percentiles,** median (IQR)									
Childhood	53·0 (24·9, 73·7)		NA	NA	NA	NA	NA	54·2 (26·3, 75·1)	48·0 (19·0, 68·0)
**Non-verbal IQ score percentiles,** median (IQR)									
Childhood	52·7 (25·4, 74·8)	49·0 (25·0, 75·0)	NA	52·1 (27·0, 74·2)	NA	52·0 (27·0, 74·0)	NA	58·7 (26·4, 75·3)	47·0 (21·0, 72·0)

Values are median (IQR). Characteristics are based on observed not imputed data. ADHD: attention-deficit hyperactivity disorder, ASD: autism spectrum disorder, Non-verbal IQ: non-verbal intelligence. Childhood measurements are between 4 and 10 years old, early adolescence measurements are between 11 and 16 years old and late adolescence measurements are between 17 and 20 years old.

### Birth outcomes, emotional, behavior and cognitive outcomes

Table 3 shows that one week older gestational age at birth and higher birth weight were associated with lower scores for internalizing and externalizing problems in childhood and early adolescence (all p-values < 0·01). In late adolescence, the associations of gestational age at birth with externalizing problems were reversed, suggesting that one week older gestational age at birth was associated with higher externalizing problems symptoms (p-value < 0·01). As compared to children born term, those born preterm had increased scores for internalizing behavior in childhood (3·43 percentiles (95% Confidence Interval (CI) 2·52, 4·33)), early adolescence (1·77 percentiles (95% CI: 0·57, 2·96)), and late adolescence (1·20 percentiles (95% CI: 0·10, 2·30)), and increased scores for externalizing behavior in childhood (2·28 percentiles (95% CI: 1·43, 3·13)) and early adolescence (2·31 percentiles (95% CI: 1·16, 3·46)). This latter association reversed in late adolescence. The associations between low birth weight with internalizing and externalizing scores tended to be similar as for preterm birth suggesting that as compared to normal birth weight, low birth weight is associated with higher scores for internalizing and externalizing behavior in childhood and early adolescence, whereas in late adolescence these association were absent or inversed.

**Table 3:** Birth characteristics and scores for internalizing problems and externalizing problems.

	Internalizing problems Difference in percentiles (95% CI)			Externalizing problems Difference in percentiles (95% CI)		
	Childhood (n = 89,004)	Early adolescence (n = 54,978)	Late adolescence (n = 47,522)	Childhood (n = 88,975)	Early adolescence (n = 54,980)	Late adolescence (n = 47,523)
Gestational age at birth, weeks	−0·48 (−0·59, −0·37)∗∗ n = 109,481	−0·21 (−0·36, −0·07)∗∗ n = 94,987	−0·13 (−0·27, 0·01) n = 85,404	−0·34 (−0·44, −0·23)∗∗ n = 109,481	−0·31 (−0·45, −0·17)∗∗ n = 94,987	0·26 (0·12, 0·40)∗∗ n = 85,404
Gestational age in categories, weeks						
<37	3·43 (2·52, 4·33)∗∗ n = 4780	1·77 (0·57, 2·96)∗∗ n = 2873	1·20 (0·10, 2·30)∗ n = 2668	2·28 (1·43, 3·13)∗∗ n = 4777	2·31 (1·16, 3·46)∗∗ n = 2873	−2·27 (−3·42, −1·13)∗∗ n = 2668
37–42	*Reference* n = *78,605*	*Reference* n = *48,345*	*Reference* n = *42,080*	*Reference* n = *78,581*	*Reference* n = *48,347*	*Reference* n = *42,080*
>42	0·78 (−0·11, 1·68) n = 4938	0·47 (−0·64, 1·58) n = 3320	0·34 (−0·73, 1·42) n = 2774	−0·75 (−1·59, 0·09) n = 4935	−0·69 (−1·75, 0·38) n = 3320	0·13 (−0·99, 1·25) n = 2775
Birth weight, per 500 gr	−0·88 (−1·07, −0·69)∗∗ n = 109,481	−0·59 (−0·83, −0·35)∗∗ n = 94,987	−0·41 (−0·64, −0·19)∗∗ n = 85,404	−1·09 (−1·26, −0·91)∗∗ n = 109,481	−1·19 (−1·42, −0·96)∗∗ n = 94,987	0·14 (−0·09, 0·37) n = 85,404
Birth weight in categories, grams						
<2500	3·20 (2·20, 4·20)∗∗ n = 3887	1·92 (0·52, 3·32)∗∗ n = 2054	1·08 (−0·19, 2·36) n = 1954	3·77 (2·83, 4·71)∗∗ n = 3885	3·60 (2·26, 4·95)∗∗ n = 2054	−1·92 (−3·25, −0·60)∗∗ n = 1954
2500–4500	*Reference* n = *81,751*	*Reference* n = *50,179*	*Reference* n = *43,829*	*Reference* n = *81,725*	*Reference* n = *50,181*	*Reference* n = *43,830*
>4500	−0·20 (−1·39, 1·00) n = 2685	−0·10 (−1·55, 1·34) n = 1949	1·55 (0·20, 2·90)∗ n = 1739	−1·72 (−2·85, −0·60)∗∗ n = 2683	−1·74 (−3·13, −0·35)∗ n = 1949	0·13 (−1·28, 1·54) n = 1739
Cohorts	ALSPAC, BIB, CHOP^a^, DNBC, EDEN, ELFE, GENR, INMA	ALSPAC, CHOP^a^, DNBC, GENR^b^^c^, INMA^b^^c^	ALSPAC, DNBC	ALSPAC, BIB, CHOP^a^, DNBC, EDEN, ELFE, GENR^b^^c^, INMA^b^^c^	ALSPAC, CHOP^a^, DNBC, GENR^b^^c^, INMA^b^^c^	ALSPAC, DNBC

CI: Confidence interval, SDS: standard deviation score, ∗p value < 0·05, ∗∗p value < 0·01. Childhood measurements are between 4 and 10 years old, early adolescence measurements are between 11 and 16 years old and late adolescence measurements are between 17 and 20 years old. Values are regression coefficients (95% confidence interval) obtained from one stage meta-analysis and reflect the differences in internalizing problems (percentiles) or externalizing problems (percentiles) for birth characteristics from pooled multiple imputed data. The confounder model is adjusted for sex, age at outcome measurement, cohort effect, maternal age at birth, maternal educational level, parity, pre-pregnancy body mass index, and smoking during pregnancy.

a CHOP cohort is not included in the birth weight and gestational age categories due to missing data on preterm born and low birth weight children.

b GenerationR and INMA are not included in the analyses of birth weight categories and internalizing or externalizing problems in early adolescence due to a small number of participants and DataSHIELD data security protections.

c GenerationR was due to the same issue not included in the analysis of gestational age and sex adjusted birth weight categories with internalizing or externalizing problems in early adolescence.

Table 4 shows that one week older gestational age at birth and higher birth weight were associated with lower scores for ADHD symptoms in childhood and early adolescence, and with a higher Non-verbal IQ in childhood (all p-values < 0·01). Higher birth weight was also associated with lower ASD symptoms in childhood (p-value < 0·01). In late adolescence the associations of gestational age at birth with ADHD symptoms were reversed, suggesting that one week older gestational age at birth was associated with higher ADHD symptoms (p-value < 0·01). As compared to children born term, those born preterm had increased scores for ADHD symptoms in childhood (2·51 percentiles (95% CI: 1·61, 3·40)) and early adolescence (2·33 percentiles (95% CI: 1·16, 3·50)). This association reversed in late adolescence. The associations between low birth weight with ADHD symptoms tended to be similar as for preterm birth. Not preterm birth, but low birth weight was associated with ASD symptoms (3·23 percentiles (95% CI: 0·37, 6·08)). Preterm birth and low birth weight were also associated with a −3·41 percentiles (95% CI: −5·46, −1·36) and −5·44 percentiles (95% CI: −7·44, −3·44) lower Non-verbal IQ in childhood respectively. Although not consistent, tendencies for opposite associations were observed for high birth weight. The corresponding basic models showed similar associations and are shown in Tables S4 and S5.

**Table 4:** Birth characteristics and scores for ADHD symptoms, ASD symptoms and Non-verbal Intelligence scores.

	ADHD symptoms Difference in percentiles (95% CI)			ASD symptoms Difference in percentiles (95% CI)	Non-verbal IQ Difference in percentiles (95% CI)
	Childhood (n = 84,782)	Early adolescence (n = 55,097)	Late adolescence (n = 47,522)	Childhood (n = 6420)	Childhood (n = 16,453)
Gestational age at birth, weeks	−0·38 (−0·49, −0·27)∗∗ n = 107,135	−0·28 (−0·42, −0·14)∗∗ n = 87,656	0·21 (0·07, 0·36)∗∗ n = 85,404	−0·33 (−0·69, 0·02) n = 8860	0·65 (0·41, 0·89)∗∗ n = 21,353
Gestational age in categories, weeks					
<37	2·51 (1·61, 3·40)∗∗ n = 4522	2·33 (1·16, 3·50)∗∗ n = 2878	−1·74 (−2·89, −0·58)∗∗ n = 2668	0·60 (−2·60, 3·79) n = 279	−3·41 (−5·46, −1·36)∗∗ n = 780
37–42	*Reference* n = *74,977*	*Reference* n = *48,461*	*Reference* n = *42,080*	*Reference* n = *5392*	*Reference* n = *13,771*
>42	−0·73 (−1·61, 0·16) n = 4680	−0·42 (−1·50, 0·67) n = 3318	0·22 (−0·91, 1·35) n = 2774	0·94 (−1·11, 2·99) n = 749	0·60 (−0·99, 2·18) n = 1390
Birth weight, per 500 gr	−1·13 (−1·31, −0·95)∗∗ n = 107,135	−1·19 (−1·42, −0·95)∗∗ n = 87,656	0·14 (−0·09, 0·38) n = 85,404	−0·81 (−1·41, −0·22)∗∗ n = 8860	2·23 (1·82, 2·63)∗∗ n = 21,353
Birth weight in categories, grams					
<2500	4·15 (3·15, 5·16)∗∗ n = 3531	3·51 (2·15, 4·87)∗∗ n = 2078	−1·95 (−3·29, −0·61)∗∗ n = 1954	3·23 (0·37, 6·08)∗ N = 353	−5·44 (−7·44, −3·44)∗∗ N = 821
2500–4500	*Reference* n = *78,023*	*Reference* n = *50,626*	*Reference* n = *43,829*	*Reference* n = *5932*	*Reference* n = *14,845*
>4500	−1·45 (−2·62, −0·28)∗ n = 2625	−1·54 (−2·95, −0·13)∗ n = 1953	−0·01 (−1·43, 1·41) n = 1739	3·02 (−1·52, 7·56) n = 135	4·85 (1·44, 8·26)∗∗ n = 275
Cohorts	ALSPAC, CHOP^a^, DNBC, EDEN, ELFE, GENR, INMA	ALSPAC, CHOP^a^, DNBC, INMA	ALSPAC, DNBC	GENR, INMA	ALSPAC, CHOP^a^, EDEN, GENR, INMA

ADHD: Attention-deficit hyperactivity disorder, ASD: Autism Spectrum Disorder, Non-verbal IQ: Non-Verbal Intelligence, CI: Confidence interval, SDS: standard deviation score, ∗p value < 0·05, ∗∗p value < 0·01. Childhood measurements are between 4 and 10 years old, early adolescence measurements are between 11 and 16 years old and late adolescence measurements are between 17 and 20 years old. Values are regression coefficients (95% confidence interval) obtained from one stage meta-analysis and reflect the differences in ADHD symptoms (percentiles), ASD symptoms (percentiles), and Non-verbal IQ score (percentiles) for birth characteristics from pooled multiple imputed data. For birth characteristics from pooled multiple imputed data. The confounder model is adjusted for sex, age at outcome measurement, cohort effect, maternal age at birth, maternal educational level, parity, pre-pregnancy body mass index, and smoking during pregnancy.

a CHOP cohort is not included in the birth weight and gestational age categories due to missing data on preterm born and low birth weight children.

### Size at birth, childhood BMI and emotional, behavior and cognitive outcomes

Table 5 shows that as compared to children born AGA, those born SGA had higher scores for internalizing problems, externalizing problems and ADHD symptoms, and lower scores for Non-verbal IQ in childhood (all p-values < 0·01). Although not consistent, tendencies for opposite associations were observed for LGA born children. The corresponding basic models showed similar associations and are shown in Table S6.

**Table 5:** Size for gestational age at birth categories and emotional, behavior and cognitive outcomes in childhood.

Size for gestational age at birth, SD score	Internalizing problems		Externalizing problems		ADHD symptoms		Intelligence score	
	n	Difference in percentiles (95 CI)	n	Difference in percentiles (95 CI)	n	Difference in percentiles (95 CI)	n	Difference in percentiles (95 CI)
Small <5th percentile	3909	2·37 (1·37, 3·37)∗	3908	3·94 (3·01, 4·88)∗	3470	4·88 (3·87, 5·90)∗	1013	−7·02 (−8·84, −5·21)∗
Appropriate 5th^–^95th percentile	*75,169*	*Reference*	*75,144*	*Reference*	*71,594*	*Reference*	*14,252*	*Reference*
Large >95th percentile	9926	−0·28 (−0·94, 0·38)	9923	−1·18 (−1·80, −0·57)∗	9718	−1·09 (−1·73, −0·45)∗	1188	2·47 (0·77, 4·18)∗
Cohorts	ALSPAC, BIB, DNBC, EDEN, ELFE, GENR, INMA		ALSPAC, BIB, DNBC, EDEN, ELFE, GENR, INMA		ALSPAC, DNBC, EDEN, ELFE, GENR, INMA		ALSPAC, EDEN, GENR, INMA	

ADHD: Attention-deficit hyperactivity disorder, Non-verbal IQ: Non-Verbal Intelligence, CI: Confidence interval, SDS: standard deviation score, ∗p value < 0·01. Childhood measurements are between 4 and 10 years old. Values are regression coefficients (95% confidence interval) obtained from one stage meta-analysis and reflect the differences in childhood internalizing problems (percentiles), externalizing problems (percentiles), ADHD symptoms (percentiles), and Non-verbal IQ score (percentiles) for size for gestational age at birth in categories from pooled multiple imputed data. The confounder model is adjusted for sex, age at outcome measurement, cohort effect, maternal age at birth, maternal educational level, parity, pre-pregnancy body mass index, and smoking during pregnancy.

Table 6 shows the statistical interaction for the associations between size at birth and BMI in early childhood in relation to emotional, behavior and cognitive outcomes was not significant for any association. The results for the explorative analysis showed that when combining size at birth with BMI in early childhood, as compared to children born AGA with a normal early childhood BMI, those born LGA and a normal BMI at the age of 2 years had lower scores for internalizing problems (−1·40 (95% CI: −2·38, −0·42)). Children born SGA who remained in the lowest tertile of BMI at 2 years had the lowest Non-verbal IQ score (−8·14 percentiles (95% CI: −11·89, −4·39)). Children within the lowest tertile of BMI at 2 years had, independent of their size at birth, increased scores for ADHD (all p-values < 0·05), however these associations attenuated into non-significance after correction for multiple testing was applied. The corresponding basic models showed similar associations and are shown in Table S7.

**Table 6:** Growth patterns and emotional, behaviour and cognitive outcomes in childhood.

	Internalizing problems		Externalizing problems		ADHD symptoms		Intelligence score	
	n	Difference in percentiles (95 CI)	n	Difference in percentiles (95 CI)	n	Difference in percentiles (95 CI)	n	Difference in percentiles (95 CI)
p-value of interaction of size for gestational age and infant BMI	32,327	0.07	32,335	0.40	28,233	0.90	14,745	0.80
Small for gestational age								
BMI at 2 years								
1st tertile	564	−0·27 (−2·78, 2·24)	564	0·25 (−2·12, 2·62)	453	3·17 (0·47, 5·88)∗	230	−8·14 (−11·89, −4·39)∗∗
2nd tertile	4894	−0·64 (−1·62, 0·34)	4895	−0·29 (−1·22, 0·64)	4285	0·20 (−0·82, 1·22)	1814	−1·00 (−2·51, 0·51)
3rd tertile	164	−0·63 (−5·21, 3·94)	164	−0·42 (−4·77, 3·92)	144	0·40 (−4·33, 5·13)	86	0·45 (−5·61, 6·51)
Appropriate for gestational age								
BMI at 2 years								
1st tertile	339	2·86 (−0·35, 6·07)	339	2·42 (−0·62, 5·46)	266	3·82 (0·32, 7·32)∗	129	−2·45 (−7·41, 2·52)
2nd tertile	*20,457*	*Reference*	*20,461*	*Reference*	*18,007*	*Reference*	*10,244*	*Reference*
3rd tertile	*292*	−2·51 (−5·96, 0·94)	292	−4·01 (−7·27, −0·74)∗	256	0·41 (−3·15, 3·97)	125	2·23 (−2·80, 7·27)
Large for gestational age								
BMI at 2 years								
1st tertile	192	1·61 (−2·62, 5·84)	192	3·77 (−0·23, 7·78)	144	4·92 (0·20, 9·65)∗	72	−5·79 (−12·40, 0·82)
2nd tertile	*4902*	−1·40 (−2·38, −0·42)∗∗	4904	−0·55 (−1·48, 0·38)	4231	−1·14 (−2·17, −0·11)∗	1823	1·59 (0·07, 3·10)∗
3rd tertile	523	−1·82 (−4·42, 0·78)	524	−1·38 (−3·84, 1·08)	447	−2·35 (−5·07, 0·37)	222	3·36 (−0·47, 7·20)
Cohorts	ALSPAC, BIB, EDEN, ELFE, GENR, INMA		ALSPAC, BIB, EDEN, ELFE, GENR, INMA		ALSPAC, EDEN, ELFE, GENR, INMA		ALSPAC, GENR, INMA	

ADHD: Attention-deficit hyperactivity disorder, Non-verbal IQ: Non-Verbal Intelligence, CI: Confidence interval, SDS: standard deviation score, ∗p value < 0·05, ∗∗p value < 0·01. Childhood measurements are between 4 and 10 years old. Values are regression coefficients (95% confidence interval) obtained from one stage meta-analysis and reflect the differences in childhood internalizing problems (percentiles), externalizing problems (percentiles), ADHD symptoms (percentiles), and Non-verbal IQ score (percentiles) for the combinations of size for gestational age with early childhood BMI growth patterns from pooled multiple imputed data. The confounder model is adjusted for sex, age at outcome measurement, cohort effect, maternal age at birth, maternal educational level, parity, pre-pregnancy body mass index, and smoking during pregnancy.

### Sensitivity analyses

Tables S8–S11 show the results of a complete-case sensitivity analysis. The direction and magnitude of effect estimates were consistent with the main analysis. Figure S4 shows the results of sensitivity analyses from 2-stage individual participant data meta-analysis. The direction of the associations was consistent with the observations of the 1-stage analysis, but the results were less precise.

## Discussion

In this large scale European individual participant data meta-analysis, we observed that older gestational age at birth and higher birth weight were associated with lower scores for internalizing problems, externalizing problems and ADHD in childhood and early adolescence and with a higher non-verbal intelligence score in childhood. The associations tended to be weaker or even inversed during late adolescence. Furthermore, children born SGA with a BMI in the lowest tertile at 2 years of age had decreased Non-verbal IQ in childhood as compared to children born AGA with a normal BMI at 2 years of age. Our findings suggest fetal and early childhood growth have both effects on behavioral and cognitive outcomes in later life. Also, the adverse effects of suboptimal fetal growth, might be partly attenuated by compensatory early childhood growth.

Results from a previous large meta-analysis, showed that in 81% of studies, children born premature had higher internalizing and externalizing problems and a higher risk of ADHD.^5^ Another review reported that children born preterm have a 3–4 times higher risk of psychiatric disorders in childhood.^7^ A study conducted in Sweden among 546,000 sibling pairs examined the association of birth weight and psychiatric disorders and found higher rates of anxiety, externalizing problems and ADHD with decreasing birth weight and that by an increase of 1 kg in birth weight the risk of general and specific neurodevelopmental factors was reduced significantly.^4^ Using Norwegian health registries, a population-based study among 1·8 million term born children, reported that birth weight below 3500 g was associated with more behavioral disorders including ADHD.^3^

Results from our current study showed that both internalizing problems, externalizing problems and ADHD symptoms were increased in children or young adolescents who were born preterm or with low birth weight. These associations tended to be weaker or even inversed during late adolescence. Previous studies have shown that the increased behavioral problems of preterm born children in childhood do not seem to track into adulthood. Compared to their term born peers, preterm born adolescents were less likely to drink alcohol or use drugs and undertook in less risk full behavior.^48^^,^^49^ A potential explanation for the weaker or inversed associations in late adolescence could be maturation and puberty effects, that lead temporarily or permanent to other effects. Results from previous studies showed that adults born preterm might have slightly altered personalities in regard to their term born peers.^50^ Also from early adolescence onwards, agreeableness and conscientiousness tends to decrease.^51^ Another explanation for the difference in direction of the association might be that we only had data regarding externalizing problems and ADHD symptoms in ALSPAC and DNBC. The instrument used to measure internalizing, externalizing and ADHD symptoms in late adolescence was in DNBC cohort, as opposed to all other cohorts, self-reported. Previous studies have shown there can be discrepancies in parent-reported and self-reported assessment of emotional and behavioral problems.^52^^,^^53^ Though, these studies suggest that when children self-report, they are more likely to report more problems. However, we observed less problems in late adolescence.

Preterm birth and low birth weight have previously been associated with higher ASD.^3^^,^^4^^,^^7^^,^^54^ We observed that higher birth weight was associated with lower ASD symptoms. The lack of association for preterm birth might also be explained by lower numbers since ASD was only available in two cohorts, that have both a rather healthy population, without extremely premature children.

Preterm birth and low birth weight were both associated with lower Non-verbal IQ scores in childhood. In a recent meta-analysis over 30,000 participants, increasing birth weight and gestational age were both associated with higher IQ scores. However, the most profound effect was seen in preterm born children with a gestational age below 32 weeks.^55^ Our relatively healthy populations contain mostly late preterm children.

In this study, the observed effect estimates were quite strong, although not always statistically significant after correction for multiple testing. Our results are in line with results from previous studies, which show that children born preterm, low birth weight or with SGA more often have emotional, behavioral and cognitive problems. In clinical and population health settings, adverse birth outcomes should be taken into account as risk factor for emotional, behavioral and cognitive problems.

Birth characteristics are the starting point for early childhood growth. Both fetal and infant growth may have independent and synergistic effects in relation to later life outcomes. Results from a study among 11,000 children born at term and with normal birth weight from Belarus, suggest that increased birth weight and infancy weight growth was associated with higher IQ and with lower externalizing problems at age 5–6 years old.^16^ A review of studies that assessed the associations of birth weight and infant weight gain among non-SGA children showed no associations between growth trajectories and IQ in children.^56^ Another study from the United States among more than 1000 children showed that greater infancy BMI velocity was associated with lower verbal IQ.^57^ We observed that children born LGA with a normal BMI in early childhood had lower internalizing problems. Furthermore, we observed that children born SGA followed by an infant BMI in the smallest tertile was associated with lower IQ. In our results we found tendencies toward a compensatory effect of growth in early childhood for adverse effects of being SGA at birth. This trend was most visible for ADHD symptoms. However, the results attenuated after correction for multiple testing was applied. Fetal and infancy development are known critical and sensitive periods in brain development. The optimum infancy weight growth in relation to birth weight is unknown and should be subject of further research.

The mechanisms linking size in early life with emotional, behavioral and cognitive outcomes might be explained by better nutritional status and related head and brain development. In children with fetal and infant growth deceleration smaller brain volumes are observed. Children with fetal growth restriction have reduced total brain volume and less brain connectivity.^58^ Suboptimal brain development may be associated with developmental disorders, ADHD, and ASD.^59^, ^60^, ^61^, ^62^ Whereas children showing catch-up growth after fetal growth deceleration have been shown to have similar brain volumes as children of normal growth.^63^ Future studies are needed to identify whether determinants of early life growth, such as pregnancy complications, placenta insufficiency and maternal or infant nutrition explain the observed associations. Identification of birth characteristics and early life growth patterns could lead to windows of opportunity for novel strategies, such as monitoring behavior and cognitive development in children born preterm or of low birth weight and strategies to optimize neurodevelopmental outcomes in children at risk.

Strengths of this study are the large sample size available in the infrastructure of the EU Child Cohort Network enabled us to use harmonized data from different pregnancy and childhood cohort studies. This approach enables assessment of the effect estimates in different subgroups of birth characteristics and childhood BMI, and assessment of the consistency of the associations in different populations and at different time points. Another strength is we assessed full range scores for emotional, behavioral and cognitive outcomes. This study also has some limitations, though the sample sizes are large, there is a discrepancy in sample size between the different cohorts. Data availability differed greatly between cohorts. Ideally, we would have used sex and gestational age standardized z scores for birth weight, and length standardized z-scores for weight during infancy. However, these data were not available in the in the EU Child Cohort Network database. Therefore, we adjusted the analyses focused on the associations of birth weight categories and growth patterns with childhood outcomes for sex and gestational age at birth. The emotional, behavioral and cognitive outcomes were measured using different instruments and although harmonized, are not completely homogenous. We could not take account for different respondents of the emotional, behavior and cognitive outcomes questionnaires because this information was not completely available in the EU Child Cohort Network. Also, covariates were present in varying degrees, but missing covariates of interest were imputed. Residual confounding by various lifestyle, diet, sociodemographic or ethnic related factors cannot be excluded. Also, we did not have detailed information about other health conditions or comorbidities, which could affect physical, behavioral or cognitive development. Our study was conducted in relatively healthy children. Due to observational nature of the study, no conclusions can be drawn on the causality of the reported associations. Finally, the effect estimates may be different in clinical populations and care should be taken when extrapolating our findings to other populations.

### Conclusion

Both fetal and early childhood growth are associated with emotional, behavioural and cognitive outcomes in childhood and adolescence. Compensatory infant growth might partly attenuate the adverse effects of suboptimal foetal growth. Future studies are needed to identify the potential for optimizing mental health outcomes in new generations by improving early-life growth.

## Contributors

Romy Gonçalves is responsible for the study design, performed the statistical analyses and wrote the manuscript and had primary responsibility for the final content. Romy Gonçalves and Vincent V.W. Jaddoe have accessed and verified the underlying data and were responsible for the decision to submit the manuscript for publication. Sophia Blaauwendraad and Demetris Avraam contributed to the statistical analyses and critically reviewed the manuscript. Vincent V.W. Jaddoe and Romy Gaillard contributed to the design of the study, interpretation of the results and were responsible for critical review of the manuscript. All other co-authors contributed to the interpretation of the results and critically reviewed the manuscript. All authors have read and approved the final manuscript and agree to be accountable for all aspects of the work.

## Data sharing statement

The data used in this study is not freely available as access is managed by each individual cohort. Data access would need to be requested from each individual cohort.

## Declaration of interests

Prof. Dr. Berthold Koletzko has received reimbursement for active contribution to scientific and continuing medical education events by Danone, DGC, Nestlé and Reckitt. This study was not funded by Danone, DGC, Nestlé or Reckitt. No other conflicts of interest to declare.
