# Supplementary material for: Early-life growth and emotional, behavior and cognitive outcomes in childhood and adolescence in the EU child cohort network: individual participant data meta-analysis of over 109,000 individuals
Source: Lancet Reg Health Eur. 2025 Feb 26;52:101247. doi: 10.1016/j.lanepe.2025.101247 (PMC11910110; doi:10.1016/j.lanepe.2025.101247)
Supplement: Supplementary Materials [file mmc1.docx]

**Original article**

**Early-life Growth and Emotional, Behaviour and Cognitive Outcomes in Childhood and Adolescence in the EU Child Cohort Network: Individual Participant Data Meta-analysis of over 109,000 Individuals**

*Short title: Early-life growth and Development Outcomes*

Romy Gonçalves et al.

**Text S1.** Information on the profile and design of each cohort**.**

**Text S2.** Instruments used to measure emotional, behaviour and cognitive outcomes in childhood

**Text S3.** Method Multiple Imputation

**Text S4.** Cohort specific acknowledgements

**Text S5.** Cohort and persona specific details on funding

**Table S1.** Subject characteristics including missing values

**Table S2.** Emotional, behaviour and cognitive outcomes descriptives including missing values

**Table S3.** Descriptive statistics of the emotional, behaviour and cognitive outcomes in each cohort

**Table S4.** Birth characteristics and internalizing and externalizing problems score at school-age, basic model

**Table S5.** Birth characteristics and ADHD symptoms, ASD symptoms and Intelligence score at school-age, basic model

**Table S6.** Size for gestational age at birth categories and emotional, behaviour and cognitive outcomes in childhood, basic model

**Table S7.** Growth patterns and emotional, behaviour and cognitive outcomes in childhood, basic model

**Table S8.** Birth characteristics and internalizing and externalizing problems score at school-age, complete-case analysis

**Table S9.** Birth characteristics and ADHD symptoms, ASD symptoms and Intelligence score at school-age, complete-case analysis

**Table S10.** Size for gestational age at birth categories and emotional, behaviour and cognitive outcomes in childhood, complete-case analysis

**Table S11.** Growth patterns and emotional, behaviour and cognitive outcomes in childhood, complete-case analysis

**Figure S1.** Flowchart.

**Figure S2.** Cohort-specific flowcharts

**Figure S3.** Directed Acyclic Graph used to identify potential confounders.

**Figure S4.** Sensitivity analyses, two-stage individual participant data meta-analysis

**STROBE Checklist**

**Text S1. Information on the profile and design of each cohort.**

Avon Longitudinal Study of Parents and Children (ALSPAC)

The Avon Longitudinal Study of Parents and Children (ALSPAC) is a population-based cohort study, designed to explore numerous environmental and genetic health and development factors over multiple generations. Pregnant women resident in Avon, UK with expected dates of delivery between 1st April 1991 and 31st December 1992 were invited to take part in the study. 20,248 pregnancies have been identified as being eligible and the initial number of pregnancies enrolled was 14,541. Of the initial pregnancies, there was a total of 14,676 foetuses, resulting in 14,062 live births and 13,988 children who were alive at 1 year of age. When the oldest children were approximately 7 years of age, an attempt was made to bolster the initial sample with eligible cases who had failed to join the study originally. As a result, when considering variables collected from the age of seven onwards (and potentially abstracted from obstetric notes) there are data available for more than the 14,541 pregnancies mentioned above: The number of new pregnancies not in the initial sample (known as Phase I enrolment) that are currently represented in the released data and reflecting enrolment status at the age of 24 is 906, resulting in an additional 913 children being enrolled (456, 262 and 195 recruited during Phases II, III and IV respectively). The phases of enrolment are described in more detail in the cohort profile paper and its update (see footnote 5 below). The total sample size for analyses using any data collected after the age of seven is therefore 15,447 pregnancies, resulting in 15,658 foetuses. Of these 14,901 children were alive at 1 year of age. Extensive follow-up included 59 questionnaires from 4 weeks to 18 years of age, and 9 clinical assessment visits conducted between 7 and 17 years old. ALSPAC's data contains a wide range of phenotypic and environmental measures alongside biological samples. Ethical approval for the study was obtained from the ALSPAC Ethics and Law Committee and the Local Research Ethics Committees. Informed consent for the use of data collected via questionnaires and clinics was obtained from participants following the recommendations of the ALSPAC Ethics and Law Committee at the time.

Data availability for ALSPAC study: Please note that the study website contains details of all the data that is available through a fully searchable data dictionary and variable search tool" and reference the following webpage: <http://www.bristol.ac.uk/alspac/researchers/our-data/>.

Born in Bradford (BiB)

Established in 2007, the Born in Bradford cohort study, also known as BiB, aims to investigate the impact of genetic, nutritional, environmental, behavioural, and social factors on health and development in a deprived multi-ethnic population, both during childhood and later in adult life.

Bradford, home to one of the busiest maternity units in the UK, situated at Bradford Royal Infirmary, witnesses over 6000 new births annually. Women scheduled for delivery at this facility are offered an oral glucose tolerance test (OGTT) between 26 and 28 weeks of gestation, with approximately 80% opting for the test. Women are informed about the study during their booking appointment with a midwife and are invited to participate. Upon attendance at the OGTT clinic, women provide full consent for recruitment into BiB and complete an interviewer-administered questionnaire.

Between March 2007 and November 2010, more than 80% of the women attending the OGTT accepted the invitation, resulting in the recruitment of 12,453 women with 13,776 pregnancies to the cohort. OGTT results were available for 11,442 of them. Partners of the recruited women were also invited to participate in the study. Out of the total 13,776 pregnancies, 13,740 ended in live births.

European childhood obesity project trial (CHOP)

The primary objective of the EU Childhood Obesity Programme was to conduct a one-year multicenter intervention trial on newborn infants, focusing on the influence of varying levels of milk proteins in infant formulae on the later risk of childhood obesity. The trial, spanning five countries with diverse habitual total protein intakes, aimed to broaden the range of protein intakes and enhance statistical power for testing the 'early protein hypothesis,' which posits that early protein intake predicts infant growth and subsequent childhood obesity.

A total of 1678 children were recruited for the study. The source population was center based, with study centres in Belgium, Germany, Italy, Poland, and Spain. The enrolment period for the study extended from October 1, 2002, to July 31, 2004, with enrolment occurring between months 0 to 2. Eligibility criteria included infants up to 8 weeks old, both genders, and healthy volunteers. Inclusion criteria comprised gestational age greater than 37 weeks, appropriate for gestational age (above 10th percentile, Lubchenko), mothers aged at least 18 years, singleton pregnancies, residence in the study area, and maternal command of the language.

Exclusion criteria involved gestational diabetes, major malformations in the child that could interfere with nutrition or growth, hormonal or metabolic diseases in the mother or child, and drug addiction during pregnancy. The planned age of children at the end of the follow-up period was 11 years.

The Danish National Birth Cohort (DNBC)

Stands out for its strengths, including prospectively collected exposure data, biological samples, an exceptionally large sample size, and the ability to track participants through linkages to national health registries. Established with the aim of investigating the causal links between early-life exposures and later disease, the DNBC focuses on diseases such as cardiovascular morbidity, cancer, mental illnesses, asthma, and allergy, recognizing that many of these conditions may have causes acting early in life.

The choice of a large cohort of pregnant women with long-term follow-up for offspring was strategic, considering that some exposures of interest cannot be accurately reconstructed retroactively. The goal was to recruit 100,000 women early in pregnancy and continue follow-up for decades. The Nordic countries, with their population-based registers covering diseases, demography, and social conditions, are ideal for such research, thanks to the individual-level linkages facilitated by the unique ID numbers assigned to all citizens.

In terms of data and biological samples, the DNBC employed computer-assisted telephone interviews (CATI) with women twice during pregnancy and when their children were six and 18 months old. Participants also filled in a self-administered food frequency questionnaire in mid-pregnancy. The establishment of a biological bank included blood samples taken from the mother twice during pregnancy and from the umbilical cord shortly after birth. Data collection commenced in 1996, covering all regions in Denmark by 1999, and by August 2000, a significant milestone was achieved with the recruitment of 60,000 pregnant women. The study successfully reached its goal of 100,000 recruitments in October 2002.

Etude des Déterminants pré et post natals du développement et de la santé de l’Enfant (EDEN)

The French bicentric generalist cohort study known as the EDEN study was initiated in 2003 at two university maternity clinics, located in Nancy and Poitiers, France. The local clinical teams from the respective university hospitals, in collaboration with research teams from the National Institute of Health and Medical Research (Inserm), played a pivotal role in establishing EDEN.

The study offered participation to all women attending the prenatal clinic of the obstetrics and gynaecology department at the Poitiers and Nancy university hospitals before their 24th week of amenorrhea. Exclusion criteria comprised multiple pregnancies, pre-existing diabetes, French illiteracy, or plans to relocate out of the region within the next 3 years. Recruitment spanned from 2003 to 2006.

Mothers underwent clinical examinations between 24 and 28 weeks of amenorrhea and at the time of delivery, with the child clinically examined at birth. In addition to clinical visits, mothers and fathers completed questionnaires, either self-administered or administered by midwives, addressing information about their offspring, themselves, and their household. Biological samples were gathered from mothers during pregnancy and at birth, as well as from the child (or cord) at birth. Fathers also provided blood samples at the child’s birth.

Out of the 3758 women invited to participate, 2002 (53%) were successfully enrolled in the study, with 1034 women from Nancy and 968 from Poitiers. A total of 1899 mother-child pairs were followed up at birth.

Etude Longitudinale Francaise depuis l'Enfance (ELFE)

ELFE, has a primary focus on investigating the impact of various exposures on health outcomes, aims to shed light on the intricate interplay between familial history, socio-economic conditions, education, and a range of environmental stressors. These stressors encompass heavy metals, organic volatile compounds, pesticides, persistent organic pollutants (POP), phthalates, environmental tobacco smoke (ETS), molds, air pollutants, electromagnetic fields (EMF), ultraviolet (UV) radiation, ionizing radiations, and noise. The study seeks to evaluate outcomes related to neurodevelopment and school achievements, fertility, puberty and sexual development, asthma and allergies, obesity and growth, as well as hearing loss.

The cohort comprises 20,000 children which is approximately 3% of the nation-based source population. The enrolment period spans from April 1, 2011, to December 5, 2011, with enrolment occurring at birth.

Enrolment criteria specify that participants must have a recorded date of delivery, major mothers, term pregnancies of 33 weeks of amenorrhea or more, and single or twin pregnancies. Maternal mental incapacity serves as the sole exclusion criterion. The planned age for children at the end of the follow-up is 20 years, aiming to track and analyse their health outcomes over an extended period.

Generation R

The Generation R Study, based in Rotterdam, the second-largest city in the Netherlands, is a population-based prospective cohort study with the aim of identifying early environmental and genetic factors influencing normal and abnormal growth, development, and health from foetal life through young adulthood. Eligible for this study were mothers with delivery dates between April 2002 and January 2006, residing in the study area. Extensive assessments were conducted during various stages of pregnancy, including early (gestational age <18 weeks), mid (gestational age 18–25 weeks), and late pregnancy (gestational age >25 weeks).

A total of 9,778 mothers were enrolled in the study, with 91% (n = 8,880) enrolled during pregnancy. Only partners of mothers enrolled during pregnancy were invited, resulting in the enrolment of 71% (n = 6,347) of all partners. Among participating mothers, enrolment occurred in early pregnancy for 69% (n = 6,748), mid-pregnancy for 19% (n = 1,857), late pregnancy for 3% (n = 275), and at the birth of their child for 9% (n = 898). The 9,778 enrolled mothers gave birth to 9,749 live-born children.

INfancia y Medio Ambiente (INMA)

The INMA (Environment and Childhood) Project is a network of birth cohorts in Spain focused on studying the impact of environmental pollutants in air, water, and diet during pregnancy and early childhood on child growth and development. The project includes pregnant women residing in specific study areas [Ribera d’Ebre, Menorca, Granada, Valencia, Sabadell, Asturias, and Gipuzkoa] and their children. Inclusion criteria for mothers were residence in a study area, being over 16 years old, a singleton pregnancy, no assisted reproduction treatment, a desire to deliver in the reference hospital, and no communication problems.

Each cohort had a unique recruitment period. In Ribera d’Ebre and Granada, mothers were recruited during hospital admission for delivery between March 1997 and December 1999 and October 2000 and July 2002, respectively. Menorca invited eligible pregnant women presenting for prenatal care over 12 months starting in mid-1997. Valencia, Sabadell, Asturias, and Gipuzkoa recruited during the first prenatal visit (10–13 weeks of gestation) in the main public hospital or health center. Recruitment periods varied across areas, ranging from November 2003 to January 2008. The participation rate varied from 45% to 98%, with 96% in Ribera d’Ebre, 98% in Menorca, 54% in Valencia, 60% in Sabadell, 45% in Asturias, and 68% in Gipuzkoa.

Reasons for not participating in Sabadell and Gipuzkoa included 27.6% not wanting to participate, 30.3% citing lack of time, 9.3% expressing no interest, and 32.7% could not be located for the baseline interview after being identified as eligible by their attending physician. In total, 3,174 women were recruited during pregnancy, with 2,998 mother-child pairs followed up at birth.

**Text S2. Instruments used to measure emotional, behaviour and cognitive outcomes in childhood**

Internalizing and externalizing problems were assessed using either the Strengths and Difficulties

Questionnaire (SDQ; ALSPAC, ELFE, BIB, CHOP, DNBC, EDEN, INMA)(1) or the Child Behavior Checklist (CBCL; GenerationR, CHOP, INMA)(2). Both are parent-reported and higher scores indicate more emotional and behavioural problems. The SDQ is a 25‐item questionnaire, items are rated on a 3‐point Likert scale (“not true”, “somewhat true”, “certainly true”) containing five subscales: Emotional Problems, Conduct Problems, Hyperactivity/inattention, Peer problems and Pro‐social behaviour. The internalizing problems score was calculated by summing the scores on the Emotional Problems and Peer Problems scales. The externalizing problems score was calculated by summing scores on the Conduct Problems and Hyperactivity subscales. The CBCL measures emotional and behavioural problems on a continuous severity scale. Based on the behaviour of the child in the preceding 6 months, each of the total 113 items is rated on a 3-point scale: 0 (not true), 1 (somewhat true), 2 (very true or often true). Together the 113 items result in the total problems sum score, which can be subdivided into internalizing and externalizing problems. Internalizing problems consist of emotionally reactive and anxious/depressed symptoms, as well as somatic complaints and symptoms of being withdrawn. Externalizing problems consist of rule breaking and aggressive behaviour (3).

ADHD symptoms were measured using the SDQ, using the hyperactivity subscale (ALSPAC, ELFE, CHOP, DNBC, EDEN), the Conners’ Parent Rating Scale–Revised Short Form (CPRS-R:S; GenerationR, INMA)(4) or Diagnostic and Statistical Manual of Mental Disorders IV (DSM-IV; INMA)(5). For the CPRS-R:S Mothers reported on their children’s ADHD symptoms in the last month using a 27-item questionnaire. The questionnaire is measured on a 3‐point Likert scale (0 “not true at all”, 1 “just a little true”, 2 “pretty much true ” and 3 “very much true”). The CPRS-R:S includes the following 4 scales: Cognitive Problems/Inattention (6 items), Hyperactivity (6 items), Oppositional (6 items), and the ADHD index (ADHDi; 12 items)(4). The DSM-IV consists of questions on 9 inattention symptoms and 9 hyperactivity-impulsivity symptoms on a 4-point Likert scale, which was filled out by teachers to assess ADHD symptoms. The sum score of these 18 questions resulted in the total ADHD symptoms score with higher scores indicating more ADHD symptoms(5).

ASD symptoms were measured by the Social Responsiveness Scale (SRS; GENR) or the Childhood Autism Spectrum Test (CAST; INMA) (6, 7). For both tests, higher scores indicate more ASD symptoms. An adapted version of the SRS consisting of 18 items was completed by a parent. This abbreviated version shows high correlations with the full scale (8, 9). The SRS is a quantitative measure of autistic traits for children aged between 4 and 18 years (8, 10). Scoring is on a four-point Likert scale. The shortened 18-item SRS contained items from three subscales: social cognition, social communication and autistic mannerisms. For the CAST a psychologist questioned a 37-item test to the child’s parents. Scores ranging from 0 to 31 points. With the CAST also subtle manifestations of autism spectrum conditions (social impairments, communication impairments, and repetitive or stereotyped behaviours) are identified (6, 7).

Non-verbal IQ was assessed using the Snijders-Oomen Niet-verbale intelligentie Test- Revisie (SON-R 2.5–7; GENR)(11), The [Wechsler Preschool and Primary Scale of Intelligence](https://www.sciencedirect.com/topics/medicine-and-dentistry/wechsler-preschool-and-primary-scale-of-intelligence) Third Edition (WPPSI-III; ALSPAC, EDEN)(12), the McCarthy Scales of Children's Abilities (MSCA; INMA) (13) or the Cattell Culture Fair Intelligence Test (CFIT; CHOP)(14). The SON-R 2½–7, is a nonverbal intelligence test suited for children of 2.5–7 years of age. A shortened version consisting of two subtests; Mosaics, for visuospatial abilities, and Categories, for abstract reasoning. Non-verbal IQ was constructed by converting raw scores to exact age (11). The WPPSI-III was administered by trained psychologists and core subtests (Information, Vocabulary, Word Reasoning, Block Design, Matrix Reasoning, Picture Concepts, and Coding) were reviewed (12). For the MSCA a validated Spanish adaptation was used. The MSCA is comprised of 18 subtests in 5 sub-areas: verbal, quantitative, memory, perceptual performance, and motor abilities, including fine and gross [motor skills](https://www.sciencedirect.com/topics/earth-and-planetary-sciences/sensorimotor-performance) (13). From these subsets a global Non-verbal IQ can be established(15).

As previously described; ‘The LifeCycle Project has developed a protocol to generate harmonized variables across a selection of important cognitive and mental health domains. This harmonization approach creates standardized scores and percentiles for important domains, such as internalizing and externalizing symptoms, ADHD and ASD symptoms and diagnosis, and language and motor functions. Percentiles and standardized scores were used, as they allow the pooling of mental health outcome data collected using different scales or instruments. One of the biggest harmonization challenges this project faced was obtaining a thorough inventory of the available mental health data in individual cohorts, which was overcome by mapping the available data by instrument, measure, age group, and domain (16).’ In this study we used the harmonized variables which represent percentiles of each emotional, behaviour and cognitive outcome.

**Text S3. Method Multiple Imputation**

The function miceDS (17) was used, which is a wrapper function of the mice from R package (18). This function creates multiple imputations for multivariate missing data. The method is based on Fully Conditional Specification, where each incomplete variable is imputed by a separate model, this generates multivariate imputations by Chained Equations method (MICE) (19) We performed 5 imputations, using 5 iterations. Five imputed data frames are saved on the dataSHIELD servers.

**REFERENCES**

1. Goodman R. The Strengths and Difficulties Questionnaire: a research note. J Child Psychol Psychiatry. 1997;38(5):581-6.

2. Achenbach TM, Ruffle TM. The Child Behavior Checklist and related forms for assessing behavioral/emotional problems and competencies. Pediatr Rev. 2000;21(8):265-71.

3. Blok E, Schuurmans IK, Tijburg AJ, Hillegers M, Koopman-Verhoeff ME, Muetzel RL, et al. Cognitive performance in children and adolescents with psychopathology traits: A cross-sectional multicohort study in the general population. Dev Psychopathol. 2023;35(2):926-40.

4. Conners CK, Sitarenios G, Parker JD, Epstein JN. The revised Conners' Parent Rating Scale (CPRS-R): factor structure, reliability, and criterion validity. J Abnorm Child Psychol. 1998;26(4):257-68.

5. Battle DE. Diagnostic and Statistical Manual of Mental Disorders (DSM). Codas. 2013;25(2):191-2.

6. Baron-Cohen S, Scott FJ, Allison C, Williams J, Bolton P, Matthews FE, Brayne C. Prevalence of autism-spectrum conditions: UK school-based population study. Br J Psychiatry. 2009;194(6):500-9.

7. Lopez-Vicente M, Sunyer J, Lertxundi N, Gonzalez L, Rodriguez-Dehli C, Espada Saenz-Torre M, et al. Maternal circulating Vitamin D(3) levels during pregnancy and behaviour across childhood. Sci Rep. 2019;9(1):14792.

8. Lyall K, Hosseini M, Ladd-Acosta C, Ning X, Catellier D, Constantino JN, et al. Distributional Properties and Criterion Validity of a Shortened Version of the Social Responsiveness Scale: Results from the ECHO Program and Implications for Social Communication Research. J Autism Dev Disord. 2021;51(7):2241-53.

9. Sturm A, Kuhfeld M, Kasari C, McCracken JT. Development and validation of an item response theory-based Social Responsiveness Scale short form. J Child Psychol Psychiatry. 2017;58(9):1053-61.

10. Cheon KA, Park JI, Koh YJ, Song J, Hong HJ, Kim YK, et al. The social responsiveness scale in relation to DSM IV and DSM5 ASD in Korean children. Autism Res. 2016;9(9):970-80.

11. P.J. Tellegen JAL. Snijders-Oomen Niet-verbale Intelligentietest SON-R 2½ - 7. Lisse: Swets & Zeitlinger; 1998. 168 p.

12. Wechsler D. Wechsler Preschool and Primary Scale of Intelligence. Third ed. San Antonio, TX: Psychological Corporation; 2002.

13. McCarthy. Escalas McCarthy de Aptitudes y Psicomotricidad para Niños. Madrid: TEA ediciones; 2009.

14. Cattell RB. Culture Free Intelligence Test, Scale 1, Handbook. Journal of Educational Psychologyg 1949.

15. Julvez J, Forns M, Ribas-Fito N, Torrent M, Sunyer J. Attention behavior and hyperactivity and concurrent neurocognitive and social competence functioning in 4-year-olds from two population-based birth cohorts. Eur Psychiatry. 2011;26(6):381-9.

16. Nader JL, Lopez-Vicente M, Julvez J, Guxens M, Cadman T, Elhakeem A, et al. Measures of Early-life Behavior and Later Psychopathology in the LifeCycle Project - EU Child Cohort Network: A Cohort Description. J Epidemiol. 2023;33(6):321-31.

17. Avraam D. ds.mice: Multivariate Imputation by Chained Equations: Github; 2024 [Available from: <https://rdrr.io/github/datashield/dsBaseClient/man/ds.mice.html>.

18. Stef van Buuren [aut c, Karin Groothuis-Oudshoorn [aut], Gerko Vink [ctb], Rianne Schouten [ctb], Alexander Robitzsch [ctb], Patrick Rockenschaub [ctb], Lisa Doove [ctb], Shahab Jolani [ctb], Margarita Moreno-Betancur [ctb], Ian White [ctb], Philipp Gaffert [ctb], F. mice: Multivariate Imputation by Chained Equations 2024 [Available from: <https://cran.r-project.org/web/packages/mice/index.html>.

19. Stef van Buuren KG-O. mice: mice: Multivariate Imputation by Chained Equations [Available from: <https://www.rdocumentation.org/packages/mice/versions/3.17.0/topics/mice>.

16. Avraam D. ds.mice: Multivariate Imputation by Chained Equations: Github; 2024 [Available from: <https://rdrr.io/github/datashield/dsBaseClient/man/ds.mice.html>.

17. Stef van Buuren [aut c, Karin Groothuis-Oudshoorn [aut], Gerko Vink [ctb], Rianne Schouten [ctb], Alexander Robitzsch [ctb], Patrick Rockenschaub [ctb], Lisa Doove [ctb], Shahab Jolani [ctb], Margarita Moreno-Betancur [ctb], Ian White [ctb], Philipp Gaffert [ctb], F. mice: Multivariate Imputation by Chained Equations 2024 [Available from: <https://cran.r-project.org/web/packages/mice/index.html>.

18. Stef van Buuren KG-O. mice: mice: Multivariate Imputation by Chained Equations [Available from: <https://www.rdocumentation.org/packages/mice/versions/3.17.0/topics/mice>.

**Text S4. Ethics approval**

**ALSPAC**

Ethical approval for the study was obtained from the ALSPAC Ethics and Law Committee and the Local Research Ethics Committees on the 28^th^ of November 1989. Informed consent for the use of data collected via questionnaires and clinics was obtained from participants following the recommendations of the ALSPAC Ethics and Law Committee at the time.

**BiB**

The National Health Service Health Research Authority Yorkshire and the Humber (Bradford Leeds) Research Ethics committee approved the study (Baseline recruitment: 07/H1302/112, 1^st^ April 2008; Growing up follow-up: 16/YH/0320, 22^nd^ September 2016). Written or electronic informed consent was obtained from parents. Participants are given a copy of the participant information sheet and an opportunity to discuss any questions with a member of the research team.

**CHOP**

The local ethics committees of each study center approved all study procedures: Belgium: Comitè d’Ethique de L’Hopital Universitaire des Enfants Reine Fabiola; no. CEH 14/02 (June 2002); Germany: Bayerische Landesärztekammer Ethik-Kommission; no. 02070 (October 2002); Italy: Azienda Ospedaliera San Paolo Comitato Etico; no. 14/2002 (July 2002); Poland: Instytut Pomnik–Centrum Zdrowia Dziecka Komitet Etyczny; no 243/KE/2001 (May 2001); Spain: Comité ético de investigación clínica del Hospital Universitario de Tarragona Joan XXIII (June 2002). Written informed consent was obtained from all parents before enrolment and at 6 and 11 years. At 8 and 11 years also children consent was obtained.

**DNBC**

The DNBC cohort study is approved by the Danish Data Protection Agency and the Committee on Health Research Ethics. The regional scientific ethical Committee for the municipalities of Copenhagen and Frederiksberg has approved the study on the 8th of February 1995, project (KF) 01-471/94. Data handling in the DNBC has been approved by Statens Serum Institut (SSI) under ref. no 18/04608 and is covered by the general approval (Fællesanmeldelse) given to SSI. The cohort was approved under ref. no 2008-54-0431. Written informed consent was obtained from all parents and children before enrolment.

**EDEN**

The EDEN study cohort received approval on the 12^th^ of December 2002 from the ethics committee (CCPPRB, N°02-70) of Kremlin Bicêtre and from the Commission Nationale Informatique et Liberté (CNIL, n°902267), the French data privacy institution. Written informed consent was obtained twice from parents, once at enrolment and once after the child’s birth.

**ELFE**

For the ELFE cohort Ethical approvals for data collection in maternity units and for each data collection wave during follow-up were obtained from the national advisory committee on information processing in health research (CCTIRS: Comité Consultatif sur le Traitement de l’Information en matière de Recherche dans le domaine de la Santé), the national data protection authority (CNIL: Comission Nationale Informatique et Liberté) and, in case of invasive data collection such as biological sampling, the committee for protection of persons engaged in research (CPP: Comité de Protection des Personnes). The Elfe study was also approved by the national committee for statistical information (CNIS: Conseil National de l’Information Statistique). For inclusion in the cohort, references are: CCTIRS: n°10.623 date 2010/10/26; , CNIL: Autorisation n°910504 – délibération 2011-081 date 2011/03/17 ; CPP: CPP-IDF IX-11-024  date 2011/05/13; informed consent was signed by the parents or the mother alone, with the father being informed of his right to deny consent for participation. Information and consent documents were provided in French, Arabic, Turkish and English, the most common languages of women giving birth in France.

**Generation R**

For the Generation R study cohort The Medical Ethics Committee of Erasmus Medical Center approved the study (MEC 198.782/2001/31, MEC 217.595/2002/202, MEC-2007-413; NL21545.078.08, MEC-2012-165; NL40020.078.12 en MEC 2015-749; NL55105.078.15). Written informed consent was provided by all parents and children.

**INMA**

For INMA cohort the Clinical Ethical Committee of the University Hospital of La Fe in Valencia, on October 27^th^ 2004, The Regional Clinical Research Ethical Committee, on April 3^rd^ 2003, The Regional Clinical Research Ethical Committee, on July 19^th^ 2005. All participants, regardless of the study area, are informed of the aims of the study. They are given an information sheet and a paper consent form by post. A telephone call is made to ask if they have received the information sheet, and an appointment is made on the same call. They bring the signed consent form to the appointment.

**Text S5. Cohort specific acknowledgements**

**ALSPAC**

We are extremely grateful to all the families who took part in this study, the midwives for their help in recruiting them, and the whole ALSPAC team, which includes interviewers, computer and laboratory technicians, clerical workers, research scientists, volunteers, managers, receptionists and nurses.

**BiB**

The authors acknowledge that Born in Bradford is only possible because of the enthusiasm and commitment of the children and parents in Born in Bradford. We are grateful to all participants, health professionals and researchers who have made Born in Bradford happen.

**CHOP**

The authors would particularly like to thank all the cohort participants for their generous collaboration. Furthermore, thanks to all persons who designed and conducted the study, entered the data, and participated in the data analysis and who are represented by the European Childhood Obesity Trial Study Group participants: B Koletzko, V Grote, K Gürlich, N Aumüller, V Luque, M Zaragoza-Jordana, N Ferré, J Escribano, R Closa-Monasterolo, A Xhonneux, JP Langhendries, E Verduci, E Riva, D Gruszfeld.

**DNBC**

The authors would like to thank the participants, the researchers who established and have been involved in the maintenance of the DNBC, the scientific managerial team, and DNBC secretariat for being, establishing, developing and consolidating the Danish National Birth Cohort.

**EDEN**

The authors thank the cohort participants and the EDEN mother-child study group. We are grateful to all participants, health professionals and researchers who have been making the EDEN cohort happen.

**ELFE**

The authors are grateful to 1) the former members of the Elfe unit without whom the project would never have started 2) the expertise and assistance of members of the unit for support functions, 3) all the researchers who contribute to the projects as members of the Elfe thematic groups and especially their coordinators; 4) all the field research assistants and interviewers; 5) and above all, all the Elfe families who have placed their confidence in us and given up their time to the study.

**Generation R**

The authors gratefully acknowledge the contribution of participants, research collaborators, general practitioners, hospitals, midwives, and pharmacies in Rotterdam.

**INMA**

The authors would particularly like to thank all the participants for their generous collaboration. We are grateful to all interviewers, health professionals and researchers who have assisted in contacting the families and administering the questionnaires.

**Text S6. Cohort and persona specific funding**

**ALSPAC**

The UK Medical Research Council and Wellcome (Grant ref: 217065/Z/19/Z) and the University of Bristol provide core support for ALSPAC. This publication is the work of the authors and A. Gonçalves Soares and A. Elhakeem will serve as guarantors for the contents of this paper. This project received funding from the European Union’s Horizon 2020 research and innovation programme (874739 LongITools). A comprehensive list of grants funding is available on the ALSPAC website. This research was specifically funded by Wellcome Trust and MRC (core) Grant 076467/Z/05/Z.

Ana Gonçalves Soares: Works in a Unit that is funded by the UK Medical Research Council (MC_UU_00011/1&6) and the University of Bristol.

Ahmed Elhakeem: Works in a Unit that is funded by the UK Medical Research Council (MC_UU_00011/1&6), the University of Bristol and H2020 101021566 ART-HEALTH.

**BiB**

Born in Bradford receives funding from by a joint grant from the UK Medical Research Council (MRC) and UK Economic and Social Science Research Council (ESRC) [MR/N024391/1]; the British Heart Foundation [CS/16/4/32482]; a Wellcome Infrastructure Grant [WT101597MA]; The National Institute for Health Research under its Applied Research Collaboration for Yorkshire and Humber [NIHR200166].The views expressed are those of the author(s), and not necessarily those of the NHS, the NIHR or the Department of Health and Social Care.

**CHOP**

The CHOP study has been carried out with partial financial support from the Commission of the European Community, specific RTD Programme "Quality of Life and Management of Living Resources", within the Fifth Framework Program (research grants no. QLRT-2001-00389, QLAM 2001-00582 and QLK1-CT-200230582), the Sixth Framework Program (contract no. 007036), and Seventh Framework Programme (EarlyNutrition; grant agreement no. 289346), the EU H2020 project LIFECYCLE under grant no. 733206 and the European Research Council Advanced Grant META-GROWTH (ERC-2012-AdG – no.322605) and with financial support from Polish Ministry of Science and Higher Education (2571/7.PR/2012/2). DYNAH Funding to LMU University and LMU University Hospital EALTH-633595, CoreMD 965246, EU Joint Programming Initiative JPI HDL EndObesity and BiomarKids, German Ministry of Education and Research (01EA1904, 01EA2101 and 01EA2203A), German Center for Child and Adolescent Health funded by the German Ministry of Education and Research, and Else Kröner Fresenius Foundation. Co-funded by the German Ministry of Education and Research as part of the German Center for Child and Adolescent Health (DZKJ), 01GL2406A. This article does not necessarily reflect the views of the Commission and in no way anticipates the future policy in this area. No funding bodies had any role in the study design, data collection and analysis, decision to publish, or preparation of the manuscript.

Berthold Koletzko: Is the Else Kröner Seniorprofessor of Paediatrics at LMU - University of Munich, financially supported by Else Kröner-Fresenius-Foundation, LMU Medical Faculty and LMU University Hospitals.

**DNBC**

The Danish National Birth Cohort was established with a significant grant from the Danish National Research Foundation. Additional support was obtained from the Danish Regional Committees, the Pharmacy Foundation, the Egmont Foundation, the March of Dimes Birth Defects Foundation, the Health Foundation and other minor grants. The DNBC Biobank has been supported by the Novo Nordisk Foundation and the Lundbeck Foundation. Follow-up of mothers and children have been supported by the Danish Medical Research Council (SSVF 0646, 271-08-0839/06-066023, O602-01042B, 0602-02738B), the Lundbeck Foundation (195/04, R100-A9193), The Innovation Fund Denmark 0603-00294B (09-067124), the Nordea Foundation (02-2013-2014), Aarhus Ideas (AU R9-A959-13-S804), University of Copenhagen Strategic Grant (IFSV 2012), and the Danish Council for Independent Research (DFF – 4183-00594 and DFF - 4183-00152). AP is funded by a Lundbeck Foundation fellowship (R264-2017-3099).

Demetris Avraam: is funded by the European Union's Horizon Europe Research and Innovation Programme under the Marie Skłodowska-Curie Postdoctoral Fellowship Grant Agreement No. 101106261 (ESICRI).

**EDEN**

The EDEN study was supported by Foundation for Medical Research (FRM), National Agency for Research (ANR), National Institute for Research in Public health (IRESP: TGIR cohorte santé 2008 programme), French Ministry of Health (DGS), French Ministry of Research, INSERM Bone and Joint Diseases National Research (PRO-A) and Human Nutrition National Research Programs, Paris-Sud University, Nestlé, French National Institute for Population Health Surveillance (InVS), French National Institute for Health Education (INPES), the European Union FP7 programmes (FP7/2007–2013, HELIX, ESCAPE, ENRIECO, Medall projects), Diabetes National Research Program (through a collaboration with the French Association of Diabetic Patients (AFD)), French Agency for Environmental Health Safety (now ANSES), Mutuelle Générale de l'Education Nationale complementary health insurance (MGEN), French national agency for food security, French-speaking association for the study of diabetes and metabolism (ALFEDIAM). Data availability statement: The EDEN cohort data that support the results of this study can be made available upon request to the EDEN Steering Committee. Data sharing will be subject to the European data protection regulations (GDPR). Readers may contact etude.eden@inserm.fr to request the data.

**ELFE**

The ELFE survey is a joint project between the French Institute for Demographic Studies (INED) and the National Institute of Health and Medical Research (INSERM), in partnership with the French blood transfusion service (Etablissement français du sang, EFS), Santé publique France, the National Institute for Statistics and Economic Studies (INSEE), the Direction générale de la santé (DGS, part of the Ministry of Health and Social Affairs), the Direction générale de la prévention des risques (DGPR, Ministry for the Environment), the Direction de la recherche, des études, de l’évaluation et des statistiques (DREES, Ministry of Health and Social Affairs), the Département des études, de la prospective et des statistiques (DEPS, Ministry of Culture), and the Caisse nationale des allocations familiales (CNAF), with the support of the Ministry of Higher Education and Research and the Institut national de la jeunesse et de l’éducation populaire (INJEP). Via the RECONAI platform, it receives a government grant managed by the National Research Agency under the "Investissements d'avenir" programme (ANR-11-EQPX-0038 and ANR 19 COHO-0001).

**Generation R**

The Generation R Study is financially supported by the Erasmus Medical Center, Rotterdam, the Erasmus University Rotterdam and the Netherlands Organization for Health Research and Development and the Ministry of Health, Welfare and Sport. This project received funding from the European Union's Horizon 2020 research and innovation programme (LIFECYCLE, grant agreement No 733206, 2016; EUCAN-Connect grant agreement No 824989; ATHLETE, grant agreement No 874583). This work was supported by the European Research Council [Consolidator Grant, ERC-2014-CoG-648916, received by Prof. Vincent V.W. Jaddoe and Starting Grant, ERC-2024-STG-101161004, received by Dr. Romy Gaillard). The Netherlands Organization for Health Research and Development [NWO, ZonMW, grant number 543003109 and 05430052110007, received by Dr Romy Gaillard]. s

H. El Marroun: HM was supported by the Stichting Volksbond Rotterdam, The Netherlands Organization for Health Research and Development (Aspasia No.015.016.056) and the European Union's Horizon 2020 research and innovation programme (HAPPYMUMS Grant Agreement no. 101057390).

**INMA**

Funded by: Ministry of Universities (grant CAS21/00008 and grant for the requalification of the Spanish University, financed by the European Union, NextGeneration EU). Other INMA funding for Valencia, Gipuzkoa and Sabadell subcohorts: Instituto de Salud Carlos III [FIS-FEDER: 13/1944, 16/1288, 17/00663 and 19/1338; FIS-FSE: 17/00260; Miguel Servet-FSE: MSII20/0006, FIS-PI06/0867, FIS-PI09/00090, CPII18/00018], CIBERESP, Department of Health of the Basque Government (2005111093, 2009111069, 2013111089, 2015111065), Generalitat Valenciana [BEST/2020/059, AICO/2020/285 and CIAICO/2021/132] and the Provincial Government of Gipuzkoa (DFG06/002, DFG08/001 and DFG15/221 and DFG 89/17). ISGlobal acknowledges support from the grant CEX2023-0001290-S funded by MCIN/AEI/ 10.13039/501100011033, and support from the Generalitat de Catalunya through the CERCA Program. INMA Study also acknowledge support from the Spanish Ministry of Science and Innovation and the State Research Agency through the “Centro de Excelencia Severo Ochoa 2019-2023” Program (CEX2018-000806-S), and support from the Generalitat de Catalunya through the CERCA Program.

Manuel Lozano: Ministry of Universities (grant CAS21/00008 and grant for the requalification of the Spanish University, financed by the European Union, NextGeneration EU).

| Table S1. Subject characteristics |
| --- |

|  | **All cohorts**  **(n=109481)** | **ALSPAC, United Kingdom**  **(n=10511)** | **BIB, United Kingdom**  **(n=2346)** | **CHOP,**  **Germany**  **(n=723)** | **DNBC,**  **Denmark**  **(n=74893)** | **EDEN,**  **France**  **(n=1259)** | **ELFE,**  **France**  **(n=10889)** | **Generation R**  **The Netherlands**  **(n=7331)** | **INMA**  **Spain**  **(n=1529)** |
| --- | --- | --- | --- | --- | --- | --- | --- | --- | --- |
| **Maternal** |  |  |  |  |  |  |  |  |  |
| Birth years | 1991-2011 | 1991-1992 | 2008-2011 | 2002-2004 | 1998-2002 | 2003-2006 | 2004-2011 | 2002-2006 | 2004-2008 |
| Age in years, median (IQR) | 29.9 (26.8, 33.0) | 26.0 (23.0, 30.0) | 28.0 (24.0, 32.0) | 31.0 (28.0, 34.0) | 30.0 (27.0, 33.0) | 30.0 (27.0, 33.0) | 31.0 (28.0, 34.0) | 32.0 (28.0, 35.0) | 32.0 (30.0, 35.0) |
| *Missing n (%)* | *1444 (1.3)* | *1410 (13.4)* | *0 (0.0)* | *1 (0.1)* | *2 (0.0)* | *0 (0.0)* | *26 (0.2)* | *0 (0.0)* | *5 (0.3)* |
| Pre-pregnancy BMI in kg/m^2^, median (IQR) | 22.6 (20.6, 25.3) | 22.2 (20.5, 24.4) | 24.9 (21.8, 28.8) | 22.3 (20.4, 25.5) | 22.6 (20.7, 25.4) | 22.2 (20.1, 25.4) | 22.1 (20.2, 25.1) | 22.7 (20.8, 28.7) | 22.6 (20.8, 25.1) |
| *Missing n (%)* | *9213 (8.4)* | *1133 (10.8)* | *1324 (56.4)* | *24 (3.3)* | *4926 (6.6)* | *22 (1.7)* | *118 (1.1)* | *1654 (22.6)* | *12 (0.8)* |
| Education, *n* (%) |  |  |  |  |  |  |  |  |  |
| High | 39010 (42.1) | 1430 (14.3) | 629 (28.6) | 223 (30.9) | 26926 (49.7) | 753 (60.0) | 7381 (67.7) | 3216 (48.0) | 552 (36.7) |
| Medium | 34945 (37.8) | 6927 (69.0) | 304 (13.8) | 365 (50.6) | 20023 (36.9) | 446 (35.6) | 3022 (27.7) | 2032 (30.4) | 626 (41.5) |
| Low | 18640 (20.1) | 1670 (16.7) | 1263 (57.5) | 133 (18.4) | 7255 (13.4) | 55 (4.4) | 486 (4.5) | 1448 (21.6) | 330 (21.8) |
| *Missing n (%)* | *22086 (20.2)* | *484 (4.6)* | *150 (6.4)* | *2 (0.3)* | *20689 (27.6)* | *5 (0.4)* | *0 (0.0)* | *635 (8.7)* | *21 (1.4)* |
| Ethnicity, *n* (%) |  |  |  |  |  |  |  |  |  |
| Western | 16248 (69.0) | NA | 703 (30.0) | NA | NA | 1096 (99.0) | 8718 (83.3) | 4290 (60.2) | 1441 (94.6) |
| Non-Western | 5834 (24.7) | NA | 1579 (67.4) | NA | NA | 6 (0.5) | 1018 (9.8) | 2150 (30.1) | 81 (5.4) |
| Mixed | 1481 (6.3) | NA | 60 (2.6) | NA | NA | 5 (0.5) | 722 (6.9) | 694 (9.7) | NA |
| *Missing n (%)* | *86718 (79.2)* | *NA* | *4 (0.2)* | *NA* | *NA* | *152 (12.1)* | *431 (4.0)* | *197 (2.7)* | *7 (0.5)* |
| Parity |  |  |  |  |  |  |  |  |  |
| 0 | 51461 (48.2) | 4553 (44.9) | 837 (36.3) | 361 (50.0) | 36030 (48.1) | 583 (46.4) | 4987 (46.3) | 3992 (56.4) | 818 (56.0) |
| 1 or more | 55310 (51.8) | 5538 (54.1) | 1467 (63.7) | 361 (50.0) | 38861 (51.9) | 674 (53.6) | 5782 (53.7) | 3102 (43.6) | 643 (44.0) |
| *Missing n (%)* | *890 (0.8)* | *420 (3.9)* | *42 (1.8)* | *1 (0.1)* | *0 (0.0)* | *2 (0.2)* | *120 (1.1)* | *237 (3.2)* | *68 (4.4)* |
| Smoking during pregnancy, *n* (%) | 25099 (23.6) | 2404 (25.7) | 228 (9.7) | 202 (28.0) | 18647 (25.3) | 274 (21.8) | 1803 (16.6) | 1593 (25.0) | 448 (29.7) |
| *Missing n (%)* | *4418 (4.0)* | *1144 (10.9)* | *4 (0.2)* | *2 (0.3)* | *1214 (1.6)* | *5 (0.4)* | *79 (0.7)* | *950 (13.0)* | *20 (1.3)* |
| **Birth** |  |  |  |  |  |  |  |  |  |
| Gestational age in weeks, median (IQR) | 40.2 (39.1, 41.0) | 40.4 (39.4, 41.4) | 39.9 (38.7, 40.7) | 40.6 (39.6, 41.6) | 40.1 (39.0, 41.0) | 40.4 (39.4, 40.4) | 39.9 (39.0, 40.7) | 40.7 (39.6, 41.6) | 40.0 (39.0, 40.9) |
| Preterm birth <37 wks, *n (%)* | 5959 (5.3) | 562 (5.3) | 154 (6.6) | NA | 4320 (5.8) | 71 (5.6) | 475 (4.4) | 331 (4.5) | 46 (3.0) |
| Term birth 37-42 wks, *n (%)* | 96534 (86.4) | 9109 (86.8) | 2178 (92.8) | 654 (90.6) | 66290 (88.5) | 1167 (92.7) | 10342 (95.5) | 5982 (82.2) | 1432 (94.0) |
| Postterm birth >42 wks, *n (%)* | 9259 (8.3) | 827 (7.9) | 14 (0.6) | 68 (9.4) | 4281 (5.7) | 21 (1.7) | 14 (0.1) | 966 (13.3) | 46 (3.0) |
| *Missing n (%)* | *131 (0.1)* | *13 (0.1)* | *0 (0.0)* | *1 (0.1)* | *2 (0.0)* | *0 (0.0)* | *58 (0.5)* | *52 (0.7)* | *5 (0.3)* |
| Birth weight in grams, (median, IQR) | 3515 (3167, 3855) | 3440 (3110, 3760) | 3200 (2840, 3560) | 3300 (3050, 3550) | 3580 (3220, 3930) | 3310 (3000, 3620) | 3340 (3040, 3640) | 3430 (3070, 3774) | 3290 (3000, 3550) |
| Low birth weight, <2500 gr, *n (%)* | 4807 (4.4) | 504 (4.8) | 233 (9.9) | NA | 3101 (4.1) | 61 (4.8) | 424 (3.9) | 412 (5.6) | 72 (4.7) |
| Normal birth weight, 2500-4500 gr, *n (%)* | 100264 (91.6) | 9823 (93.5) | 2092 (89.2) | 723 (100.0) | 68884 (92.0) | 1190 (94.5) | 10355 (96.0) | 6747 (92.0) | 1450 (94.8) |
| High birth weight, >4500 gr, *n (%)* | 4410 (4.0) | 184 (1.7) | 21 (0.9) | NA | 2908 (3.9) | 8 (0.7) | 110 (0.1) | 172 (2.4) | 7 (0.5) |
| *Missing n (%)* | *0 (0.0)* | *0 (0.0)* | *0 (0.0)* | *0 (0.0)* | *0 (0.0)* | *0 (0.0)* | *0 (0.0)* | *0 (0.0)* | *0 (0.0)* |
| Sex and gestational age adjusted birth weight in SDS, *n (%)* |  |  |  |  |  |  |  |  |  |
| Small (<5th percentile) | 5671 (4.9) | 603 (5.7) | 298 (12.7) | 15 (2.2) | 2449 (3.3) | 89 (7.1) | 586 (5.4) | 521 (7.3) | 110 (7.2) |
| Appropriate (5th – 95th percentile) | 98330 (84.2) | 8868 (84.7) | 1938 (82.7) | 688 (95.3) | 61786 (82.7) | 1122 (89.1) | 9680 (89.5) | 6185 (86.1) | 1363 (89.1) |
| Large (>95th percentile) | 12640 (10.9) | 1004 (9.6) | 108 (4.6) | 19 (2.5) | 10377 (14.0) | 48 (3.8) | 553 (5.1) | 475 (6.6) | 56 (3.7) |
| *Missing n (%)* | *540 (0.5)* | *36 (0.3)* | *2 (0.1)* | *1 (0.1)* | *281 (0.4)* | 0 (0.0) | *70 (0.6)* | *150 (2.0)* | *0 (0.0)* |
| Sex of the child, *n (%)* |  |  |  |  |  |  |  |  |  |
| Boy | 53180 (50.1) | 5403 (51.2) | 1169 (50.7) | 340 (47.0) | 37026 (49.4) | 667 (53.0) | 5599 (51.4) | 3694 (50.4) | 781 (51.0) |
| Girl | 52998 (49.3) | 5108 (48.8) | 1177 (49.3) | 383 (53.0) | 37866 (50.6) | 592 (47.0) | 5287 (48.6) | 3637 (49.6) | 748 (49.0) |
| *Missing n (%)* | *3 (0.0)* | *0 (0.0)* | *0 (0.0)* | *0 (0.0)* | *0 (0.0)* | *0 (0.0)* | *3 (0.0)* | *0 (0.0)* | *0 (0.0)* |
| **Childhood BMI** |  |  |  |  |  |  |  |  |  |
| Age in months, median (IQR) | 24.0 (23.1, 24.4) | 25.0 (25.0, 25.0) | 24.0 (23.0, 25.0) | 24.0 (23.0, 24.0) | 18.0 (18.0, 18.0) | 24.0 (23.0, 24.0) | 24.0 (23.0, 24.0) | 24.0 (24.0, 25.0) | 23.0 (18.0, 24.0) |
| *Missing n (%)* | 90219 (82.4) | *9382 (89.3)* | *597 (25.4)* | *24 (3.3)* | *74826 (99.9)* | *75 (6.0)* | *2933 (26.9)* | *2036 (27.8)* | *346 (22.6)* |
| Weight in kg, median (IQR) | 12.3 (11.4, 13.3) | 12.6 (11.6, 13.5) | 12.4 (11.3, 13.5) | 12.3 (11.6, 13.2) | 11.4 (10.5, 12.0) | 12.2 (11.3, 13.0) | 12.1 (11.2, 13.0) | 12.8 (11.8, 13.8) | 11.8 (10.9, 12.9) |
| *Missing n (%)* | *90219 (82.4)* | *9382 (89.3)* | *597 (25.4)* | *24 (3.3)* | *74826 (99.9)* | *75 (6.0)* | *2933 (26.9)* | *2036 (27.8)* | *346 (22.6)* |
| Height in cm, median (IQR) | 87.2 (84.9, 89.8) | 86.5 (84.5, 88.7) | 86.7 (84.4, 89.0) | 88.0 (86.0, 90.0) | 82.0 (80.5, 85.0) | 87.5 (85.0, 90.0) | 87.0 (85.0, 90.0) | 88.0 (85.5, 90.2) | 85.0 (82.5, 88.0) |
| *Missing n (%)* | 91009 (83.1) | *9453 (90.0)* | *1097 (46.8)* | *36 (5.0)* | *74826 (99.9)* | *107 (8.5)* | *3045 (28.0)* | *2124 (29.0)* | *353 (23.1)* |
| BMI in kg/m^2^, median (IQR) | 16.2 (15.4, 17.1) | 16.7 (15.9, 17.6) | 16.5 (15.6, 17.5) | 15.9 (15.2, 16.8) | 16.6 (15.7, 17.6) | 16.0 (15.1, 16.8) | 15.9 (15.  0, 16.8) | 16.5 (15.7, 17.4) | 16.4 (15.5, 17.3) |
| *Missing n (%)* | 91009 (83.1) | *9453 (90.0)* | *1097 (46.8)* | *36 (5.0)* | *74826 (99.9)* | *107 (8.5)* | *3045 (28.0)* | *2124 (29.0)* | *353 (23.1)* |
| BMI 1st tertile, median (IQR) | 15.01 (14.5, 15.4) | *15.5 (15.1, 15.9)* | *15.2 (14.6, 15.6)* | *14.8 (14.4, 15.2)* | *15.5 (14.8, 15.6)* | *14.8 (14.3, 15.1)* | *14.7 (14.3, 15.0)* | *15.3 (14.8, 15.7)* | *15.1 (14.6, 15.5)* |
| BMI 2^nd^ tertile, median (IQR) | 16.2 (15.9, 16.5) | *16.7 (16.5, 17.0)* | *16.5 (16.3, 16.8)* | *15.9 (15.7, 16.2)* | *16.6 (16.4, 17.0)* | *16.0 (15.7, 16.2)* | *15.9 (15.6, 16.2)* | *16.5 (16.3, 16.8)* | *16.4 (16.1, 16.6)* |
| BMI 3^rd^ tertile, median (IQR) | 17.5 (17.1, 18.1) | *18.1 (17.6, 18.7)* | *18.0 (17.6, 18.7)* | *17.2 (16.8, 17.8)* | *18.3 (17.6, 19.0)* | *17.2 (16.8, 17.7)* | *17.2 (16.8, 17.8)* | *17.8 (17.4, 18.5)* | *17.7 (17.3, 18.3)* |
| Values are median (IQR), or number (valid %). Characteristics are based on observed not imputed data. BMI: body mass index. The population sample comprises of participants with complete data on birth weight and at least one outcome measurement available. | | | | | | | | | |

| **Table S2. Emotional, behaviour and cognitive outcomes descriptives** | | | | | | | | | |
| --- | --- | --- | --- | --- | --- | --- | --- | --- | --- |
|  | **All cohorts**  **(n=109481)** | **ALSPAC, United Kingdom**  **(n=10511)** | **BIB, United Kingdom**  **(n=2346)** | **CHOP,**  **Germany**  **(n=723)** | **DNBC,**  **Denmark**  **(n=74893)** | **EDEN,**  **France**  **(n=1259)** | **ELFE,**  **France**  **(n=10889)** | **Generation R**  **The Netherlands**  **(n=7331)** | **INMA,**  **Spain**  **(n=1529)** |
| **Internalizing problems percentiles,** median (IQR) |  |  |  |  |  |  |  |  |  |
| Childhood age 4 – 10 years | 47·0 (8·0, 67·8) | 44·0 (22·0, 72·0) | 51·0 (23·0, 75·0) | 42·5 (22·8, 68·9) | 47·0 (1·0, 64·0) | 48·0 (19·0, 78·0) | 46·0 (12·0, 73·0) | 51·5 (28·3, 77·9) | 47·0 (19·0, 73·0) |
| *Missing n (%)* | *20477 (18.7)* | *520 (4.9)* | *0 (0.0)* | *42 (5.8)* | *18898 (25.2)* | *45 (3.6)* | *2 (0.0)* | *726 (9.9)* | *244 (16.0)* |
| Early adolescence age 11 – 16 years | 43·3 (23·9, 71·3) | 45·0 (24·0, 73·0) | NA | 40·9 (22·4, 69·2) | 43·0 (24·0, 71·0) | NA | NA | 60·4 (28·3, 88·1) | 40·0 (14·0, 68·0) |
| *Missing n (%)* | *40009 (36.5)* | *2613 (24.9)* | *NA* | *283 (39.1)* | *28609 (38.2)* | *NA* | *NA* | *7274 (99.2)* | *1230 (80.4)* |
| Late adolescence age 17 – 20 years | 47·9 (24·0, 68·2) | 46·0 (24·0, 74·0) | NA | NA | 48·0 (24·0, 68·0) | NA | NA | NA | NA |
| *Missing n (%)* | *37882 (34.6)* | *8680 (82.3)* | *NA* | *NA* | *29202 (39.0)* | *NA* | *NA* | *NA* | *NA* |
| **Externalizing problems percentiles,** median (IQR) |  |  |  |  |  |  |  |  |  |
| Childhood age 4 – 10 years | 44·9 (17·3, 71·1) | 49·0 (21·0, 72·0) | 45·0 (25·0, 74·0) | 41·6 (22·6, 71·3) | 43·0 (14·0, 70·0) | 52·0 (25·0, 75·0) | 45·0 (22·0, 70·0) | 53·5 (26·2, 79·0) | 44·0 (19·8, 72·0) |
| *Missing n (%)* | *20506 (18.7)* | *521 (5.0)* | *0 (0.0)* | *41 (5.7)* | *18935 (25.3)* | *46 (3.7)* | *1 (0.0)* | *717 (9.8)* | *245 (16.0)* |
| Early adolescence age 11 – 16 years | 40·7 (20·3, 74·5) | 44·0 (22·0, 72·0) | NA | 46·4 (22·7, 76·6) | 40·0 (20·0, 75·0) | NA | NA | 51·9 (26·2, 74·6) | 47·0 (20·0, 69·0) |
| *Missing n (%)* | *40007(36.5)* | *2611 (24.8)* | *NA* | *283 (39.1)* | *28609 (38.2)* | *NA* | *NA* | *7274 (99.2)* | *1230 (80.4)* |
| Late adolescence age 17 – 20 years | 44·0 (17·8, 68·1) | 43·0 (14·0, 70·0) | NA | NA | 44·0 (18·0, 68·0) | NA | NA | NA | NA |
| *Missing n (%)* | *37881 (34.6)* | *8679 (82.6)* | *NA* | *NA* | *29202 (39.0)* | *NA* | *NA* | *NA* | *NA* |
|  |  |  |  |  |  |  |  |  |  |
| **ADHD score percentiles,** median (IQR) |  |  |  |  |  |  |  |  |  |
| Childhood age 4 – 10 years | 43·9 (20·3, 73·2) | 48·0 (13·0, 66·0) | NA | 49·1 (20·2, 72·3) | 43·0 (22·0, 75·0) | 53·0 (23·0, 78·0) | 40·0 (14·0, 69·0) | 50·1 (28·2, 75·4) | 47·0 (24·0, 73·0) |
| *Missing n (%)* | *22353 (20.4)* | *521 (5.0)* | *NA* | *120 (16.6)* | *18947 (25.3)* | *45 (3.6)* | *2 (0.0)* | *2677 (36.5)* | *41 (2.7)* |
|  | All cohorts  (n=109481) | ALSPAC, United Kingdom  (n=10511) | BIB, United Kingdom  (n=2346) | CHOP,  Germany  (n=723) | DNBC,  Denmark  (n=74893) | EDEN,  France  (n=1259) | ELFE,  France  (n=10889) | Generation R  The Netherlands  (n=7331) | INMA,  Spain  (n=1529) |
| Early adolescence age 11 – 16 years | 46·2 (4·0, 68·1) | 47·0 (19·0, 74·0) | NA | 50·0 (23·0, 70·4) | 46·0 (1·0, 67·0) | NA | NA | NA | 50·0 (24·0, 74·0) |
| *Missing n (%)* | *32559 (29.7)* | *2615 (24.9)* | *NA* | *283 (39.1)* | *28609 (38.2)* | *NA* | *NA* | *NA* | *1052 (68.8)* |
| Late adolescence age 17 – 20 years | 38·9 (21·8, 70·2) | 36·0 (18·0, 75·0) | NA | NA | 39·0 (22·0, 70·0) | NA | NA | NA | NA |
| *Missing n (%)* | *37882 (34.6)* | *8682 (82.6)* | *NA* | *NA* | *29200 (39.0)* | *NA* | *NA* | *NA* | *NA* |
| **ASD score percentiles,** median (IQR) |  |  |  |  |  |  |  |  |  |
| Childhood age 4 – 10 years | 53·0 (24·9, 73·7) | NA | NA | NA | NA | NA | NA | 54·2 (26·3, 75·1) | 48·0 (19·0, 68·0) |
| *Missing n (%)* | *2440 (2.2)* | *NA* | *NA* | *NA* | *NA* | *NA* | *NA* | *2137 (29.2)* | *303 (19.8)* |
| **Non-verbal IQ score percentiles,** median (IQR) |  |  |  |  |  |  |  |  |  |
| Childhood age 4 – 10 years | 52·7 (25·4, 74·8) | 49·0 (25·0, 75·0) | NA | 52·1 (27·0, 74·2) | NA | 52·0 (27·0, 74·0) | NA | 58·7 (26·4, 75·3) | 47·0 (21·0, 72·0) |
| *Missing n (%)* | *15779 (14.4)* | *3309 (31.5)* | *NA* | *211 (29.2)* | *NA* | *63 (5.0)* | *NA* | *1276 (17.4)* | *41 (2.7)* |
| Values are median (IQR), or number (valid %). Characteristics are based on observed not imputed data. BMI: body mass index, ADHD: attention-deficit hyperactivity disorder, ASD: autism spectrum disorder, Non-verbal IQ: non-verbal intelligence. | | | | | | | | | |

**Table S3. Descriptive statistics of the emotional, behaviour and cognitive outcomes in each cohort**

|  | **ALSPAC**  **(n=10511)** | **BIB**  **(n=2346)** | **CHOP**  **(n=723)** | **DNBC**  **(n=74891)** | **EDEN**  **(n=1259)** | **ELFE**  **(n=10889)** | **GENR**  **(n=7331)** | **INMA**  **(n=1529)** |
| --- | --- | --- | --- | --- | --- | --- | --- | --- |
| **Internalizing problems score in percentiles,** median (IQR) |  |  |  |  |  |  |  |  |
| Age 4 | 50·0 (14·0, 66·0) | 51·0 (23·0, 75·0) | NA | NA | NA | NA | 56·4 (22·1, 73·9) | NA |
| *Missing n (%)* | *5912 (56.2)* | *77 (3.3)* | *NA* | *NA* | *NA* | *NA* | *7316 (99.8)* | *NA* |
| Age 5 | 42·0 (21·0, 67·0) | 44·0 (11·0, 69·0) | 38·6 (23·7, 67·0) | NA | 53·0 (19·0, 78·0) | 47·0 (12·0, 73·0) | (51·5 (32·1, 75·5) | NA |
| *Missing n (%)* | *10487 (99.8)* | *2269 (96.7)* | *135 (0.2)* | *NA* | *119 (9.5)* | *4736 (43.5)* | *3768 (51.4)* | *NA* |
| Age 6 | 44·0 (22·0, 73·0) | NA | NA | NA | 53·0 (19·0, 85·0) | 47·0 (12·0, 73·0) | 58·4 (32·1, 79·6) | NA |
| *Missing n (%)* | *2850 (27.1)* | *NA* | *NA* | *NA* | *1223 (97.1)* | *6155 (56.5)* | *5017 (68.4)* | *NA* |
| Age 7 | 42·0 (23·0, 72·0) | NA | 43·9 (21·7, 68·8) | 47·0 (1·0, 64·0) | 48·0 (19·0, 79·0) | NA | 65·4 (32·1, 79·6) | 49·0 (18·0, 74·0) |
| *Missing n (%)* | *9954 (94.7)* | *NA* | *557 (77.0)* | *18997 (25.4)* | *933 (74.1)* | *NA* | *7126 (97.2)* | *1133 (74.1)* |
| Age 8 | 37·0 (20·0, 67·0) | NA | 41·6 (23·0, 69·7) | 33·0 (1·0, 66·0) | 48·0 (33·0, 79·0) | NA | 58·4 (42·36, 79·6) | 48·0 (19·0, 73·0) |
| *Missing n (%)* | *3089 (29.4)* | *NA* | *367 (4.9)* | *74794 (99.9)* | *710 (56.4)* | *NA* | *7253 (98.9)* | *1144 (74.8)* |
| Age 9 | 44·0 (23·0, 72·0) | NA | NA | NA | NA | NA | 51·5 (28·3, 77·9) | 43·0 (21·0, 71·0) |
| *Missing n (%)* | *3062 (29.1)* | *NA* | *NA* | *NA* | *NA* | *NA* | *2872 (39.2)* | *1052 (68.8)* |
| Age 10 | 40·0 (7·25, 74·0) | NA | 34·2 (21·9, 73·4) | NA | NA | NA | 51·5 (28·3, 77·9) | 47·0 (14·0, 73·0) |
| *Missing n (%)* | *10301 (98.0)* | *NA* | *646 (89.3)* | *NA* | *NA* | *NA* | *6991 (95.4)* | *1029 (67.3)* |
| Age 11 | 46·0 (25·0, 74·0) | NA | 40·9 (22·4, 69·2) | 43·0 (24·0, 71·0) | NA | NA | 51·5 (28·3, 88·1) | 40·0 (14·0, 68·0) |
| *Missing n (%)* | *3783 (36.0)* | *NA* | *283 (39.1)* | *36817 (49.2)* | *NA* | *NA* | *7284 (99.4)* | *1253 (81.9)* |
| Age 12 | 43·0 (1·0, 69·0) | NA | NA | 46·0 (26·0, 73·0) | NA | NA | 69·1 (43·5, 84·7) | 48·0 (22·0, 61·0) |
| *Missing n (%)* | *10299 (98.0)* | *NA* | *NA* | *67947 (90.7)* | *NA* | *NA* | *7321 (99.9)* | *1506 (98.5)* |
| Age 13 | 45·0 (24·0, 73·0) | NA | NA | 45·0 (7·5, 73·0 | NA | NA | NA | NA |
| *Missing n (%)* | *3898 (37.1)* | *NA* | *NA* | *73775 (98.5)* | *NA* | *NA* | *NA* | *NA* |
| Age 14 | 47·0 (1·0, 59·0) | NA | NA | 46·0 (25·0, 71·0) | NA | NA | NA | NA |
| *Missing n (%)* | *10458 (99.5)* | *NA* | *NA* | *74749 (99.8)* | *NA* | *NA* | *NA* | *NA* |
| Age 15 | 47·0 (1·0, 59·0) | NA | NA | 46·0 (25·0, 71·0) | NA | NA | NA | NA |
| *Missing n (%)* | *10458 (99.5)* | *NA* | *NA* | *74749 (99.8)* | *NA* | *NA* | *NA* | *NA* |
| Age 16 | 45·0 (24·0, 73·0) | NA | NA | NA | NA | NA | NA | NA |
| *Missing n (%)* | *6950 (66.1)* | *NA* | *NA* | *NA* | *NA* | *NA* | *NA* | *NA* |
| Age 17 | 46·0 (24·0, 74·0) | NA | NA | NA | NA | NA | NA | NA |
| *Missing n (%)* | *8689 (82.7)* | *NA* | *NA* | *NA* | *NA* | *NA* | *NA* | *NA* |
| Age 18 | 37·0 (1·0, 55·0) | NA | NA | 48·0 (24·0, 68·0) | NA | NA | NA | NA |
| *Missing n (%)* | *10502 (99.9)* | *NA* | *NA* | *29474 (39.4)* | *NA* | *NA* | *NA* | *NA* |
| Age 19 | NA | NA | NA | 50·0 (16·0, 73·0) | NA | NA | NA | NA |
| *Missing n (%)* | *NA* | *NA* | *NA* | *74629 (99.7)* | *NA* | *NA* | *NA* | *NA* |
| Age 20 | NA | NA | NA | 38·0 (22·8, 66·3) | NA | NA | NA | NA |
| *Missing n (%)* | *NA* | *NA* | *NA* | *74885 (99.9)* | *NA* | *NA* | *NA* | *NA* |
| **Externalizing problems score in percentiles,** median (IQR) |  |  |  |  |  |  |  |  |
| Age 4 | 49·0 (25·0, 72·0) | 45·0 (25·0, 74·0) | NA | NA | NA | NA | 47·0 (29·1, 66·3) | NA |
| *Missing n (%)* | *5912 (56.2)* | *77 (3.3)* | *NA* | *NA* | *NA* | *NA* | *7316 (99.8)* | *NA* |
| Age 5 | 42·0 (17·0, 71·0) | 45·0 (21·0, 65·0 ) | 48·2 (20·1, 73·0) | NA | 52·0 (24·8, 77·0) | 45·0 (22·0, 75·0) | 53·5 (25·3, 77·6) | NA |
| *Missing n (%)* | *10487 (99.8)* | *2269 (96.7)* | *134 (18.5)* | *NA* | *119 (9.5)* | *4735 (43.5)* | *3755 (51.2)* | *NA* |
| Age 6 | 50·0 (15·0, 72·0) | NA | NA | NA | 61·0 (31·0, 87·0) | 49·0 (25·0, 70·0) | 53·5 (33·2, 77·6) | NA |
| *Missing n (%)* | *2850 (27.1)* | *NA* | *NA* | *NA* | *1223 (97.1)* | *6155 (56.5)* | *5009 (68.3)* | *NA* |
| Age 7 | 47·0 (25·0, 75·0) | NA | 37·8 (22·6, 66·6) | 43·0 (14·0, 70·0) | 47·0 (25·0, 75·0) | NA | 53·5 (33·2, 73·8) | 45·0 (24·0, 71·0) |
| *Missing n (%)* | *9954 (94.7)* | *NA* | *557 (77.0)* | *19034 (25.4)* | *933 (74.1)* | *NA* | *7125 (97.2)* | *1133 (74.1)* |
| Age 8 | 41·0 (18·0, 73·0) | NA | 44·9 (25·8, 70·4) | 43·0 (10·0, 75·0) | 47·0 (25·0, 75·0) | NA | 59·1 (33·2, 77·6) | 48·0 (25·0, 75·0) |
| *Missing n (%)* | *3091 (29.4)* | *NA* | *366 (50.6)* | *74794 (99.9)* | *710 (56.4)* | *NA* | *7253 (98.9)* | *1145 (74.9)* |
| Age 9 | 49·0 (21·0, 72·0) | NA | NA | NA | NA | NA | 51·9 (26·2, 74·9) | 49·0 (25·0, 72·0) |
| *Missing n (%)* | *3063 (29.1)* | *NA* | *NA* | *NA* | *NA* | *NA* | *2874 (39.2)* | *1052 (68.9)* |
| Age 10 | 47·0 (18·0, 65·0) | NA | 41·6 (23·4, 71·3) | NA | NA | NA | 61·2 (40·8, 79·0) | 44·0 (18·0, 75·0) |
| *Missing n (%)* | *10301 (98.0)* | *NA* | *646 (89.3)* | *NA* | *NA* | *NA* | *6990 (95.3)* | *1029 (67.3)* |
| Age 11 | 38·0 (24·0, 75·0) | NA | 46·4 (22·7, 76·6) | 36·0 (20·0, 75·0) | NA | NA | 51·9 (26·2, 84·4) | 47·0 (20·0, 69·0) |
| *Missing n (%)* | *3776 (35.9)* | *NA* | *283 (39.1)* | *36817 (49.2)* | *NA* | *NA* | *7284 (99.4)* | *1253 (81.9)* |
| Age 12 | 44·0 (21·0, 71·0) | NA | NA | 40·0 (23·0, 68·0) | NA | NA | 33·5 (26·2, 61·2) | 48·0 (18·0, 70·0) |
| *Missing n (%)* | *10299 (98.0)* | *NA* | *NA* | *67947 (90.7)* | *NA* | *NA* | *7321 (99.9)* | *1506 (98.5)* |
| Age 13 | 41·0 (25·0, 70·0) | NA | NA | 41·0 (25·0, 70·0) | NA | NA | NA | NA |
| *Missing n (%)* | *3897 (37.1)* | *NA* | *NA* | *73775 (98.5)* | *NA* | *NA* | *NA* | *NA* |
| Age 14 | 34·0 (22·0, 70·0) | NA | NA | 41·0 (25·0, 71·0) | NA | NA | NA | NA |
| *Missing n (%)* | *10458 (99.5)* | *NA* | *NA* | *74749 (99.8)* | *NA* | *NA* | *NA* | *NA* |
| Age 15 | NA | NA | NA | NA | NA | NA | NA | NA |
| *Missing n (%)* | *NA* | *NA* | *NA* | *NA* | *NA* | *NA* | *NA* | *NA* |
| Age 16 | 44·0 (14·0, 71·0) | NA | NA | NA | NA | NA | NA | NA |
| *Missing n (%)* | *6948 (66.1)* | *NA* | *NA* | *NA* | *NA* | *NA* | *NA* | *NA* |
| Age 17 | 43·0 (14·0, 70·0) | NA | NA | NA | NA | NA | NA | NA |
| *Missing n (%)* | *8688 (82.7)* | *NA* | *NA* | *NA* | *NA* | *NA* | *NA* | *NA* |
| Age 18 | 28·0 (1·0, 55·0) | NA | NA | 44·0 (18·0, 68·0) | NA | NA | NA | NA |
| *Missing n (%)* | *10502 (99.9)* | *NA* | *NA* | *29474 (39.4)* | *NA* | *NA* | *NA* | *NA* |
| Age 19 | NA | NA | NA | 49·0 (18·0, 72·0) | NA | NA | NA | NA |
| *Missing n (%)* | *NA* | *NA* | *NA* | *74629 (99.7)* | *NA* | *NA* | *NA* | *NA* |
| Age 20 | NA | NA | NA | 44·50 (22·8, 57·3) | NA | NA | NA | NA |
| *Missing n (%)* | *NA* | *NA* | *NA* | *74885 (99.9)* | *NA* | *NA* | *NA* | *NA* |
| **ADHD symptoms score in percentiles,** median (IQR) |  |  |  |  |  |  |  |  |
| Age 4 | 46·0 (15·0, 62·0) | NA | NA | NA | NA | NA | NA | 48·0 (23·0, 75·0) |
| *Missing n (%)* | *5913 (56.3)* | *NA* | *NA* | *NA* | *NA* | *NA* | *NA* | *792 (51.8)* |
| Age 5 | 46·0 (13·0, 63·0) | NA | 48·1 (20·2, 72·3) | NA | 54·0 (24·0, 79·0) | 40·0 (24·0, 69·0) | NA | 50·0 (17·0, 74·0) |
| *Missing n (%)* | *10487 (99.8)* | *NA* | *132 (18.3)* | *NA* | *118 (9.4)* | *4735 (43.5)* | *NA* | *1184 (77.4)* |
| Age 6 | 42·0 (25·0, 71·0) | NA | NA | NA | 54·0 (20·0, 81·3) | 46·0 (14·0, 73·0) | NA | 45·0 (25·0, 75·0) |
| *Missing n (%)* | *2851 (27.1)* | *NA* | *NA* | *NA* | *1223 (97.1)* | *6156 (56.5)* | *NA* | *1154 (75.5)* |
| Age 7 | 37·0 (23·0, 68·0) | NA | NA | 43·0 (22·0, 75·0) | 42·0 (23·0, 67·0) | NA | 55·9 (28·2, 79·6) | 46·0 (24·0, 73·0) |
| *Missing n (%)* | *9954 (94.7)* | *NA* | *NA* | *19046 (25.4)* | *933 (74.1)* | *NA* | *7300 (99.6)* | *569 (37.2)* |
| Age 8 | 50·1 (28·2, 75·4) | NA | NA | 42·0 (22·0, 74·0) | 53·0 (23·0, 78·0) | NA | 50·1 (28·2, 75·4) | 47·0 (22·0, 73·0) |
| *Missing n (%)* | *3093 (29.4)* | *NA* | *NA* | *74794 (99.9)* | *710 (56.4)* | *NA* | *2760 (37.6)* | *1501 (98.2)* |
| Age 9 | 49·0 (13·0, 66·0) | NA | NA | NA | NA | NA | 50·1 (28·2, 78·3) | 48·0 (18·0, 67·5) |
| *Missing n (%)* | *3066 (29.2)* | *NA* | *NA* | *NA* | *NA* | *NA* | *7281 (99.3)* | *1506 (98.5)* |
| Age 10 | 32·0 (12·0, 67·0) | NA | 52·4 (24·6, 76·0) | NA | NA | NA | 58·3 (54·2, 62·3) | 58·3 (54·2, 62·3) |
| *Missing n (%)* | *10301 (98.0)* | *NA* | *646 (89.3)* | *NA* | *NA* | *NA* | *7329 (99.9)* | *789 (51.6)* |
| Age 11 | 34·0 (16·0, 70·0) | NA | 50·0 (23·0, 70·4) | 46·0 (1·0, 63·0) | NA | NA | NA | 50·0 (24·0, 74·0) |
| *Missing n (%)* | *3787 (36.0)* | *NA* | *283 (39.1)* | *36817 (49.2)* | *NA* | *NA* | *NA* | *1077 (70.4)* |
| Age 12 | 44·0 (15·0, 68·8) | NA | NA | 50·0 (1·0, 67·0) | NA | NA | NA | 45·0 (25·0, 65·0) |
| *Missing n (%)* | *10299 (98.0)* | *NA* | *NA* | *67947 (90.7)* | *NA* | *NA* | *NA* | *1504 (98.4)* |
| Age 13 | 48·0 (14·0, 67·0) | NA | NA | 33·0 (1·0, 68·0) | NA | NA | NA | NA |
| *Missing n (%)* | *3899 (37.1)* | *NA* | *NA* | *73775 (98.5)* | *NA* | *NA* | *NA* | *NA* |
| Age 14 | 49·0 (11·0, 72·0) | NA | NA | 31·0 (1·0, 71·0) | NA | NA | NA | NA |
| *Missing n (%)* | *10458 (99.5)* | *NA* | *NA* | *74749 (99.8)* | *NA* | *NA* | *NA* | *NA* |
| Age 15 | NA | NA | NA | NA | NA | NA | NA | NA |
| *Missing n (%)* | *NA* | *NA* | *NA* | *NA* | *NA* | *NA* | *NA* | *NA* |
| Age 16 | 37·0 (19·0, 47·0) | NA | NA | NA | NA | NA | NA | NA |
| *Missing n (%)* | *6951 (66.1)* | *NA* | *NA* | *NA* | *NA* | *NA* | *NA* | *NA* |
| Age 17 | 36·0 (18·0, 75·0) | NA | NA | NA | NA | NA | NA | NA |
| *Missing n (%)* | *8691 (82.7)* | *NA* | *NA* | *NA* | *NA* | *NA* | *NA* | *NA* |
| Age 18 | 37·0 (1·0, 46·0) | NA | NA | 39·0 (22·0, 70·0) | NA | NA | NA | NA |
| *Missing n (%)* | *10502 (99.9)* | *NA* | *NA* | *29472 (39.4)* | *NA* | *NA* | *NA* | *NA* |
| Age 19 | NA | NA | NA | 48·0 (22·0, 64·0) | NA | NA | NA | NA |
| *Missing n (%)* | *NA* | *NA* | *NA* | *74629 (99.7)* | *NA* | *NA* | *NA* | *NA* |
| Age 20 | NA | NA | NA | 38·5 (19·8, 57·3) | NA | NA | NA | NA |
| *Missing n (%)* | *NA* | *NA* | *NA* | *74885 (99.9)* | *NA* | *NA* | *NA* | *NA* |
| **ASD symptoms score in percentiles,** median (IQR) |  |  |  |  |  |  |  |  |
| Age 4 | NA | NA | NA | NA | NA | NA | 95·3 (95·3, 95·3) | 48·0 (19·0, 64·0) |
| *Missing n (%)* | *NA* | *NA* | *NA* | *NA* | *NA* | *NA* | *7330 (99.9)* | *837 (54.7)* |
| Age 5 | NA | NA | NA | NA | NA | NA | 54·2 (26·3, 74·7) | 49·0 (16·0, 68·0) |
| *Missing n (%)* | *NA* | *NA* | *NA* | *NA* | *NA* | *NA* | *5001 (68.2)* | *1026 (67.1)* |
| Age 6 | NA | NA | NA | NA | NA | NA | 54·2 (26·6, 81·5) | 46·0 (4·0, 65·0) |
| *Missing n (%)* | *NA* | *NA* | *NA* | *NA* | *NA* | *NA* | *4780 (65.2)* | *1498 (98.0)* |
| Age 7 | NA | NA | NA | NA | NA | NA | 66·0 (40·0, 89·4) | NA |
| *Missing n (%)* | *NA* | *NA* | *NA* | *NA* | *NA* | *NA* | *7089 (96.7)* | *NA* |
| Age 8 | NA | NA | NA | NA | NA | NA | 70·5 (40·0, 86·1) | NA |
| *Missing n (%)* | *NA* | *NA* | *NA* | *NA* | *NA* | *NA* | *7261 (99.0)* | *NA* |
| Age 9 | NA | NA | NA | NA | NA | NA | 54·7 (40·0, 70·3) | NA |
| *Missing n (%)* | *NA* | *NA* | *NA* | *NA* | *NA* | *NA* | *7292 (99.5)* | *NA* |
| **Non-verbal IQ score percentiles,** median (IQR) |  |  |  |  |  |  |  |  |
| Age 4 | 49·0 (25·0, 75·0) | NA | NA | NA | 74·0 (22·0, 74·0) | NA | 26·4 (21·0, 34·5) | 46·0 (26·0, 73·0) |
| *Missing n (%)* | *9506 (90.4)* | *NA* | *NA* | *NA* | *538 (72.7)* | *NA* | *7327 (99.9)* | *655 (42.8)* |
| Age 5 | NA | NA | 52·1 (27·0, 74·2) | NA | 52·0 (27·0, 77·0) | NA | 58·7 (26·4, 75·3) | 50·0 (24·0, 73·0) |
| *Missing n (%)* | *NA* | *NA* | *211 (29.2)* | *NA* | *191 (15.2)* | *NA* | *4559 (62.2)* | *1027 (67.2)* |
| Age 6 | NA | NA | NA | NA | 29·5 (18·0, 45·0) | NA | 58·7 (42·1, 88·5) | 44·0 (19·0, 72·0) |
| *Missing n (%)* | *NA* | *NA* | *NA* | *NA* | *1221 (97.0)* | *NA* | *4485 (61.2)* | *1497 (97.9)* |
| Age 7 | NA | NA | NA | NA | NA | NA | 58·7 (42·1, 88·5) | 47·0 (20·0, 74·0) |
| *Missing n (%)* | *NA* | *NA* | *NA* | *NA* | *NA* | *NA* | *7023 (95.8)* | *1204 (78.7)* |
| Age 8 | 49·0 (25·0, 75·0) | NA | NA | NA | NA | NA | 75·3 (58·7, 88·5) | 48·0 (23·0, 65·0) |
| *Missing n (%)* | *3547 (33.7)* | *NA* | *NA* | *NA* | *NA* | *NA* | *7210 (98.3)* | *1214 (79.4)* |
| Age 9 | NA | NA | NA | NA | NA | NA | 42·1 (42·1, 42·1) | 47·0 (23·0, 72·0) |
| *Missing n (%)* | *NA* | *NA* | *NA* | *NA* | *NA* | *NA* | *7330 (99.9)* | *1342 (87.8)* |
| Age 10 | NA | NA | NA | NA | NA | NA | NA | 42·0 (21·0, 64·0) |
| *Missing n (%)* | *NA* | *NA* | *NA* | *NA* | *NA* | *NA* | *NA* | *1291 (84.4)* |
| Values are median (IQR), or number (valid %). Characteristics are based on observed not imputed data. BMI: body mass index, ADHD: attention-deficit hyperactivity disorder, ASD: autism spectrum disorder, Non-verbal IQ: non-verbal intelligence. | | | | | | | | |

**Table S4. Birth characteristics and internalizing and externalizing problems score at school-age, basic model**

|  | |  |  |  |  |  |  |  |  |
| --- | --- | --- | --- | --- | --- | --- | --- | --- | --- |
|  | **Internalizing problems** | | | | | **Externalizing problems** | | | |
|  | | Childhood  (n=89004) | | Early Adolescence  (n=54978) | Late Adolescence  (n=47522) | | Childhood  (n=88975) | Early Adolescence  (n=54980) | Late Adolescence  (n=47523) |
| Birth characteristics | | Difference in percentiles (95 CI) | | Difference in percentiles (95 CI) | Difference in percentiles (95 CI) | | Difference in percentiles (95 CI) | Difference in percentiles (95 CI) | Difference in percentiles (95 CI) |
| Gestational age at birth, weeks | | -0·55 (-0·66, -0·44)*** | | -0·25 (-0·40, -0·10)*** | -0·13 (-0·26, 0·01) | | -0·37 (-0·48, -0·26)*** | -0·32 (-0·46, -0·18)*** | 0·26 (0·12, 0·41)*** |
| N | | 109481 | | 94987 | 85404 | | 109481 | 94987 | 85404 |
| Gestational age in categories, weeks | |  | |  |  | |  |  |  |
| < 37 | | 4·73 (3·81, 5·64)*** | | 2·59 (1·39, 3·79)*** | 1·23 (0·13, 2·33)* | | 2·74 (1·89, 3·60)*** | 2·56 (1·40, 3·71)*** | -2·39 (-3·54, -1·24)*** |
| N | | 4780 | | 2873 | 2668 | | 4777 | 2873 | 2668 |
| 37-42 | | *Reference* | | *Reference* | *Reference* | | *Reference* | *Reference* | *Reference* |
| N | | *78605* | | *48345* | *42080* | | *78581* | *48347* | *42080* |
| >42 | | 1·58 (0·68, 2·49)*** | | 1·13 (0·01, 2·25)* | 0·53 (-0·56, 1·61) | | -0·53 (-1·38, 0·32) | -0·39 (-1·47, 0·69) | 0·16 (-0·97, 1·29) |
| N | | 4938 | | 3320 | 2774 | | 4935 | 3320 | 2775 |
| Birth weight, 500 gr | | -1·39 (-1·57, -1·21)*** | | -0·78 (-1·02, -055)*** | -0·32 (-0·53, -0·10)** | | -1·24 (-1·41, 1·07)*** | 1·15 (-1·38, -0·92)*** | 0·18 (-0·05, 0·41) |
| N | | 109481 | | 94987 | 85404 | | 109481 | 94987 | 85404 |
| Birth weight in categories, grams | |  | |  |  | |  |  |  |
| < 2500 | | 4·57 (3·57, 5·58)*** | | 2·82 (1·41, 4·22)*** | 1·22 (-0·06, 2·50) | | 4·40 (3·46, 5·34)*** | 3·95 (2·60, 5·30)*** | -1·80 (-3·14, -0·47)** |
| N | | 3887 | | 2054 | 1954 | | 3885 | 2054 | 1954 |
| 2500-4500 | | *Reference* | | *Reference* | *Reference* | | *Reference* | *Reference* | *Reference* |
| N | | *81751* | | *50179* | *43829* | | *81725* | *50181* | *43830* |
| > 4500 | | -1·21 (-2·41, 0·001) | | -0·10 ((-1·54, 1·35) | 2·10 (0·75, 3·45)** | | -1·78 (-2·91, -0·65)** | -1·24 (-2·63, 0·15) | 0·56 (-0·85, 1·97) |
| N | | 2685 | | 1949 | 1739 | | 2683 | 1949 | 1739 |
| Cohorts | | ALSPAC, BIB, CHOP†, DNBC, EDEN, ELFE, GENR, INMA | | ALSPAC, CHOP†, DNBC, GENR‡, INMA§ | ALSPAC, DNBC | | ALSPAC, BIB, CHOP†, DNBC, EDEN, ELFE, GENR‡, INMA§ | ALSPAC, CHOP†, DNBC, GENR‡, INMA§ | ALSPAC, DNBC |
| CI: Confidence interval, SDS: standard deviation score, **P* value <0·05 ***P* value <0·01 *** P value <0·0005. Childhood measurements are between 4-10 years old, early adolescence measurements are between 11-16 years old and adolescence between 17-20 years old. Values are regression coefficients (95% confidence interval) obtained from one stage meta-analysis and reflect the differences in internalizing problems (percentiles) for birth characteristics from pooled multiple imputed data. †CHOP cohort is not included in the birth weight and gestational age categories due to missing data on preterm born and low birth weight children. ‡§: GenerationR and INMA are not included in the analyses of birth weight categories and internalizing problems in early adolescence due to a small number of participants and DataSHIELD data security regulations. ‡GenerationR was due to the same issue not included in the analysis of gestational age and sex adjusted birth weight categories with internalizing problems in early adolescence. The basic model is adjusted for sex, age at outcome measurement and cohort effect. | | | | | | | | | |

**Table S5. Birth characteristics and ADHD symptoms, ASD symptoms and Non-verbal IQ score at school-age, basic model**

|  |  |  |  |  |  |  |
| --- | --- | --- | --- | --- | --- | --- |
|  | **ADHD symptoms** | | | | **ASD symptoms** | **Non-verbal IQ** |
|  | Childhood  (n=84782) | | Early Adolescence  (n=55097) | Late Adolescence  (n=47522) | Childhood  (n=6420) | Childhood  (n=16453) |
| Birth characteristics | Difference in percentiles (95 CI) | | Difference in percentiles (95 CI) | Difference in percentiles (95 CI) | Difference in percentiles (95 CI) | Difference in percentiles (95 CI) |
| Gestational age at birth, weeks | -0·43 (-0·55, -0·32)*** | | -0·30 (-0·44, -0·15)*** | 0·23 (0·08, 0·37)** | -0·48 (-0·85, -0·12)** | 0·75 (0·51, 1·00)*** |
| N | 107135 | | 87656 | 85404 | 8860 | 21353 |
| Gestational age in categories, weeks |  | |  |  |  |  |
| < 37 | 3·26 (2·36, 4·16)*** | | 2·59 (1·42, 3·77)*** | -1·88 (-3·04, -0·72)** | 1·35 (-1·89, 4·60) | -3·58 (-5·67, -1·49)*** |
| N | 4522 | | 2878 | 2668 | 279 | 780 |
| 37-42 | *Reference* | | *Reference* | *Reference* | *Reference* | *Reference* |
| N | *74977* | | *48461* | *42080* | *5392* | *13771* |
| >42 | -0·33 (-1·22, 0·57) | | -0·14 (-1·24, 0·95) | 0·24 (-0·90, 1·38) | 0·30 (-1·77, 2·38) | 1·14 (-0·47, 2·75) |
| N | 4680 | | 3318 | 2774 | 749 | 1390 |
| Birth weight, 500 gr | -1·40 (-1·58, -1·22)*** | | -1·18 (-1·41, -0·95)*** | 0·20 (-0·03, 0·43) | -1·14 (-1·74, -0·55)*** | 2·30 (1·89, 2·70)*** |
| N | 107135 | | 87656 | 85404 | 8860 | 21353 |
| Birth weight in categories, grams |  | |  |  |  |  |
| < 2500 | 5·15 (4·14, 6·16)*** | | 3·89 (2·52, 5·25)*** | -1·90 (-3·34, -0·55)** | 3·71 (0·82, 6·60)* | -5·68 (-7·71, -3·65)*** |
| N | 3531 | | 2078 | 1954 | 353 | 821 |
| 2500-4500 | *Reference* | | *Reference* | *Reference* | *Reference* | *Reference* |
| N | *78023* | | *50626* | *43829* | *5932* | *14845* |
| > 4500 | -1·79 (-2·97, -0·62)** | | -1·19 (-2·60, 0·23) | 0·46 (-0·97, 1·88) | 2·90 (-1·71, 7·50) | 4·08 (0·62, 7·54)* |
| N | 2625 | | 1953 | 1739 | 135 | 275 |
| Cohorts | ALSPAC, CHOP†, DNBC, EDEN, ELFE, GENR, INMA | | ALSPAC, CHOP†, DNBC, INMA | ALSPAC, DNBC | GENR, INMA | ALSPAC, CHOP, EDEN, GENR, INMA |
| ADHD: Attention-deficit hyperactivity disorder, ASD: Autism Spectrum Disorder, Non-verbal IQ: Non Verbal Intelligence, CI: Confidence interval, SDS: standard deviation score, **P* value <0·05 ***P* value <0·01 *** P value <0·0005. Childhood measurements are between 4-10 years old, early adolescence measurements are between 11-16 years old and adolescence between 17-20 years old. Values are regression coefficients (95% confidence interval) obtained from one stage meta-analysis and reflect the differences in ADHD symptoms (percentiles), ASD symptoms (percentiles) and Non-verbal IQ score (percentiles) for birth characteristics from pooled multiple imputed data. †CHOP cohort is not included in the birth weight and gestational age categories due to missing data on preterm born and low birth weight children. The basic model is adjusted for sex, age at outcome measurement and cohort effect. | | | | | | |

**Table S6. Size for gestational age at birth categories and emotional, behaviour and cognitive outcomes in childhood, basic model**

| Size for gestational age at birth, SD score | Internalizing  Problems | | Externalizing  problems | | ADHD symptoms | | Intelligence  score | |
| --- | --- | --- | --- | --- | --- | --- | --- | --- |
|  | N | Difference in percentiles (95 CI) | N | Difference in percentiles (95 CI) | N | Difference in percentiles (95 CI) | N | Difference in percentiles (95 CI) |
| Small < 5th percentile | 3909 | 3·77 (2·76, 4·77)** | 3908 | 4·84 (3·89, 5·78)** | 3470 | 6·13 (5·11, 7·15)** | 1013 | -7·55 (-9·39, -5·71)** |
| Appropriate 5^th^-95^th^ percentile | *75169* | *Reference* | *75144* | *Reference* | *71594* | *Reference* | *14252* | *Reference* |
| Large > 95^th^ percentile | 9926 | -1·24 (-1·90, -0·59)** | 9923 | -1·26 (-1·88, -0·65)** | 9718 | -1·41 (-2·05, -0·77)** | 1188 | 2·11 (0·39, 3·83)* |
| Cohorts | ALSPAC, BIB, CHOP, DNBC, EDEN, ELFE, GENR, INMA | | ALSPAC, BIB, CHOP, DNBC, EDEN, ELFE, GENR, INMA | | ALSPAC, CHOP, DNBC, EDEN, ELFE, GENR, INMA | | ALSPAC, CHOP, EDEN, GENR, INMA | |
| ADHD: Attention-deficit hyperactivity disorder, Non-verbal IQ: Non Verbal Intelligence, CI: Confidence interval, SDS: standard deviation score, **P* value <0·05 ***P* value <0·01. Childhood measurements are between 4-10 years old. Values are regression coefficients (95% confidence interval) obtained from one stage meta-analysis and reflect the differences in childhood internalizing problems (percentiles), externalizing problems (percentiles), ADHD symptoms (percentiles) and Non-verbal IQ score (percentiles) for size for gestational age at birth in categories from pooled multiple imputed data. The basic model is adjusted for sex, age at outcome measurement and cohort effect. | | | | | | | | |

**Table S7. Growth patterns and emotional, behaviour and cognitive outcomes in childhood, basic model**

|  | | Internalizing  Problems | | | Externalizing  problems | | | ADHD symptoms | | | Intelligence  score | | |
| --- | --- | --- | --- | --- | --- | --- | --- | --- | --- | --- | --- | --- | --- |
|  | | N | Difference in percentiles (95 CI) | | N | Difference in percentiles (95 CI) | | N | Difference in percentiles (95 CI) | | N | Difference in percentiles (95 CI) | |
| Small for gestational age | |  |  | |  |  | |  |  | |  |  | |
| 1^st^ tertile | | 564 | 0·40 (-2·12, 2·92) | | 564 | 0·48 (-1·92, 2·87) | | 453 | 3·96 (1·23, 6·69)** | | 230 | -7·81 (-11·62, -3·99)*** | |
| BMI at 2 years 2^nd^ tertile | | 4894 | -1·02 (-2·01, -0·03)* | | 4895 | -0·91 (-1·85, 0·02) | | 4285 | -0·45 (-1·49, 0·58) | | 1814 | -0·60 (-2·14, 0·94) | |
| 3^rd^ tertile | | 164 | -2·38 (-6·99, 2·22) | | 164 | -1·69 (-6·06, 2·69) | | 144 | -1·52 (-6·29, 3·26) | | 86 | 1·21 (-4·95, 7·37) | |
| Appropriate for gestational age | |  |  | |  |  | |  |  | |  |  | |
| 1^st^ tertile | | 339 | 3·91 (0·68, 7·13)* | | 339 | 2·95 (-0·11, 6·01) | | 266 | 4·96 (1·43, 8·50)** | | 129 | -2·51 (-7·56, 2·55) | |
| BMI at 2 years 2^nd^ tertile | | *20457* | *Reference* | | *20461* | *Reference* | | *18007* | *Reference* | | *10244* | *Reference* | |
| 3^rd^ tertile | | *292* | -3·94 (-7·40, -0·47)* | | 292 | -5·22 (-8·51, -1·93)** | | 256 | -1·09 (-4·69, 2·51) | | 125 | 3·32 (-1·81, 8·44) | |
| Large for gestational age | |  |  | |  |  | |  |  | |  |  | |
| 1^st^ tertile | | 192 | 2·50 (-1·76, 6·76) | | 192 | 4·51 (0·46, 8·55)* | | 144 | 5·99 (1·21, 10·76)* | | 72 | -6·17 (-12·90, 0·55) | |
| BMI at 2 years 2^nd^ tertile | | *4902* | -1·56 (-2·55, -0·58)** | | 4904 | -0·91 (-1·85, 0·02) | | 4231 | -1·53 (-5·56, -0·49)** | | 1823 | 2·05 (0·51, 3·59)** | |
| 3^rd^ tertile | | 523 | -2·71 (-5·32, -0·10)* | | 524 | -2·00 (-4·47, 0·48) | | 447 | -3·05 (-5·80, -0·31)* | | 222 | 3·71 (-0·17, 7·59) | |
| Cohorts | ALSPAC, BIB, EDEN, ELFE, GENR, INMA | | | ALSPAC, BIB, EDEN, ELFE, GENR, INMA | | | ALSPAC, EDEN, ELFE, GENR, INMA | | | ALSPAC, GENR, INMA | | |  |
| ADHD: Attention-deficit hyperactivity disorder, Non-verbal IQ: Non Verbal Intelligence, CI: Confidence interval, SDS: standard deviation score, **P* value <0·05 ***P* value <0·01. Childhood measurements are between 4-10 years old. Values are regression coefficients (95% confidence interval) obtained from one stage meta-analysis and reflect the differences in childhood internalizing problems (percentiles), externalizing problems (percentiles), ADHD symptoms (percentiles) and Non-verbal IQ score (percentiles) for the combinations of size for gestational age with early childhood BMI growth patterns from pooled multiple imputed data. The basic model is adjusted for sex, age at outcome measurement and cohort effect. | | | | | | | | | | | | |  |

**Table S8. Birth characteristics and scores for internalizing problems and externalizing problems, complete-case analyses (n=108758)**

|  | Internalizing problems  Difference in percentiles (95% CI) | | | Externalizing problems  Difference in percentiles (95% CI) | | |
| --- | --- | --- | --- | --- | --- | --- |
|  | **Childhood**  **(n=88323)** | **Early Adolescence**  **(n=54538)** | **Late Adolescence**  **(n=47522)** | **Childhood**  **(n=88293)** | **Early Adolescence**  **(n=54540)** | **Late Adolescence**  **(n=47523)** |
| Gestational age  at birth, weeks | -0·49 (-0·64, -0·35)**  N = 65369 | -0·25 (-0·44, -0·06)*  N = 39389 | -0·13 (-0·31, 0·05)  N = 32812 | -0·38 (-0·51, -0·24)**  N = 65359 | -0·30 (-0·49, -0·12)**  N = 39390 | 0·19 (0·00, 0·38)  N = 32812 |
| Gestational age in categories, weeks |  |  |  |  |  |  |
| < 37 | 3·63 (2·45, 4·81)** | 2·27 (0·68, 3·87)** | 1·05 (-0·49, 2·60) | 2·14 (1·03, 3·24)** | 2·33 (0·81, 3·86)** | -2·27 (-3·42, -1·13)** |
|  | N = 2721 | N = 1570 | N = 1321 | N = 2721 | N = 1570 | N = 1321 |
| 37-42 | *Reference* | *Reference* | *Reference* | *Reference* | *Reference* | *Reference* |
|  | N = *59085* | N = *35367* | N = *29508* | N = *59074* | N = *35368* | N = *29508* |
| >42 | 0·50 (-0·54, 1·54) | 0·21 (-1·09, 1·50) | 0·22 (-1·06, 1·49) | -1·03 (-2·00, -0·06)* | -0·92 (-2·16, 0·32) | 0·13 (0·99, 1·25) |
|  | N = 3620 | N = 2417 | N = 1985 | N = 3621 | N = 2417 | N = 1985 |
| Birth weight, | -0·80 (-1·03, -0·57)** | -0·74 (-1.04, -0·44)** | -0·42 (-0·71, -0·13)** | -1·17 (-1·39, -0·96)** | -1·37 (-1·66, -1·08)** | 0·02 (-0·28, 0·32) |
| per 500 gr | N = 65426 | N = 39393 | N = 32814 | N = 65416 | N = 39393 | N = 32814 |
| Birth weight in categories, grams |  |  |  |  |  |  |
| < 2500 | 3·15 (1·80, 4·50)** | 2·43 (0·44, 4·41)* | 0·76 (-1·17, 2·69) | 3·55 (2·29, 4·81)** | 3·37 (1·48, 5·27)** | -2·14 (-4·13, -0·14)** |
|  | N = 2076 | N = 1001 | N = 834 | N = 2075 | N = 1001 | N = 834 |
| 2500-4500 | *Reference* | *Reference* | *Reference* | *Reference* | *Reference* | *Reference* |
|  | N = *61301* | N = *37930* | N = *30696* | N = *61293* | N = *36596* | N = *30696* |
| > 4500 | 0·17 (-1·19, 1·54) | -0·69 (-2·35, 0·98) | 1·17 (-0·41, 2·74) | -1·33 (-2·60, -0·05)* | -1·87 (-3·45, -0·28)* | -0·21 (-1·84, 1·42) |
|  | N = 2049 | N = 1463 | N = 1284 | N = 2048 | N = 1463 | N = 1284 |
| Cohorts | ALSPAC, BIB, DNBC, EDEN, ELFE, GENR, INMA | ALSPAC, DNBC, GENR‡, INMA‡ | ALSPAC, DNBC | ALSPAC, BIB, DNBC, EDEN, ELFE, GENR‡, INMA‡ | ALSPAC, DNBC, GENR‡, INMA‡ | ALSPAC, DNBC |

**CI: Confidence interval, SDS: standard deviation score, **P* value <0·05 ***P* value <0·01. Childhood measurements are between 4-10 years old, early adolescence measurements are between 11-16 years old and adolescence between 17-20 years old. Values are regression coefficients (95% confidence interval) obtained from one stage meta-analysis and reflect the differences in internalizing problems (percentiles) or externalizing problems (percentiles) for birth characteristics from pooled multiple imputed data. ‡: GenerationR and INMA are not included in the analyses of birth weight categories and internalizing or externalizing problems in early adolescence due to a small number of participants and DataSHIELD data security protections. ‡GenerationR was due to the same issue not included in the analysis of gestational age and sex adjusted birth weight categories with internalizing or externalizing problems in early adolescence. The confounder model is adjusted for sex, age at outcome measurement, cohort effect, maternal age at birth, maternal educational level, parity, pre-pregnancy body mass index and smoking during pregnancy.**

**Table S9. Birth characteristics and scores for ADHD symptoms, ASD traits and Non-verbal Intelligence scores, complete-case analyses (n=108758)**

|  | | ADHD symptoms  Difference in percentiles (95% CI) | | | | | | ASD traits  Difference in percentiles (95% CI) | | Non-verbal IQ  Difference in percentiles (95% CI) |
| --- | --- | --- | --- | --- | --- | --- | --- | --- | --- | --- |
|  | | **Childhood**  **(n=84179)** | | **Early Adolescence**  **(n=54657)** | | **Late Adolescence**  **(n=47522)** | | **Childhood**  **(n=6420)** | | **Childhood**  **(n=15941)** |
| Gestational age at birth, weeks | -0·45 (-0·59, -0·31)**  N = 63234 | | -0·27 (-0·46, -0·08)**  N = 39527 | | 0·12 (-0·07, 0·32)  N = 32813 | | -0·35 (-0·79, 0·09)  N = 4926 | | 0·70 (0·41, 0·99)**  N = 12260 | |
| Gestational age in categories, weeks |  | |  | |  | |  | |  | |
| < 37 | 2·40 (1·24, 3·56)** | | 2·28 (0·73, 3·83)** | | -0·87 (-2·49, 0·75) | | -0·50 (-4·41, 3·41) | | -2·87 (-5·28, -0·45)** | |
|  | N = 2581 | | N = 1580 | | N = 1321 | | N = 186 | | N = 550 | |
| 37-42 | *Reference* | | *Reference* | | *Reference* | | *Reference* | | *Reference* | |
|  | N = *57280* | | N = *35529* | | N = *29509* | | N = *4144* | | N = *10660* | |
| >42 | -1·11 (-2·13, -0·08)* | | -0·75 (-2·02, 0·51) | | -0·26 (-1·59, 1·07) | | 0·77 (-1·14, 3·08) | | 0·45 (-1·36, 2·26) | |
|  | N = 3430 | | N = 2422 | | N = 1985 | | N = 596 | | N = 1052 | |
| Birth weight, | -1·28 (-1·51, -1·06)** | | -1·34 (-1·63, -1·05)** | | 0·01 (-0·30, 0·31) | | -0·79 (-1·49, -0·09)* | | 2·13 (1·65, 2·61)** | |
| per 500 gr | N = 63291 | | N = 39531 | | N = 32815 | | N = 4926 | | N = 12262 | |
| Birth weight in categories, grams |  | |  | |  | |  | |  | |
| < 2500 | 4·37 (3·02, 5·72)** | | 3·13 (1·22, 5·04)** | | -1·95 (-3·97, 0·07) | | 2·11 (-1·28, 5·50) | | -4·53 (-6·90, -2·17)** | |
|  | N = 1901 | | N = 1022 | | N = 834 | | N = 249 | | N = 575 | |
| 2500-4500 | *Reference* | | *Reference* | | *Reference* | | *Reference* | | *Reference* | |
|  | N = *59378* | | N = *37042* | | N = *30697* | | N = *4582* | | N = *11491* | |
| > 4500 | -1·11 (-2·44, -0·21)* | | -1·61 (-3·22, 0·01) | | -0·33 (-1·98, 1·32) | | 5·74 (0·33, 11·15)* | | 4·77 (0·78, 8·76)** | |
|  | N = 2012 | | N = 1467 | | N = 1284 | | N = 95 | | N = 196 | |
| Cohorts | ALSPAC, DNBC, EDEN, ELFE, GENR, INMA | | ALSPAC, DNBC, INMA | | ALSPAC, DNBC | | GENR, INMA | | ALSPAC, EDEN, GENR, INMA | |
| ADHD: Attention-deficit hyperactivity disorder, ASD: Autism Spectrum Disorder, Non-verbal IQ: Non Verbal Intelligence, CI: Confidence interval, SDS: standard deviation score, **P* value <0·05 ***P* value <0·01. Childhood measurements are between 4-10 years old, early adolescence measurements are between 11-16 years old and adolescence between 17-20 years old. Values are regression coefficients (95% confidence interval) obtained from one stage meta-analysis and reflect the differences in ADHD symptoms (percentiles), ASD traits (percentiles) and Non-verbal IQ score (percentiles) for birth characteristics from pooled multiple imputed data. For birth characteristics from pooled multiple imputed data. The confounder model is adjusted for sex, age at outcome measurement, cohort effect, maternal age at birth, maternal educational level, parity, pre-pregnancy body mass index and smoking during pregnancy. | | | | | | | | | | |

**Table S10. Size for gestational age at birth categories and emotional, behaviour and cognitive outcomes in childhood, complete case analyses (n=108758)**

| Size for gestational age at birth, SD score | Internalizing  Problems | | Externalizing  problems | | ADHD symptoms | | Intelligence  score | |
| --- | --- | --- | --- | --- | --- | --- | --- | --- |
|  | N | Difference in percentiles (95 CI) | N | Difference in percentiles (95 CI) | N | Difference in percentiles (95 CI) | N | Difference in percentiles (95 CI) |
| Small < 5th percentile | 2576 | 1·85 (0·63, 3·07)** | 2574 | 3·45 (2·31, 4·59)** | 2370 | 4·71 (3·49, 5·93)** | 752 | -6·26 (-8·36, -4·17)** |
| Appropriate 5^th^-95^th^ percentile | *55462* | *Reference* | *55454* | *Reference* | *53642* | *Reference* | *10635* | *Reference* |
| Large > 95^th^ percentile | 7388 | -0·29 (-1·05, 0·47) | 7388 | -1·41 (-2·12, -0·70)** | 7279 | -1·23 (-1·96, -0·49)** | 875 | 2·09 (0·11, 4·05)* |
| Cohorts | ALSPAC, BIB, DNBC, EDEN, ELFE, GENR, INMA | | ALSPAC, BIB, DNBC, EDEN, ELFE, GENR, INMA | | ALSPAC, DNBC, EDEN, ELFE, GENR, INMA | | ALSPAC, EDEN, GENR, INMA | |
| ADHD: Attention-deficit hyperactivity disorder, Non-verbal IQ: Non Verbal Intelligence, CI: Confidence interval, SDS: standard deviation score, **P* value <0·05 ***P* value <0·01. Childhood measurements are between 4-10 years old. Values are regression coefficients (95% confidence interval) obtained from one stage meta-analysis and reflect the differences in childhood internalizing problems (percentiles), externalizing problems (percentiles), ADHD symptoms (percentiles) and Non-verbal IQ score (percentiles) for size for gestational age at birth in categories from pooled multiple imputed data. The confounder model is adjusted for sex, age at outcome measurement, cohort effect, maternal age at birth, maternal educational level, parity, pre-pregnancy body mass index and smoking during pregnancy | | | | | | | | |

**Table S11. Growth patterns and emotional, behaviour and cognitive outcomes in childhood, complete-case analyses (n=108758)**

|  | Internalizing  Problems | | | Externalizing  problems | | | ADHD symptoms | | | Intelligence  score | | | |
| --- | --- | --- | --- | --- | --- | --- | --- | --- | --- | --- | --- | --- | --- |
|  | N | | Difference in percentiles (95 CI) | N | | Difference in percentiles (95 CI) | N | | Difference in percentiles (95 CI) | N | | Difference in percentiles (95 CI) | |
| Small for gestational age |  | |  |  | |  |  | |  |  | |  | |
| 1^st^ tertile | 457 | | -1·12 (-3·90, 1·66) | 457 | | -0·09 (-2·72, 2·54) | 400 | | 3·08 (0·20, 5·95)* | 170 | | -7·00 (-11·32, -2·68)** | |
| BMI at 2 years 2^nd^ tertile | 4204 | | -0·91 (-1·97, 0·15) | 4206 | | -0·47 (-1·47, 0·54) | 3868 | | 0·21 (-0·87, 1·28) | 1396 | | -1·25 (-2·96, 0·46) | |
| 3^rd^ tertile | 131 | | 1·49 (-3·62, 6·60) | 131 | | 2·86 (-1·98, 7·69) | 119 | | 3·11 (-2·07, 8·30) | 58 | | 1·95 (-9·24, 5·35) | |
| Appropriate for gestational age |  | |  |  | |  |  | |  |  | |  | |
| 1^st^ tertile | 273 | | 0·84 (-2·73, 4·41) | 273 | | 1·57 (-1·81, 4·94) | 241 | | 3·29 (-0·38, 6·97) | 98 | | -1·40 (-7·04, 4·25) | |
| BMI at 2 years 2^nd^ tertile | *15967* | | *Reference* | *15971* | | *Reference* | *14749* | | *Reference* | *7674* | | *Reference* | |
| 3^rd^ tertile | *243* | | -2·88 (-6·65, 0·89) | 243 | | -3·37 (-6·94, 0·20) | 218 | | -0·45 (-4·30, 3·40) | 90 | | 2·06 (-3·82, 7·93) | |
| Large for gestational age |  | |  |  | |  |  | |  |  | |  | |
| 1^st^ tertile | 152 | | 1·99 (-2·76, 6·74) | 152 | | 3·50 (-1·00, 7·99) | 128 | | 5·53 (0·53, 10·53)* | 54 | | -4·89 (-12·45, 2·68) | |
| BMI at 2 years 2^nd^ tertile | *4195* | | -1·17 (-2·24, -0·11)* | 4197 | | -0·21 (-1·22, 0·80) | 3823 | | -0·99 (-2·08, 0·09) | 1396 | | 1·20 (-0·51, 2·91) | |
| 3^rd^ tertile | 427 | | -1·21 (-4·08, 1·66) | 428 | | -1·06 (-3·77, 1·65) | 387 | | -2·40 (-5·31, 0·52) | 157 | | 3·15 (-1·34, 7·63) | |
| Cohorts | | ALSPAC, BIB, EDEN, ELFE, GENR, INMA | | | ALSPAC, BIB, EDEN, ELFE, GENR, INMA | | | ALSPAC, EDEN, ELFE, GENR, INMA | | | ALSPAC, GENR, INMA | |  |
| ADHD: Attention-deficit hyperactivity disorder, Non-verbal IQ: Non Verbal Intelligence, CI: Confidence interval, SDS: standard deviation score, **P* value <0·05 ***P* value <0·01. Childhood measurements are between 4-10 years old. Values are regression coefficients (95% confidence interval) obtained from one stage meta-analysis and reflect the differences in childhood internalizing problems (percentiles), externalizing problems (percentiles), ADHD symptoms (percentiles) and Non-verbal IQ score (percentiles) for the combinations of size for gestational age with early childhood BMI growth patterns from pooled multiple imputed data. The confounder model is adjusted for sex, age at outcome measurement, cohort effect, maternal age at birth, maternal educational level, parity, pre-pregnancy body mass index and smoking during pregnancy. | | | | | | | | | | | | |  |

**Figure S1. Study Flowchart**

**Children with data on birth weight and at least one neuro outcome available**

n= 109481 (68.2%)

**Children with birth weight available**

n= 156709 (97.6%)

**Children with no data on at least one neuro outcome**

n= 47228 (29.4%) excluded

**No birth weight available**

N= 3799 (2.4%) excluded

**All offspring**

N= 160508

**Figure S2. Cohort specific Flowchart**

**ALSPAC**

**All offspring**

N= 15645

**No birth weight available**

N= 1782 (11.4) excluded

**Children with no data on at least one neuro outcome**

n= 3352 (21.4%) excluded

**Children with data on birth weight and at least one neuro outcome available**

n= 10511 (67.2%)

**Children with birth weight available**

n= 13863 (88.6%)

**BIB**

**All offspring**

N= 13858

**No birth weight available**

N= 334 (2.4%) excluded

**Children with no data on at least one neuro outcome**

n= 11178 (80.7%) excluded

**Children with data on birth weight and at least one neuro outcome available**

n= 2346 (16.9%)

**Children with birth weight available**

n= 13524 (97.6%)

**CHOP**

**CHOP**

**All offspring**

N= 1678

**Children with data on birth weight and at least one neuro outcome available**

n= 723 (43.1%)

**Children with no data on at least one neuro outcome**

n= 950 (56.6%) excluded

**No birth weight available**

N= 5 (0.3%) excluded

**Children with birth weight available**

n= 1673 (99.7%)

**DNBC**

**Children with birth weight available**

n= 142843

**All offspring**

N= 13858

**No birth weight available**

N= 2020 excluded

**Children with no data on at least one neuro outcome**

n= 43873 excluded

**Children with data on birth weight and at least one neuro outcome available**

n= 98970

**Children with birth weight available**

n= 96142 (99.3%)

**All offspring**

N= 96825

**No birth weight available**

N= 683 (0.7%) excluded

**Children with no data on at least one neuro outcome**

n= 21249 (21.9%) excluded

**Children with data on birth weight and at least one neuro outcome available**

n= 74893 (77.3%)

**EDEN**

**EDEN**

**Children with birth weight available**

n= 1899 (94.9%)

**All offspring**

N= 2002

**No birth weight available**

N= 103 (5.1%) excluded

**Children with no data on at least one neuro outcome**

n= 640 (32.0%) excluded

**Children with data on birth weight and at least one neuro outcome available**

n= 1259 (63.9%)

**ELFE**

**Children with birth weight available**

n= 17826 (97.3%)

**All offspring**

N= 18329

**No birth weight available**

N= 503 (2.7%) excluded

**Children with no data on at least one neuro outcome**

n= 6937 (37.8%) excluded

**Children with data on birth weight and at least one neuro outcome available**

n= 10889 (59.4%)

**Generation R**

**Children with birth weight available**

n= 9662 (97.6%)

**All offspring**

N= 9901

**No birth weight available**

N= 239 (2.4%) excluded

**Children with no data on at least one neuro outcome**

n= 2331 (23.5%) excluded

**Children with data on birth weight and at least one neuro outcome available**

n= 7331 (74.0%)

**INMA**

**INMA**

**Children with birth weight available**

n= 2120 (93.4%)

**All offspring**

N= 2270

**No birth weight available**

N= 150 (6.6%) excluded

**Children with no data on at least one neuro outcome**

n= 591 (26.0%) excluded

**Children with data on birth weight and at least one neuro outcome available**

n= 1529 (67.4%)

**Figure S3. Directed Acyclic Graph used to identify potential confounders.**


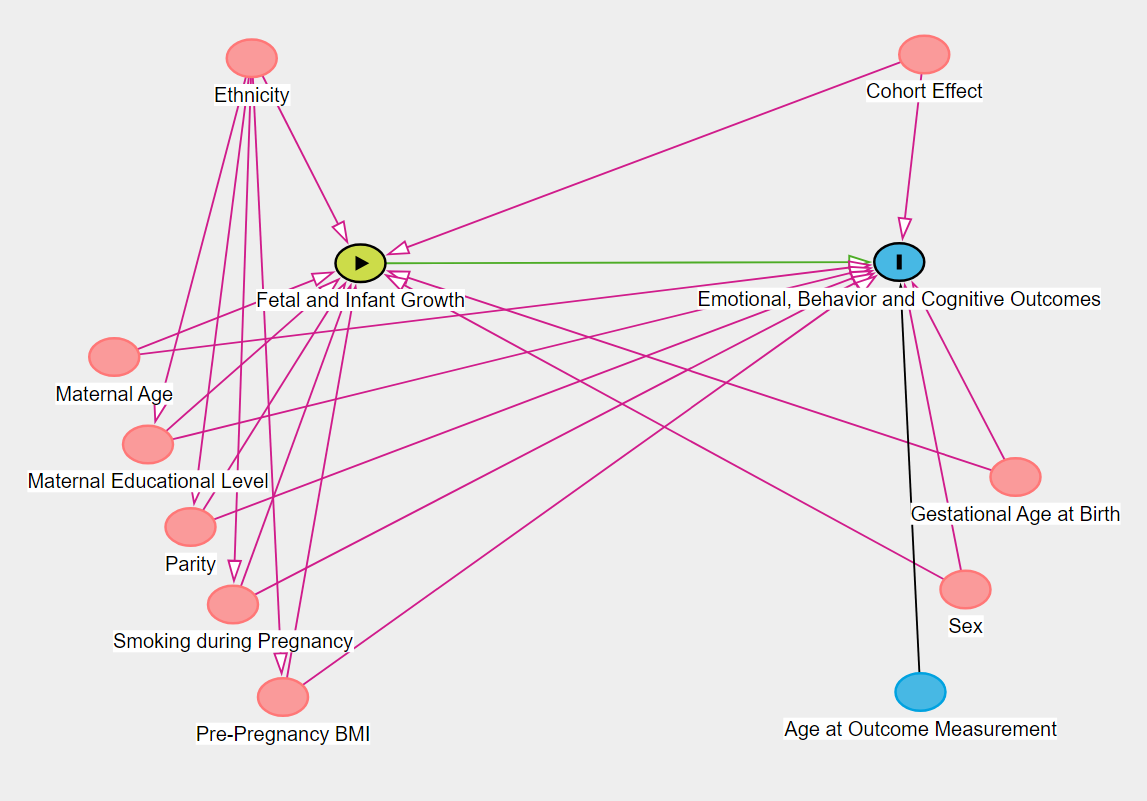


**Figure S4. Sensitivity analyses two-stage individual participant data meta-analysis of birth characteristics and emotional, behaviour and cognitive outcomes.**

**Outcome: Internalizing problems score**
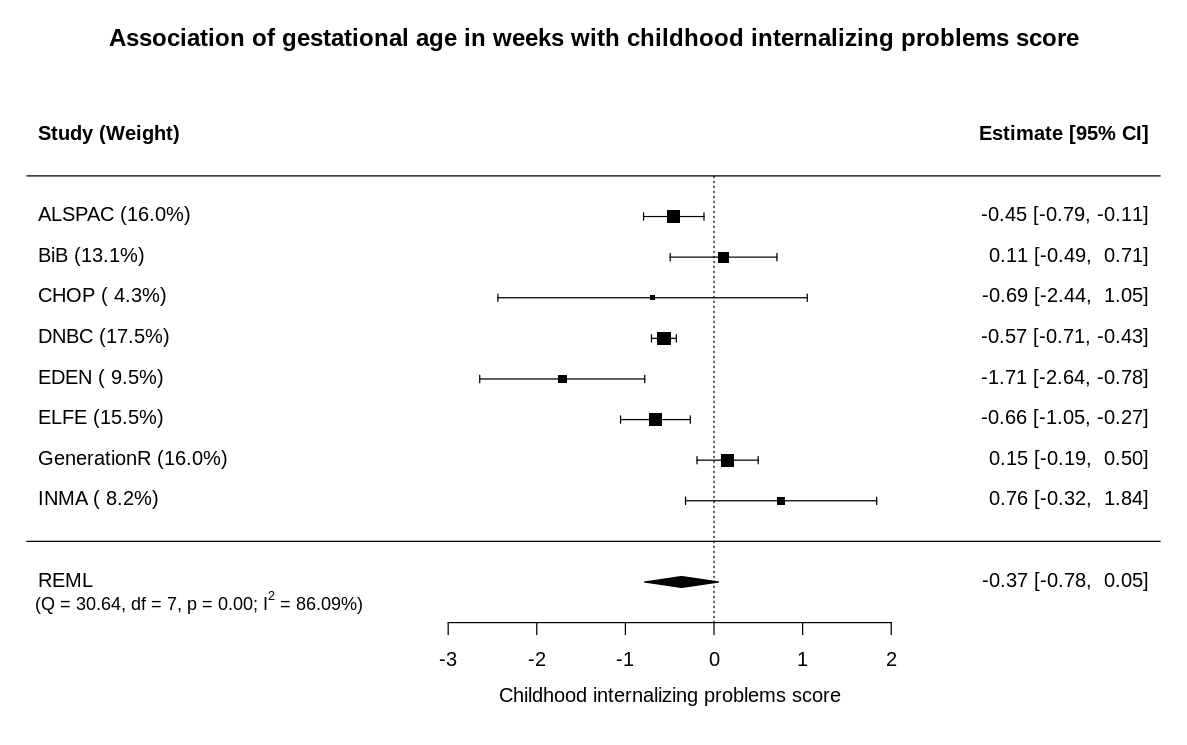

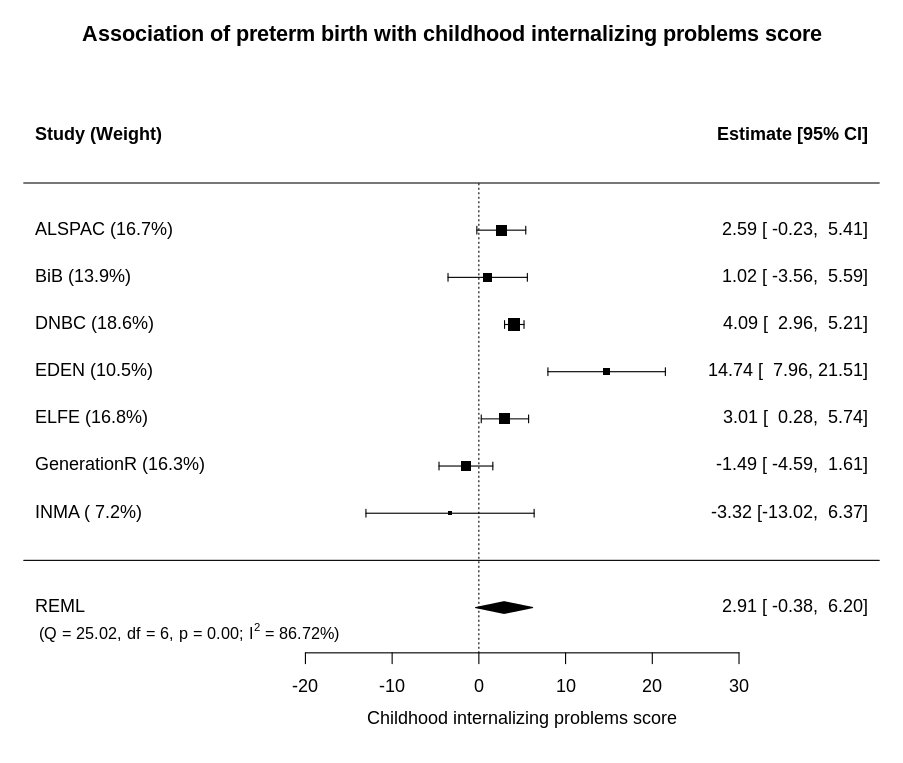

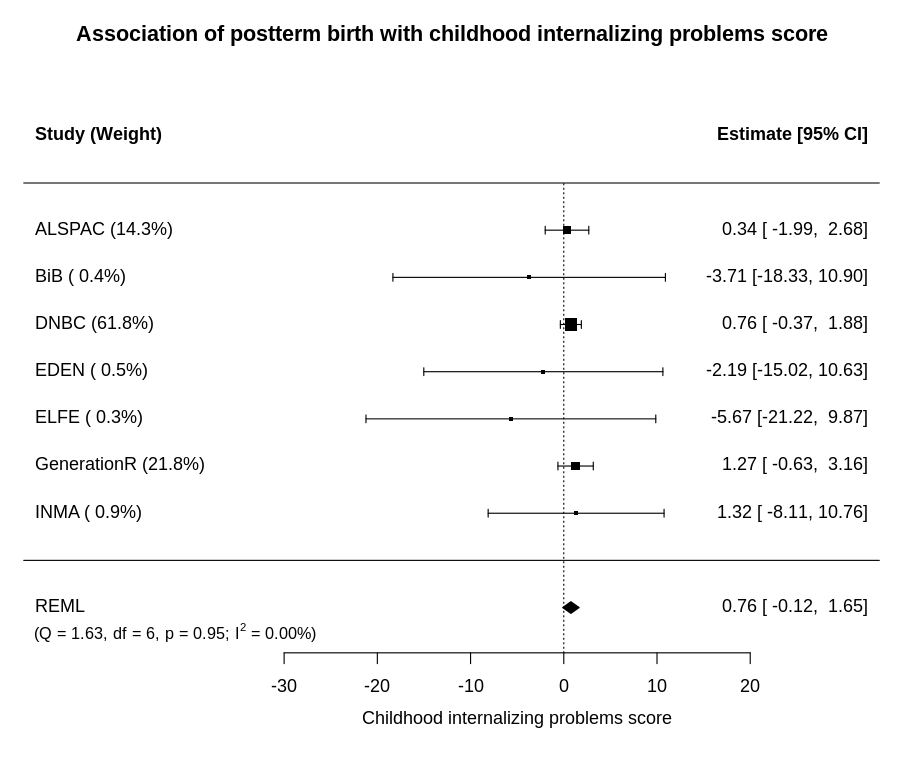


**
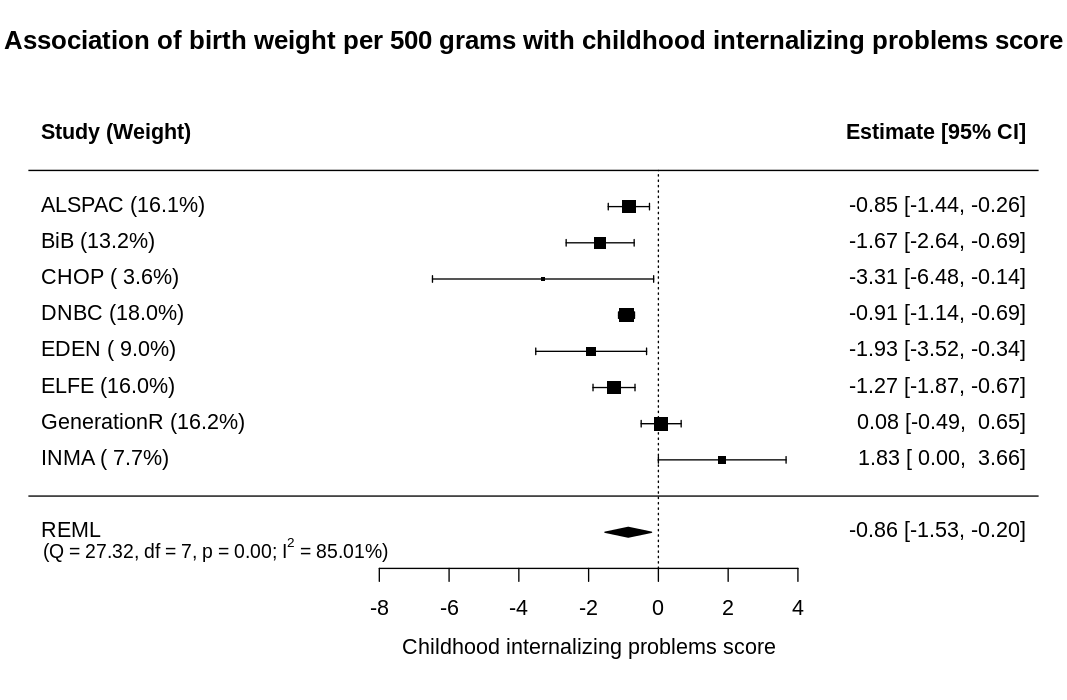
**


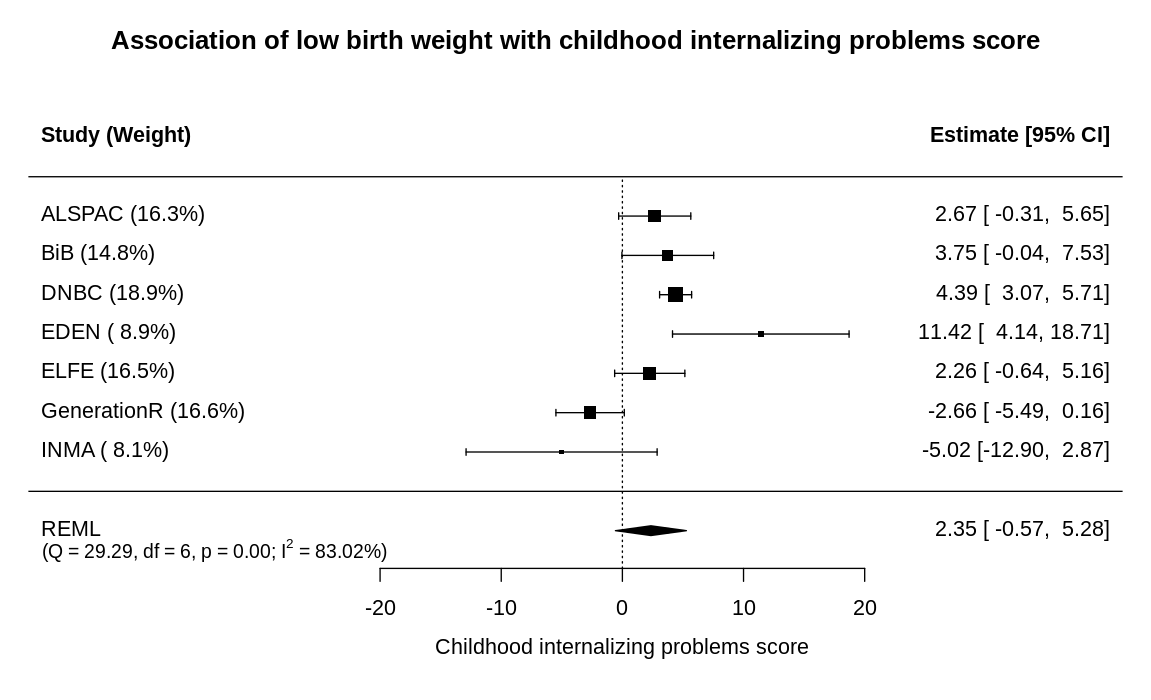


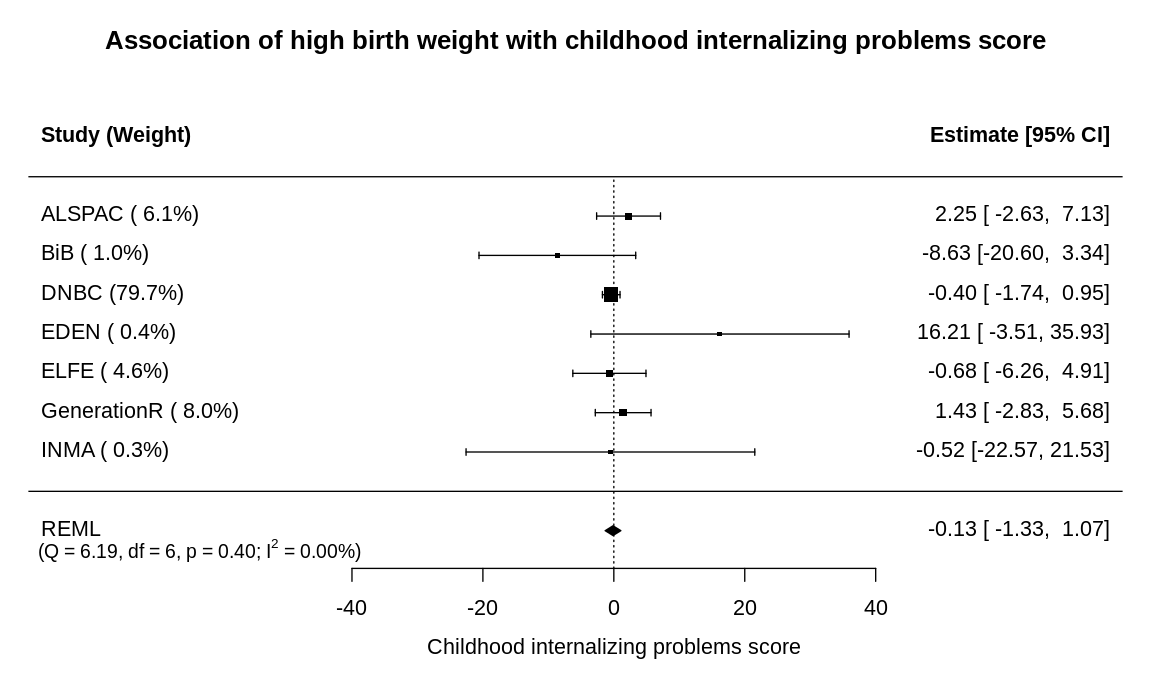


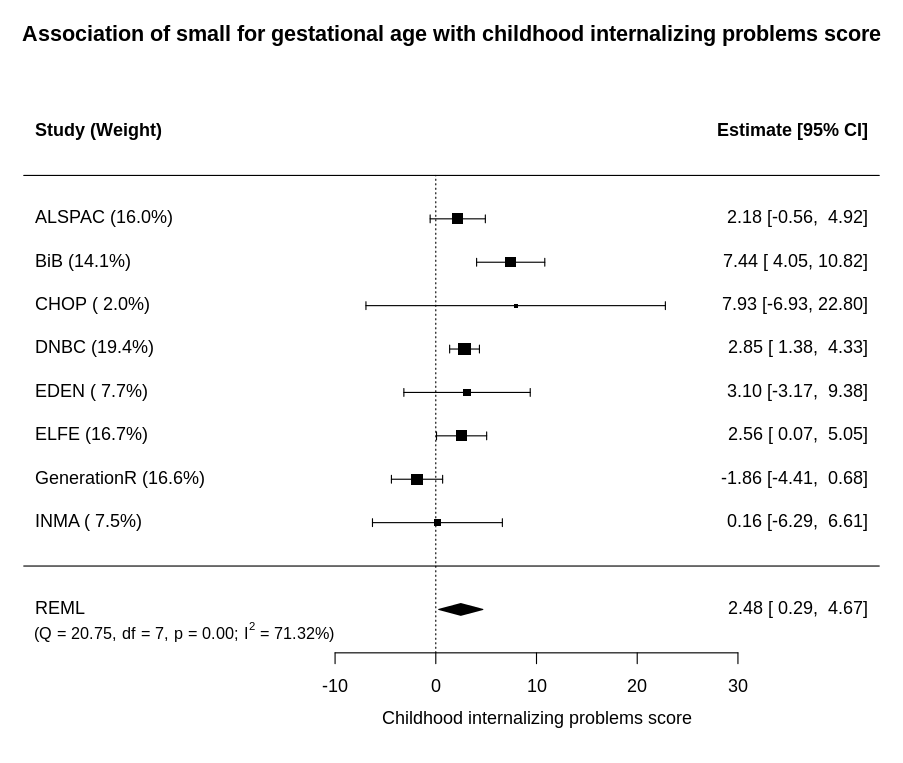


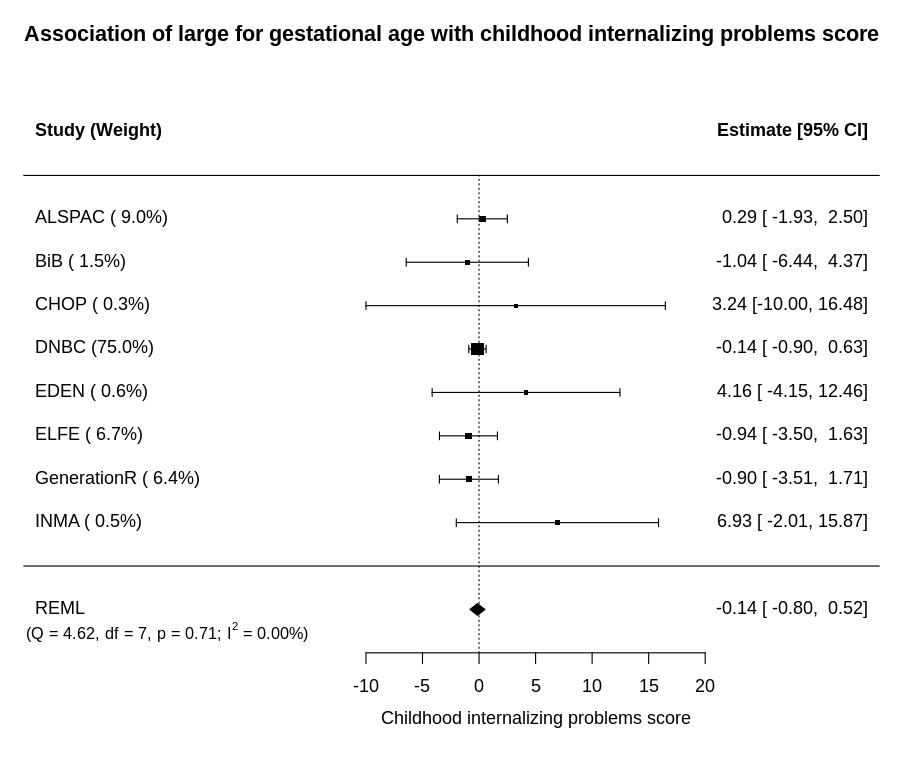


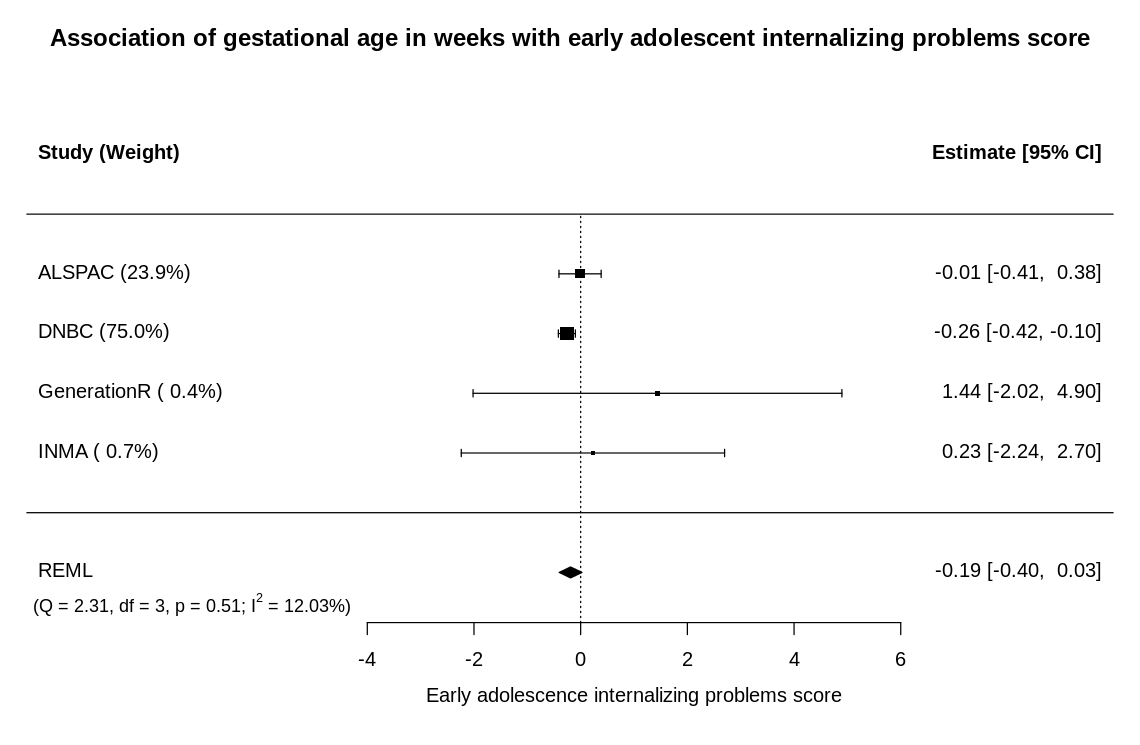


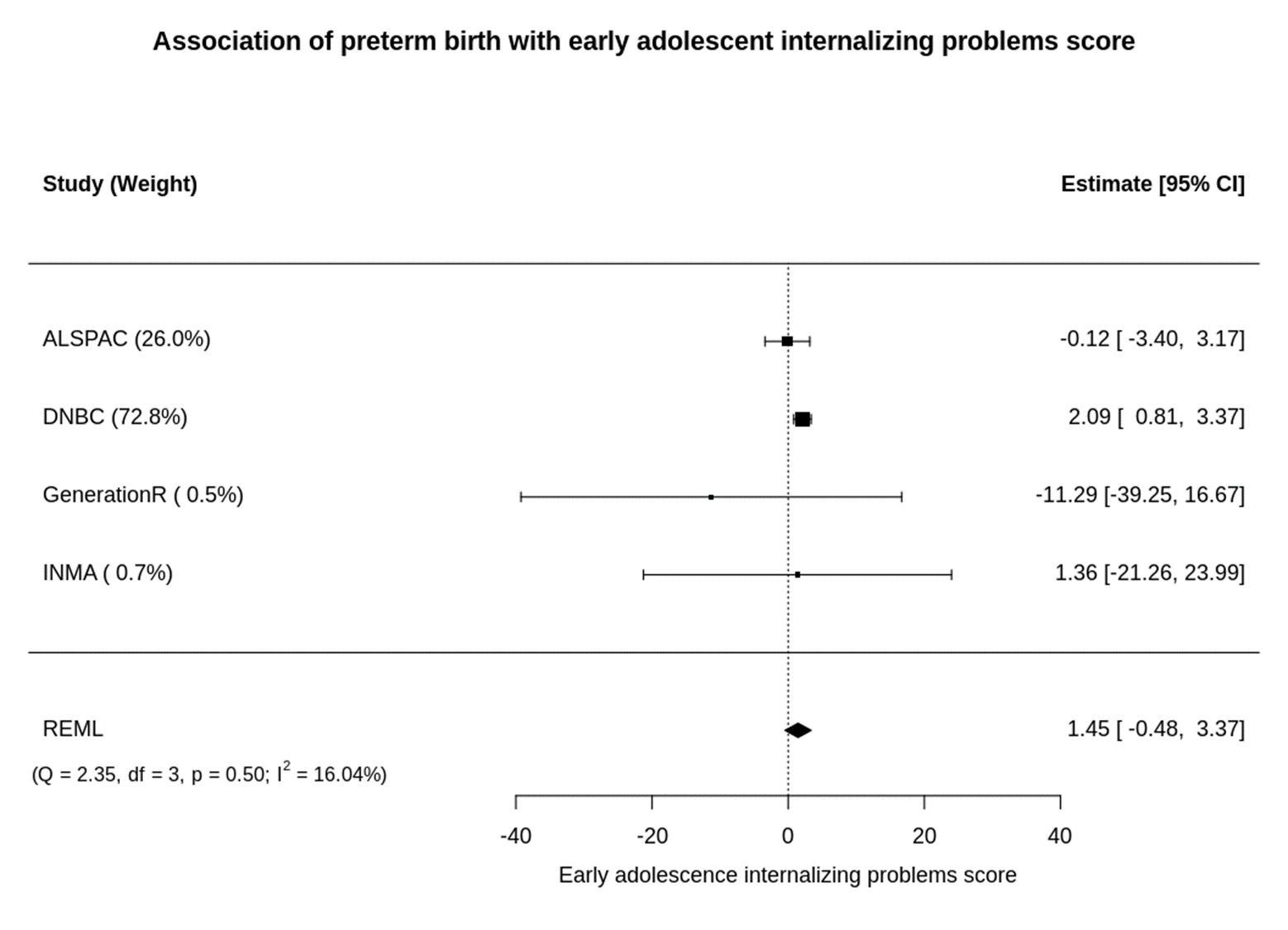


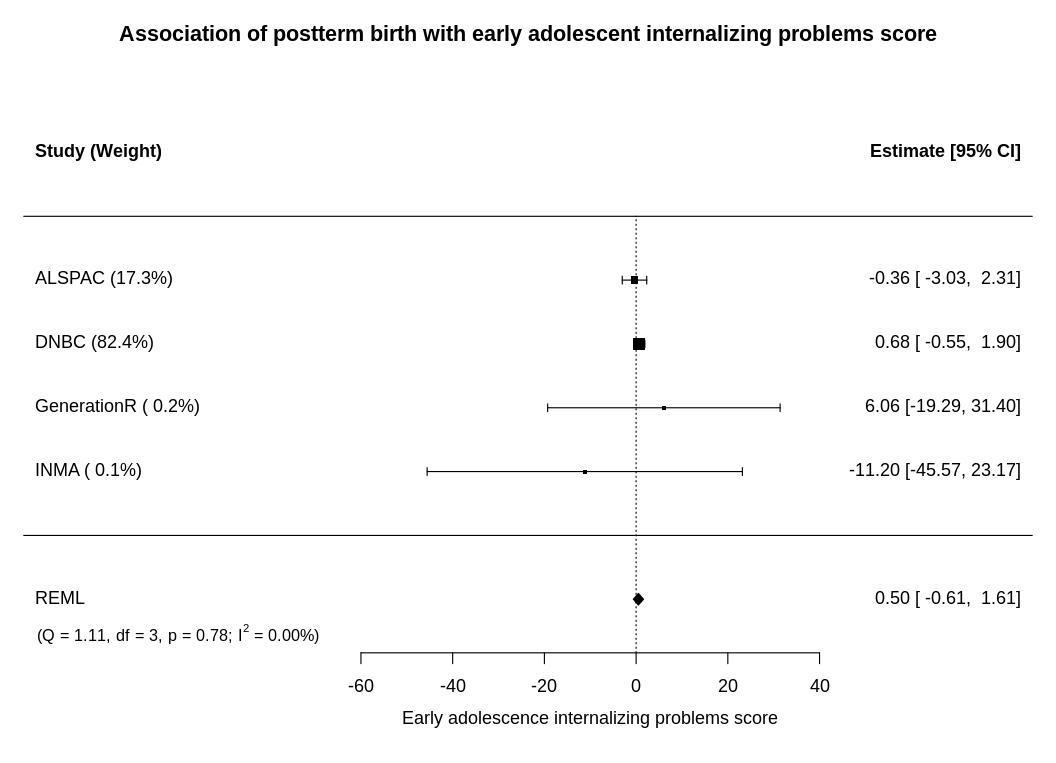


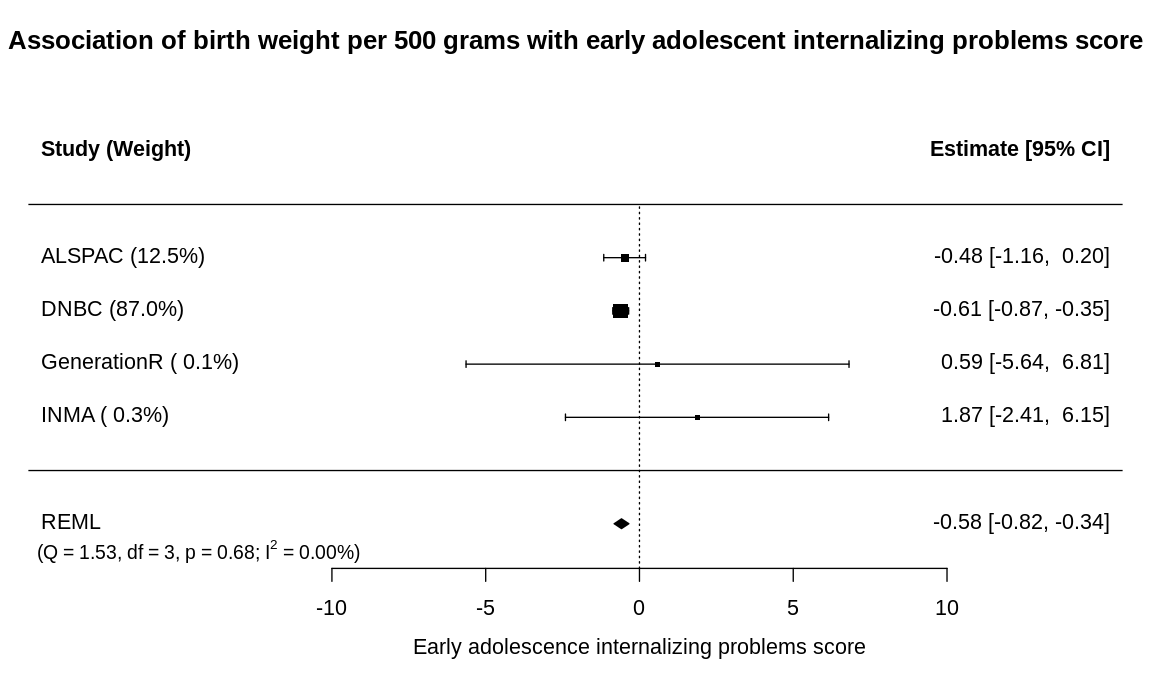

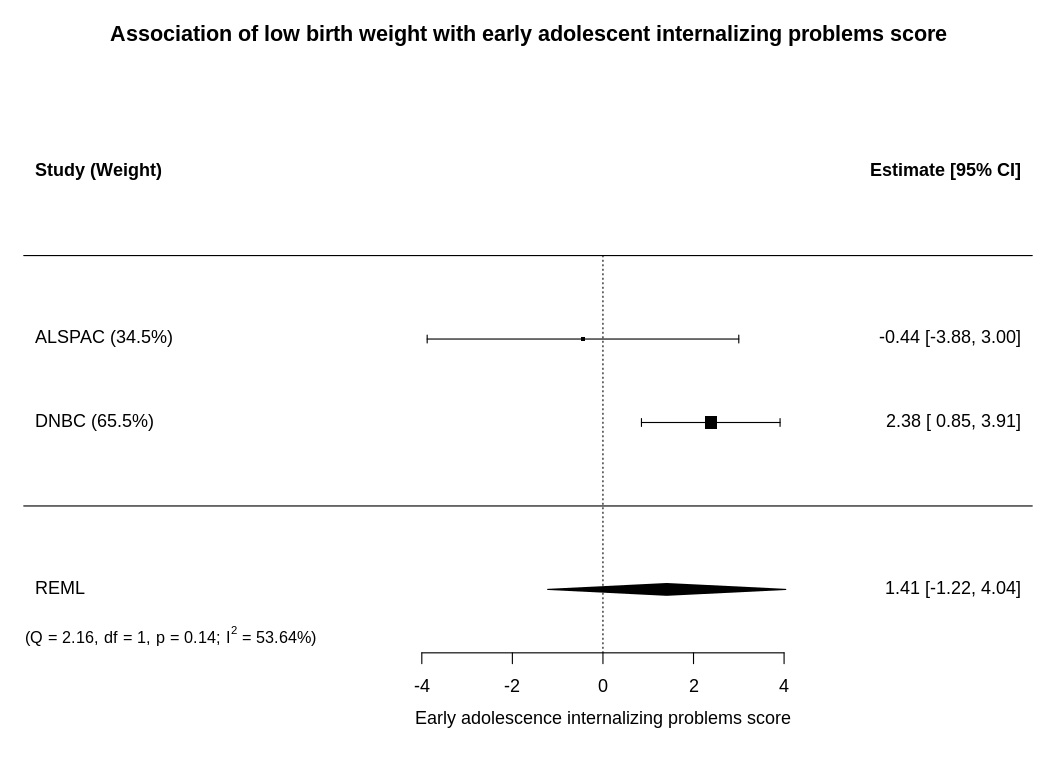


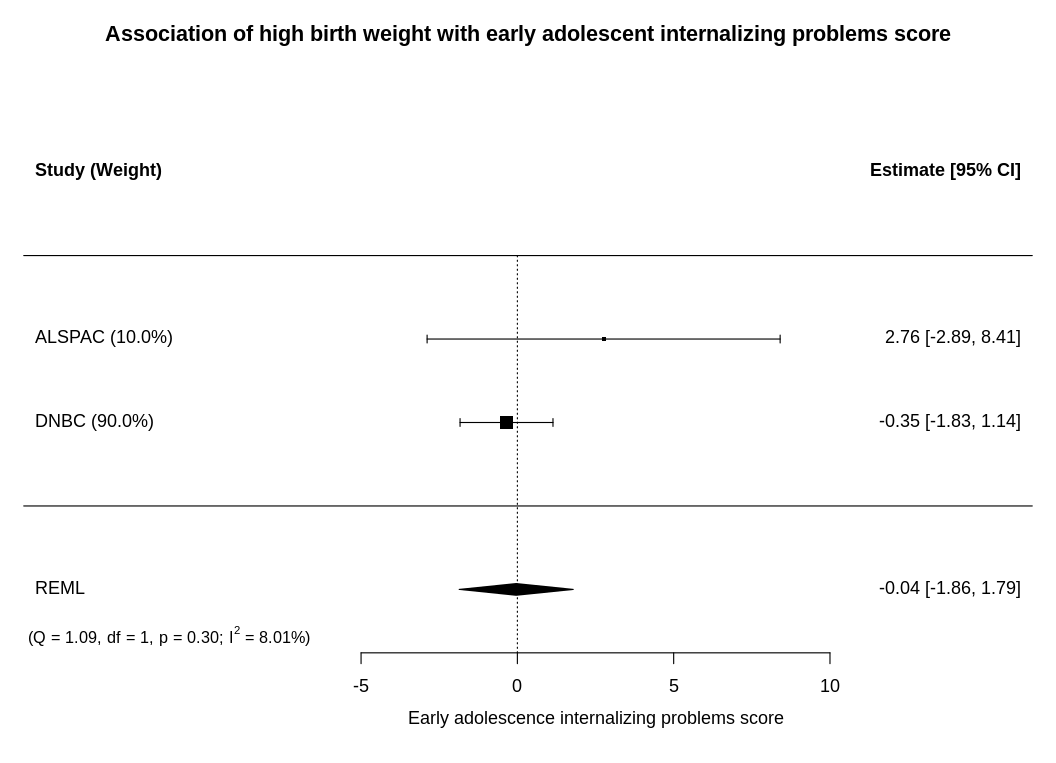


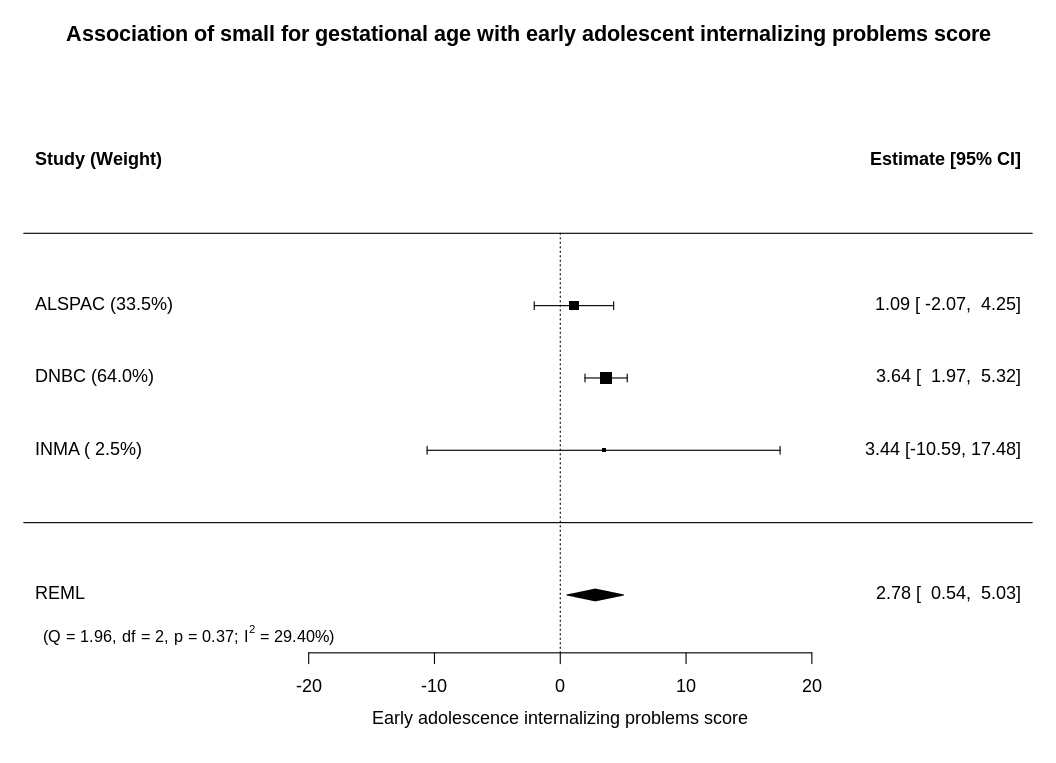


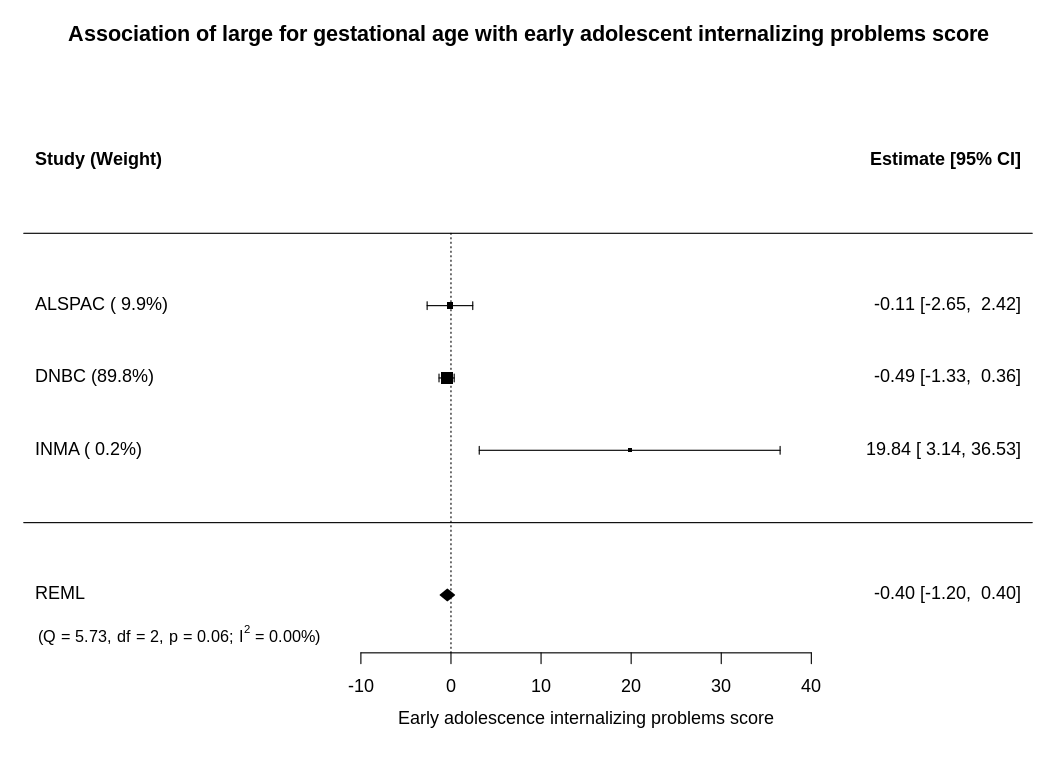


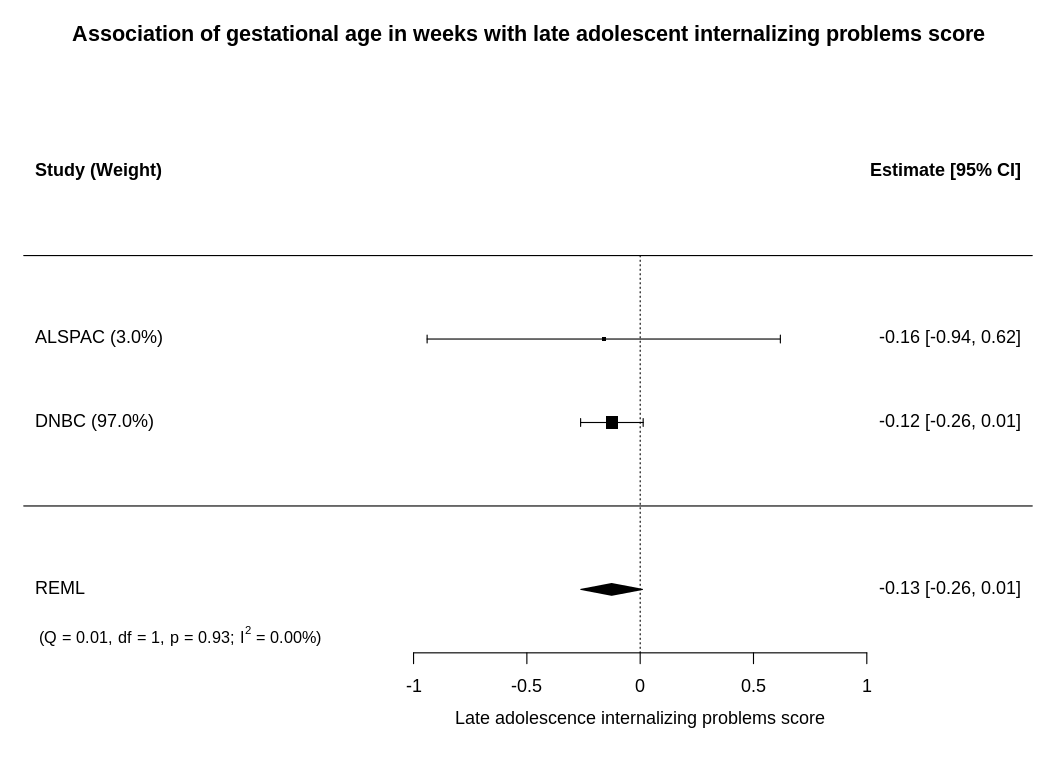


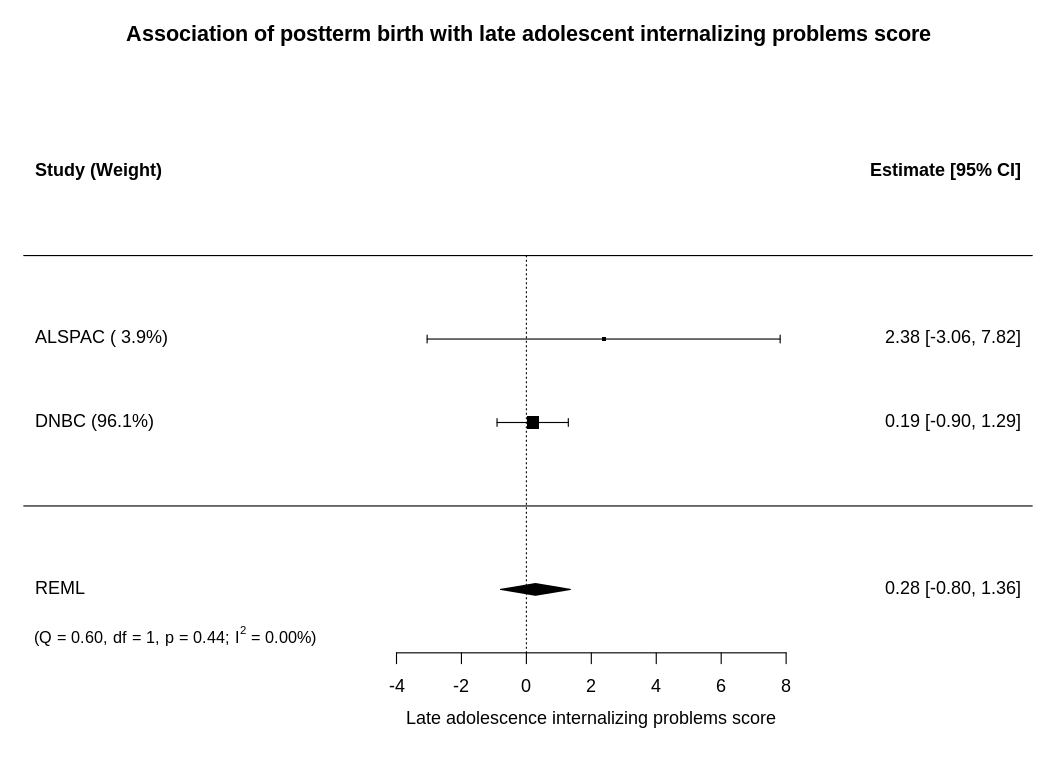

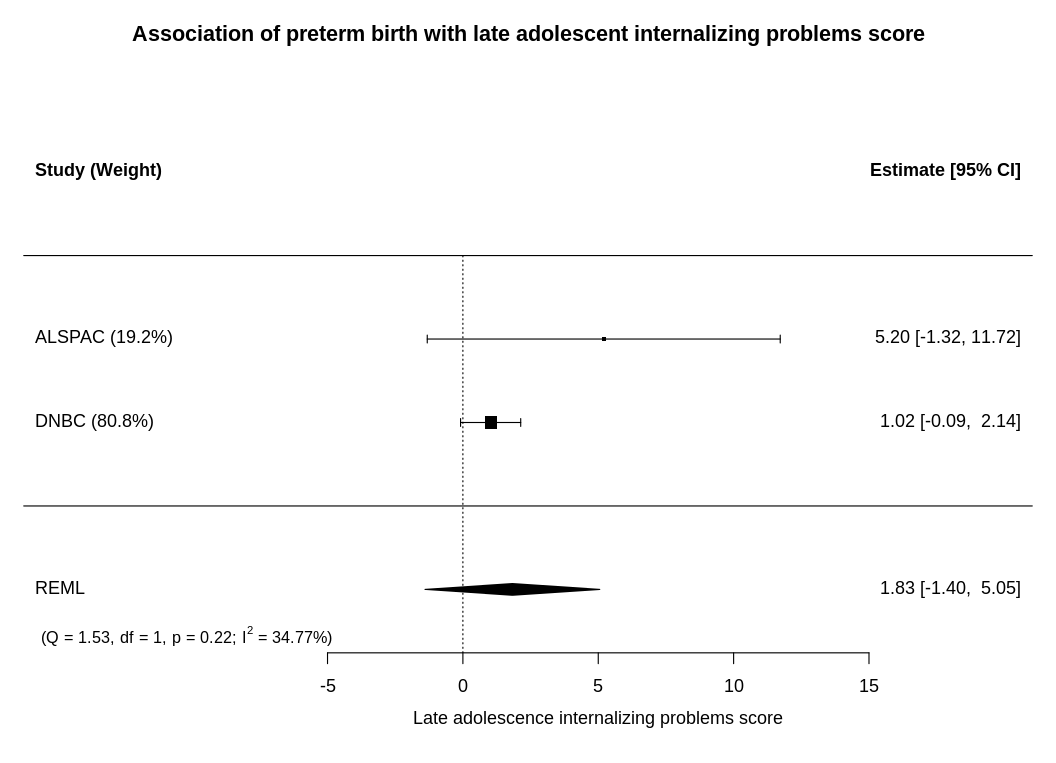


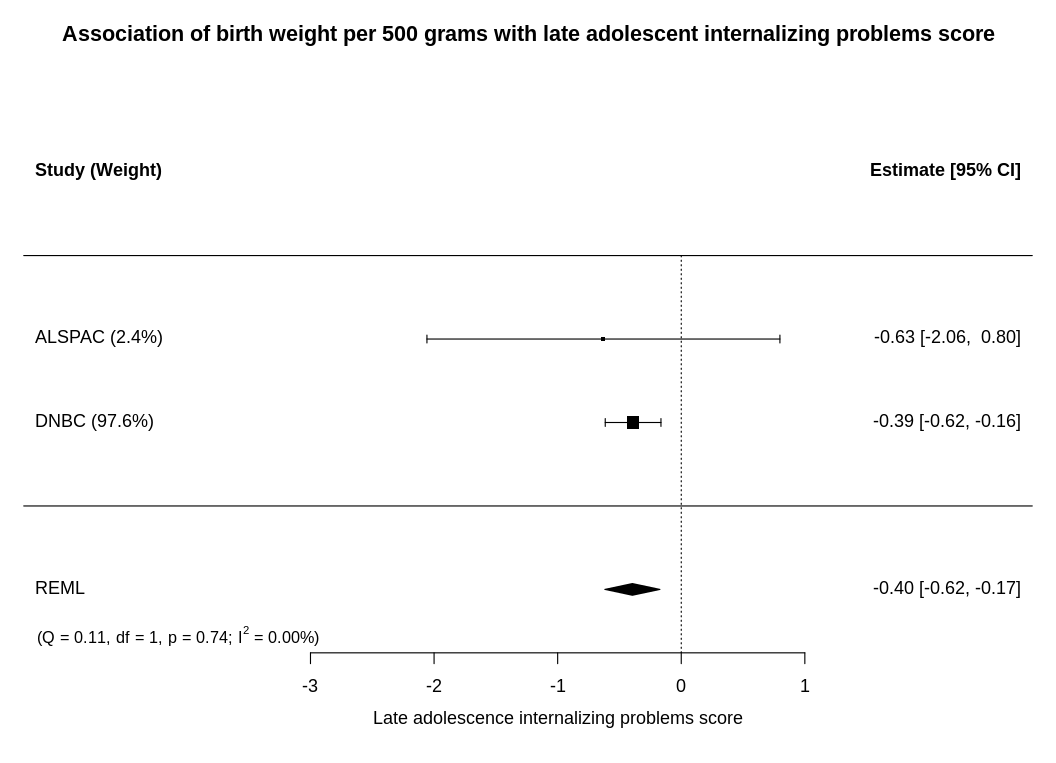


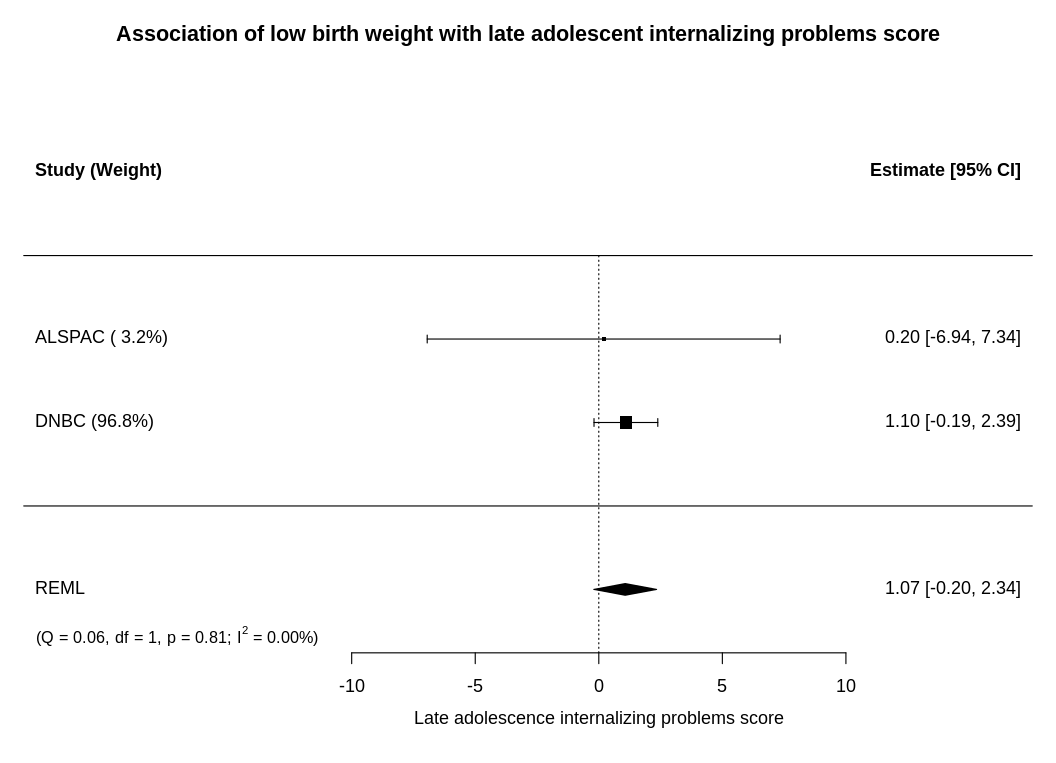


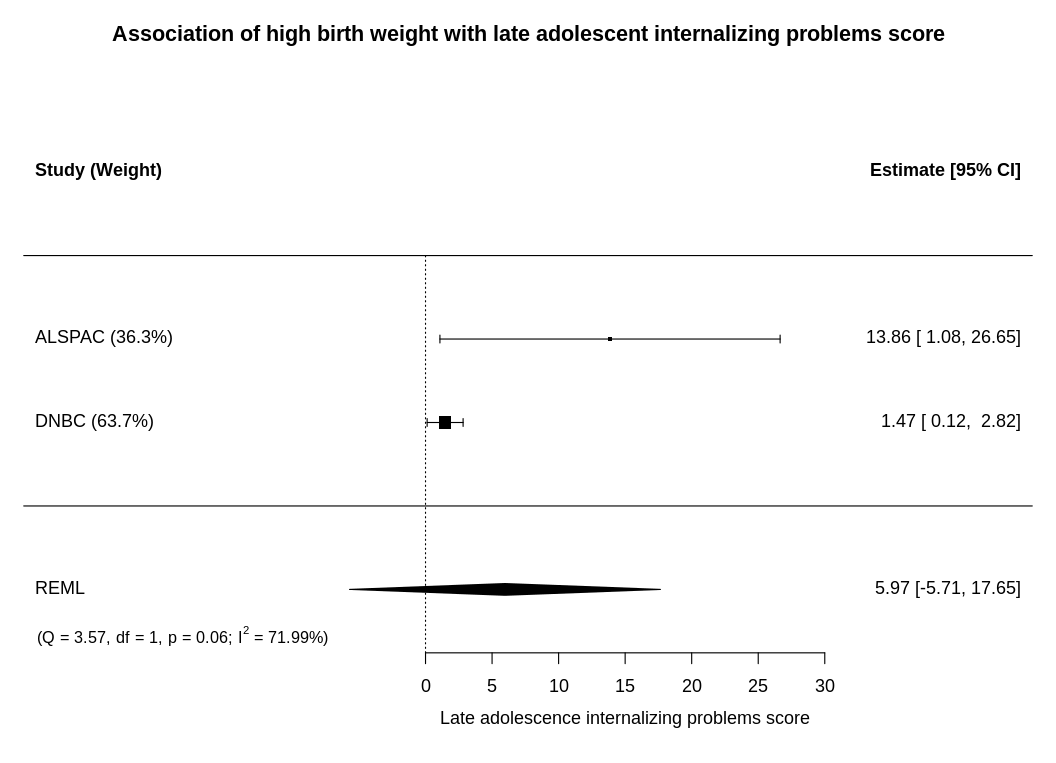


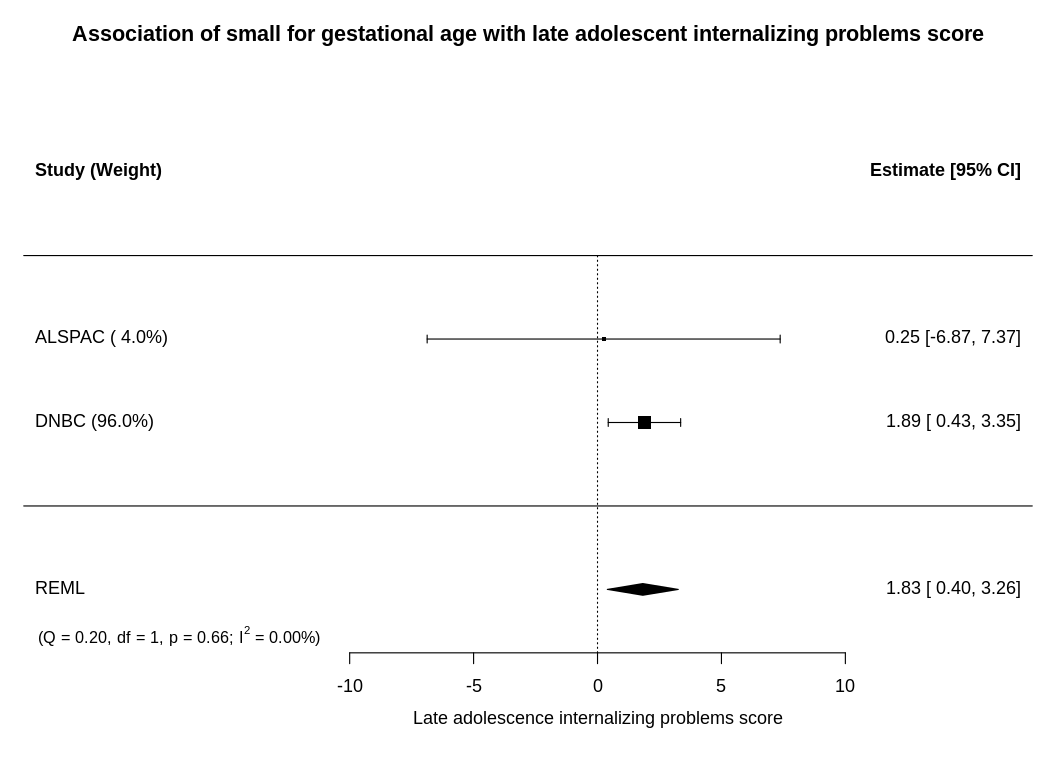


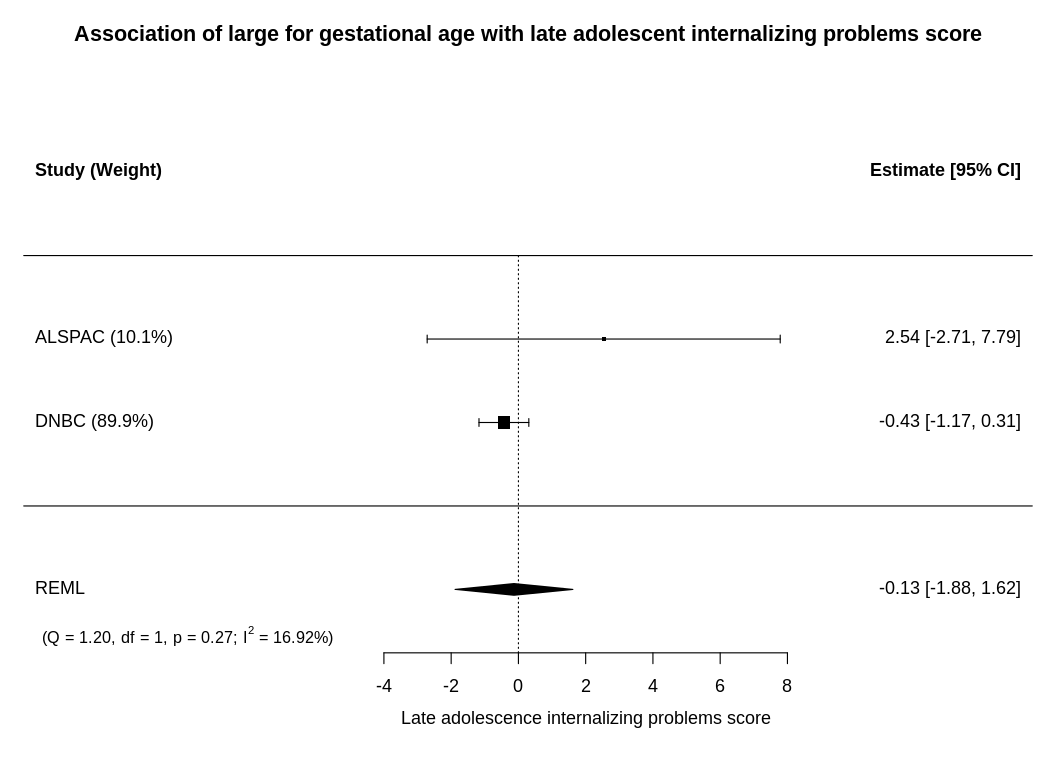


**Outcome: Externalizing problems score**

**
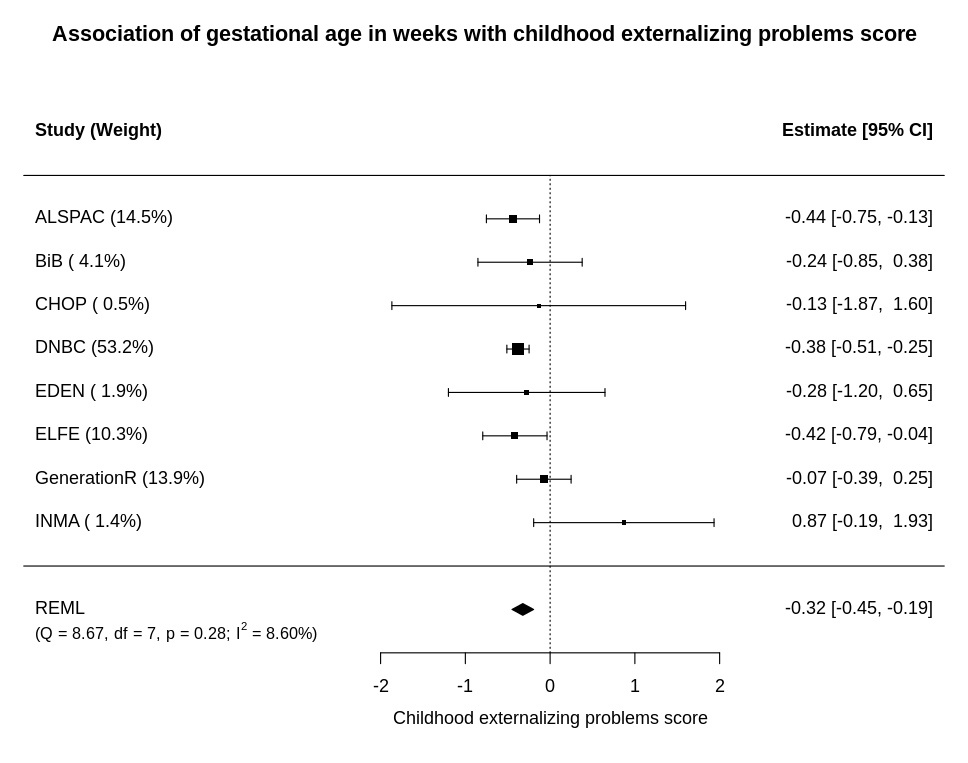
**

**
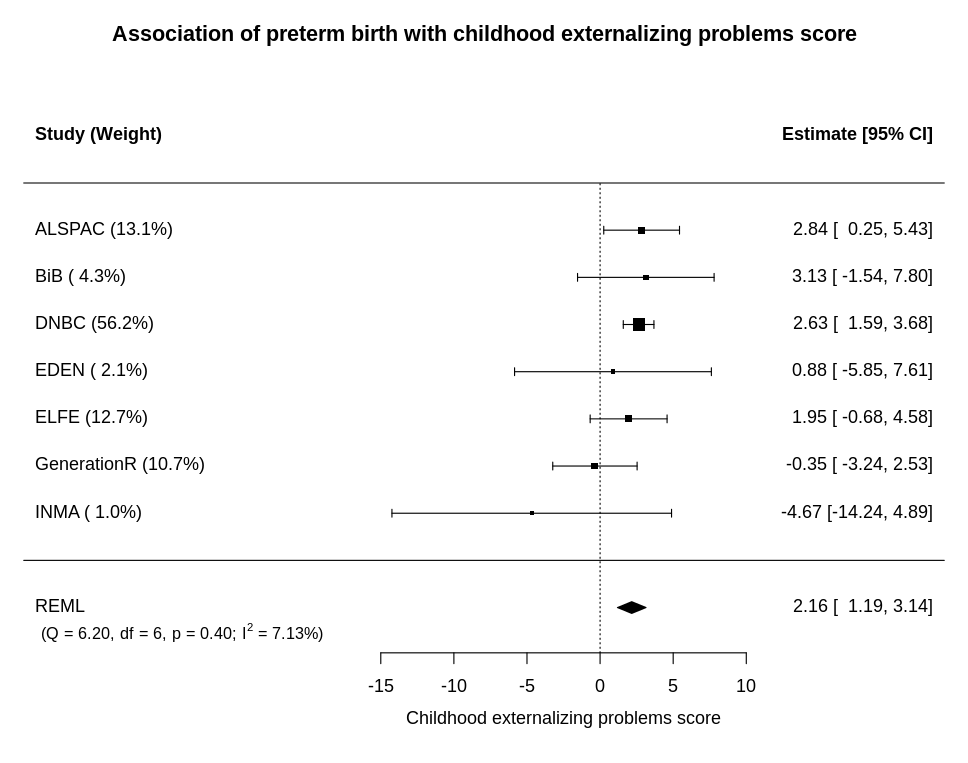
**

**
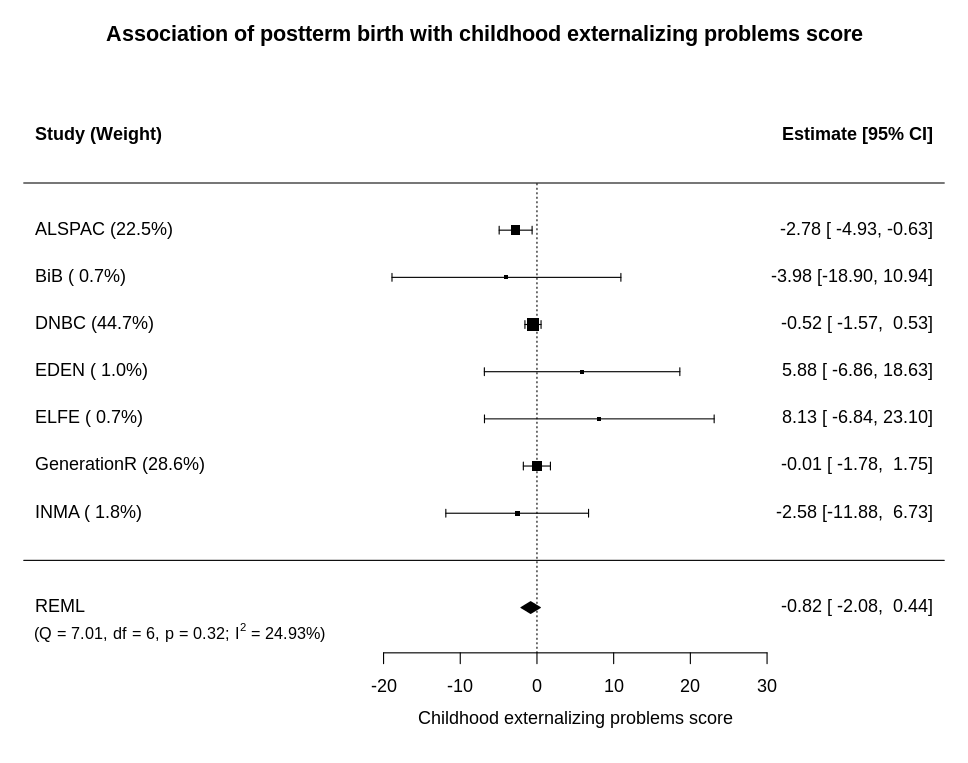
**

**
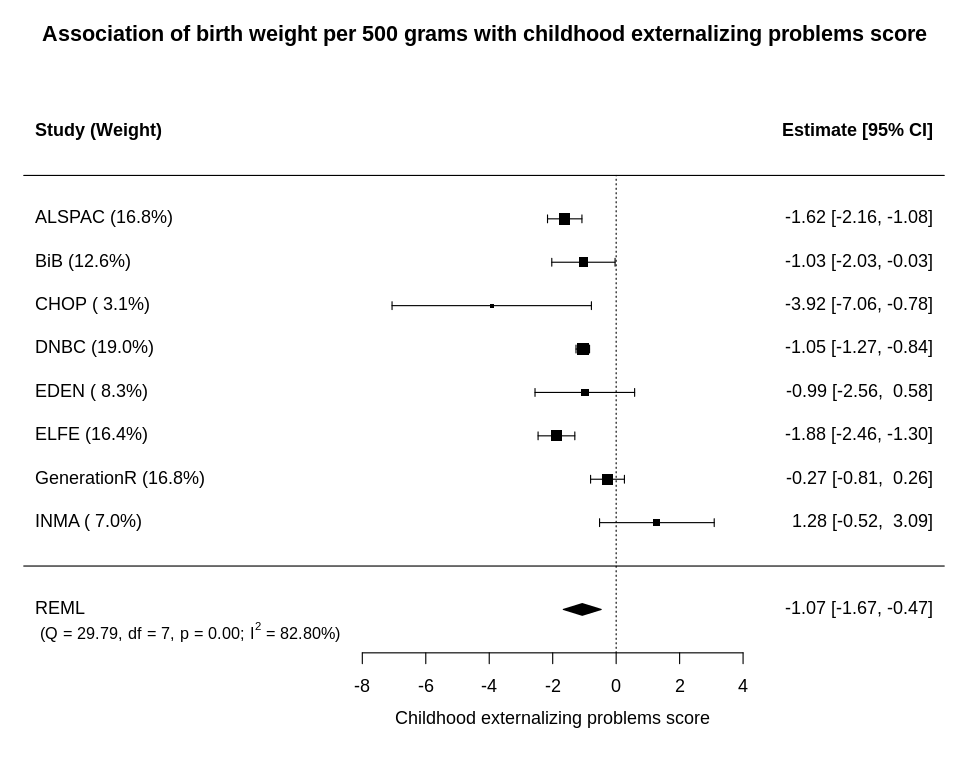
**

**
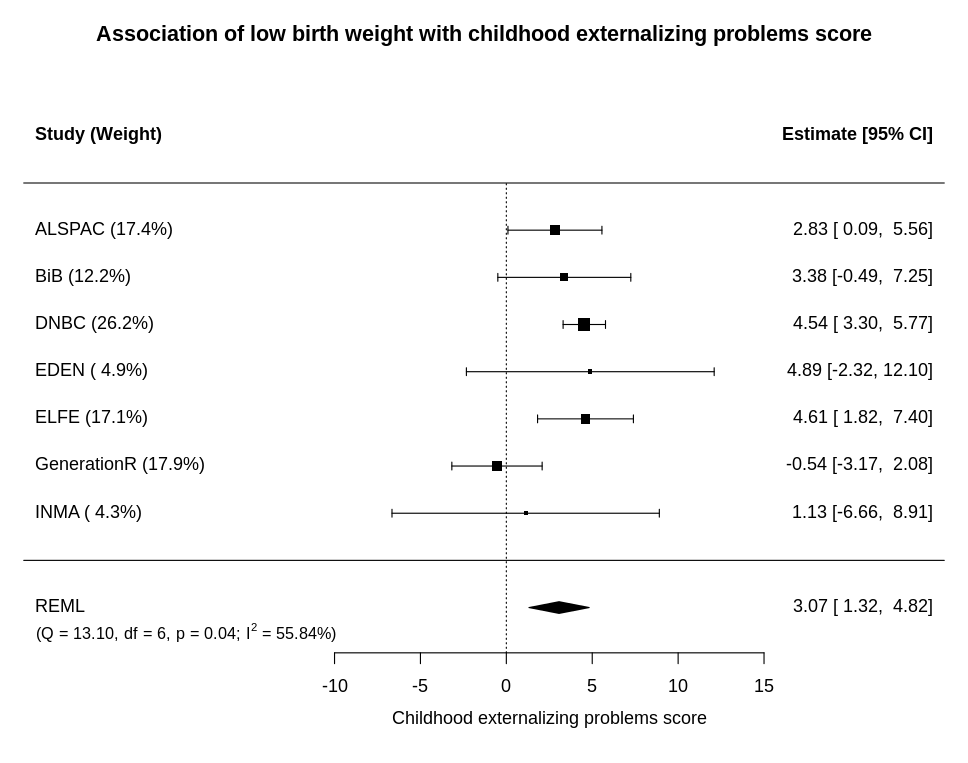
**

**
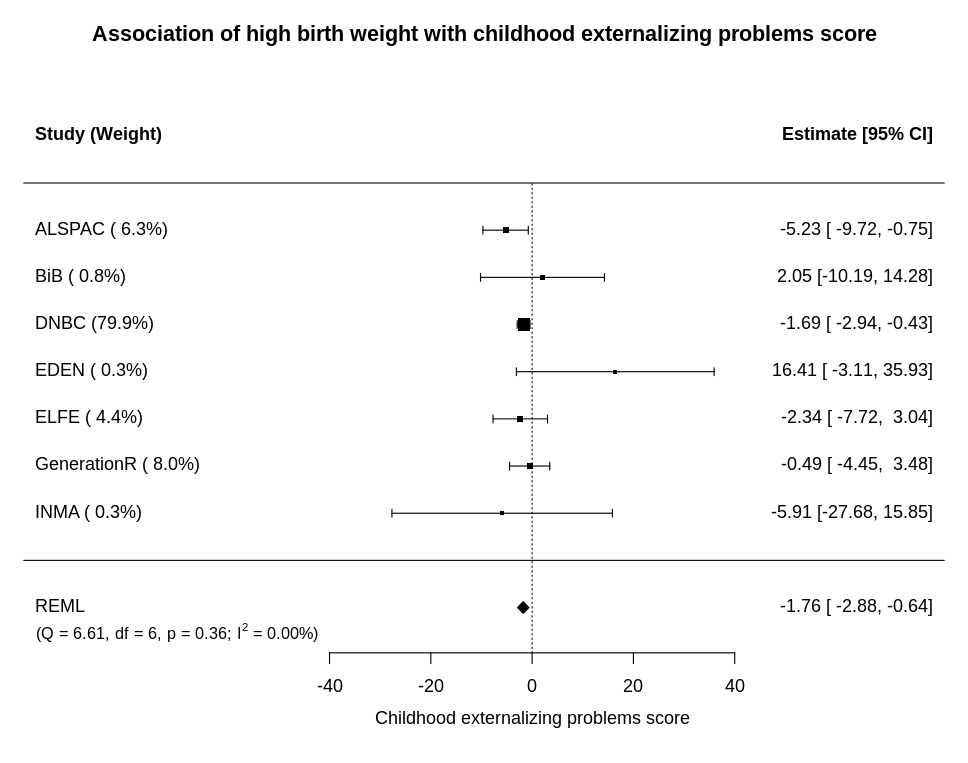
**

**
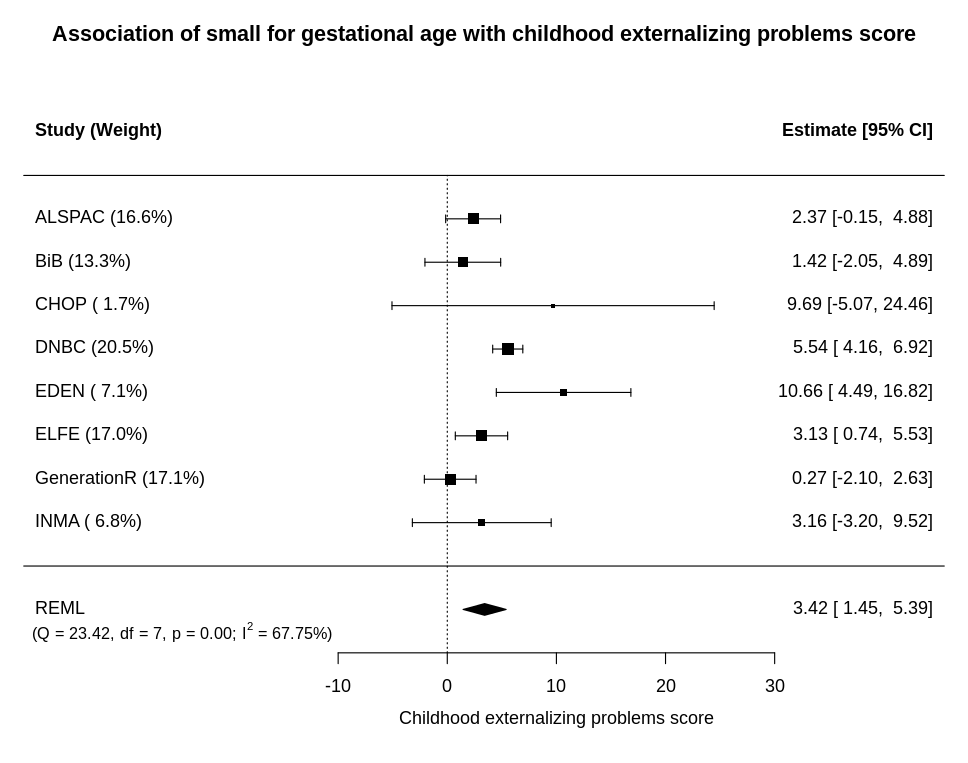
**

**
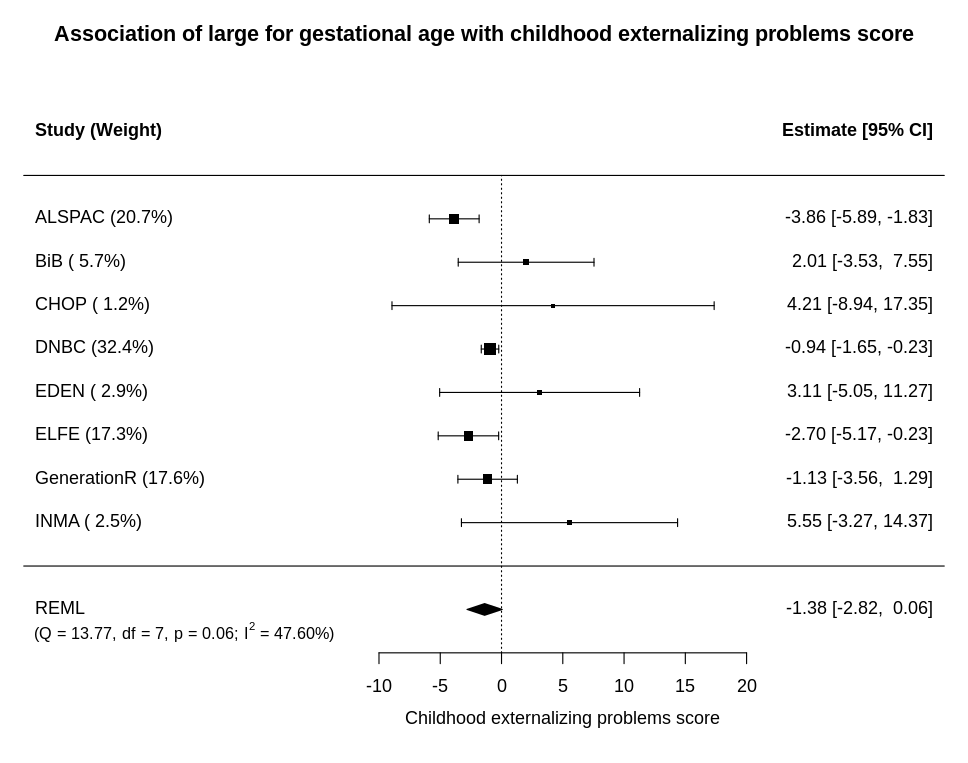
**

**
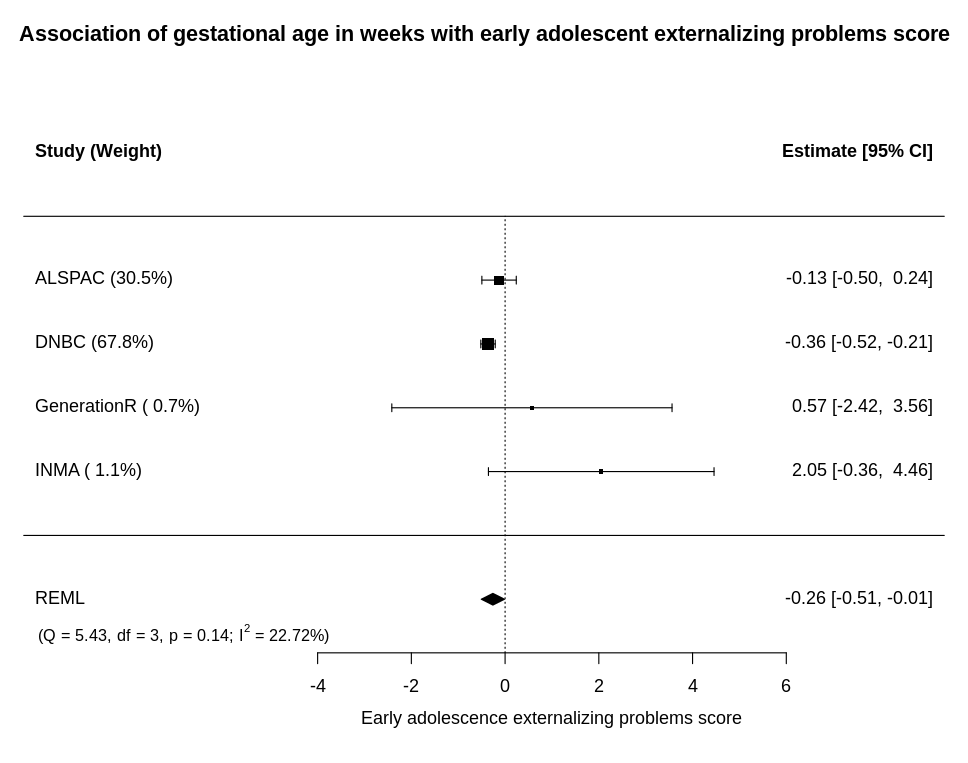
**

**
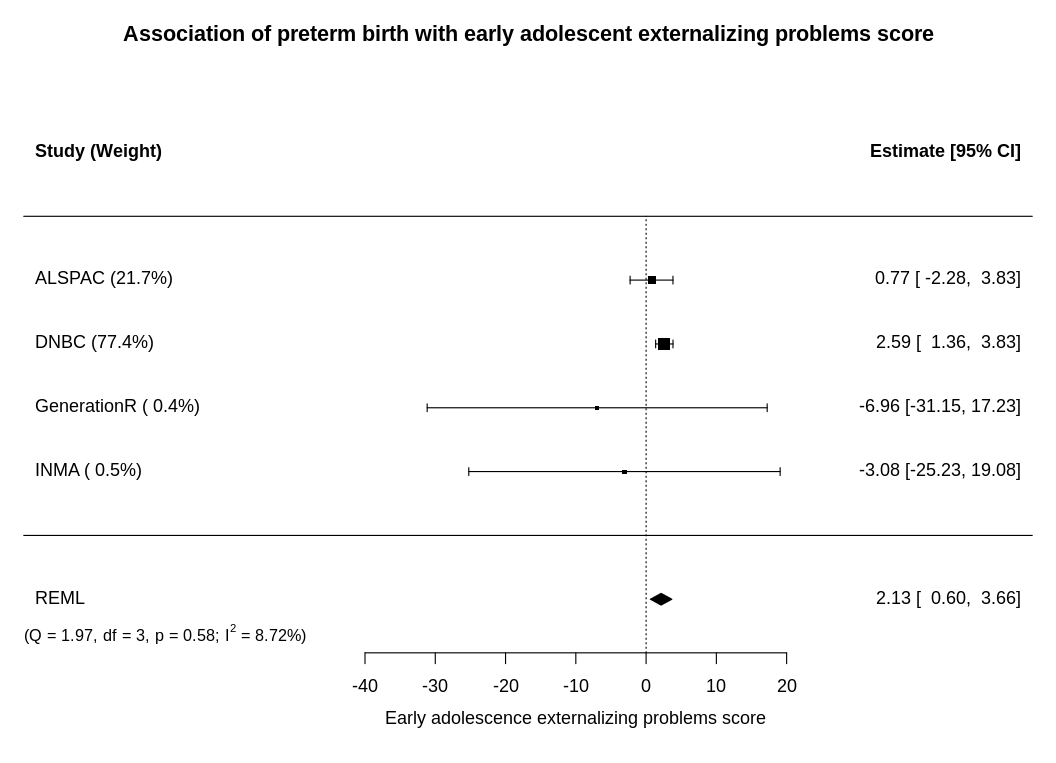
**

**
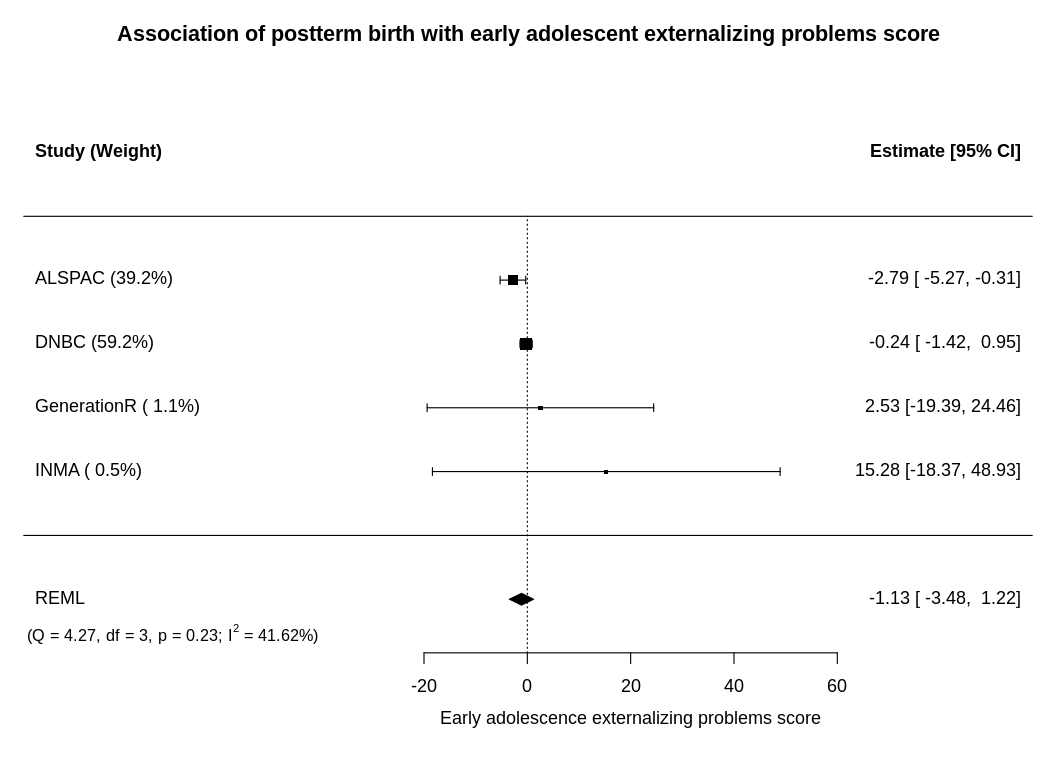
**

**
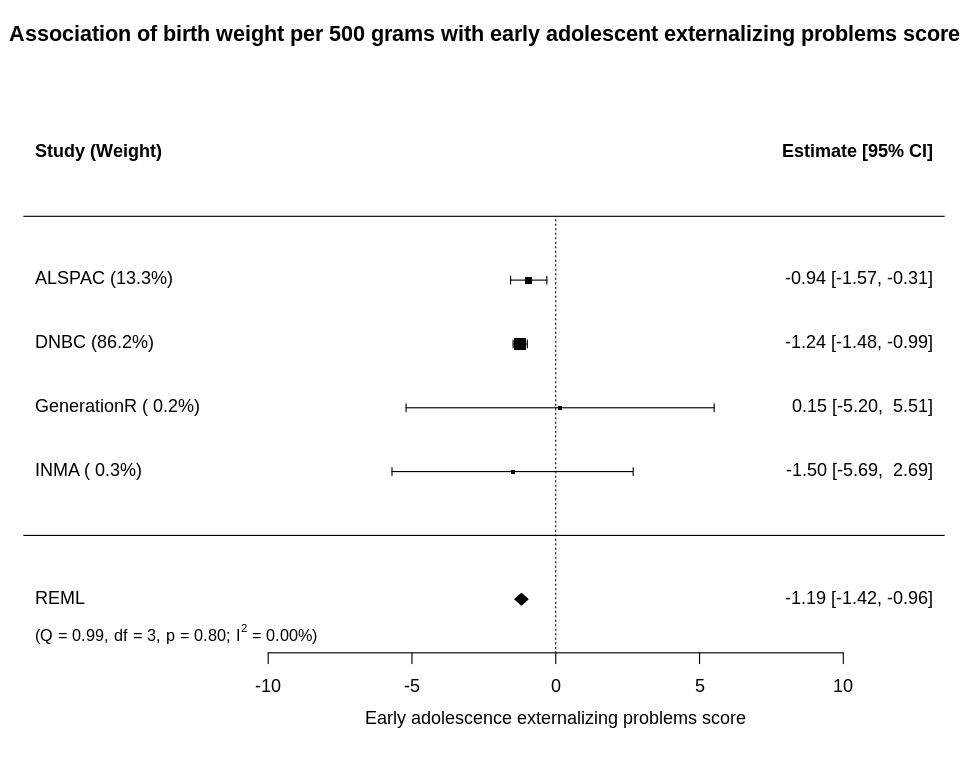
**

**
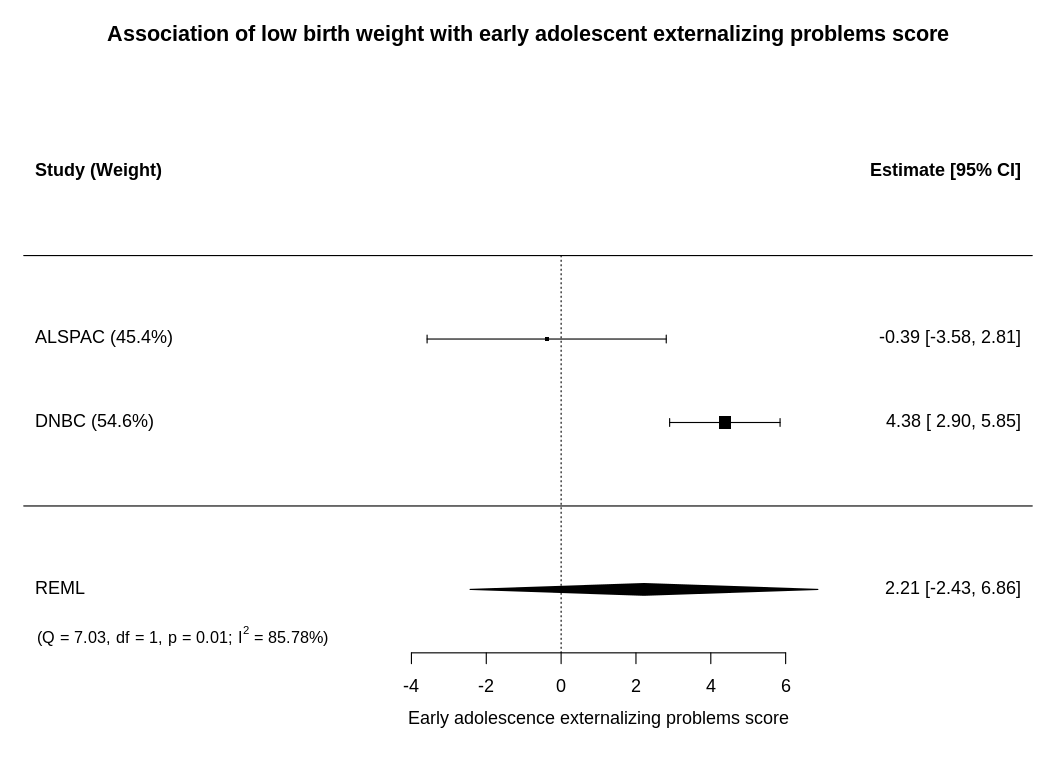
**

**
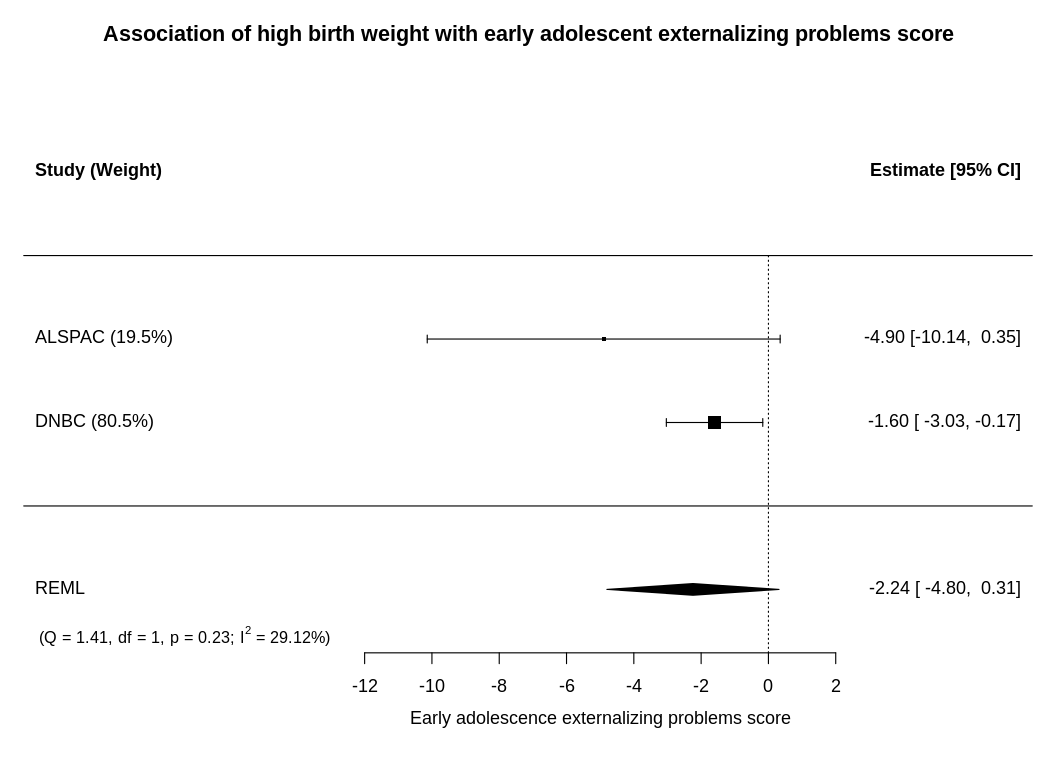
**

**
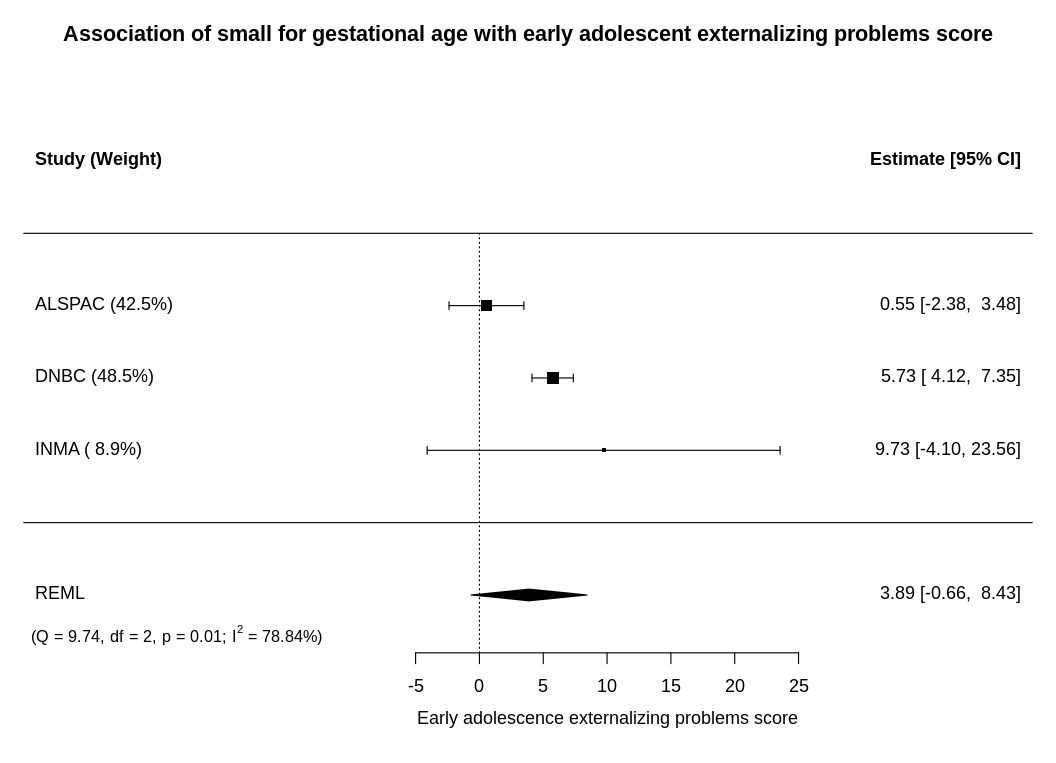
**

**
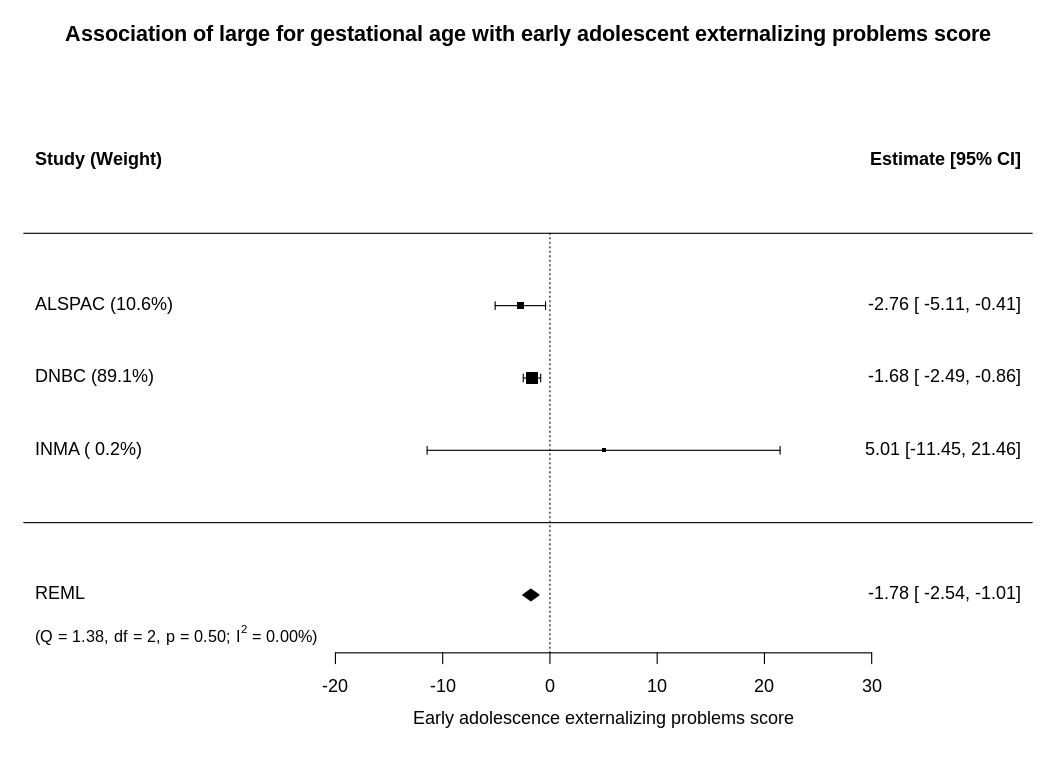
**

**
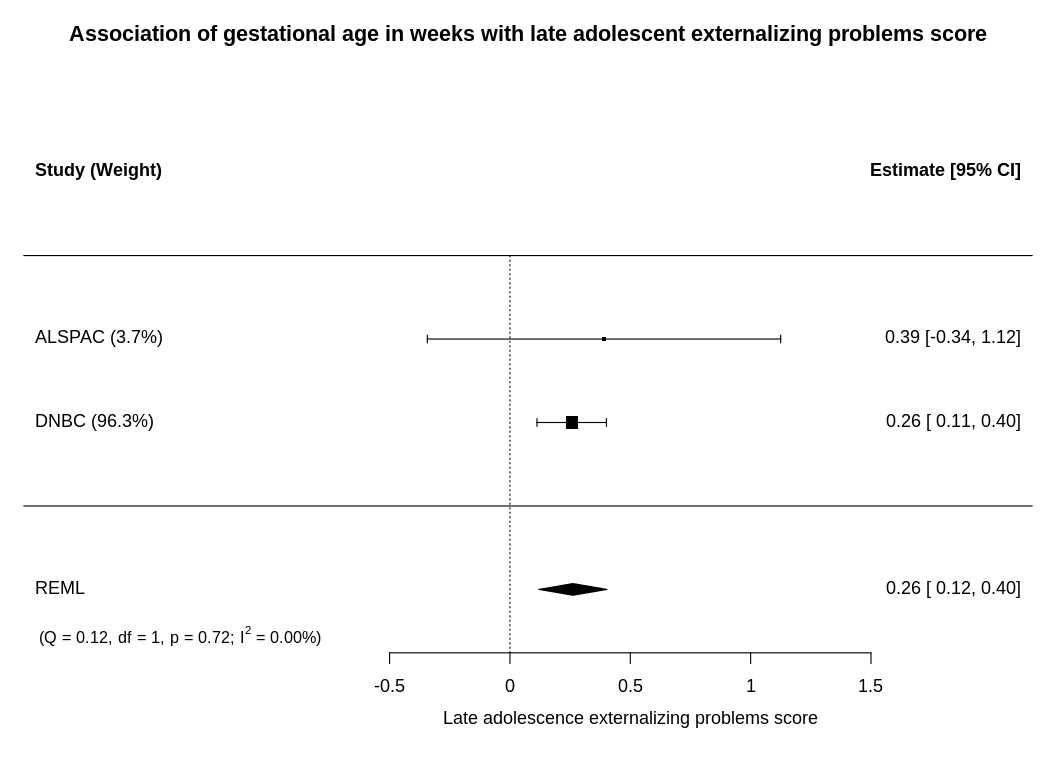
**

**
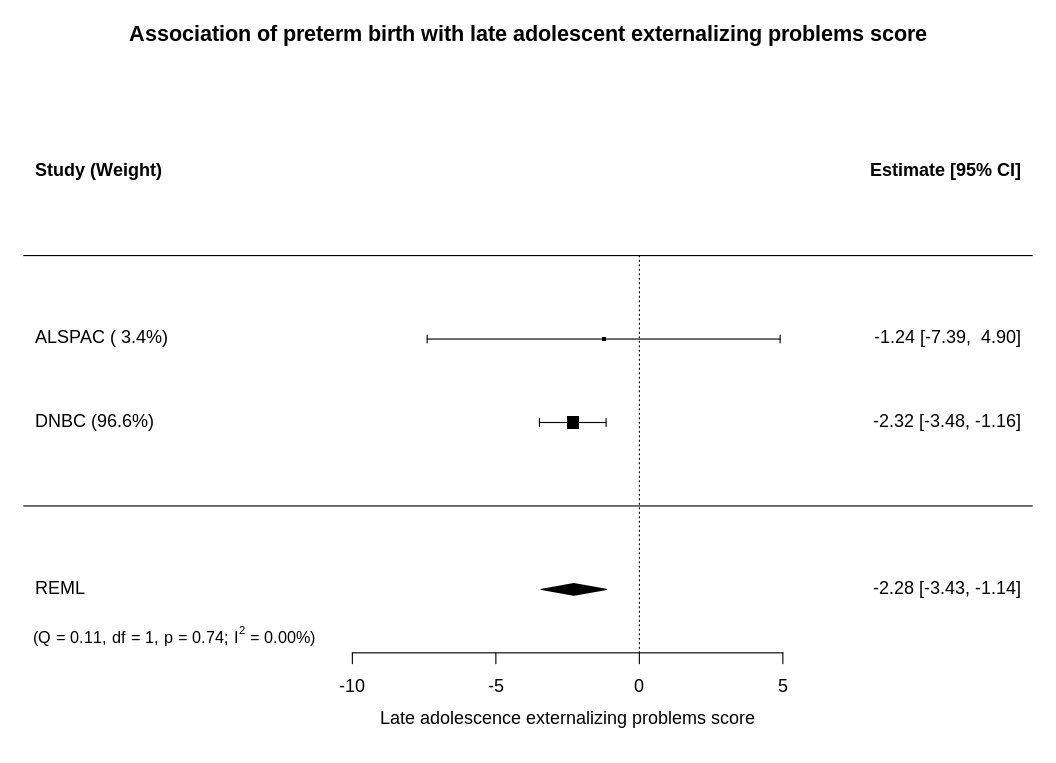
**

**
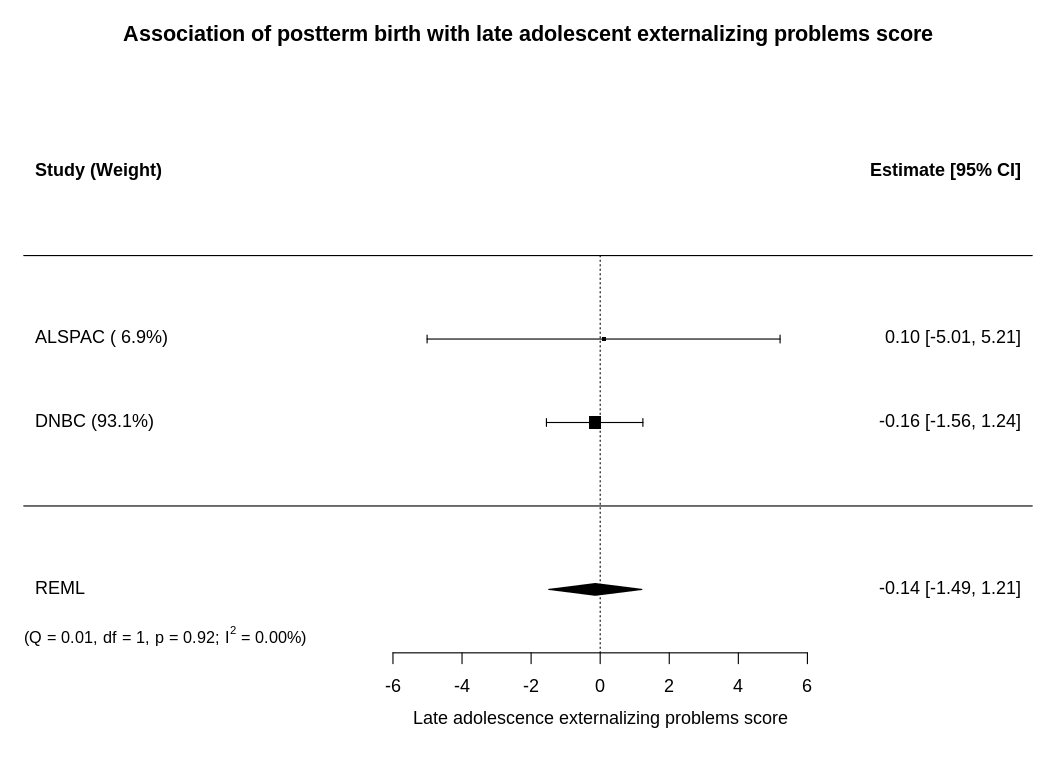
**

**
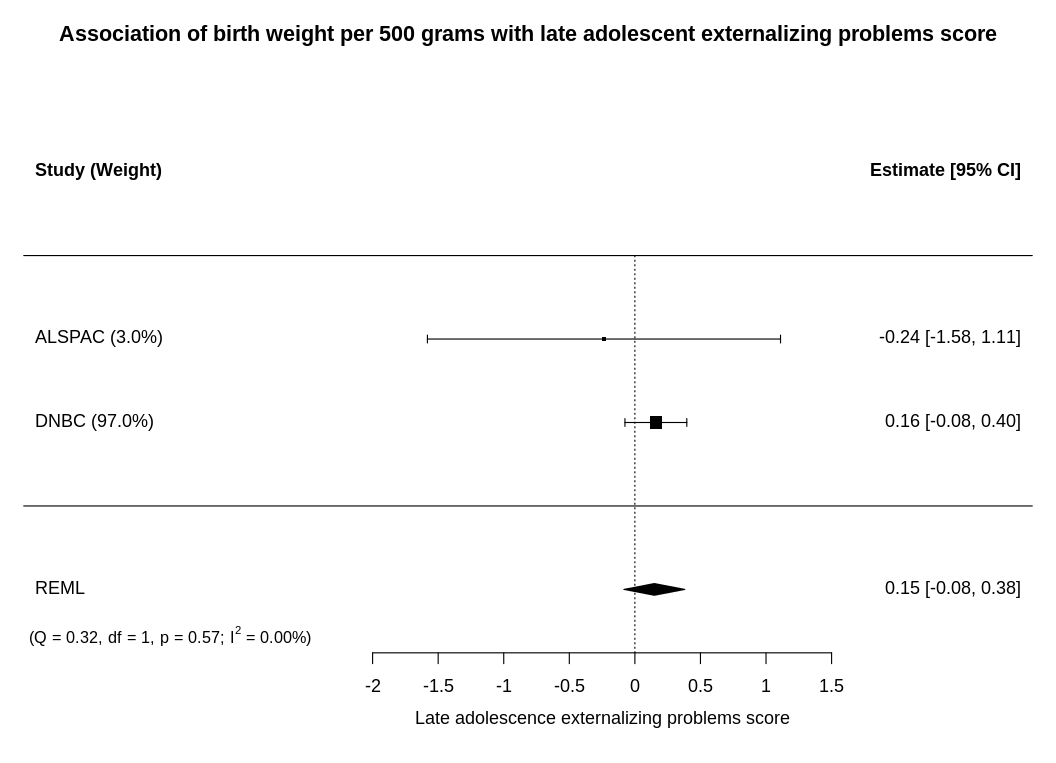
**

**
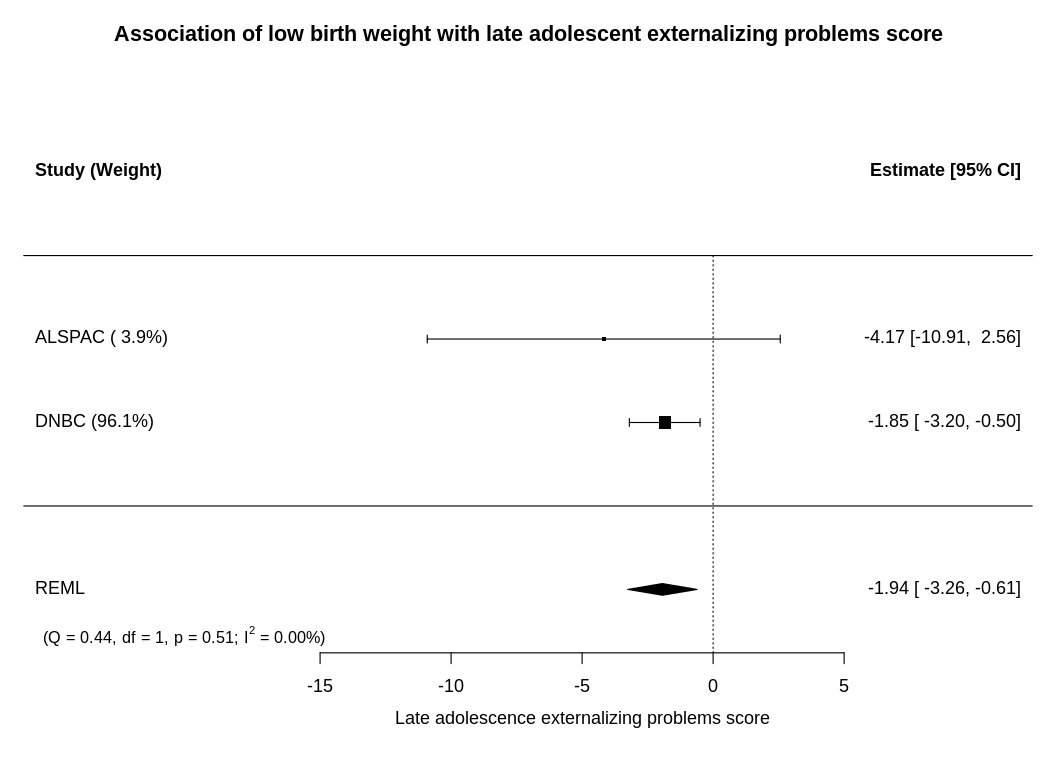
**

**
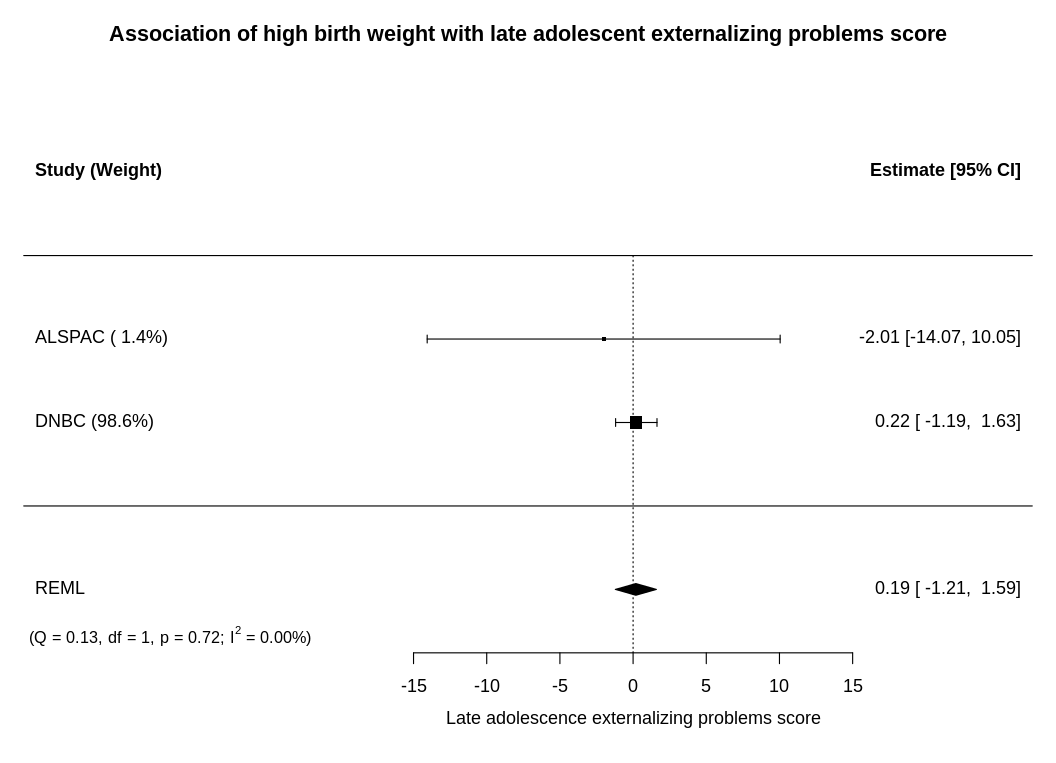
**

**
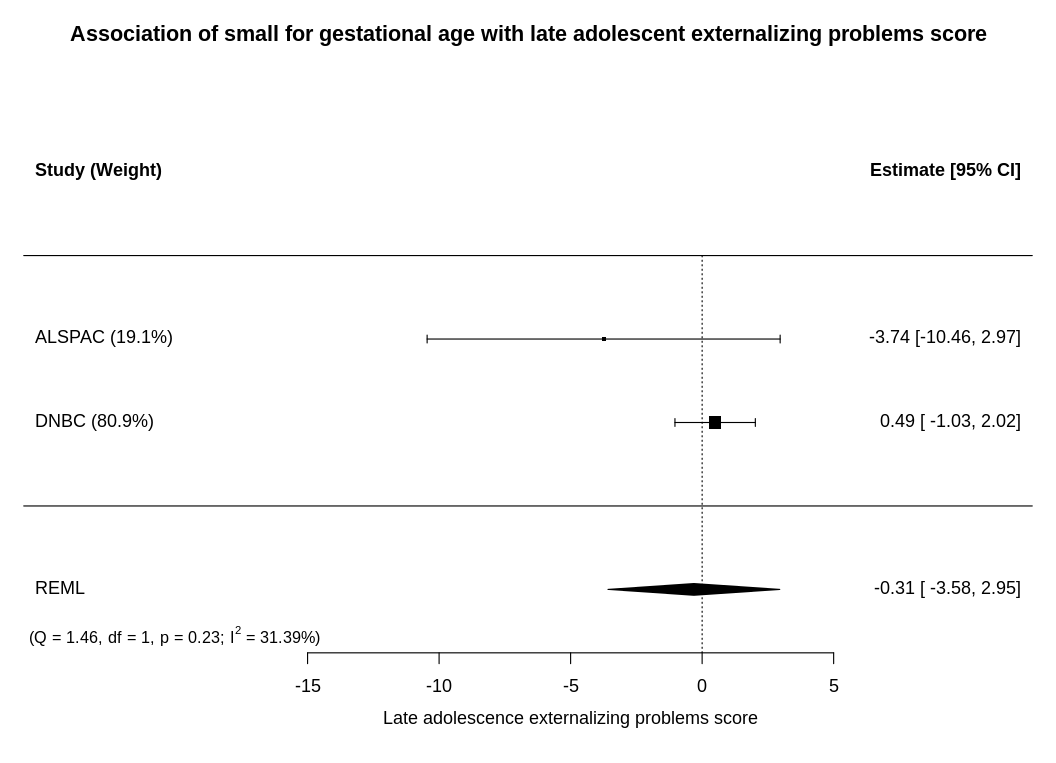
**

**
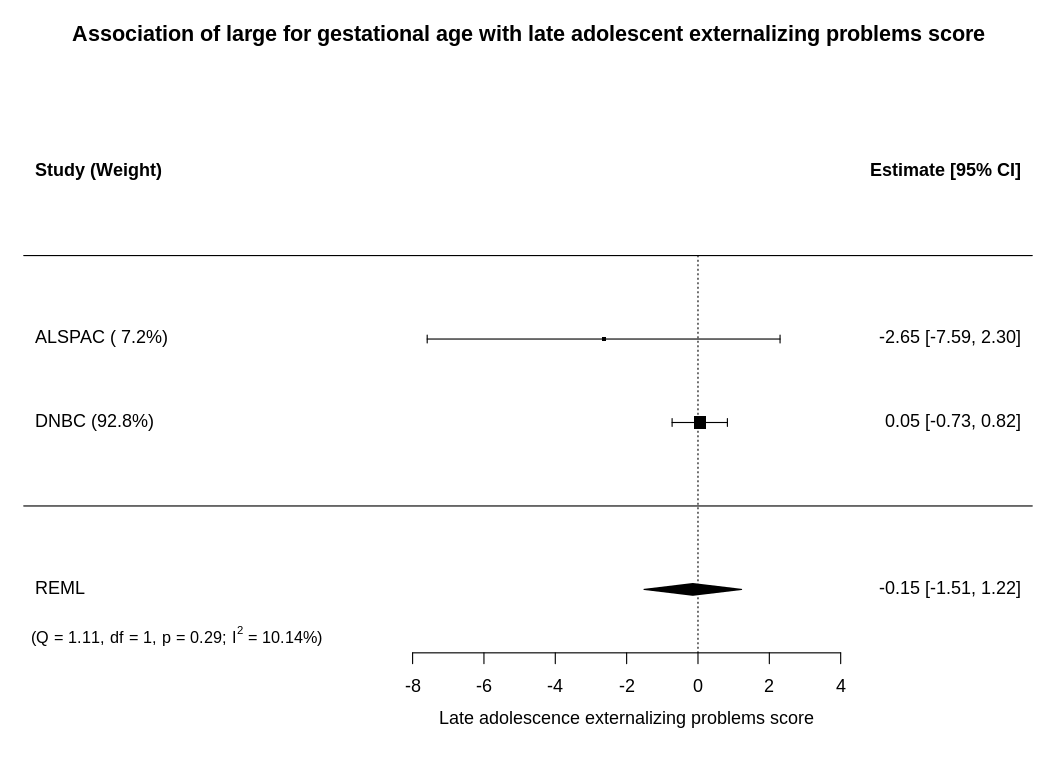
**

**Outcome: ADHD symptoms**

**
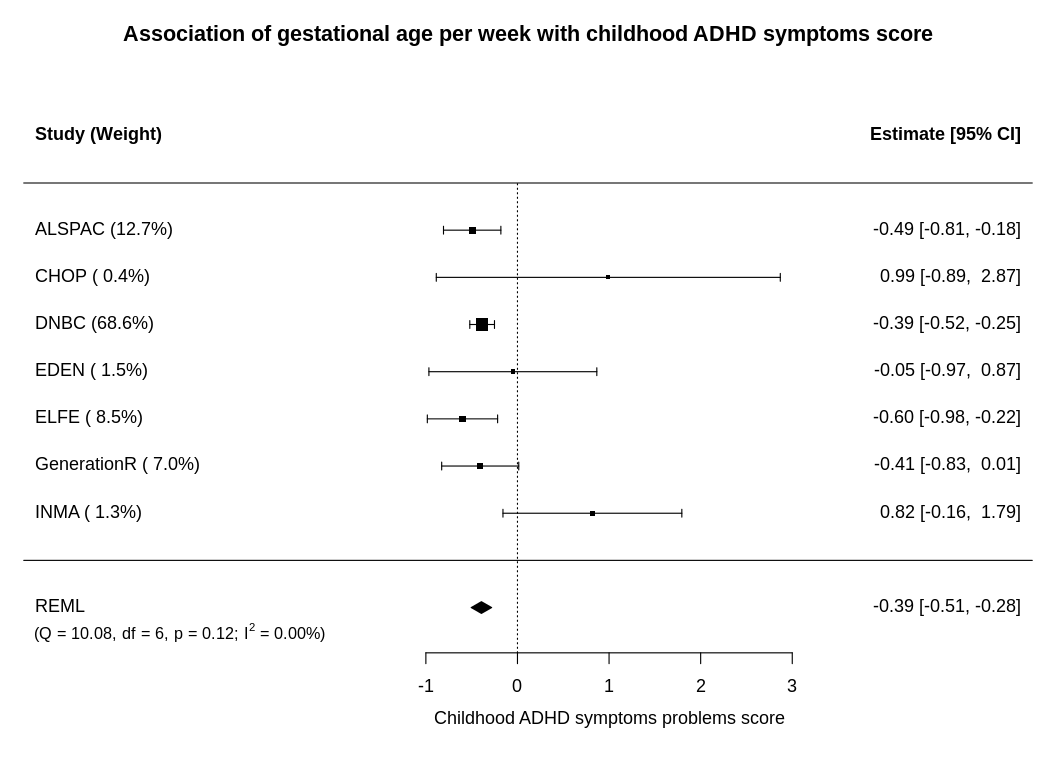
**


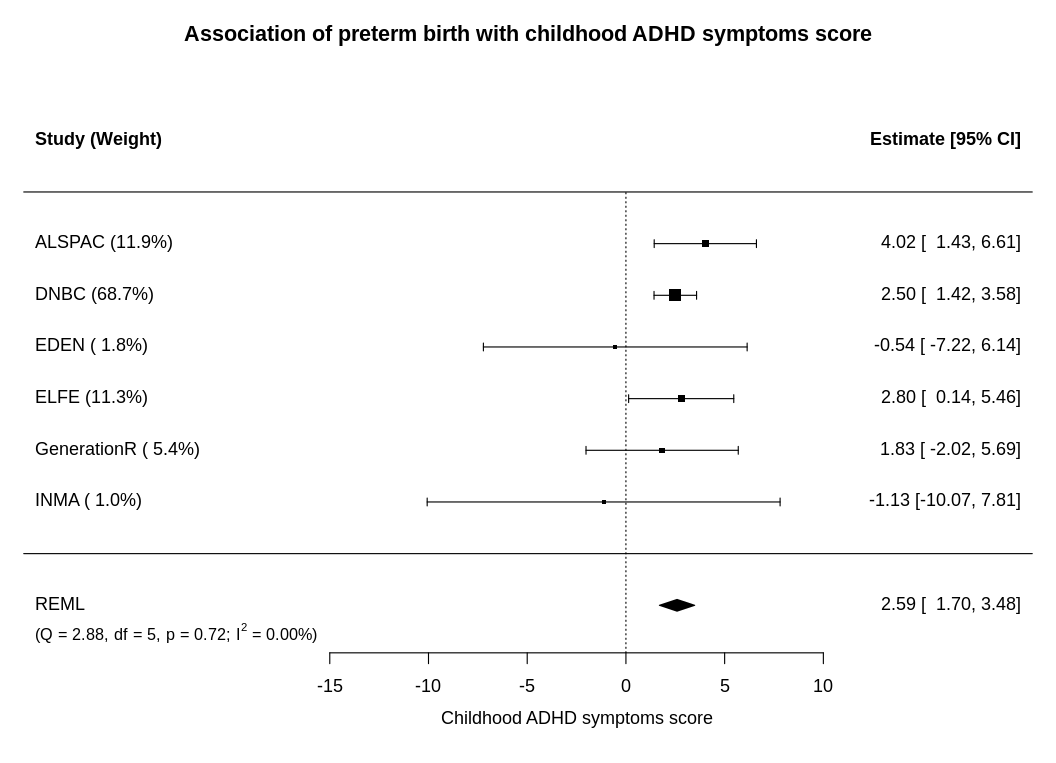


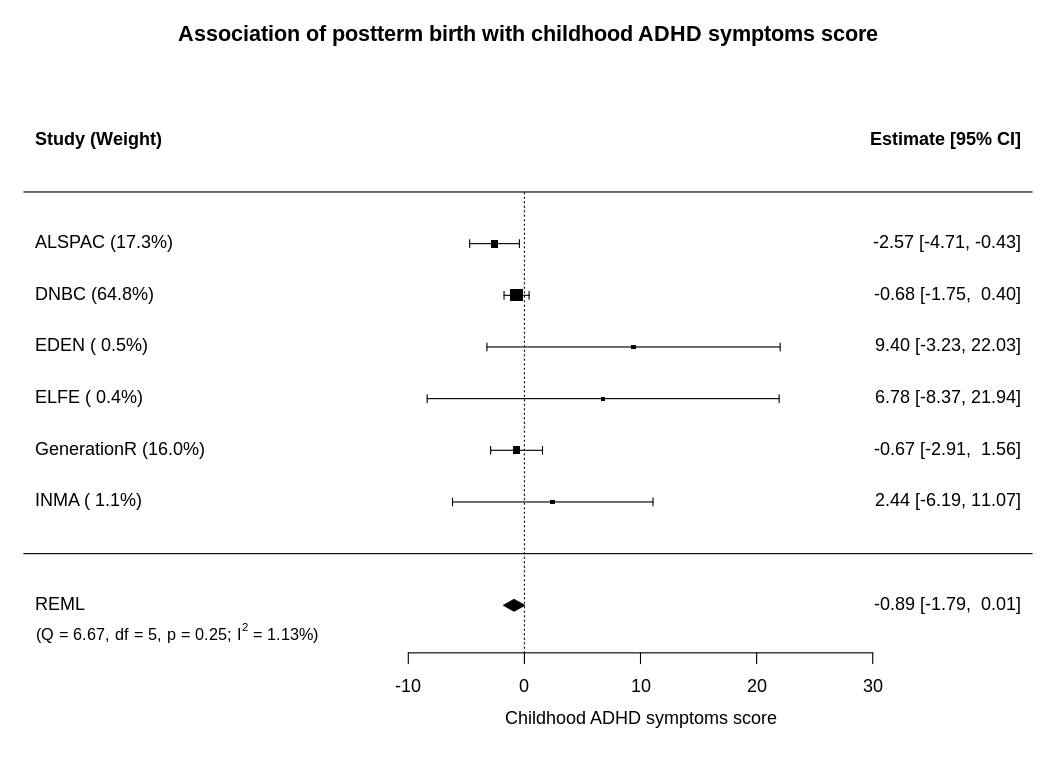


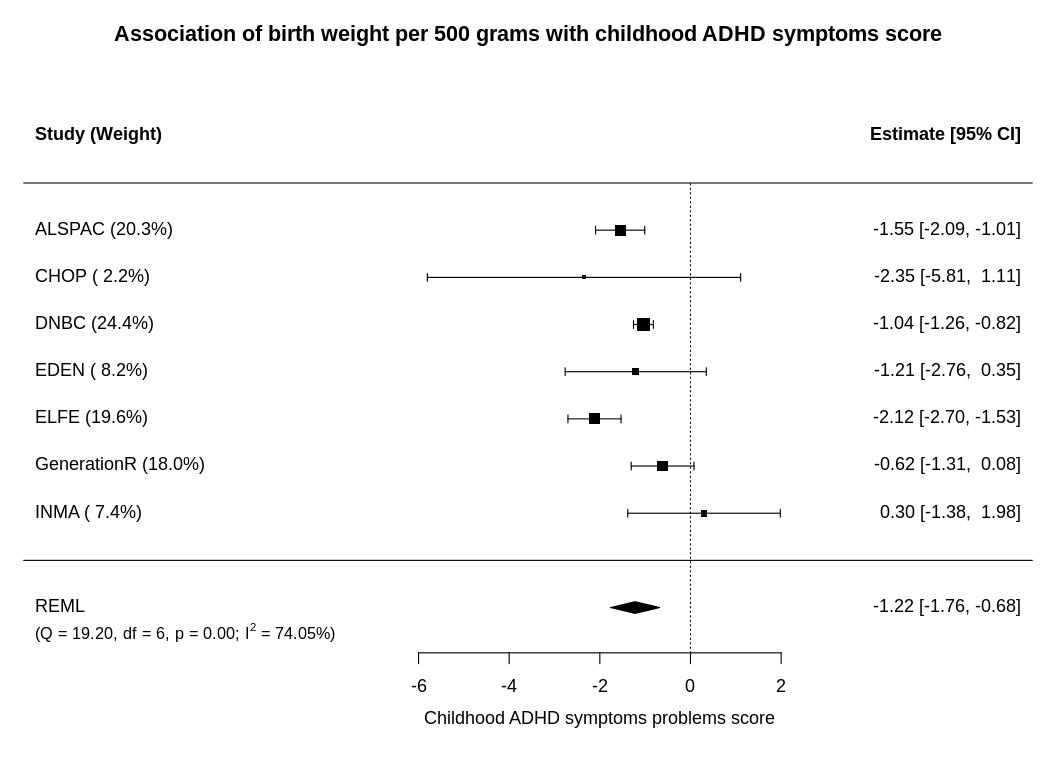


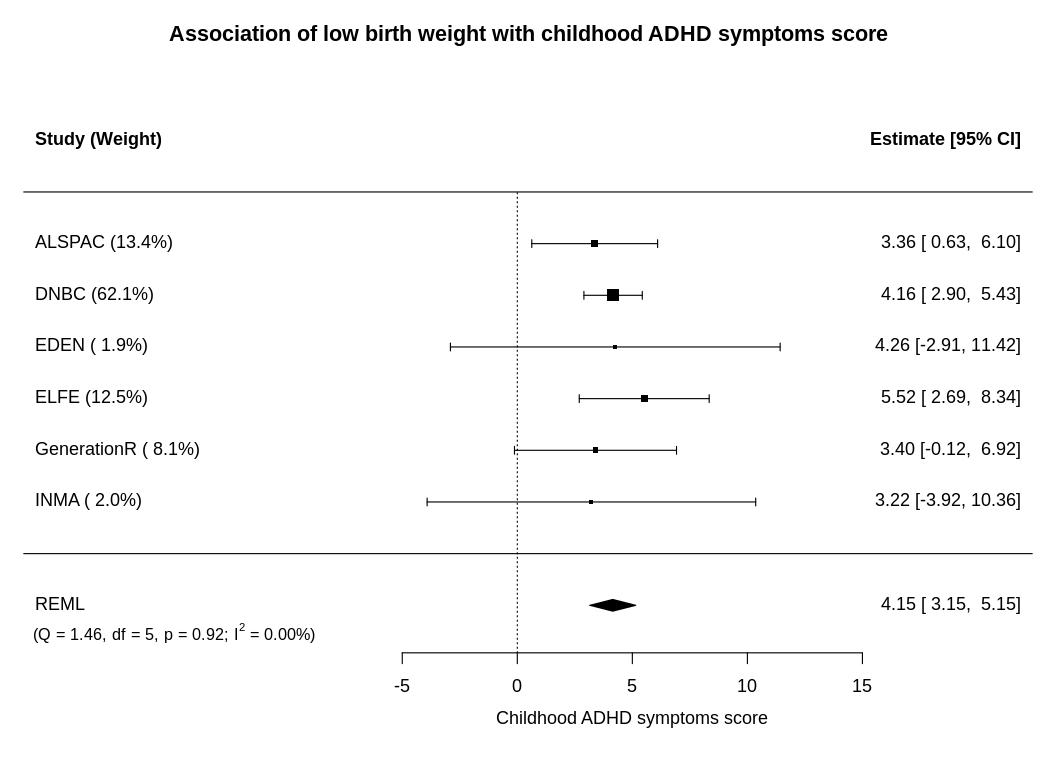


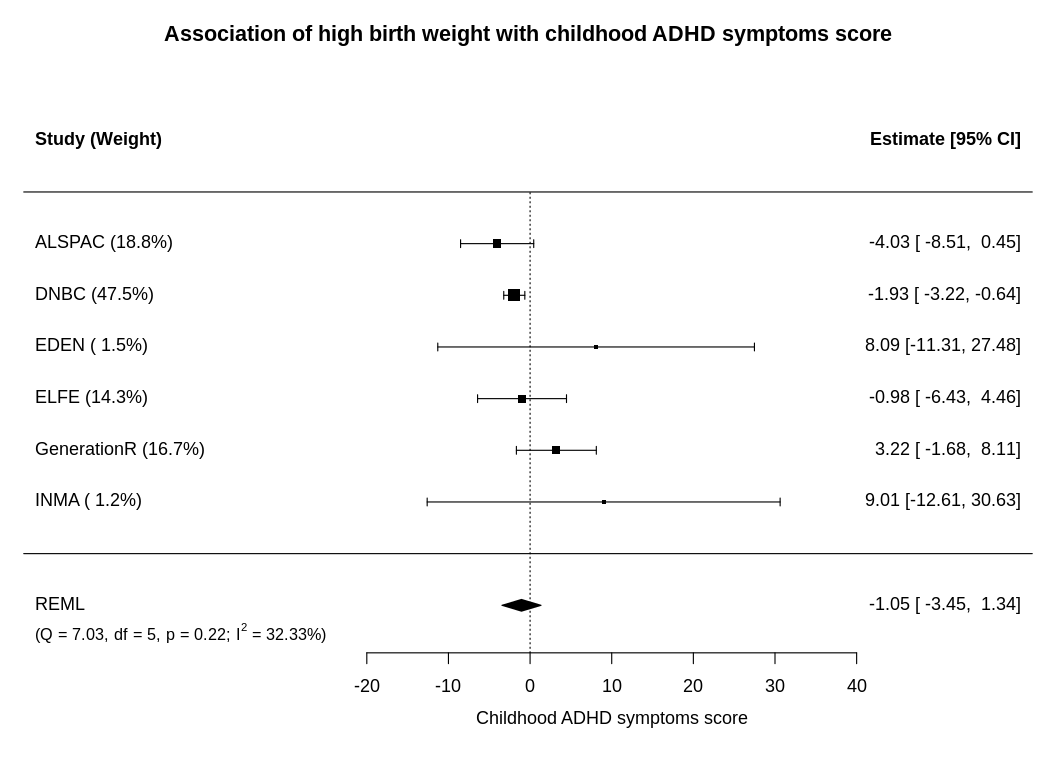


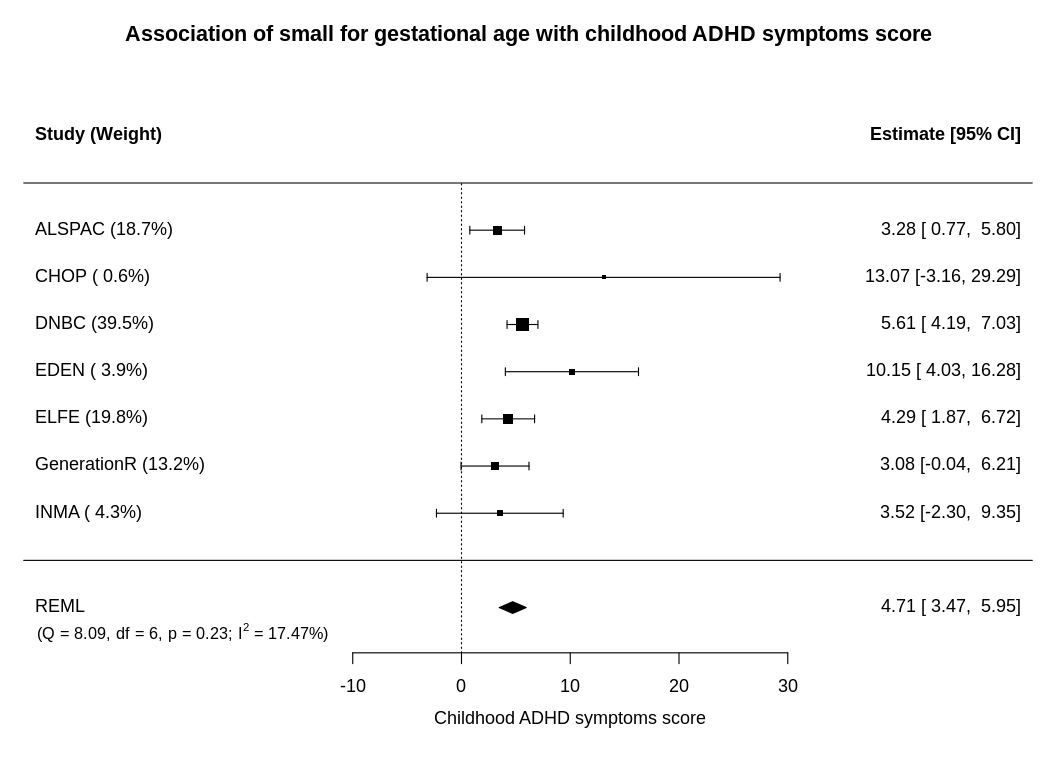


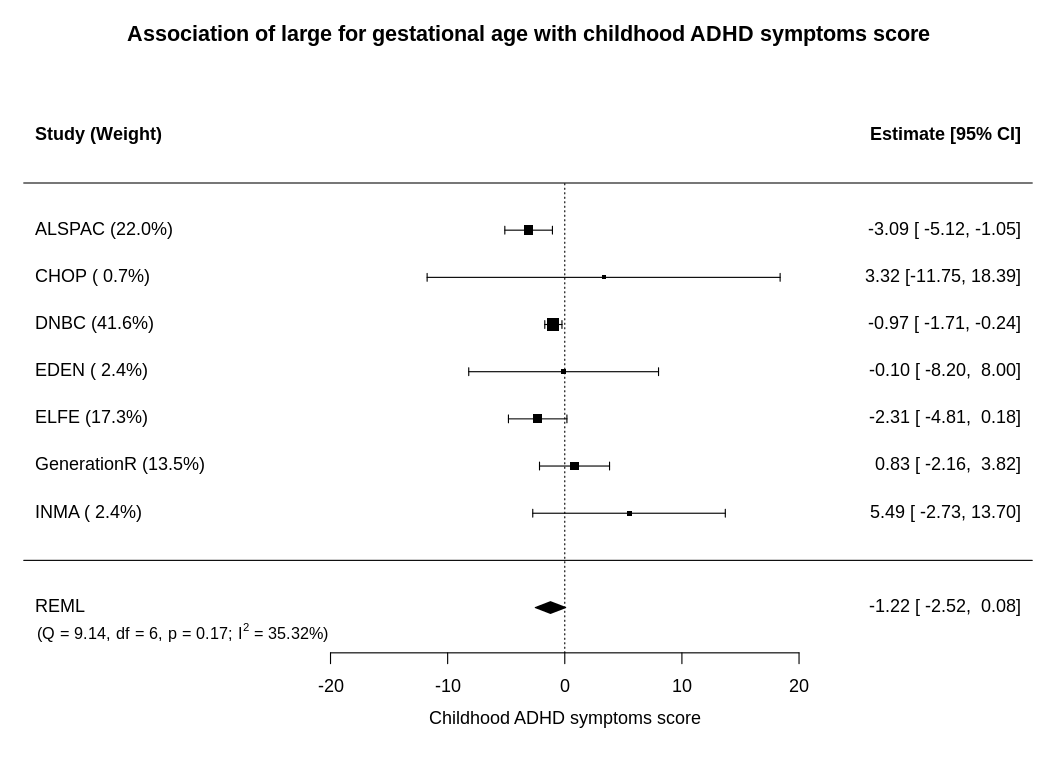


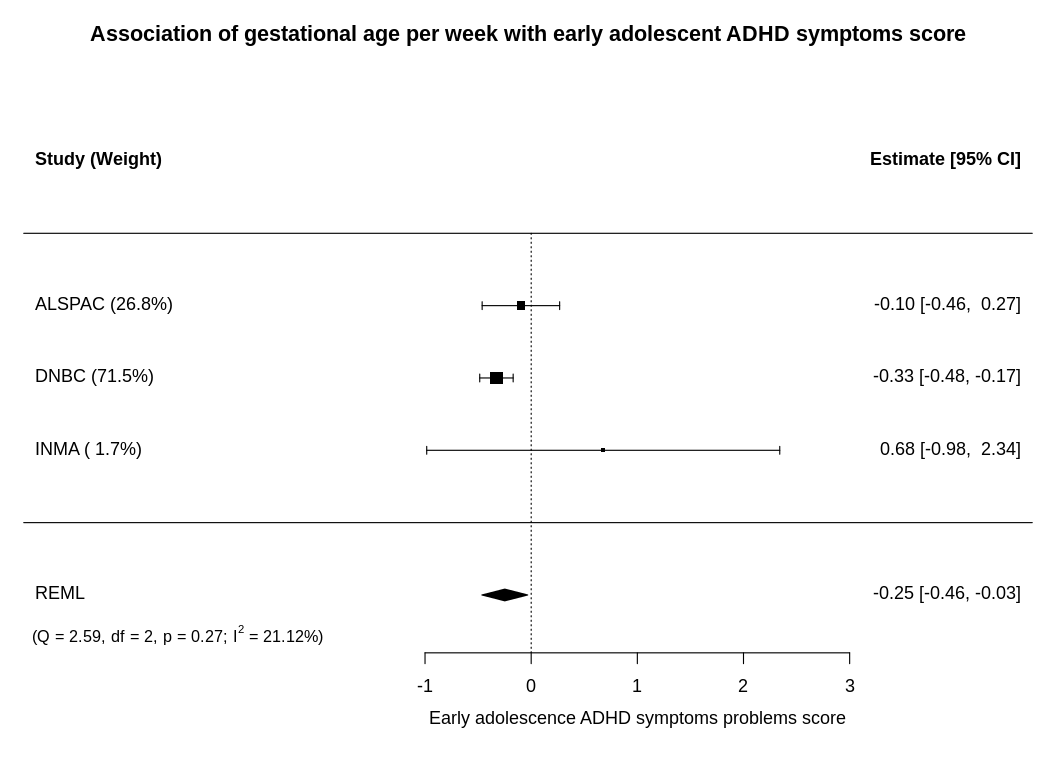


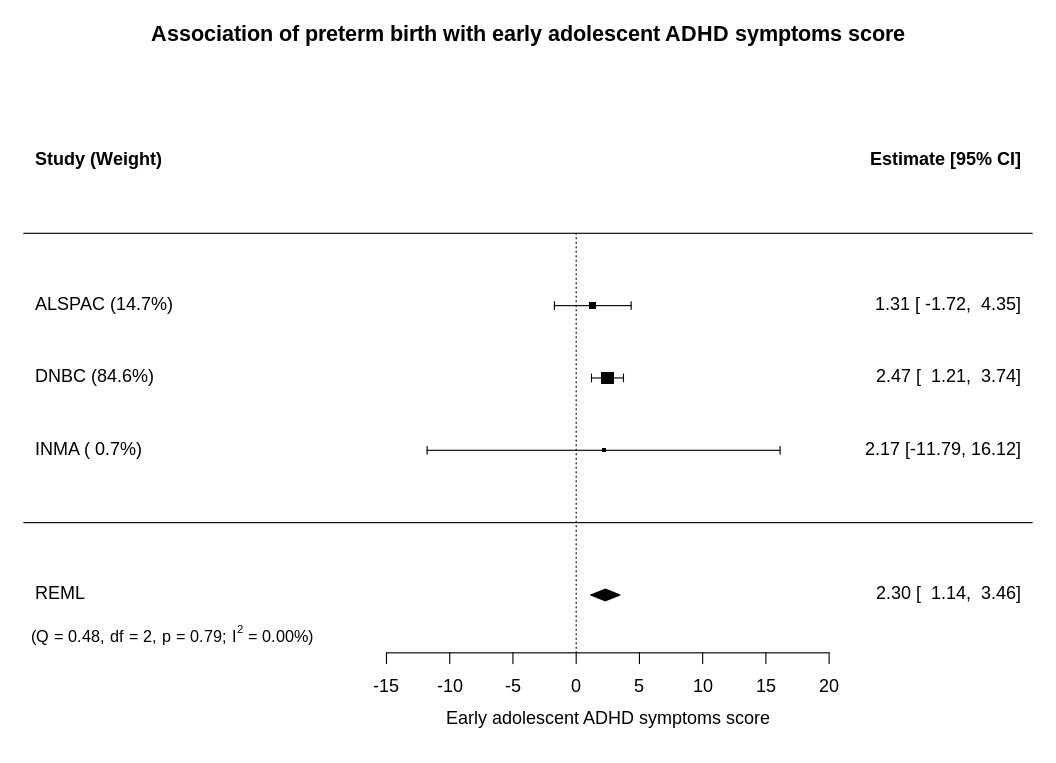


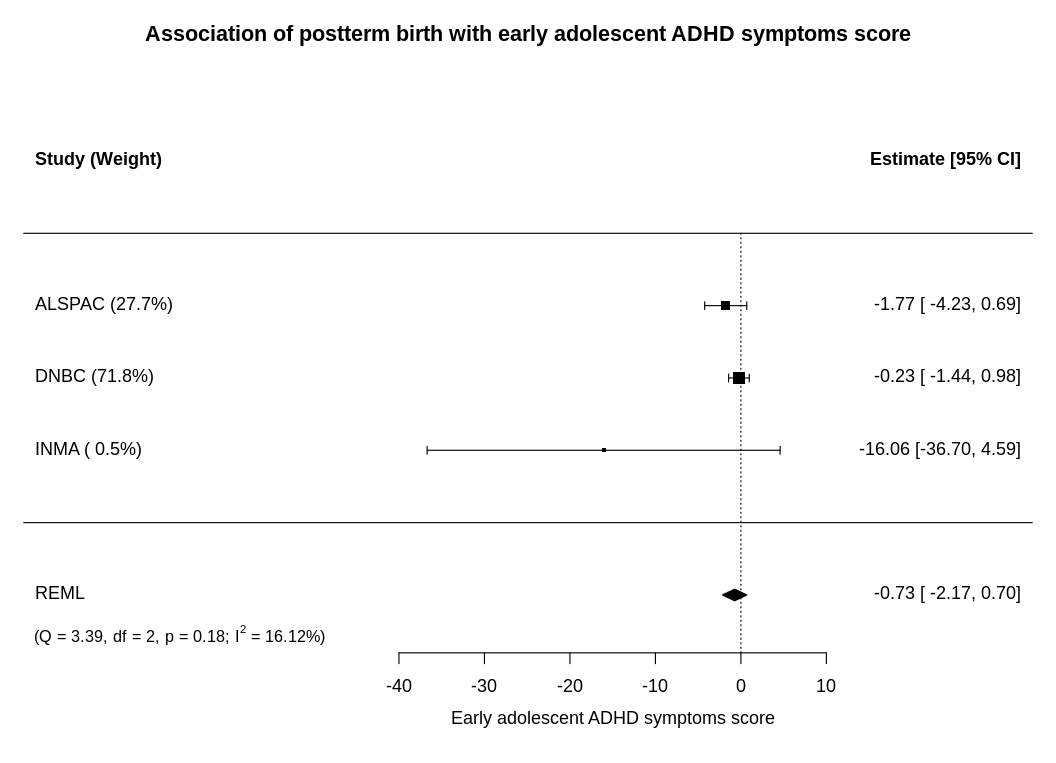


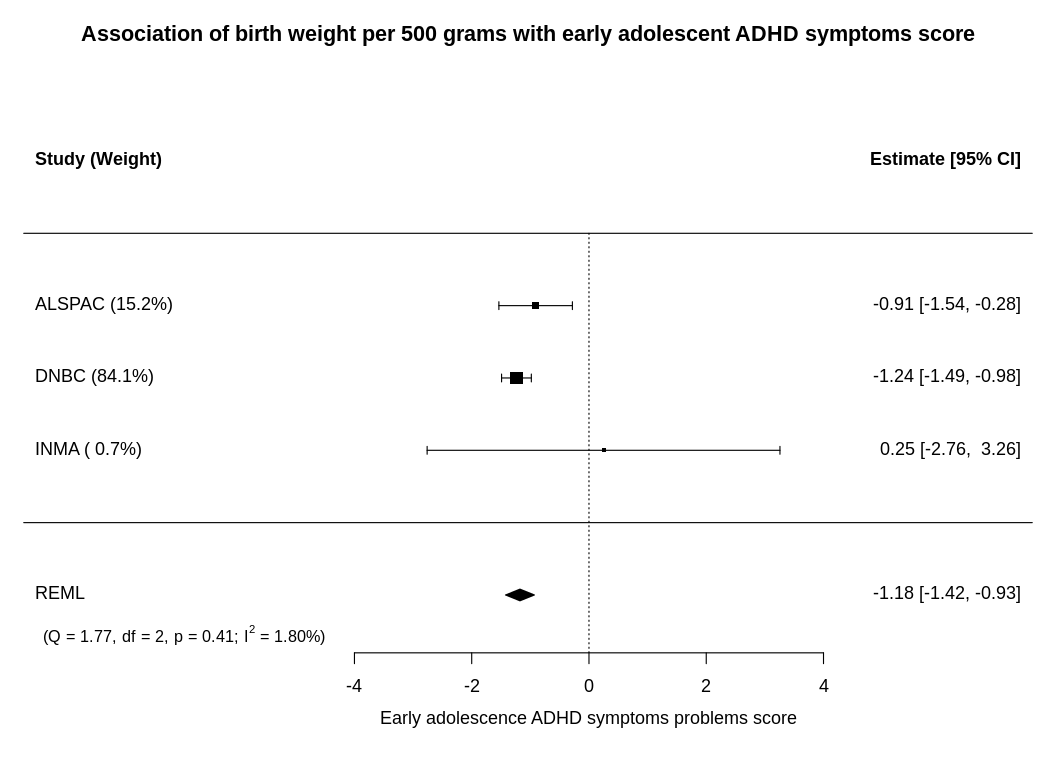


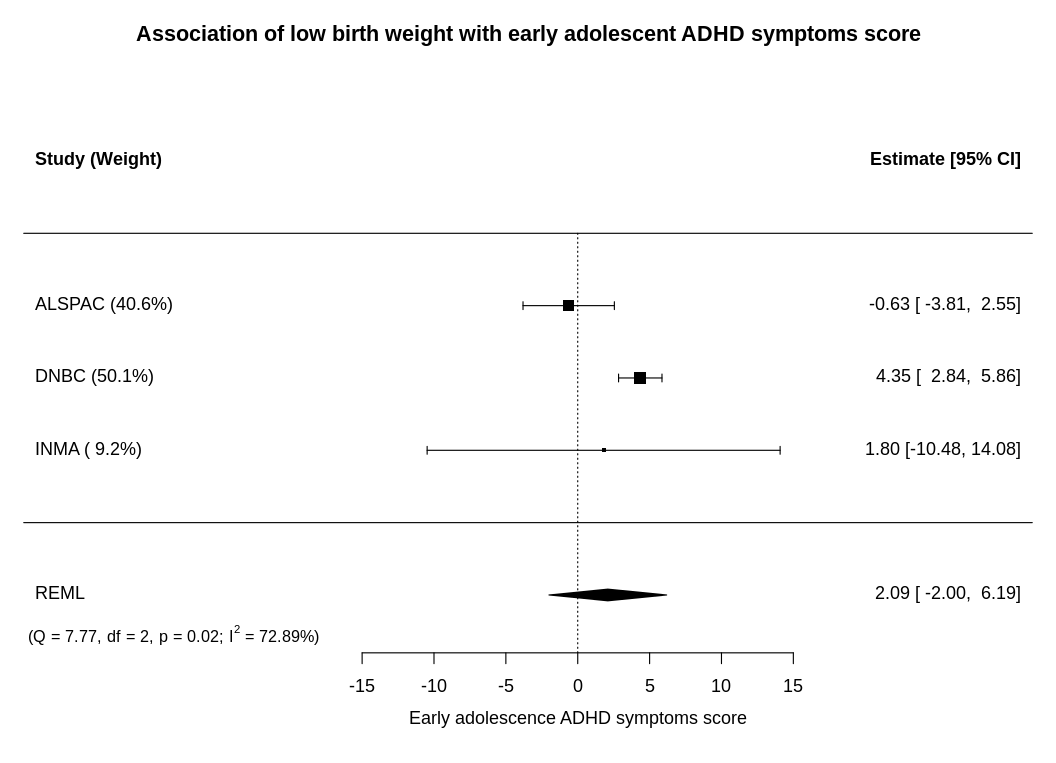


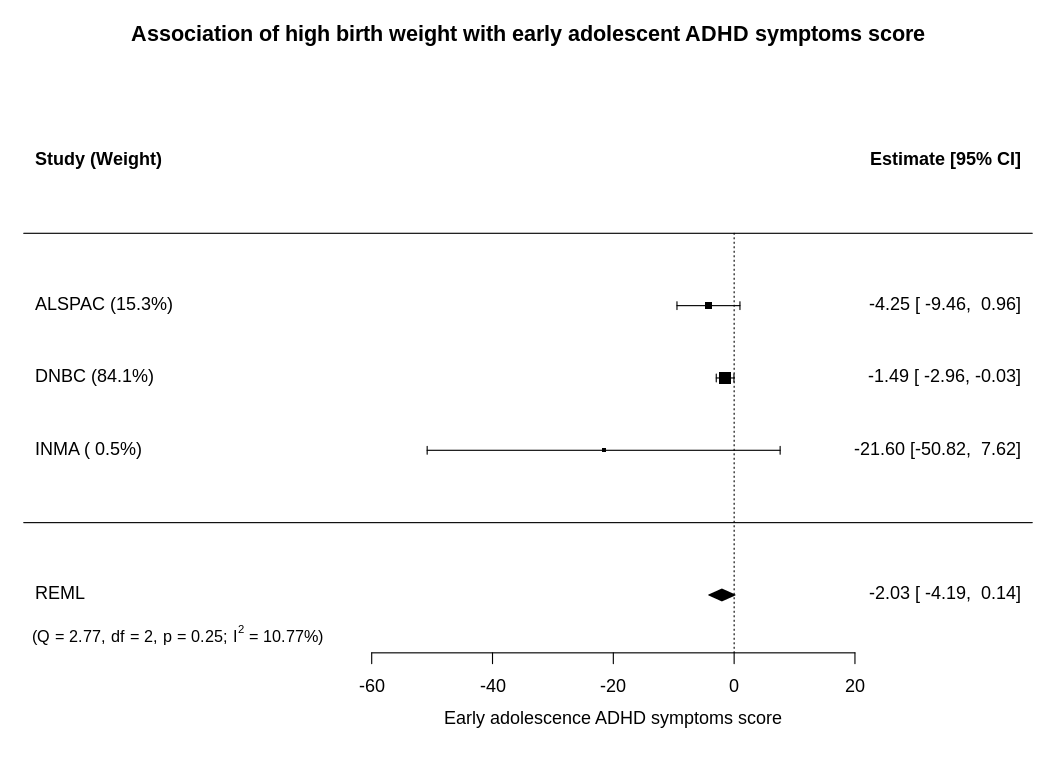


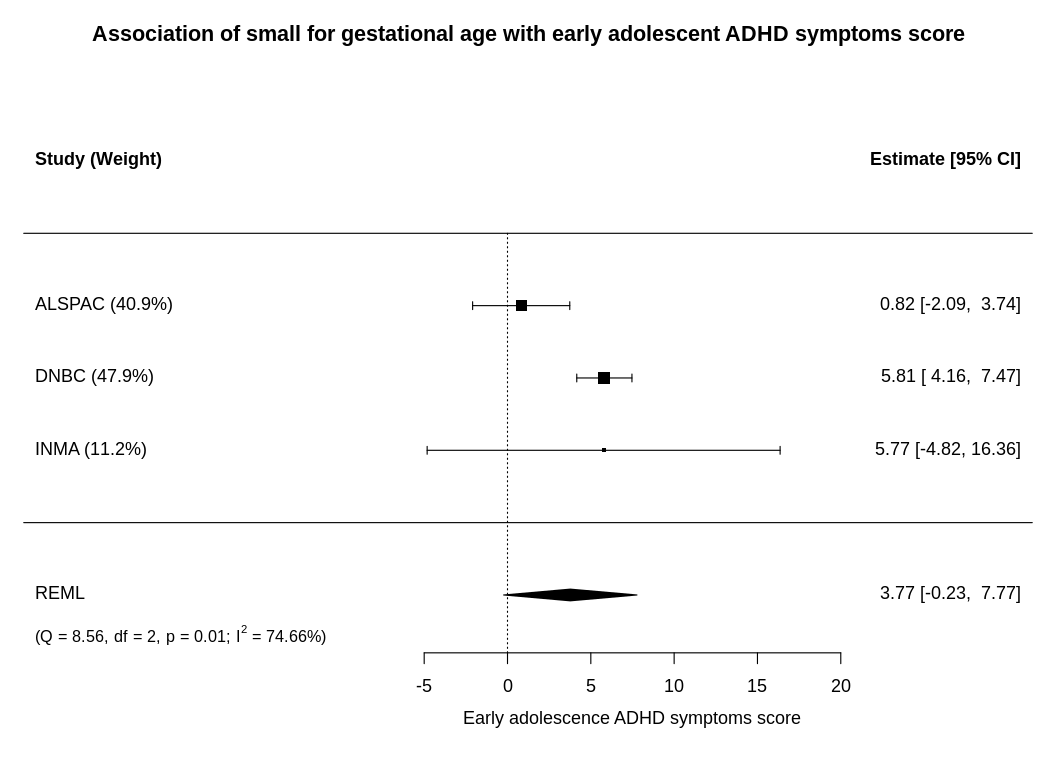


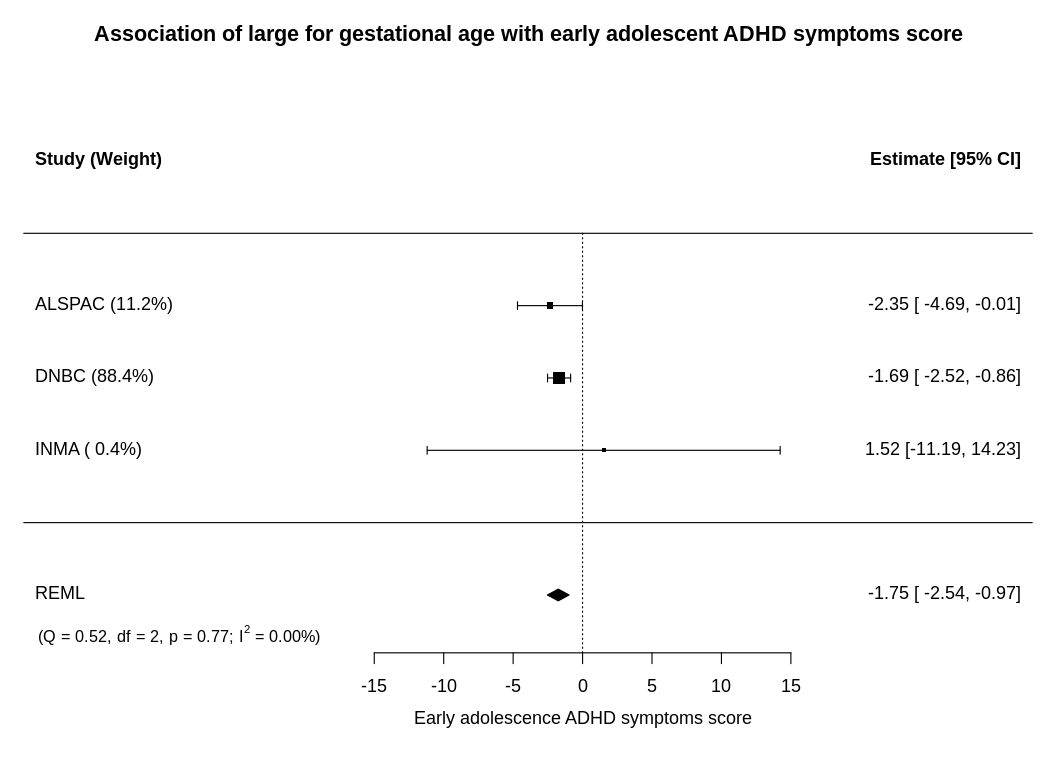


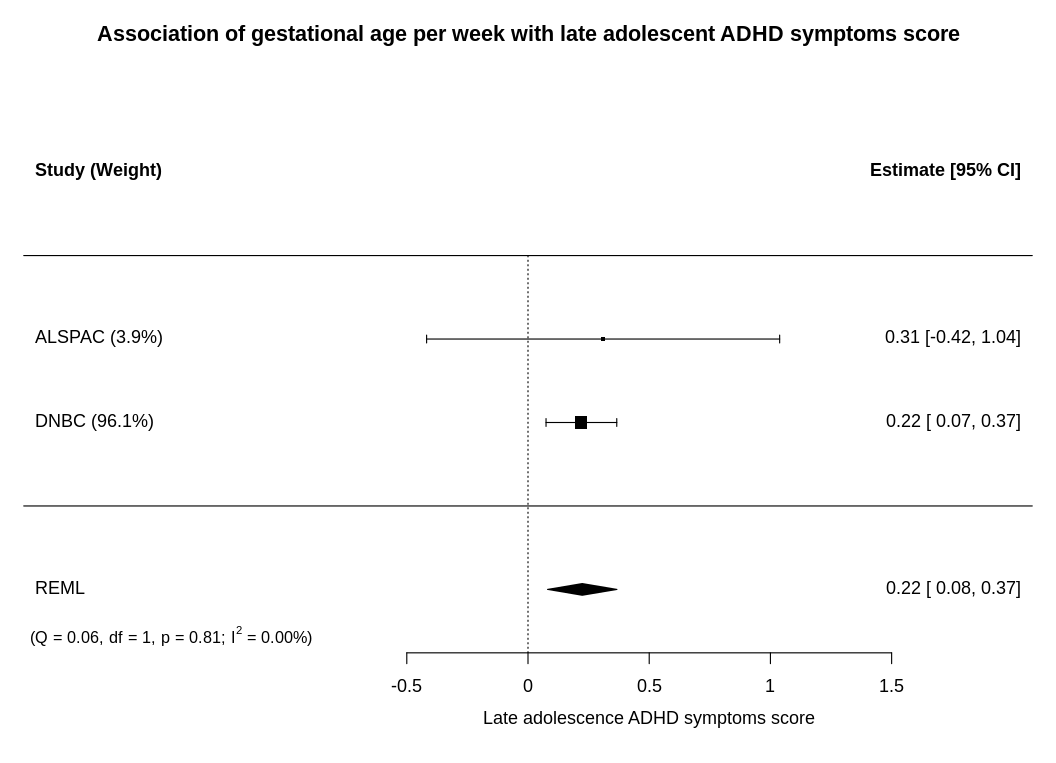


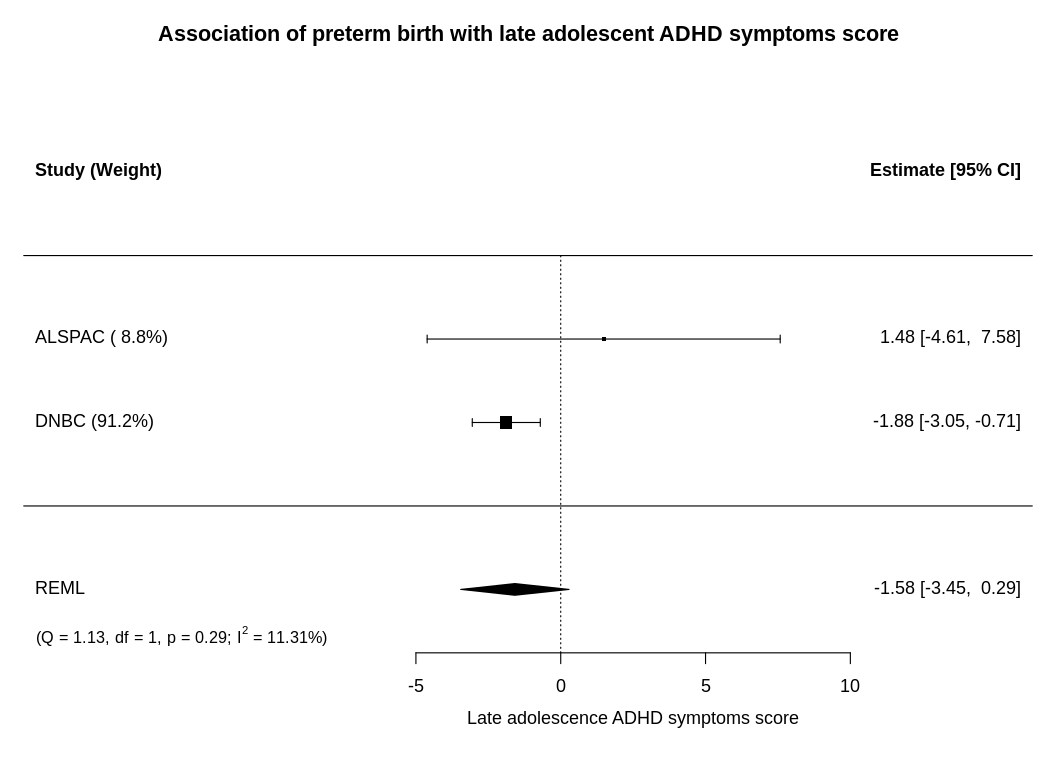


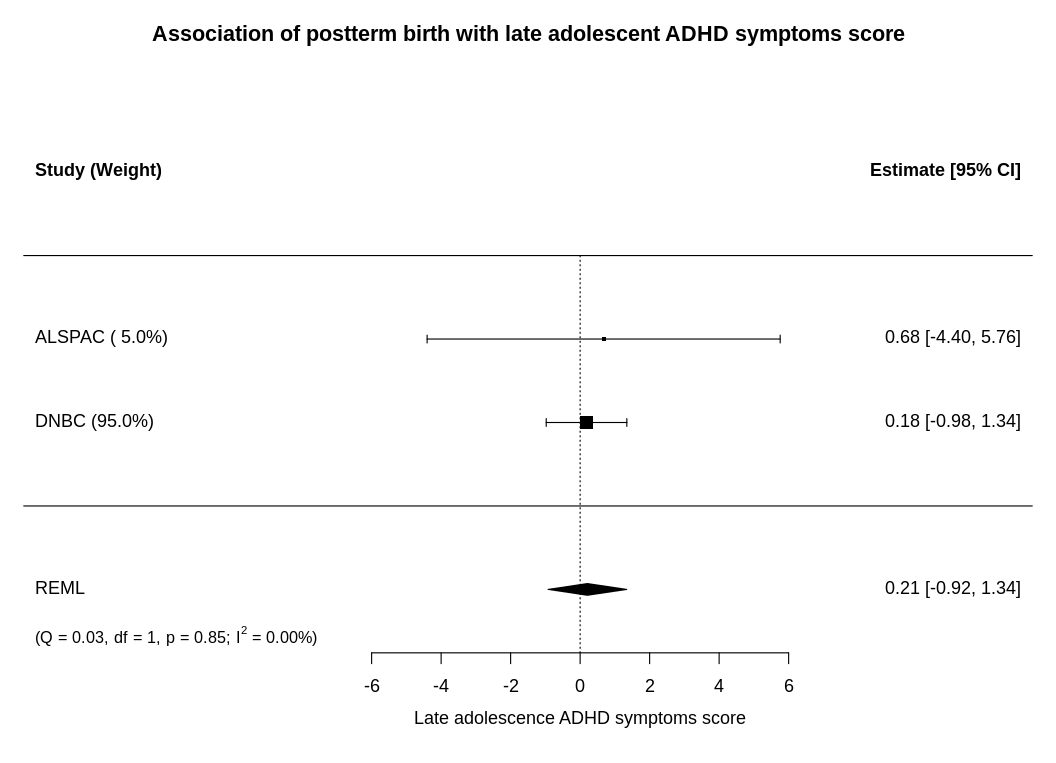


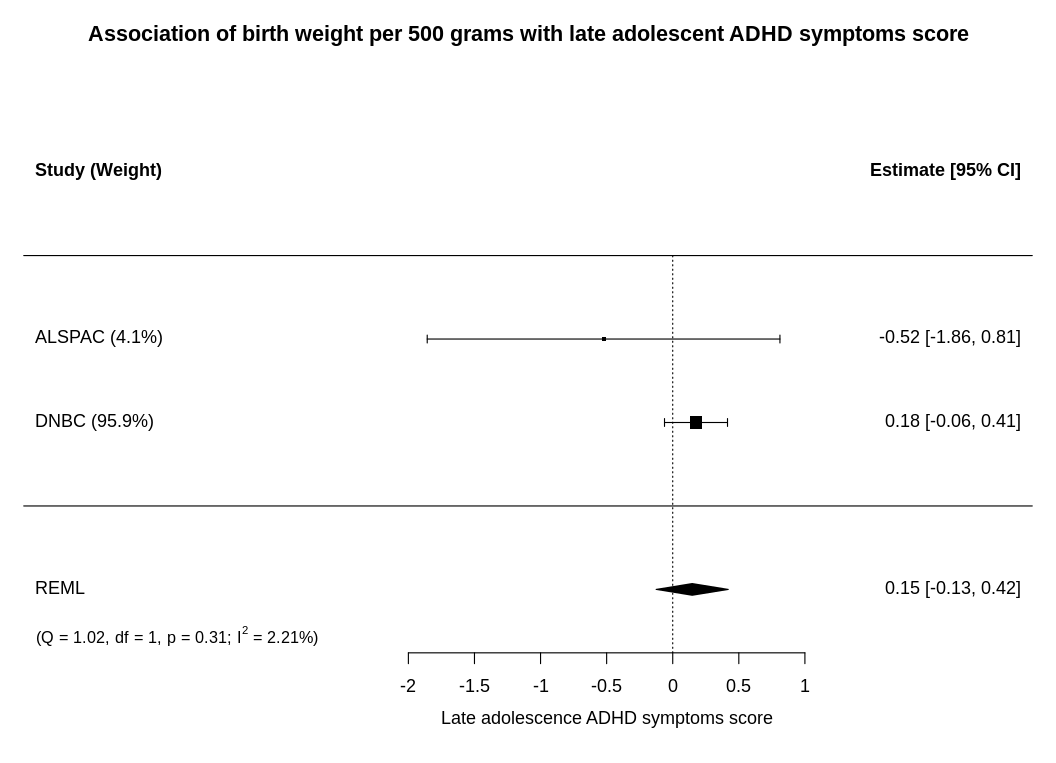


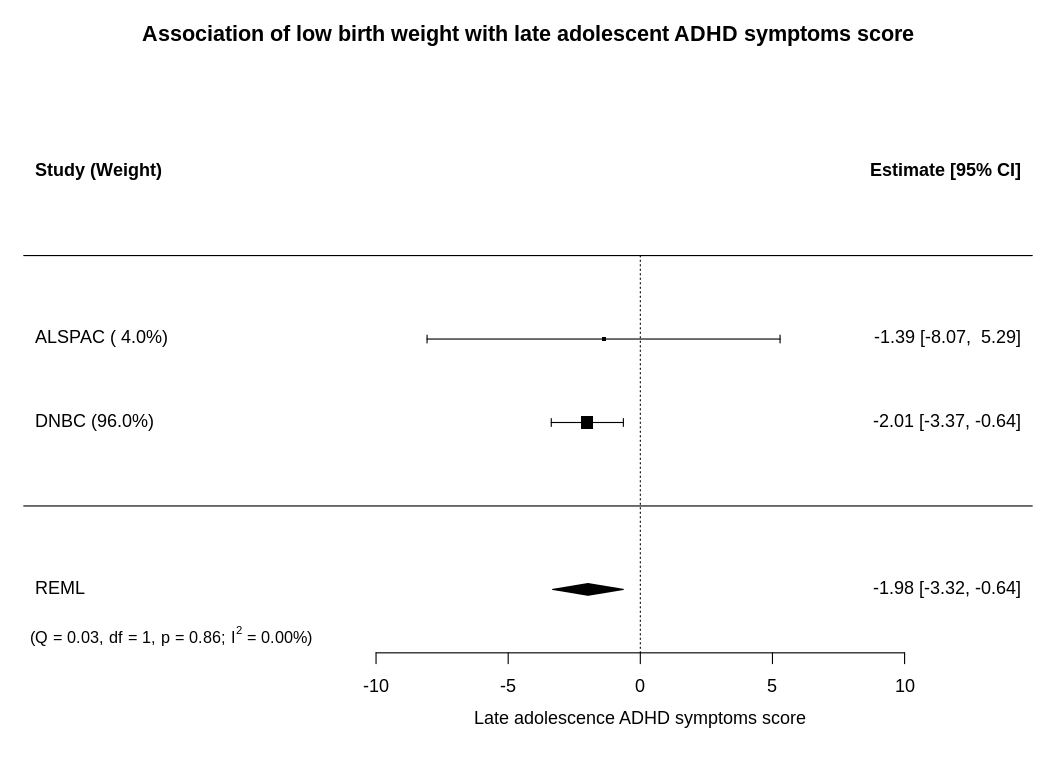


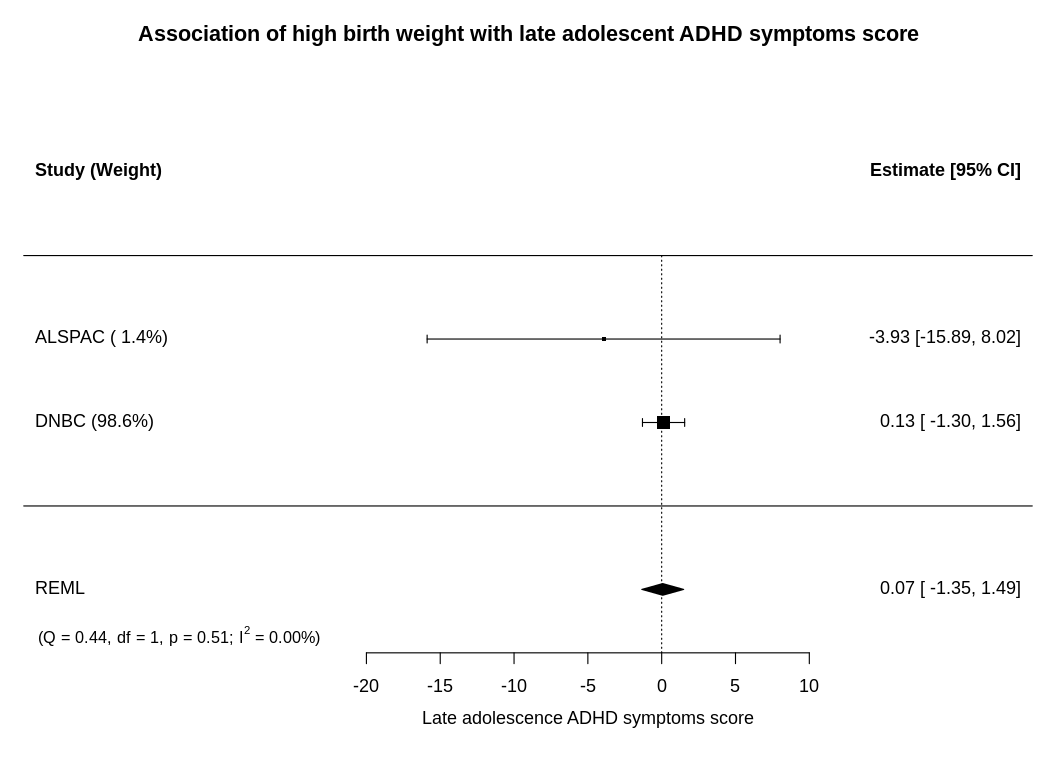


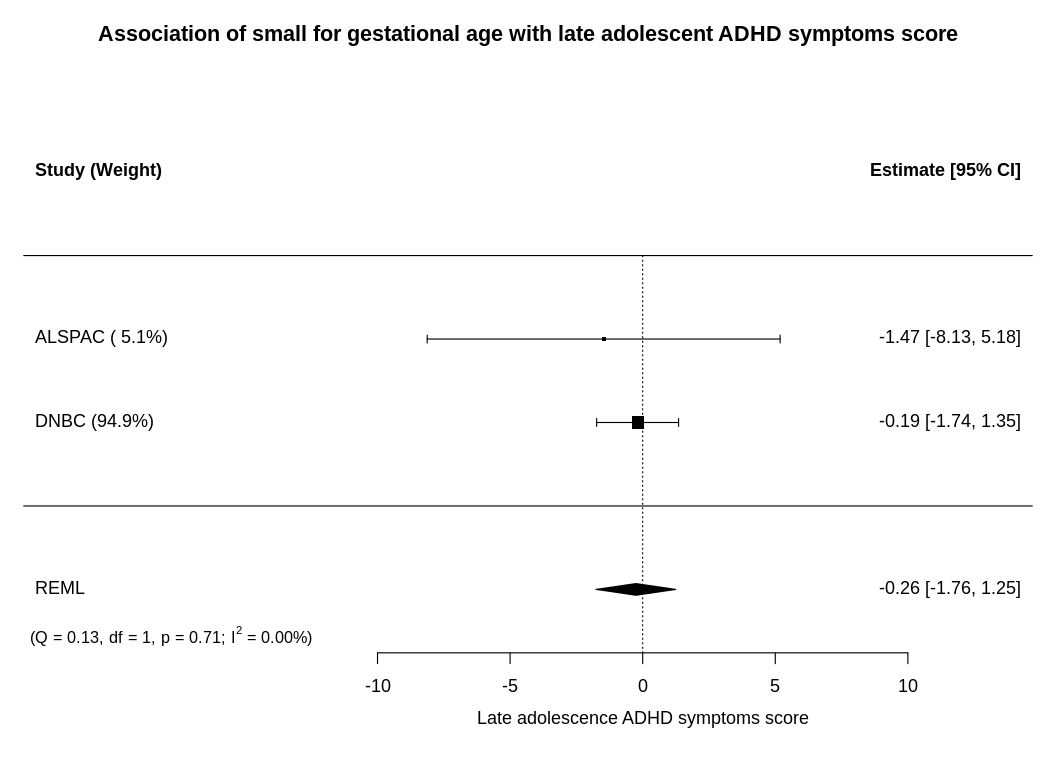


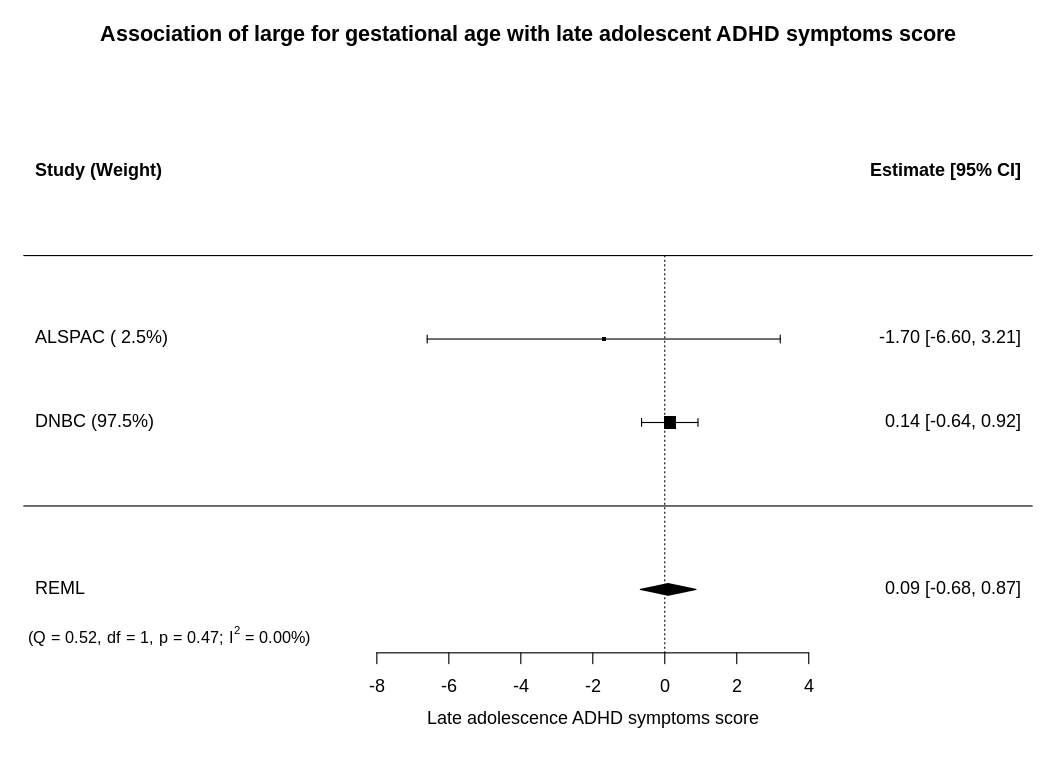


**Outcome: ASD symptoms**

**
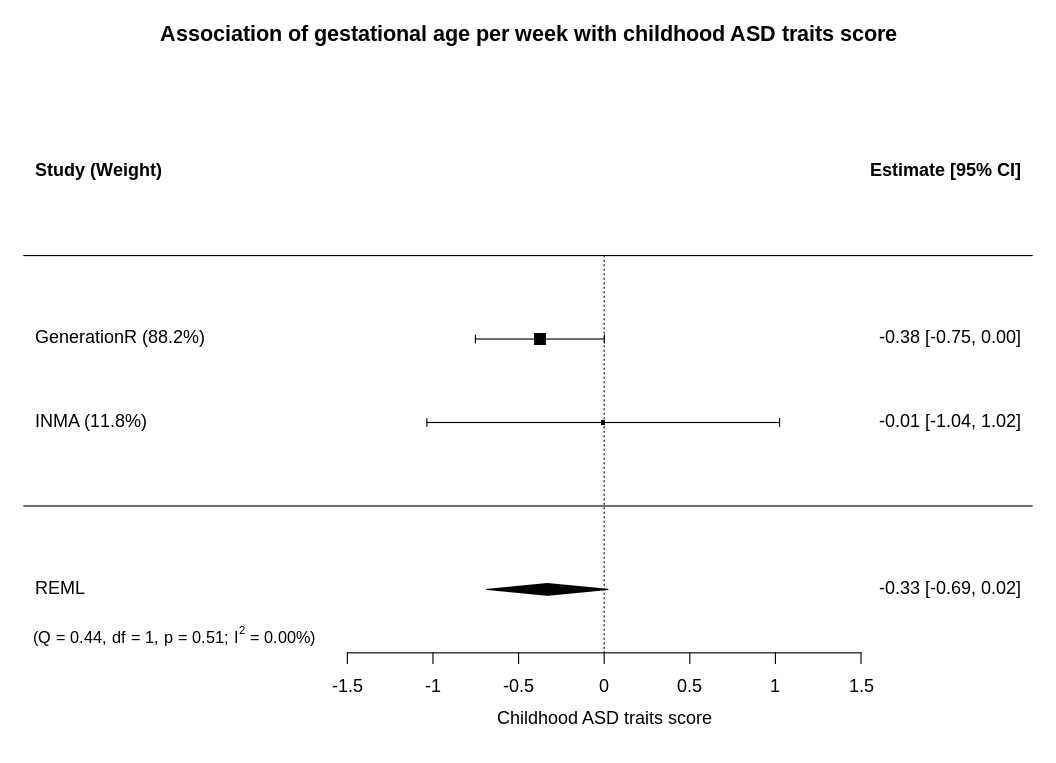
**

**
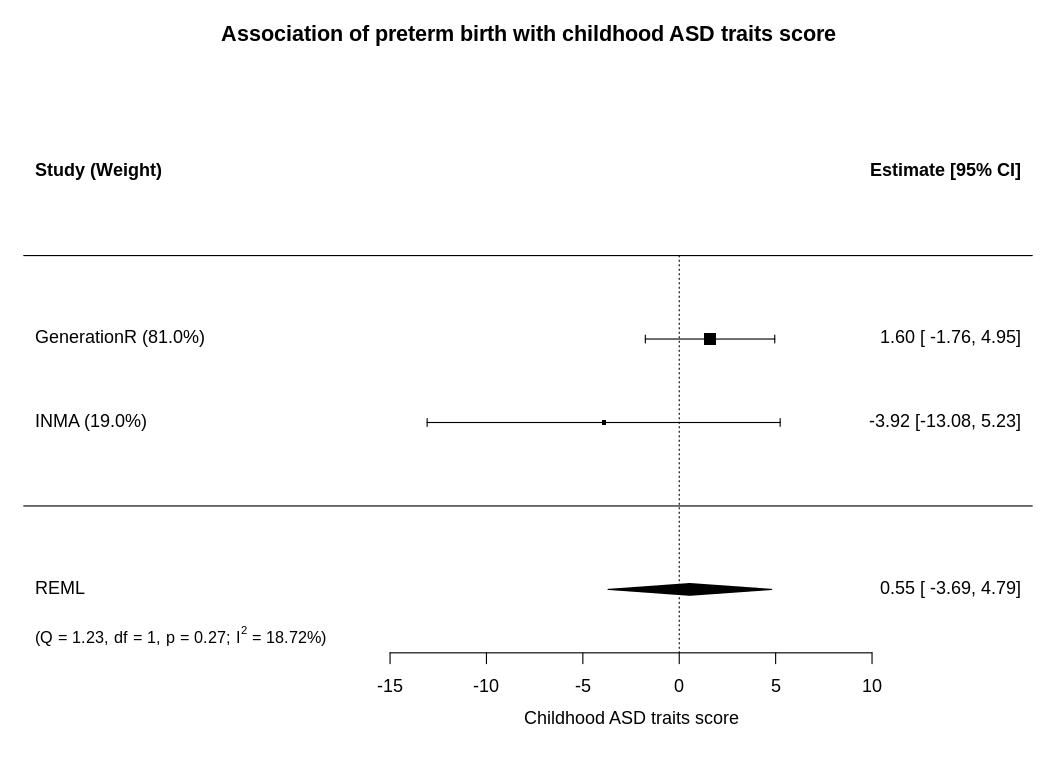
**

**
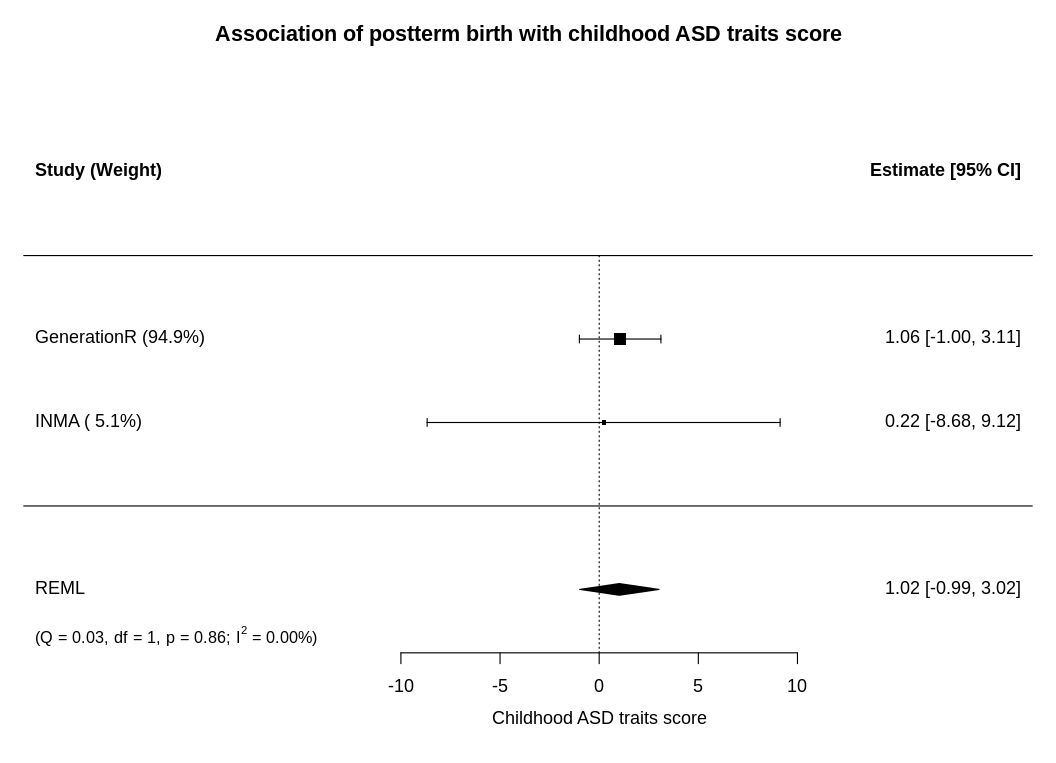
**

**
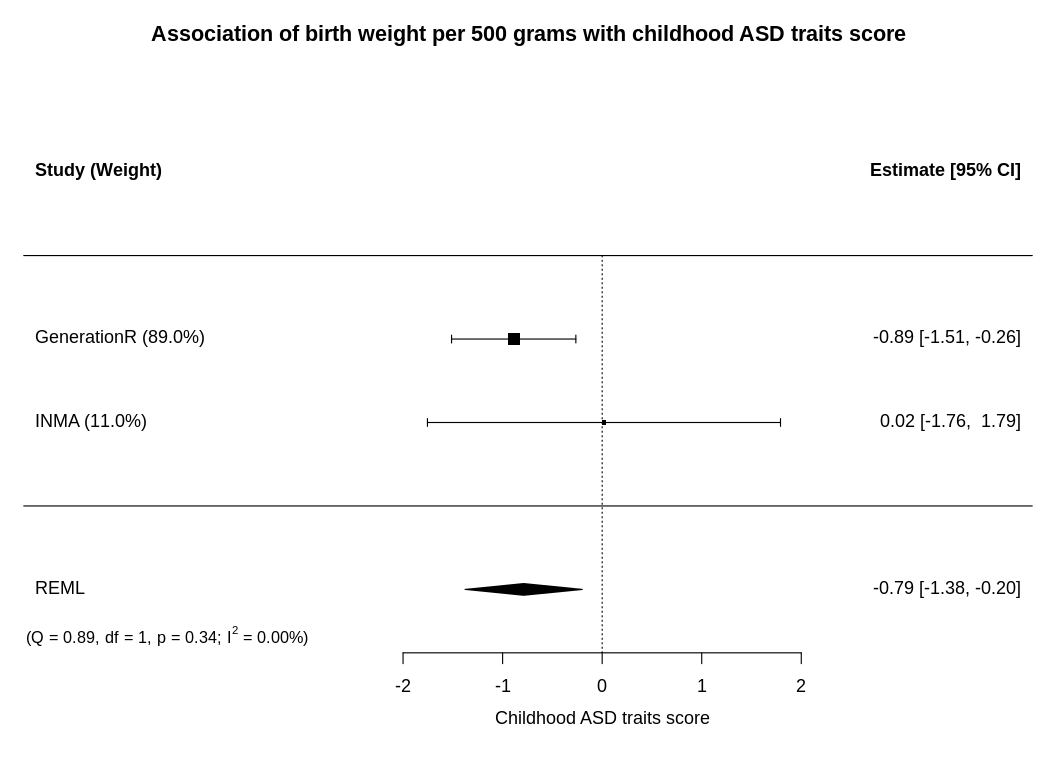
**

**
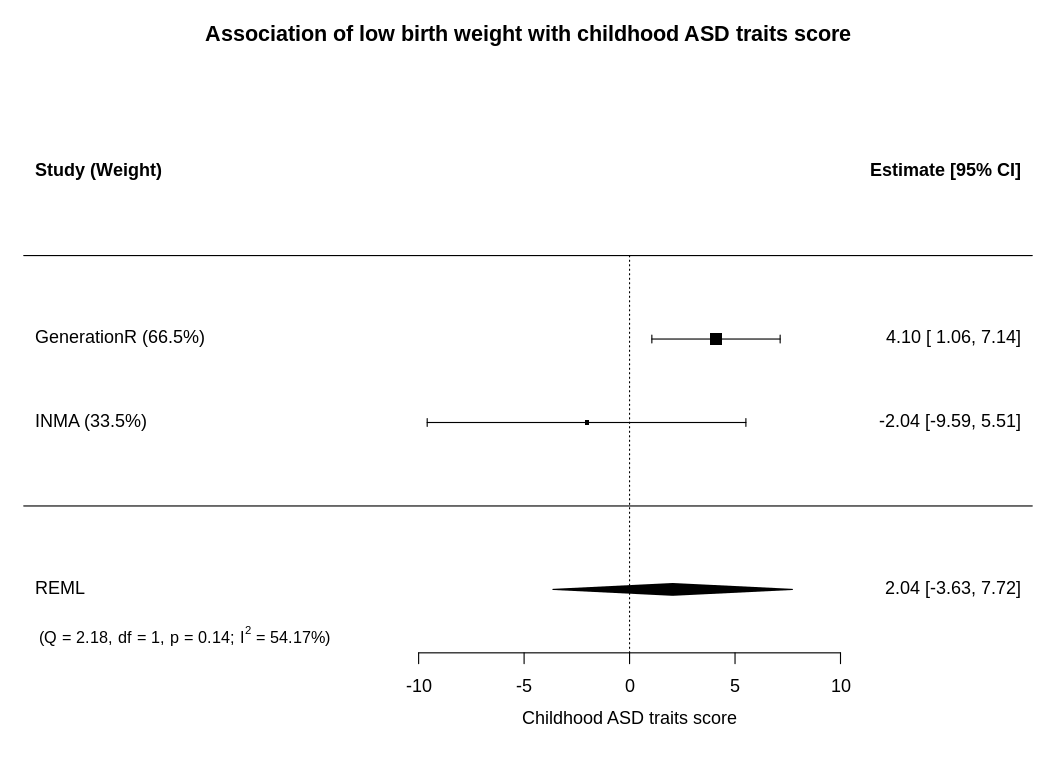
**

**
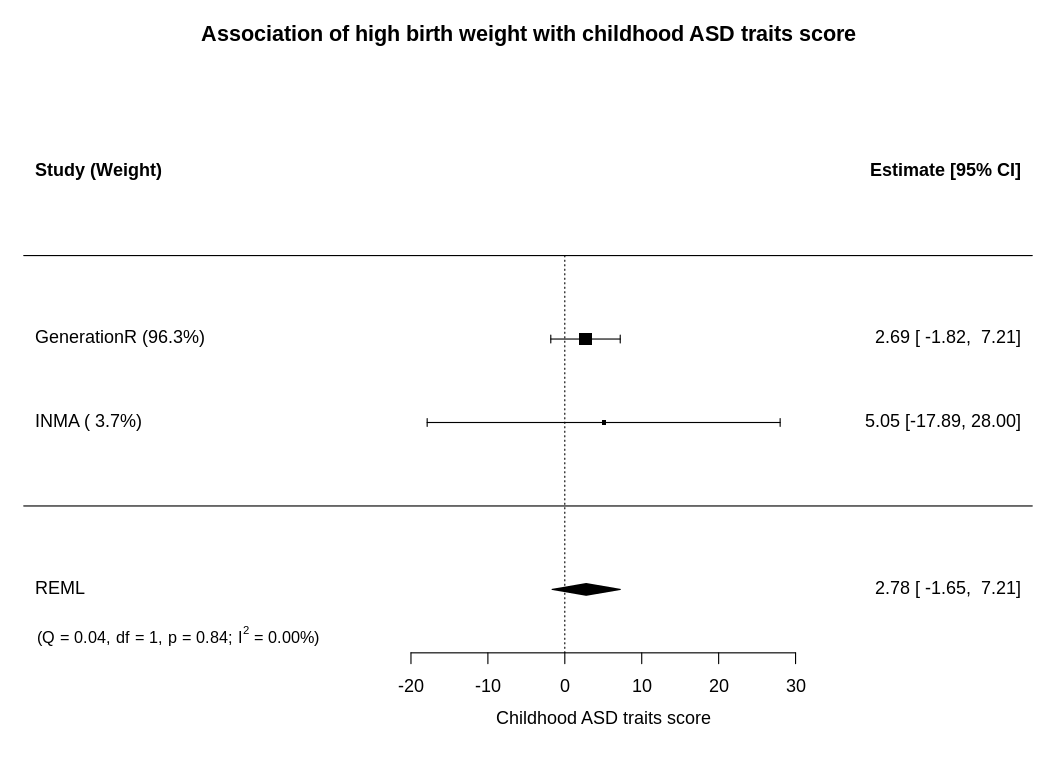
**

**
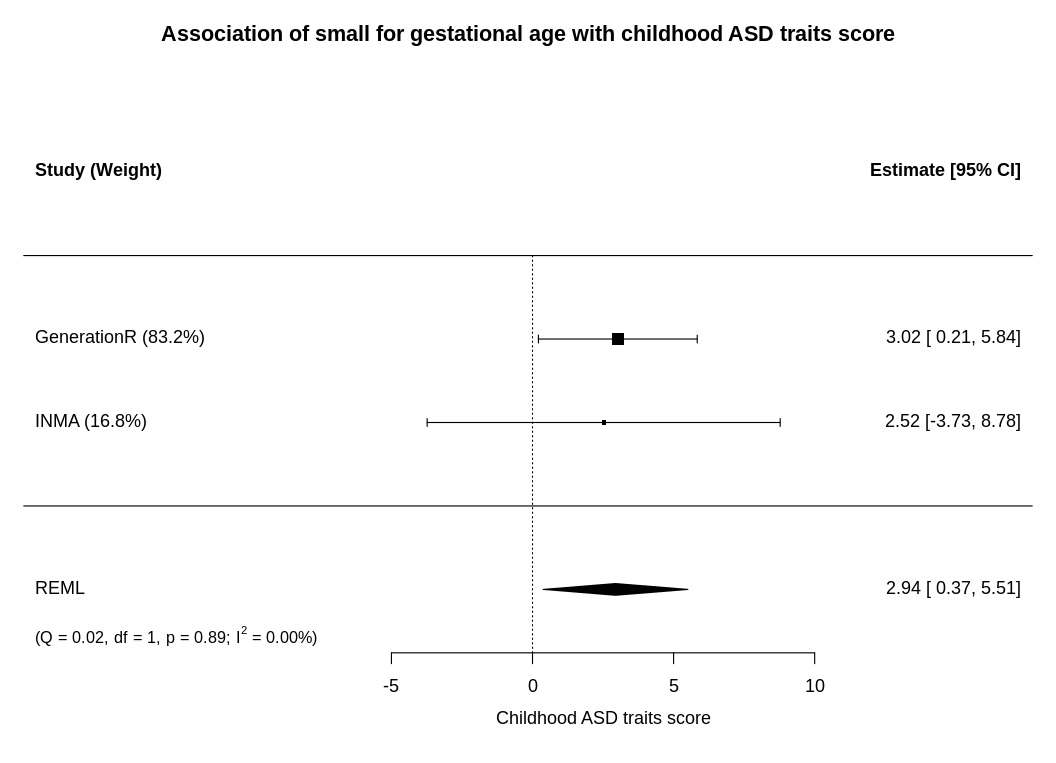
**

**
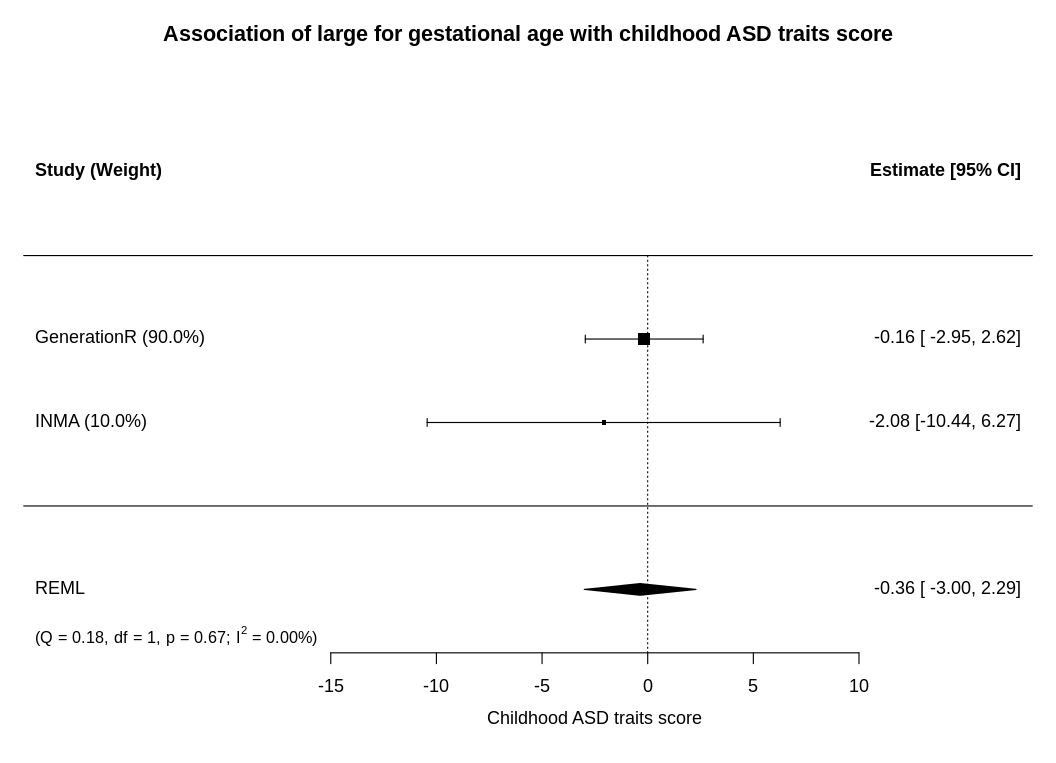
**

**Outcome: Non-verbal IQ score**

**
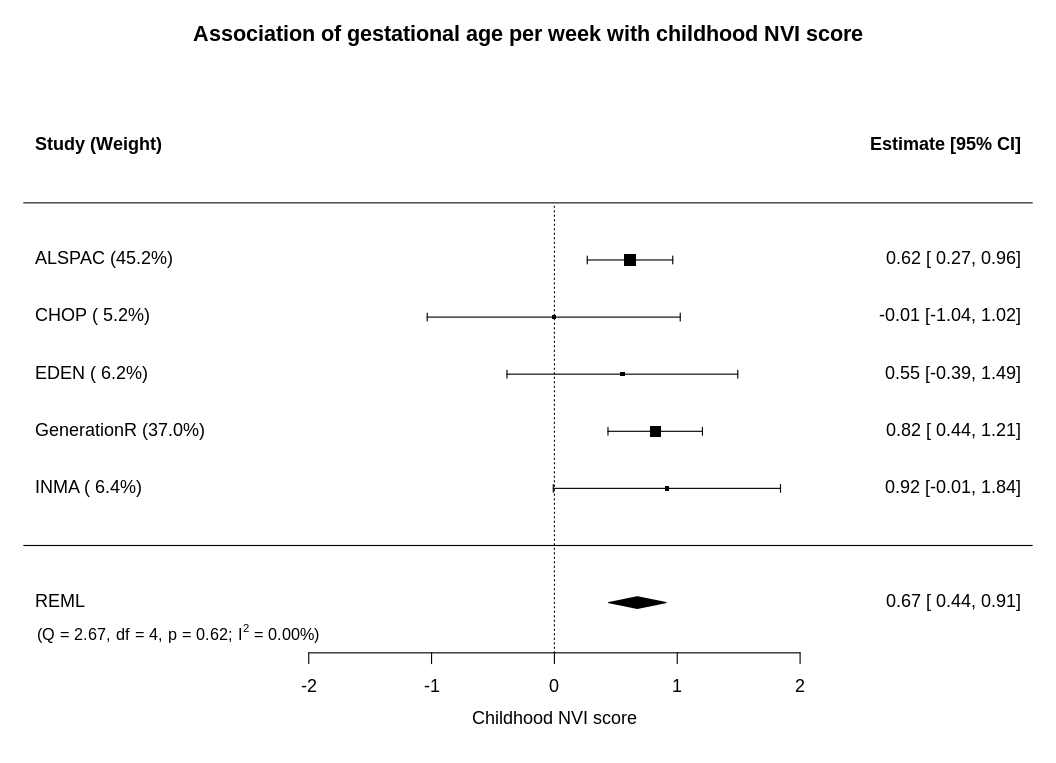
**

**
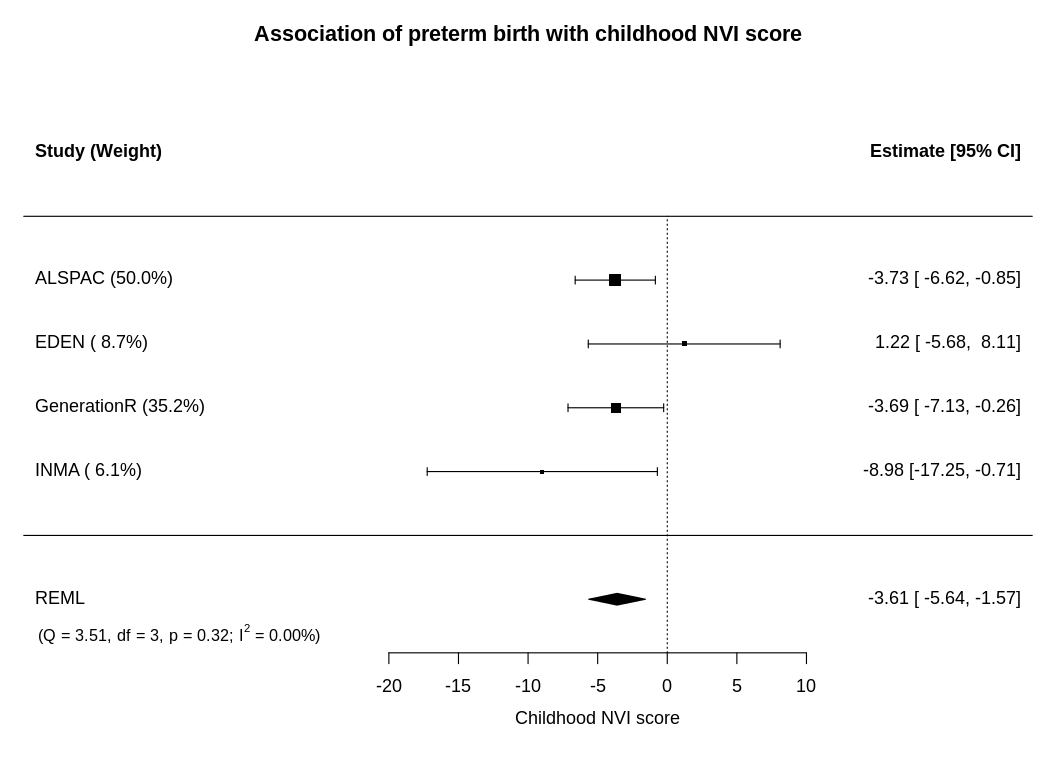
**

**
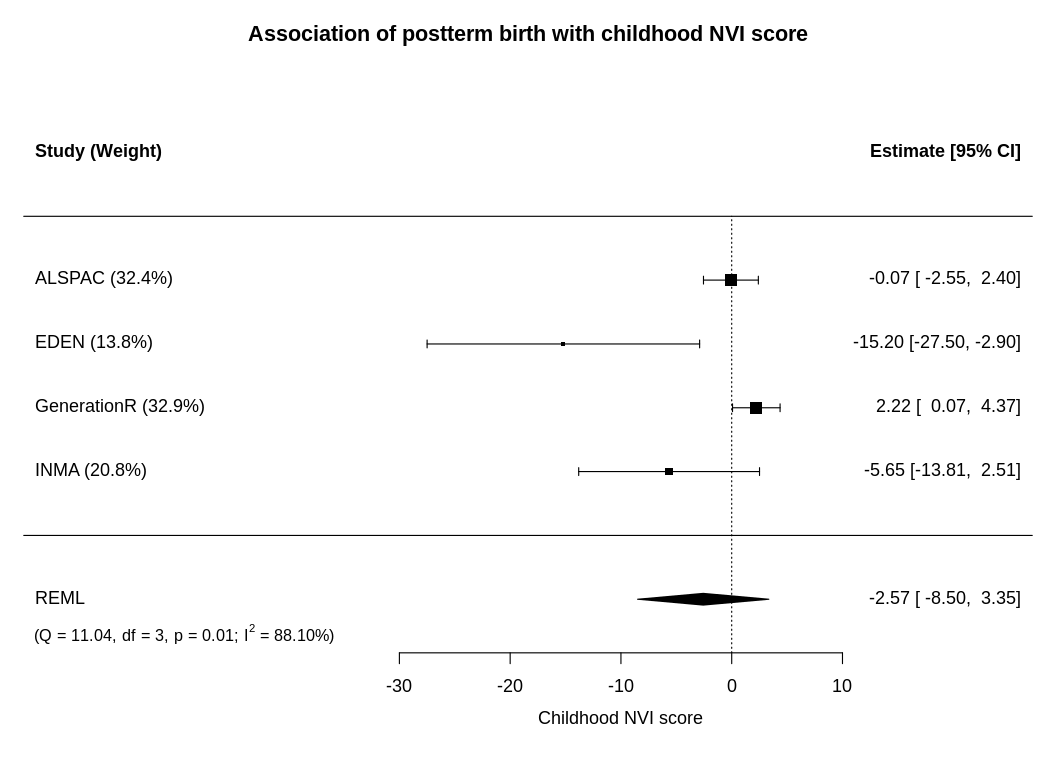
**

**
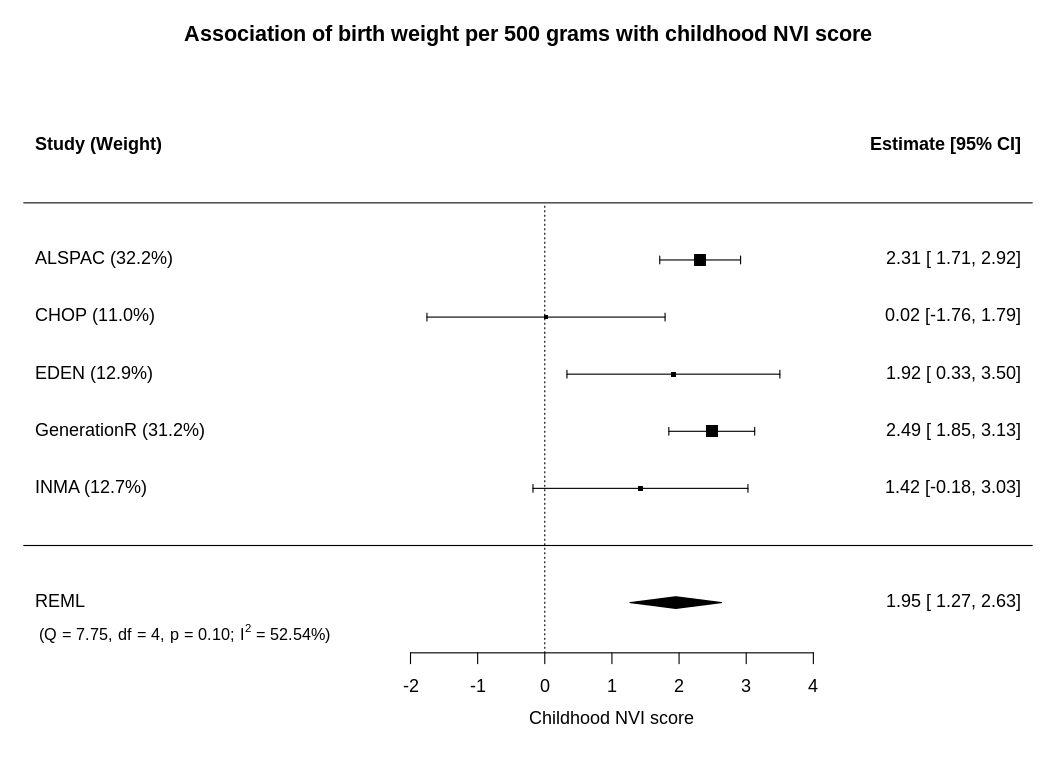
**

**
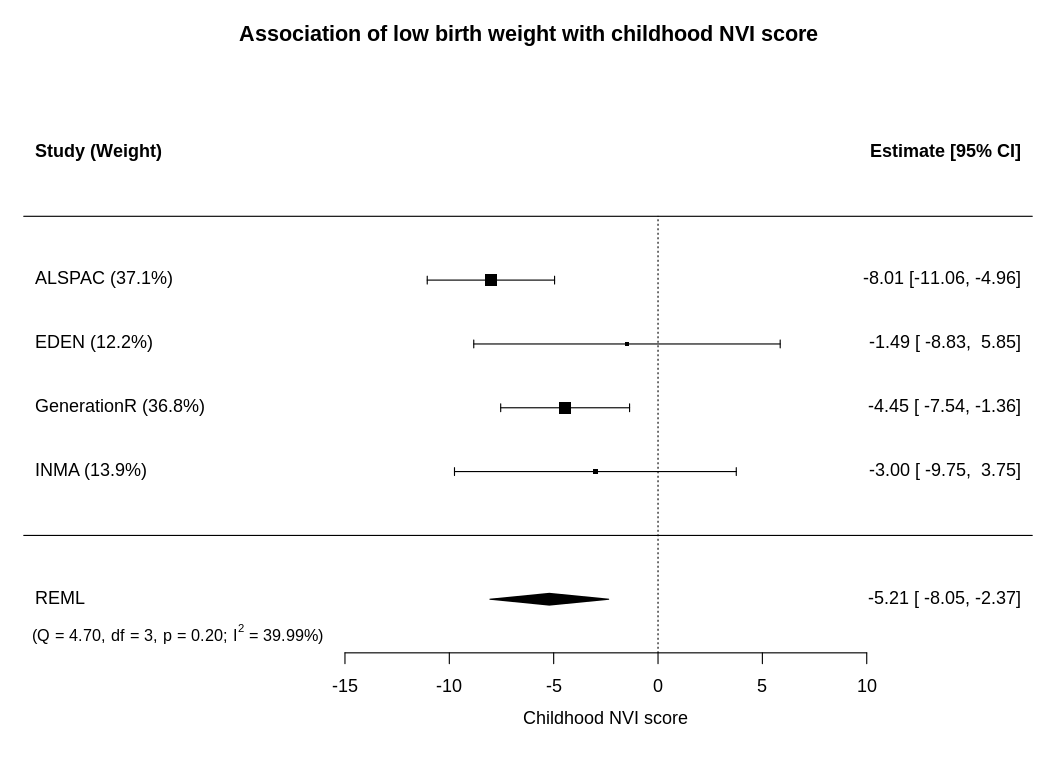
**

**
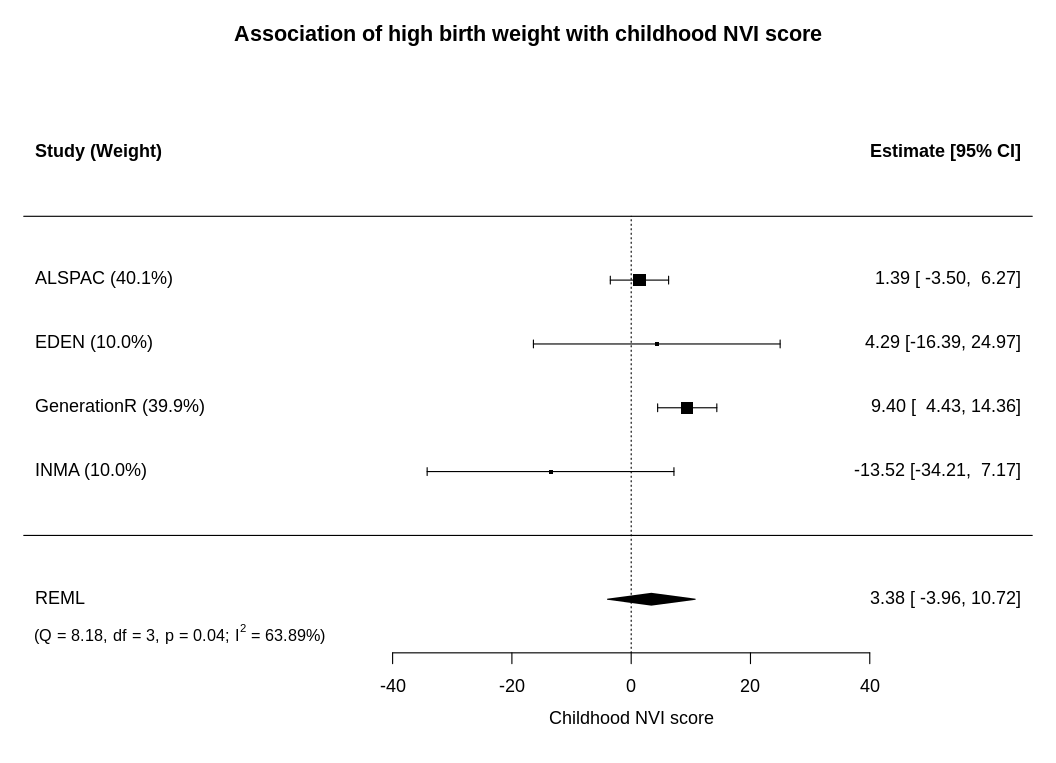
**

**
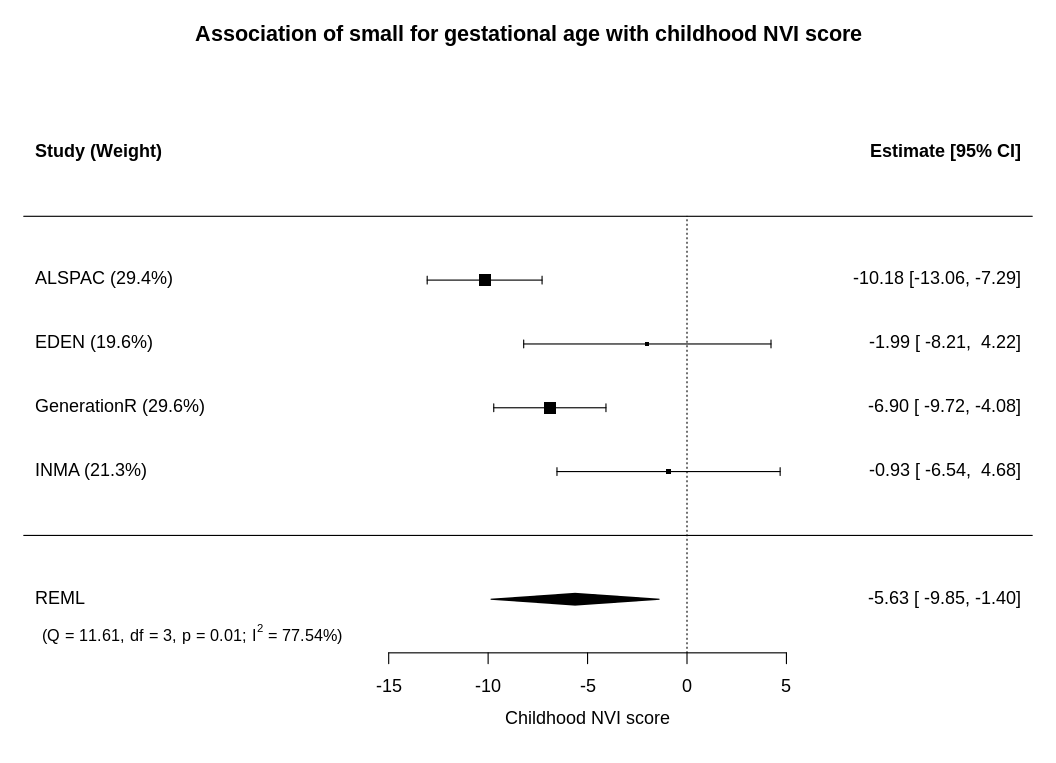
**

**
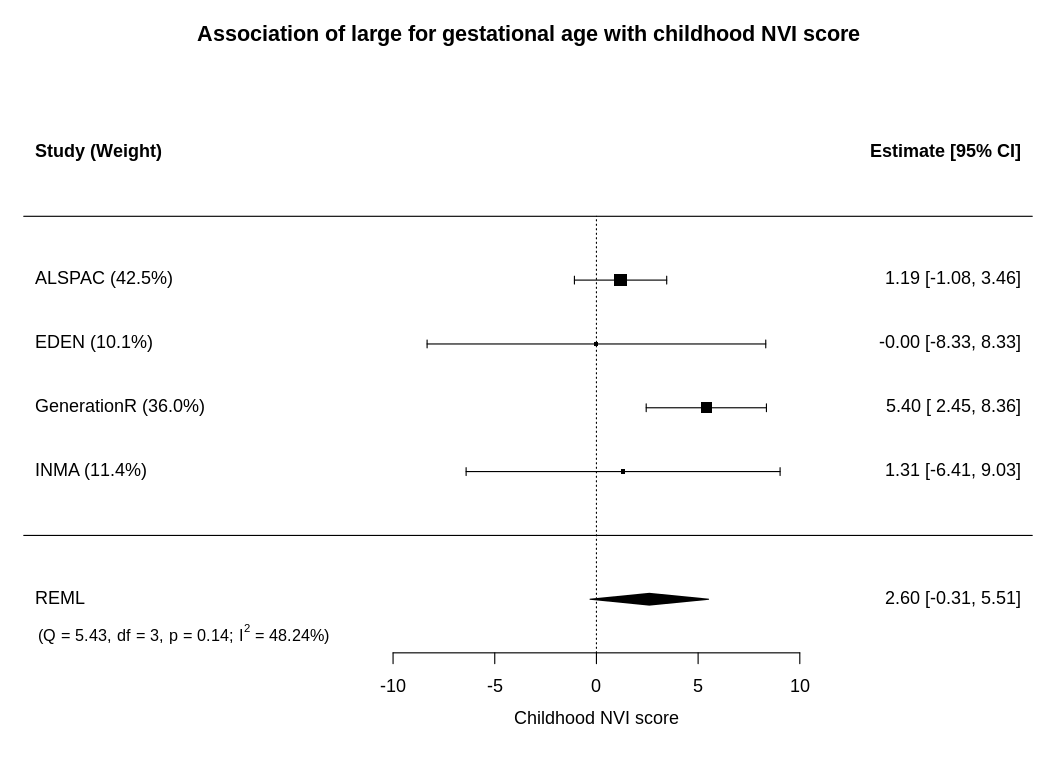
**

**STROBE Statement—checklist of items that should be included in reports of observational studies**

|  | Item No. | Recommendation | Page  No. | Relevant text from manuscript |
| --- | --- | --- | --- | --- |
| **Title and abstract** | 1 | (*a*) Indicate the study’s design with a commonly used term in the title or the abstract | 1 | Early-life Growth and Emotional, Behaviour and Cognitive Outcomes in Childhood and Adolescence in the EU Child Cohort Network: Individual Participant Data Meta-analysis of over 109,000 Individuals |
|  |  | (*b*) Provide in the abstract an informative and balanced summary of what was done and what was found | 4 | **Abstract**  **Background**  Foetal and infant development might be critical for cognitive outcomes and psychopathology later in life. We assessed the associations of birth characteristics and early life growth with behaviour and cognitive outcomes from childhood to adolescence.  **Methods**  We used harmonized data of 109,481 children from 8 European birth cohorts. Birth weight, gestational age, and body mass index (BMI) tertiles at the age of 2 years were used as the exposure variables. Outcomes included internalizing and externalizing problems and attention deficit hyperactivity disorder (ADHD), autism spectrum disorder (ASD) and non-verbal intelligence quotient (Non-verbal IQ) in childhood (4-10 years), early adolescence (11-16 years) and late adolescence (17-20 years). We used 1-stage individual participant data meta-analyses using generalized linear models.  **Findings**  A one-week older gestational age was associated with lower scores for internalizing problems (difference -0·48 (95% CI: -0·59, -0·37), externalizing problems (difference -0·34 (95% CI: -0·44, -0·23), and ADHD symptoms (difference -0·38 (95% CI: -0·49, -0·27), and with higher scores for non-verbal IQ (difference 0·65 (95% CI: 0·41, 0·89). As compared to term birth, preterm birth was associated with higher internalizing problems (difference 3·43 (95% CI: 2·52, 4·33) and externalizing problems (difference 2·31 (95% CI: 1·16, 3·46), ADHD symptoms (difference 4·15 (95% CI: 3·15, 5·16), ASD symptoms (difference 3·23 (95% CI: 0·37, 6·08) and lower non-verbal IQ (difference -5·44 (95% CI: -7·44, -3·44). Small size for gestational age at birth (SGA) in comparison with appropriate size for gestational age (AGA) was associated with higher ADHD symptoms (difference 4·88 (95% CI: 3·87, 5·90) and lower Non-verbal IQ (difference -7·02 (95% CI: -8·84, -5·21). Large size for gestational age at birth was associated with lower ADHD symptoms (difference -1·09 (95% CI: -1·73, 0·45) and higher non-verbal IQ (difference 2·47 (95% CI: 0·77, 4·18). Explorative analyses showed that as compared to children with an appropriate size for gestational age at birth and a normal BMI at the age of 2 years, children born SGA who remained small at 2 years had the lowest non-verbal IQ score (difference -8·14 percentiles (95% CI: -11·89, -4·39).  **Interpretation**  Both foetal and early childhood growth are associated with emotional, behavioural and cognitive outcomes throughout childhood and adolescence. Compensatory infant growth might partly attenuate the adverse effects of suboptimal foetal growth. Future studies are needed to identify the potential for optimizing mental health outcomes in new generations by improving early-life growth.  **Funding**  This project received funding from the European Union’s Horizon 2020 research and innovation programme (LIFECYCLE, grant agreement No 733206, 2016; EUCAN-Connect grant agreement No 824989; ATHLETE, grant agreement No 874583). |
| Introduction | | | |  |
| Background/rationale | 2 | Explain the scientific background and rationale for the investigation being reported | 7 | Central nervous system development starts in the very early stages of pregnancy and continues throughout life (1, 2). Disruptions in optimal foetal development are associated with cognitive and psychopathology outcomes in adulthood (3, 4). Previous studies have reported associations between low birth weight and preterm birth with an increased risk of internalizing and externalizing problems, attention-deficit hyperactivity disorder (ADHD), autism spectrum disorder (ASD) and lower intelligence quotient (IQ) (4-9). These associations might not be restricted to these extremes and be present across the full spectrum (9-12). Also, birth weight and gestational age at birth, may not be the causal factor per se, but may reflect foetal growth adaptations and are the starting point of infant development (9, 13). Children born preterm or with a low birth weight might have been exposed to different exposures and are known to follow different postnatal growth patterns as compared to their term born and normal birth weight peers (14, 15). It is not known whether compensatory infant growth and development leads to better neurodevelopmental outcomes in children born preterm or with a low birth weight. Large-scale studies examining the associations of birth weight combined with subsequent infant growth with behaviour and cognitive outcomes are scarce (16, 17). One study conducted in Belarus among more than 11,000 children showed that both increased birth weight and weight gain during infancy was associated with higher IQ (16). Identification of birth characteristics and early life growth patterns at risk for adverse long-term outcomes could identify windows of opportunity for novel strategies, such as monitoring behaviour and cognitive development in children born preterm or of low birth weight and strategies to optimize neurodevelopmental outcomes in children at risk. |
| Objectives | 3 | State specific objectives, including any prespecified hypotheses | 7-8 | We hypothesized that both adverse birth outcomes, birth weight as the end point of foetal development, and early childhood growth might be critical for cognitive and psychopathology later in life. Compensatory early childhood growth in children born after suboptimal foetal growth might be beneficial for emotional, behavioural and cognitive development in children (16, 18). We assessed the associations of birth characteristics and early-life growth with behaviour and cognitive outcomes in an individual participant data (IPD) meta-analysis among 109,481 children from 8 European cohort studies from pregnancy onwards. We used the infrastructure of the EU Child Cohort Network, which brings together data from pregnancy and childhood from European cohort studies and focused on the outcomes assessed in the age ranges of 4 to 20 years (19). |
| Methods | | | |  |
| Study design | 4 | Present key elements of study design early in the paper | 8-9 | **Study design and participating cohorts**  We included 109,481 mother-child pairs from eight European population-based birth cohorts collaborating in the EU Child Cohort Network established by the LifeCycle Project (19). Cohorts included the Avon Longitudinal Study of Parents and Children (ALSPAC, United Kingdom) (20-23), Born in Bradford (BiB United Kingdom) (24, 25), European Childhood Obesity Project Trial (CHOP; Germany, Belgium, Italy, Poland & Spain) (26, 27)), the Danish National Birth Cohort (DNBC, Denmark) (28), the French Longitudinal Study since Childhood (Etude Longitudinale Française depuis l’Enfance) (ELFE, France) (29), Etude sur les Déterminants de la santé de l’Enfant Nancy & Poitiers (EDEN‐Nancy & EDEN-Poitiers, France) (30), the Generation R Study (GenerationR, The Netherlands) (31), The INfancia y Medio Ambiente Project (INMA, Spain) (32). All cohorts participated in either the LifeCycle – EU Child Cohort Network or the Advancing Tools for Human Early Lifecourse Exposome Research and Translation (ATHLETE) Project (19, 33). The populations in the cohorts used in this study were recruited between 1991 and 2011. Information on the profile and design of each study is provided in **Text S1**. Cohorts were eligible for this study if they had the following exposure data: birth weight, gestational age at birth and weight and height at about 24 months of age, and information on either internalizing and externalizing problems, autism spectrum disorder (ASD), attention-deficit hyperactivity disorder (ADHD) or non-verbal intelligence quotient (Non-verbal IQ) in children or adolescents aged 4-20 years. The flowchart of the current study population is given in **Figure S1 and S2**. All cohorts received approval from their local institutional review boards and all study participants gave written informed consent to participate in the respective cohorts and secondary data analyses. Data were harmonized across these cohorts. The harmonized data were kept within each institution and analysed through the R-based federated data analysis platform DataSHIELD (34-36). This study followed the Strengthening the Reporting of Observational Studies in Epidemiology (STROBE) reporting guideline (37). |
| Setting | 5 | Describe the setting, locations, and relevant dates, including periods of recruitment, exposure, follow-up, and data collection | 8-9 | See text given at Methods - 4 |
| Participants | 6 | (*a*) *Cohort study*—Give the eligibility criteria, and the sources and methods of selection of participants. Describe methods of follow-up  *Case-control study*—Give the eligibility criteria, and the sources and methods of case ascertainment and control selection. Give the rationale for the choice of cases and controls  *Cross-sectional study*—Give the eligibility criteria, and the sources and methods of selection of participants | 8-9 | See text given at Methods - 4 |
|  |  | (*b*) *Cohort study*—For matched studies, give matching criteria and number of exposed and unexposed  *Case-control study*—For matched studies, give matching criteria and the number of controls per case |  | Not applicable |
| Variables | 7 | Clearly define all outcomes, exposures, predictors, potential confounders, and effect modifiers. Give diagnostic criteria, if applicable | 9-10 | **Birth characteristics and early childhood growth measures**  Birth characteristics were collected from midwife and hospital records in all cohorts. We used gestational age continuous (weeks) and categorized as preterm (<37 weeks), term (37-42 weeks) and post term (>42 weeks) birth. We used birth weight continuous (per 500 grams) and categorized as low birth weight (<2,500 grams), normal birth weight (2,500-4,500 grams) and high birth weight (>4,500 grams). Size for gestational age was defined using the WHO foetal growth charts (38). Small for gestational age (SGA) was defined as weight being below the 5^th^ percentile, appropriate size for gestational age (AGA) was defined as weight being between the 5^th^ and the 95^th^ percentile and large for gestational age (LGA) was defined as weight being above the 95^th^ percentile. Early childhood weight and height was assessed by using an age interval between 18 to 30 months, in which the measurement closest to 24 months was selected, and body mass index (BMI) was calculated (kg/m^2^). Because age intervals were constructed and healthy BMI ranges differ per age, we used BMI tertiles in our population, defining the lowest tertile as low BMI, the middle tertile as normal BMI and the third tertile as high BMI.  **Emotional, behavioural and cognitive outcomes**  Information about the emotional, behaviour and cognitive outcomes in the EU Child Cohort Network has been described previously (39). Internalizing and externalizing problems, ADHD symptoms, ASD symptoms and Non-verbal IQ were assessed in the cohorts at different ages and with different instruments. Briefly, internalizing and externalizing problems were measured by using the Strengths and Difficulties Questionnaire (SDQ) (40) or the Child Behavior Checklist (CBCL) (41) for both instruments higher scores indicate more emotional and behavioural problems. Both are parent-reported, except for in DNBC cohort for late adolescents. ADHD symptoms were measured by using the hyperactivity subscale of the SDQ (40), the Conner’s Parent Rating Scale-Revised Short Form (CPRS-R:S) (42) or the Diagnostic and Statistical Manual of Mental Disorders IV (DSM-IV) (43), but only in INMA cohort. The SDQ and the CPRS-R:S are both parent-reported, whereas the DSM-IV questionnaire was filled out by teachers. For all instruments higher scores, indicate more ADHD symptoms. ASD symptoms were measured by using the Social Responsiveness Scale (SRS; GENR) (44) or the Childhood Autism Spectrum Test (CAST; INMA)(45). For both tests, higher scores indicate more ASD symptoms. ASD symptoms were assessed with either parent-reported questionnaires or an interview conducted at the research facility. Last, non-verbal IQ was mainly assessed using the Snijders-Oomen Niet-verbale intelligentie Test- Revisie (SON-R 2.5–7) (46) and the [Wechsler Preschool and Primary Scale of Intelligence](https://www.sciencedirect.com/topics/medicine-and-dentistry/wechsler-preschool-and-primary-scale-of-intelligence) Third Edition (WPPSI-III)(47). A global non-verbal IQ can be established using these tests. The tests were administered during a visit to the research facility and either handwritten or computer based. In each separate cohort at each age point internal percentiles were constructed as part of the EU Child Cohort Network data harmonization (19, 39). A detailed description of the instruments used in the cohorts is given in **Text S2.** A detailed description of the instruments used in the cohorts is given in **Text S2.** In each separate cohort at each age point internal percentiles were constructed as part of the EU Child Cohort Network data harmonization (19). **Table S2 and S3** show the descriptive statistics and distribution of emotional, behaviour and cognitive outcomes in each cohort. We constructed age intervals (childhood: 4·0-10·9 years; early adolescence: 11·0-16·9 years; and late adolescence: 17·0-20·0 years) for internalizing problems, externalizing problems and for ADHD symptoms. ASD symptoms and Non-verbal IQ scores were only available in childhood (4·0-10·9 years). When more than one assessments were available in one age interval, we used the assessments at the oldest age. |
| Data sources/ measurement | 8* | For each variable of interest, give sources of data and details of methods of assessment (measurement). Describe comparability of assessment methods if there is more than one group | 9-10 | See text given at Variables - 7 |
| Bias | 9 | Describe any efforts to address potential sources of bias | 8-10 | Full methods |
| Study size | 10 | Explain how the study size was arrived at | 8 | See text given Study design – 4  And the flowcharts given in Figure S1 and S2 of the supplementary material. |

| Quantitative variables | 11 | Explain how quantitative variables were handled in the analyses. If applicable, describe which groupings were chosen and why | 11-12 | **Statistical analysis**  First, we described the participant characteristics per cohort and for all cohorts combined. Second, we conducted a 1-stage individual participant data meta-analysis using generalized linear models to analyse the associations of birth characteristics and the behaviour and cognitive outcomes in different age windows. Main exposures included birth characteristics included gestational age at birth and birth weight both continuously and categorized. Outcomes included internalizing problems, externalizing problems and ADHD symptoms in childhood, early adolescence, and late adolescence, and ASD symptoms and Non-verbal IQ scores in childhood. Third, we assessed the associations of nine combinations of different gestational age adjusted sizes at birth (SGA, AGA, LGA) and early childhood BMI tertiles as growth patterns, with behaviour and cognitive outcomes. To correct for multiple testing, we used the Bonferroni correction method taking account for five outcomes and specified multiple-test corrected p-values as <0·01. CHOP was not included in the categorical analyses of birth weight and gestational age, since preterm born and low birth weight children were specifically excluded in this cohort. DataSHIELD withholds results if the number of data in a cohort is less than 30 for data-security reasons. As a result, GenerationR and INMA were not included in the birth weight categories analyses for early adolescence internalizing and externalizing problems. When constructing the nine growth patterns some cohorts had few or no data in any of the nine categories, therefore the analyses could only be performed in childhood for the internalizing and externalizing problems, ADHD symptoms and Non-verbal IQ score. All analysis were performed using DataSHIELD dsBaseClient package version 6·3·0 (34-36) |
| --- | --- | --- | --- | --- |
| Statistical methods | 12 | (*a*) Describe all statistical methods, including those used to control for confounding | 11 | All models were adjusted for sex, age at outcome measurement and cohort in the basic models, and additionally for maternal age, education, pre-pregnancy BMI, parity, and smoking during pregnancy in the main model. We did not adjust for ethnicity since most cohorts had one predominant ethnicity group. |
|  |  | (*b*) Describe any methods used to examine subgroups and interactions | 11 | We assessed the statistical interaction between size at birth and early childhood BMI for these associations. |
|  |  | (*c*) Explain how missing data were addressed | 12 | Missing values for covariates were imputed using multiple imputation on cohort level by the fully conditional specification method, and pooled results from 5 imputed datasets were reported. A detailed description is given in **Text S3**. The percentage of missing values ranged from 0 to 27·6% (DNBC, maternal education level). |
|  |  | (*d*) *Cohort study*—If applicable, explain how loss to follow-up was addressed  *Case-control study*—If applicable, explain how matching of cases and controls was addressed  *Cross-sectional study*—If applicable, describe analytical methods taking account of sampling strategy | 12 | See text given at Quantitative variables - 11 |
|  |  | (*e*) Describe any sensitivity analyses | 12 | As a sensitivity analysis, we performed a 2-stage individual participant meta-analysis of the main models, by calculating the effect estimates in each cohort separately. Subsequently the combined estimate across all cohorts was calculated by random effects meta-analysis using a restricted maximum likelihood estimation (REML). |
| Results | | | | |
| Participants | 13* | (a) Report numbers of individuals at each stage of study—eg numbers potentially eligible, examined for eligibility, confirmed eligible, included in the study, completing follow-up, and analysed | supplement | See flowchart Figure S1 and S2 in Supplementary material |
|  |  | (b) Give reasons for non-participation at each stage | supplement | See flowchart Figure S1 and S2 in Supplementary material |
|  |  | (c) Consider use of a flow diagram | supplement | See flowchart Figure S1 and S2 in Supplementary material |
| Descriptive data | 14* | (a) Give characteristics of study participants (eg demographic, clinical, social) and information on exposures and potential confounders | 24-26 | Table 1 and Table 2 |
|  |  | (b) Indicate number of participants with missing data for each variable of interest | 24-26 | Table 1 and Table 2 |
|  |  | (c) *Cohort study*—Summarise follow-up time (eg, average and total amount) | supplement | Table S1-S3 |
| Outcome data | 15* | *Cohort study*—Report numbers of outcome events or summary measures over time | 24-26 and supplement | Table 1 and Table 2. Table S1-S3. |
|  |  | *Case-control study—*Report numbers in each exposure category, or summary measures of exposure |  | Not applicable |
|  |  | *Cross-sectional study—*Report numbers of outcome events or summary measures |  | Not applicable |
| Main results | 16 | (*a*) Give unadjusted estimates and, if applicable, confounder-adjusted estimates and their precision (eg, 95% confidence interval). Make clear which confounders were adjusted for and why they were included | 11 and 13-14 | All models were adjusted for sex, age at outcome measurement and cohort in the basic models, and additionally for maternal age, education, pre-pregnancy BMI, parity, and smoking during pregnancy in the main model. We did not adjust for ethnicity since most cohorts had one predominant ethnicity group.  **Birth outcomes, emotional, behaviour and cognitive outcomes**  **Table 3** shows that one week older gestational age at birth and higher birth weight were associated with lower scores for internalizing and externalizing problems in childhood and early adolescence (all p-values <0·01). In late adolescence, the associations of gestational age at birth with externalizing problems were reversed, suggesting that one week older gestational age at birth was associated with higher externalizing problems symptoms (p-value <0·01). As compared to children born term, those born preterm had increased scores for internalizing behaviour in childhood (3·43 percentiles ( 95% Confidence Interval (CI) 2·52, 4·33)), early adolescence (1·77 percentiles (95% CI 0·57, 2·96)), and late adolescence (1·20 percentiles (95% CI 0·10, 2·30), and increased scores for externalizing behaviour in childhood (2·28 percentiles (95% CI 1·43, 3·13) and early adolescence (2·31 percentiles (95% CI 1·16, 3·46). This latter association reversed in late adolescence. The associations between low birth weight with internalizing and externalizing scores tended to be similar as for preterm birth suggesting that as compared to normal birth weight, low birth weight is associated with higher scores for internalizing and externalizing behaviour in childhood and early adolescence, whereas in late adolescence these association were absent or inversed.  **Table 4** shows that one week older gestational age at birth and higher birth weight were associated with lower scores for ADHD symptoms in childhood and early adolescence, and with a higher Non-verbal IQ in childhood (all p-values <0·01). Higher birth weight was also associated with lower ASD symptoms in childhood (p-value <0·01). In late adolescence the associations of gestational age at birth with ADHD symptoms were reversed, suggesting that one week older gestational age at birth was associated with higher ADHD symptoms (p-value <0·01). As compared to children born term, those born preterm had increased scores for ADHD symptoms in childhood (2·51 percentiles ( 95% CI 1·61, 3·40)) and early adolescence (2·33 percentiles (95% CI 1·16, 3·50)). This association reversed in late adolescence. The associations between low birth weight with ADHD symptoms tended to be similar as for preterm birth. Not preterm birth, but low birth weight was associated with ASD symptoms (3·23 percentiles (95% CI 0·37, 6·08). Preterm birth and low birth weight were also associated with a -3·41 percentiles (95% CI: -5·46, -1·36) and -5·44 percentiles (95% CI: -7·44, -3·44) lower Non-verbal IQ in childhood respectively. Although not consistent, tendencies for opposite associations were observed for high birth weight. The corresponding basic models showed similar associations and are shown in **Tables S4 and S5.**  **Size at birth, childhood BMI and emotional, behaviour and cognitive outcomes**  **Table 5** shows that as compared to children born AGA, those born SGA had higher scores for internalizing problems, externalizing problems and ADHD symptoms, and lower scores for Non-verbal IQ in childhood (all p-values <0·01). Although not consistent, tendencies for opposite associations were observed for LGA born children. The corresponding basic models showed similar associations and are shown in **Table S6.**  **Table 6** shows the statistical interaction for the associations between size at birth and BMI in early childhood in relation to emotional, behaviour and cognitive outcomes was not significant for any association. The results for the explorative analysis showed that when combining size at birth with BMI in early childhood, as compared to children born AGA with a normal early childhood BMI, those born LGA and a normal BMI at the age of 2 years had lower scores for internalizing problems (-1·40 (95% CI: -2·38, -0·42). Children born SGA who remained in the lowest tertile of BMI at 2 years had the lowest Non-verbal IQ score (-8·14 percentiles (95% CI: -11·89, -4·39). Children within the lowest tertile of BMI at 2 years had, independent of their size at birth, increased scores for ADHD (all p-values <0·05), however these associations attenuated into non-significance after correction for multiple testing was applied. The corresponding basic models showed similar associations and are shown in **Table S7.** |
|  |  | (*b*) Report category boundaries when continuous variables were categorized | 13-14 | See text given at Main results - 16 |
|  |  | (*c*) If relevant, consider translating estimates of relative risk into absolute risk for a meaningful time period |  | Not applicable |

| Other analyses | 17 | Report other analyses done—eg analyses of subgroups and interactions, and sensitivity analyses | 14-15 | **Sensitivity analyses**  **Tables S8-S11** show the results of a complete-case sensitivity analysis. The direction and magnitude of effect estimates were consistent with the main analysis. **Figure S4** shows the results of sensitivity analyses from 2-stage individual participant data meta-analysis. The direction of the associations was consistent with the observations of the 1-stage analysis, but the results were less precise. |
| --- | --- | --- | --- | --- |
| Discussion | | | | |
| Key results | 18 | Summarise key results with reference to study objectives | 15 | In this large scale European individual participant data meta-analysis, we observed that older gestational age at birth and higher birth weight were associated with lower scores for internalizing problems, externalizing problems and ADHD in childhood and early adolescence and with a higher non-verbal intelligence score in childhood. The associations tended to be weaker or even inversed during late adolescence. Furthermore, children born SGA with a BMI in the lowest tertile at 2 years of age had decreased Non-verbal IQ in childhood as compared to children born AGA with a normal BMI at 2 years of age. Our findings suggest foetal and early childhood growth have both effects on behavioural and cognitive outcomes in later life. Also, the adverse effects of suboptimal foetal growth, might be partly attenuated by compensatory early childhood growth. |
| Limitations | 19 | Discuss limitations of the study, taking into account sources of potential bias or imprecision. Discuss both direction and magnitude of any potential bias | 18 | Strengths of this study are the large sample size available in the infrastructure of the EU Child Cohort Network enabled us to use harmonized data from different pregnancy and childhood cohort studies. This approach enables assessment of the effect estimates in different subgroups of birth characteristics and childhood BMI, and assessment of the consistency of the associations in different populations and at different time points. Another strength is we assessed full range scores for emotional, behavioural and cognitive outcomes. This study also has some limitations, though the sample sizes are large, there is a discrepancy in sample size between the different cohorts. Data availability differed greatly between cohorts. Ideally, we would have used sex and gestational age standardized z scores for birth weight, and length standardized z-scores for weight during infancy. However, these data were not available in the in the EU Child Cohort Network database. Therefore, we adjusted the analyses focused on the associations of birth weight categories and growth patterns with childhood outcomes for sex and gestational age at birth. The emotional, behavioural and cognitive outcomes were measured using different instruments and although harmonized, are not completely homogenous. We could not take account for different respondents of the emotional, behaviour and cognitive outcomes questionnaires because this information was not completely available in the EU Child Cohort Network. Also, covariates were present in varying degrees, but missing covariates of interest were imputed. Residual confounding by various lifestyle, diet, sociodemographic or ethnic related factors cannot be excluded. Also, we did not have detailed information about other health conditions or comorbidities, which could affect physical, behavioural or cognitive development. Our study was conducted in relatively healthy children. Due to observational nature of the study, no conclusions can be drawn on the causality of the reported associations. Finally, the effect estimates may be different in clinical populations and care should be taken when extrapolating our findings to other populations. |
| Interpretation | 20 | Give a cautious overall interpretation of results considering objectives, limitations, multiplicity of analyses, results from similar studies, and other relevant evidence | 15-18 | In this large scale European individual participant data meta-analysis, we observed that older gestational age at birth and higher birth weight were associated with lower scores for internalizing problems, externalizing problems and ADHD in childhood and early adolescence and with a higher non-verbal intelligence score in childhood. The associations tended to be weaker or even inversed during late adolescence. Furthermore, children born SGA with a BMI in the lowest tertile at 2 years of age had decreased Non-verbal IQ in childhood as compared to children born AGA with a normal BMI at 2 years of age. Our findings suggest foetal and early childhood growth have both effects on behavioural and cognitive outcomes in later life. Also, the adverse effects of suboptimal foetal growth, might be partly attenuated by compensatory early childhood growth.  Results from a previous large meta-analysis, showed that in 81% of studies, children born premature had higher internalizing and externalizing problems and a higher risk of ADHD (5). Another review reported that children born preterm have a 3-4 times higher risk of psychiatric disorders in childhood (7). A study conducted in Sweden among 546,000 sibling pairs examined the association of birth weight and psychiatric disorders and found higher rates of anxiety, externalizing problems and ADHD with decreasing birth weight and that by an increase of 1 kg in birth weight the risk of general and specific neurodevelopmental factors was reduced significantly (4). Using Norwegian health registries, a population-based study among 1·8 million term born children, reported that birth weight below 3,500 grams was associated with more behavioural disorders including ADHD (3).  Results from our current study showed that both internalizing problems, externalizing problems and ADHD symptoms were increased in children or young adolescents who were born preterm or with low birth weight. These associations tended to be weaker or even inversed during late adolescence. Previous studies have shown that the increased behavioural problems of preterm born children in childhood do not seem to track into adulthood. Compared to their term born peers, preterm born adolescents were less likely to drink alcohol or use drugs and undertook in less risk full behaviour (48, 49). A potential explanation for the weaker or inversed associations in late adolescence could be maturation and puberty effects, that lead temporarily or permanent to other effects. Results from previous studies showed that adults born preterm might have slightly altered personalities in regard to their term born peers (50). Also from early adolescence onwards, agreeableness and conscientiousness tends to decrease (51). Another explanation for the difference in direction of the association might be that we only had data regarding externalizing problems and ADHD symptoms in ALSPAC and DNBC. The instrument used to measure internalizing, externalizing and ADHD symptoms in late adolescence was in DNBC cohort, as opposed to all other cohorts, self-reported. Previous studies have shown there can be discrepancies in parent-reported and self-reported assessment of emotional and behavioural problems (52, 53). Though, these studies suggest that when children self-report, they are more likely to report more problems. However, we observed less problems in late adolescence.  Preterm birth and low birth weight have previously been associated with higher ASD (3, 4, 7, 54). We observed that higher birth weight was associated with lower ASD symptoms. The lack of association for preterm birth might also be explained by lower numbers since ASD was only available in two cohorts, that have both a rather healthy population, without extremely premature children.  Preterm birth and low birth weight were both associated with lower Non-verbal IQ scores in childhood. In a recent meta-analysis over 30,000 participants, increasing birth weight and gestational age were both associated with higher IQ scores. However, the most profound effect was seen in preterm born children with a gestational age below 32 weeks (55). Our relatively healthy populations contain mostly late preterm children.  In this study, the observed effect estimates were quite strong, although not always statistically significant after correction for multiple testing. Our results are in line with results from previous studies, which show that children born preterm, low birth weight or with SGA more often have emotional, behavioural and cognitive problems. In clinical and population health settings, adverse birth outcomes should be taken into account as risk factor for emotional, behavioural and cognitive problems.  Birth characteristics are the starting point for early childhood growth. Both foetal and infant growth may have independent and synergistic effects in relation to later life outcomes. Results from a study among 11,000 children born at term and with normal birth weight from Belarus, suggest that increased birth weight and infancy weight growth was associated with higher IQ and with lower externalizing problems at age 5-6 years old (16). A review of studies that assessed the associations of birth weight and infant weight gain among non-SGA children showed no associations between growth trajectories and IQ in children (56). Another study from the United States among more than 1,000 children showed that greater infancy BMI velocity was associated with lower verbal IQ (57). We observed that children born LGA with a normal BMI in early childhood had lower internalizing problems. Furthermore, we observed that children born SGA followed by an infant BMI in the smallest tertile was associated with lower IQ. In our results we found tendencies toward a compensatory effect of growth in early childhood for adverse effects of being SGA at birth. This trend was most visible for ADHD symptoms. However, the results attenuated after correction for multiple testing was applied. Foetal and infancy development are known critical and sensitive periods in brain development. The optimum infancy weight growth in relation to birth weight is unknown and should be subject of further research.  The mechanisms linking size in early life with emotional, behavioural and cognitive outcomes might be explained by better nutritional status and related head and brain development. In children with foetal and infant growth deceleration smaller brain volumes are observed. Children with foetal growth restriction have reduced total brain volume and less brain connectivity (58). Suboptimal brain development may be associated with developmental disorders, ADHD, and ASD (59-62). Whereas children showing catch-up growth after foetal growth deceleration have been shown to have similar brain volumes as children of normal growth (63). Future studies are needed to identify whether determinants of early life growth, such as pregnancy complications, placenta insufficiency and maternal or infant nutrition explain the observed associations. Identification of birth characteristics and early life growth patterns could lead to windows of opportunity for novel strategies, such as monitoring behaviour and cognitive development in children born preterm or of low birth weight and strategies to optimize neurodevelopmental outcomes in children at risk.  Strengths of this study are the large sample size available in the infrastructure of the EU Child Cohort Network enabled us to use harmonized data from different pregnancy and childhood cohort studies. This approach enables assessment of the effect estimates in different subgroups of birth characteristics and childhood BMI, and assessment of the consistency of the associations in different populations and at different time points. Another strength is we assessed full range scores for emotional, behavioural and cognitive outcomes. This study also has some limitations, though the sample sizes are large, there is a discrepancy in sample size between the different cohorts. Data availability differed greatly between cohorts. Ideally, we would have used sex and gestational age standardized z scores for birth weight, and length standardized z-scores for weight during infancy. However, these data were not available in the in the EU Child Cohort Network database. Therefore, we adjusted the analyses focused on the associations of birth weight categories and growth patterns with childhood outcomes for sex and gestational age at birth. The emotional, behavioural and cognitive outcomes were measured using different instruments and although harmonized, are not completely homogenous. We could not take account for different respondents of the emotional, behaviour and cognitive outcomes questionnaires because this information was not completely available in the EU Child Cohort Network. Also, covariates were present in varying degrees, but missing covariates of interest were imputed. Residual confounding by various lifestyle, diet, sociodemographic or ethnic related factors cannot be excluded. Also, we did not have detailed information about other health conditions or comorbidities, which could affect physical, behavioural or cognitive development. Our study was conducted in relatively healthy children. Due to observational nature of the study, no conclusions can be drawn on the causality of the reported associations. Finally, the effect estimates may be different in clinical populations and care should be taken when extrapolating our findings to other populations. |
| Generalisability | 21 | Discuss the generalisability (external validity) of the study results | 18 | Our study was conducted in relatively healthy children. Due to observational nature of the study, no conclusions can be drawn on the causality of the reported associations. Finally, the effect estimates may be different in clinical populations and care should be taken when extrapolating our findings to other populations. |
| Other information | |  | | |
| Funding | 22 | Give the source of funding and the role of the funders for the present study and, if applicable, for the original study on which the present article is based | 12, 19-20 | **Role of the funding source**  The funding sources did not play any role in the study design, the data collection, the data analysis, the interpretation of results, the writing of the report and in the decision to submit the study for publication.  The authors are grateful to researchers and participants from the EU Child Cohort Network, ATHLETE and LifeCycle who have supported and contributed to each cohort included in this study. In addition, acknowledgments are sent to the DataSHIELD and Molgenis team. Cohort specific acknowledgements can be found in supplementary **Text S5.** This project received funding from the European Union’s Horizon 2020 research and innovation programme (LIFECYCLE, grant agreement No 733206, 2016; EUCAN-Connect grant agreement No 824989; ATHLETE, grant agreement No 874583). Cohort specific funding details can be found in supplementary **Text S6.** |

*Give information separately for cases and controls in case-control studies and, if applicable, for exposed and unexposed groups in cohort and cross-sectional studies.

**Note:** An Explanation and Elaboration article discusses each checklist item and gives methodological background and published examples of transparent reporting. The STROBE checklist is best used in conjunction with this article (freely available on the Web sites of PLoS Medicine at http://www.plosmedicine.org/, Annals of Internal Medicine at http://www.annals.org/, and Epidemiology at http://www.epidem.com/). Information on the STROBE Initiative is available at www.strobe-statement.org.
